# Synthesis and biological evaluation of oridonin derivatives as antiproliferative agents against colorectal cancer

**DOI:** 10.1039/d6md00432f

**Published:** 2026-07-14

**Authors:** Pedro J. M. Sobral, Rita I. Oliveira, Silvia Marin, Marta Cascante, Jorge A. R. Salvador

**Affiliations:** a Laboratory of Pharmaceutical Chemistry, Faculty of Pharmacy, University of Coimbra 3000-548 Coimbra Portugal salvador@ci.uc.pt +351 239 488 479; b Center for Neuroscience and Cell Biology (CNC), University of Coimbra 3004-504 Coimbra Portugal; c Center for Innovative Biomedicine and Biotechnology (CIBB), University of Coimbra 3004-504 Coimbra Portugal; d Department of Biochemistry and Molecular Biomedicine, Faculty of Biology, University of Barcelona 08028 Barcelona Spain martacascante@ub.edu +34 934 021 593; e Centro de Investigación Biomédica en Red de Enfermedades Hepáticas y Digestivas (CIBEREHD), Instituto de Salud Carlos III (ISCIII) 28029 Madrid Spain; f Institute of Biomedicine of University of Barcelona (IBUB) 08036 Barcelona Spain

## Abstract

In this study, a series of oridonin (ORI 1) derivatives were synthesized, and their antiproliferative activities were evaluated against colorectal cancer (CRC) cell lines. Structure–activity relationship (SAR) analysis revealed that aromatic and heteroaromatic substitutions significantly enhanced the antiproliferative potency compared to the parent compound. Among the synthesized derivatives, difuroate enmein-type compound 11b emerged as the most interesting candidate (IC_50_ = 0.49 μM), displaying a favorable balance between anticancer activity and cytotoxicity toward normal colon cells. Mechanistic studies revealed that compound 11b significantly inhibited the proliferation of SW620 cells, increased the doubling time, and induced S and G2/M cell cycle arrest. In addition, treatment with this compound reduced intracellular ROS levels, decreased the expression of CDK1 and CDK6, and attenuated mTOR signaling. Collectively, these findings identify compound 11b as a valuable scaffold for further development of novel anticancer agents targeting CRC.

## Abstract 2

A series of oridonin derivatives were synthesized and screened for their antiproliferative activity against colorectal cancer cell lines.

## Graphical abstract

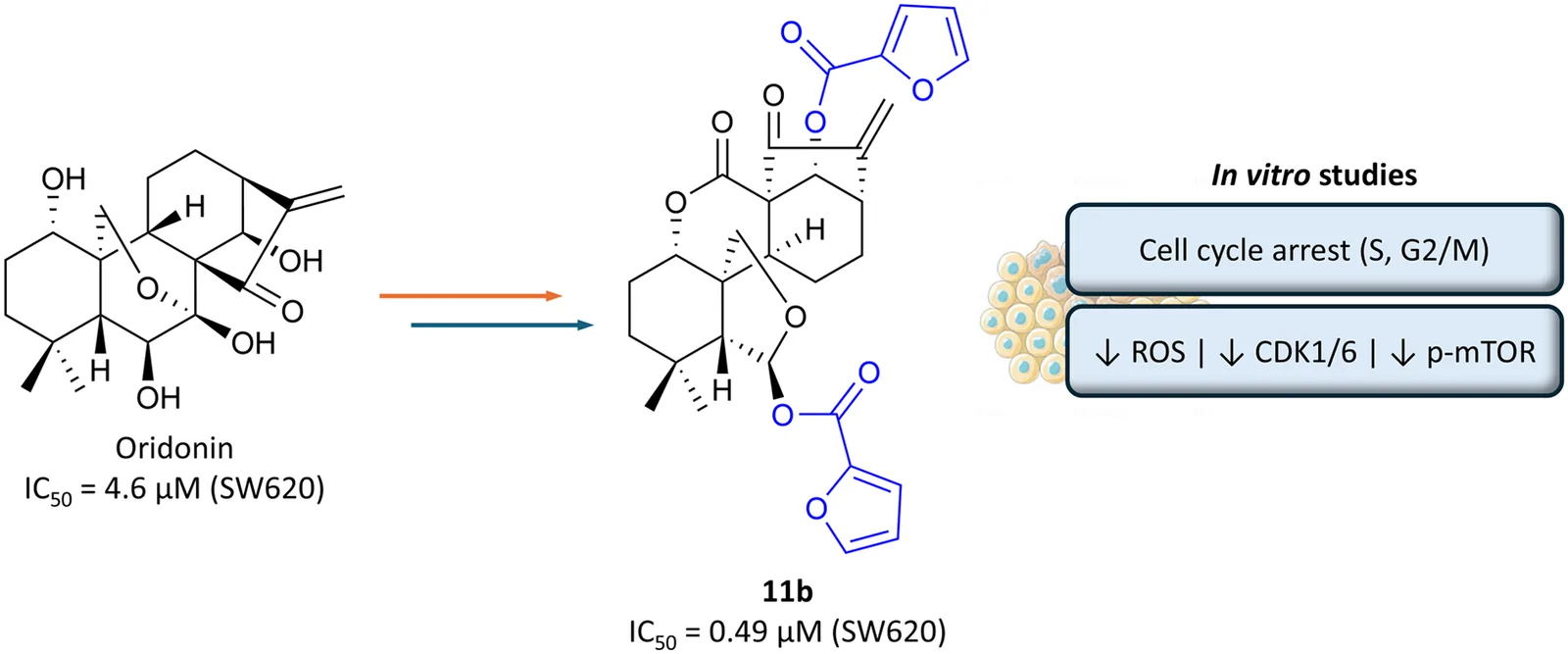

## Introduction

Colorectal cancer (CRC) is one of the most diagnosed cancers worldwide, accounting for nearly 10% of all cancer cases and deaths.^1^ Despite advances in its prevention, diagnosis, and treatment, CRC remains a major global health burden. Current treatments include surgical resection, often combined with systemic chemotherapy based on 5-fluorouracil (5-FU), oxaliplatin, or irinotecan, and targeted therapies for advanced stages.^2^ Immunotherapy has also demonstrated clinical benefits, particularly in microsatellite instability-high (MSI-H) tumors.^3^ However, these approaches are limited by systemic toxicity, acquired resistance, and variable efficacy, particularly in metastatic CRC.^4^ Therefore, the identification and optimization of promising molecular scaffolds remain important strategies for the development of novel therapeutics with improved pharmacological properties.

Natural products continue to represent a valuable source of structurally diverse compounds for drug discovery.^5,6^ Several anticancer agents have been derived from natural products, including camptothecin analogs, such as irinotecan, which remains a key component of many CRC chemotherapy regimens.^2^ Diterpenoids have provided notable therapeutic agents, particularly those belonging to the taxane family (*e.g.*, paclitaxel and docetaxel), which are widely used in oncology.^7,8^ Other diterpenoid classes, including the *ent-*kaurane group, have also attracted growing attention due to their diverse biological activities. Notably, several members of this class have shown antiproliferative activity across various cancer cell lines, supporting their potential as promising scaffolds for developing novel anticancer agents.^9,10^

**Fig. 1:**
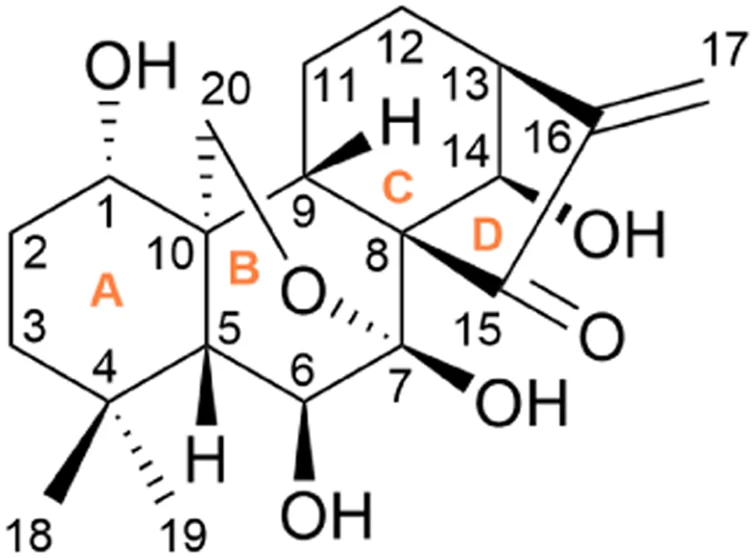
Chemical structure and numbering of oridonin (ORI 1).

*Isodon rubescens* is a traditional medicinal herb widely used in Chinese medicine for the treatment of inflammation and cancer.^11,12^ Oridonin (ORI 1) (Fig. 1), a tetracyclic *ent-*kaurane diterpenoid isolated from this plant, has been identified as one of its most important bioactive compounds and has attracted increasing attention owing to its diverse pharmacological activities, particularly its anticancer properties.^13–15^ ORI 1 is now a well-studied anticancer agent with reported activity in several cancer cell lines, including some derived from CRC.^16^ Previous studies have demonstrated that ORI 1 exerts multiple anticancer effects, including inhibition of tumor cell proliferation, induction of apoptosis and autophagy, modulation of oncogenic signaling pathways, promotion of cell-cycle arrest, and suppression of angiogenesis in CRC cell lines.^17–22^

In addition, anti-metastatic effects have been reported.^23^ ORI 1 has further been shown to overcome drug resistance mechanisms, and to induce apoptosis through activation of the ROS/JNK/c-Jun signaling axis in both 5-FU sensitive and resistant CRC cells.^24,25^ Mechanistically, the α,β-unsaturated ketone moiety of ORI 1 acts as an electrophile, reacting *via* Michael reaction with nucleophilic cysteine residues in proteins, a mechanism experimentally demonstrated for targets such as HSP70 and NLRP3.^26,27^ In addition, ORI 1 has been reported to interact directly with nucleolin through a non-covalent binding mode.^28^

Despite these promising biological activities, factors such as moderate potency, poor water solubility, and low bioavailability have hindered the clinical development of ORI 1.^29^ Consequently, extensive efforts have been focused on the structural modification of this scaffold to improve its anticancer activity and pharmacological properties.^30–33^

Among the various modification sites explored, the hydroxyl group at the C14 position has emerged as one of the most frequently functionalized sites. Numerous C14-modified derivatives have demonstrated enhanced anticancer activity across multiple cancer cell lines,^34–38^ and at least one derivative, the l-alanine-(14-oridonin) ester trifluoroacetate (HAO472), has advanced into phase I human clinical trial (CTR20150246) for the treatment of acute myelogenous leukemia.^39^ Accordingly, modifications at the C14 position continue to attract considerable interest for the structural optimization of ORI 1. In parallel, the skeletal transformation into enmein-type derivatives represents another common strategy to diversify this scaffold, which has also yielded derivatives with promising anticancer activity.^40,41^ Although this transformation alters the core skeleton, it retains key structural features of the diterpenoid, allowing subsequent functionalization at positions such as C14. However, in CRC cell lines, the relationship between specific structural modifications, whether direct on ORI 1 or following its skeletal transformation, and their biological effects remains relatively underexplored.

Our research group has extensive experience in the chemical modification and biological evaluation of terpenoid scaffolds with demonstrated anticancer activity.^42–45^ Building on this expertise, we designed and synthesized a series of derivatives based on both the original ORI 1 and enmein-type scaffolds, utilizing, among other modification sites, the C14 position, and evaluated their antiproliferative activities in CRC cell lines. The derivatives showing the highest potency were subsequently tested in additional CRC cell lines and in normal colon epithelial cells (NCM460) to assess selectivity. Among these, the most promising derivative was further investigated to elucidate the potential mechanisms underlying its anticancer activity.

## Results and discussion

### Chemistry

**Scheme 1:**
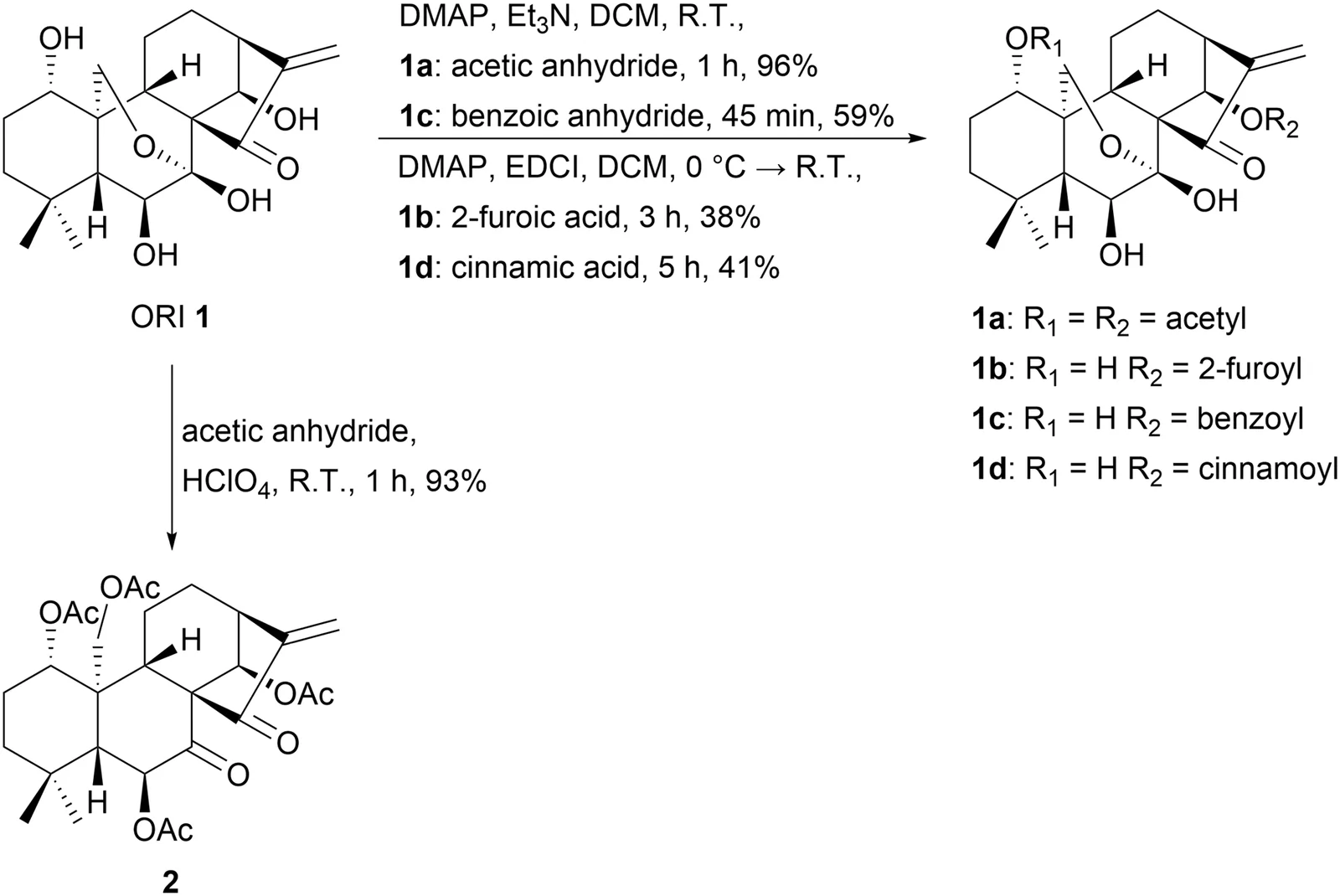
Synthesis of derivatives 1a–1d and 2 of ORI 1.

**Scheme 2:**
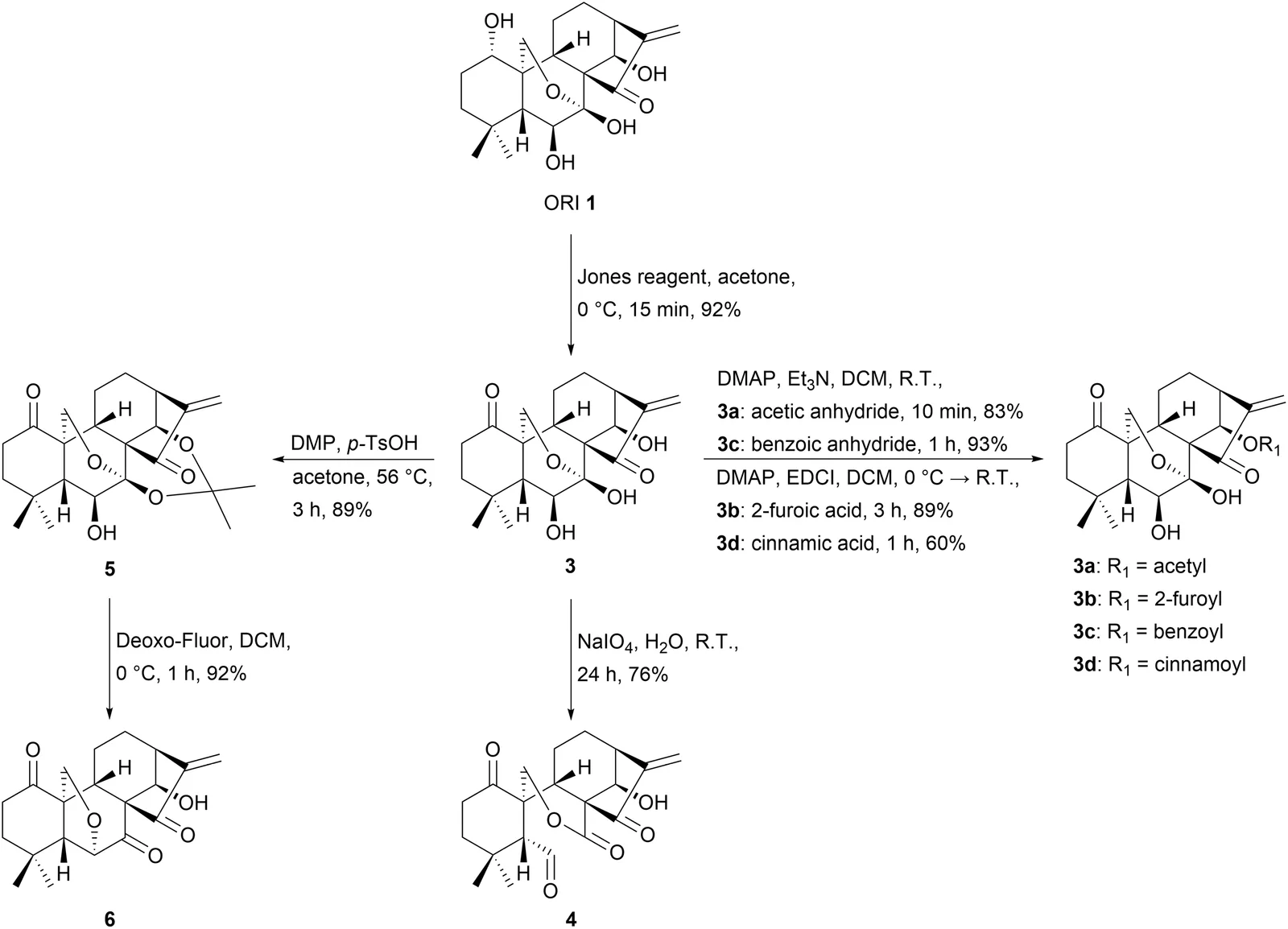
Synthesis of derivatives 3–6 and 3a–3d of ORI 1.

**Scheme 3:**
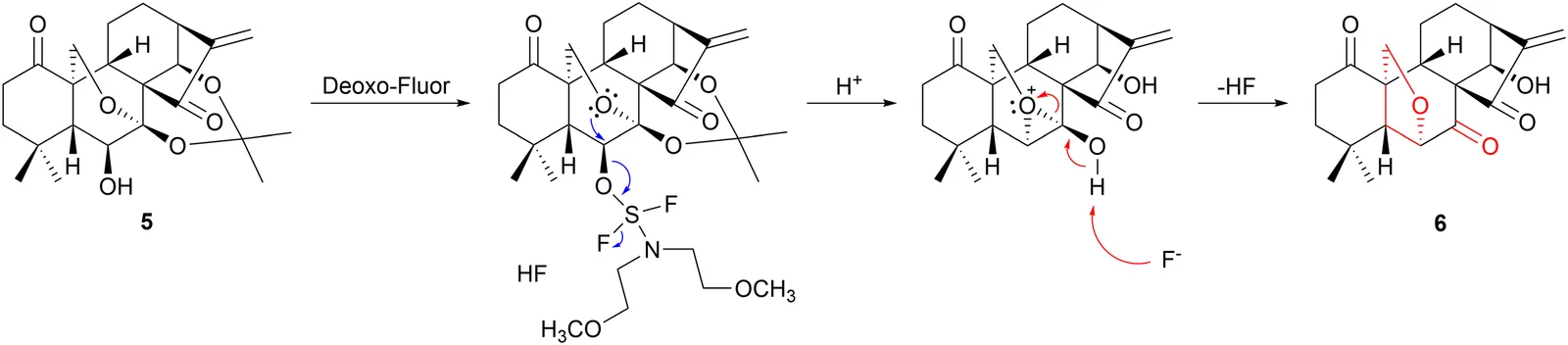
Proposed mechanism for the synthesis of compound 6.

**Scheme 4:**
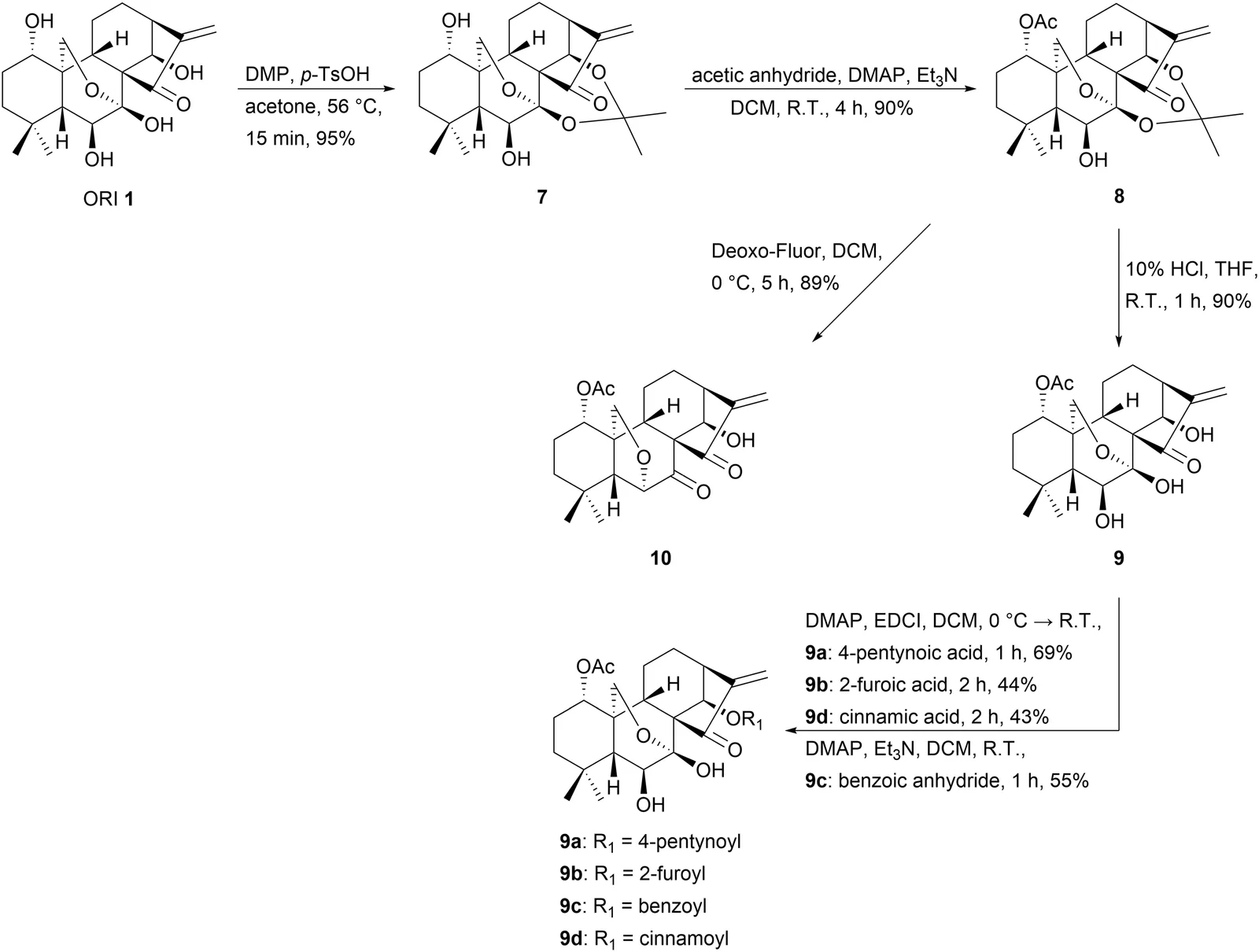
Synthesis of derivatives 7–10 and 9a–9d of ORI 1.

**Scheme 5:**
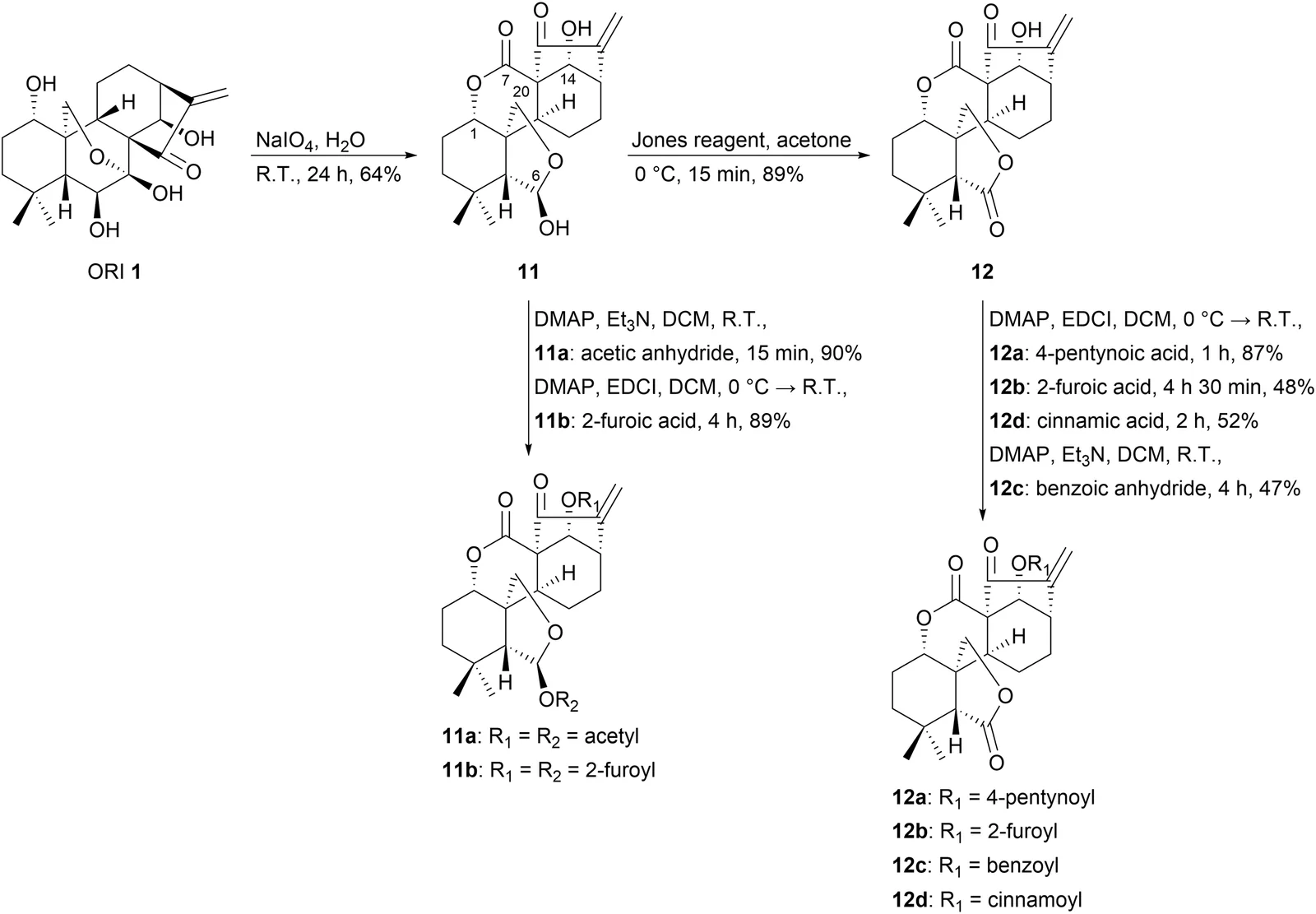
Synthesis of derivatives 11, 11a–11b, 12 and 12a–12d of ORI 1.

A series of ORI 1 derivatives were synthesized following the synthetic routes outlined in Schemes 1–5. All compounds were prepared in our laboratory and were fully characterized. The structural elucidation was confirmed by 1D (^1^H, ^13^C, and ^19^F) and 2D NMR experiments (COSY, NOESY, HSQC, and HMBC), IR spectroscopy, and mass spectrometry (MS). The purity was verified using elemental analysis. Melting points (MP) were determined after purification by preparative TLC followed by recrystallization, and the solvents used are specified in the individual characterization data. Synthetic strategies targeted either direct modification of ORI 1 or previously reported oridonin-derived scaffolds, with *O-*acylation as the main approach. To the best of our knowledge, derivatives 1b, 3b, 6, 9a–c, 11b, 12a and 12b are described herein for the first time. Previously reported compounds were also synthesized to enable comparison of their antiproliferative activities.

*O*-Acylation was performed using either acid anhydrides with triethylamine, or carboxylic acids activated with EDCI/DMAP at the C1, C6, and/or C14 positions. A structurally diverse series was prepared by introducing acetyl, alkynyl, aromatic, and heteroaromatic acyl groups. The acetyl group was included as the simplest acyl substituent of the series, whereas the terminal alkynyl group (4-pentynoyl) was included to evaluate the influence of a linear, rigid, and hydrophobic moiety.^46^ Terminal alkynyl functionalities are present in several approved anticancer drugs, including erlotinib and pralatrexate, highlighting the relevance of this functional group.^47^ Aromatic substituents, such as benzoyl and cinnamoyl, were incorporated to modulate lipophilicity and intermolecular interactions.^48^ The furoyl substituent was included to assess the impact of a heteroaromatic acyl group, commonly found in biologically active compounds, including the corticosteroid mometasone furoate.^49^

Direct *O*-acylation of ORI 1 afforded compounds 1a–1d (Scheme 1). The moderate yields observed for compounds 1b and 1d are likely attributed to competitive acylation at other hydroxyl positions of the parental core. The incorporation of the furoyl moiety (compound 1b) was confirmed by three characteristic ^1^H NMR signals at *δ* 7.55, 7.23, and 6.51 ppm (doublets of doublets, furan protons), and ^13^C NMR resonances at *δ* 147, 143, 119, and 112 ppm. Similar signals were observed for all other furoate derivatives. Treatment of ORI 1 with acetic anhydride and perchloric acid yielded compound 2, featuring a modified diterpenoid framework with C7–C20 hemiacetal ring opening.

As shown in Scheme 2, the oxidation of ORI 1 with Jones reagent in an ice-water bath selectively afforded compound 3. Subsequent *O*-acylation at C14 yielded compounds 3a–3d, allowing comparisons between directly modified and pre-oxidized scaffolds. Treatment of 3 with sodium periodate afforded the spirolactone-type compound 4, offering an alternative to the commonly reported lead(iv) acetate-mediated oxidation.^50^ Given the presence of multiple reactive hydroxyl groups in ORI 1, a protection strategy was implemented to minimize undesired reactions. Protection of the 7,14-dihydroxyl groups of compound 3 with 2,2-dimethoxypropane catalyzed by *p*-TsOH, afforded the cyclic ketal compound 5. Subsequent treatment of 5 with Deoxo-Fluor yielded compound 6. Deoxofluorination reagents are known to promote rearrangements in addition to fluorination.^51,52^ In this case, a structural rearrangement was observed, involving the migration of the hemiacetal linkage from C7 to C6, accompanied by oxidation at C7. A related rearrangement has been described using DAST;^52^ however, this transformation has not been previously reported with Deoxo-Fluor. The proposed mechanism is detailed in Scheme 3.

An alternative diversification strategy is shown in Scheme 4. Direct ketal protection afforded compound 7, followed by A-ring acetylation to produce compound 8. Deprotection regenerated the hydroxyl groups while retaining the acetyl substituent, enabling subsequent C14 *O*-acylation, to yield compounds 9a–9d. This allowed comparisons between the A-ring acetylated and non-acetylated scaffolds. The incorporation of the pentynoyl moiety (compound 9a) was confirmed by the terminal alkyne proton in the ^1^H NMR spectrum at *δ* 1.98 ppm (triplet), and the alkyne carbons in the ^13^C NMR spectrum at *δ* 82 and 69 ppm. Crucially, HMBC data confirmed the regioselective attachment at the C14 position through the key correlation between the corresponding proton and the ester carbonyl carbon (see SI). Similar signals and correlations were observed for the other pentynoate derivative. Treatment of 8 with Deoxo-Fluor afforded compound 10, consistent with the rearrangement observed for compound 6.

Finally, transformation of ORI 1 into the enmein-type scaffold 11 allowed further diversification (Scheme 5). Direct *O*-acylation at C6 and C14 produced diacetate 11a and difuroate 11b. Selective oxidation of the C6 hydroxyl yielded compound 12, which retained the free C14 hydroxyl group suitable for subsequent *O*-acylation (compounds 12a–12d). This approach enabled comparisons between the original ORI 1 and the rearranged enmein-type scaffolds. Overall, these strategies generated a diverse library of derivatives for subsequent structure–activity relationship studies in CRC cell lines.

### Biological studies

#### *In vitro* antiproliferative activity

**Fig. 2:**
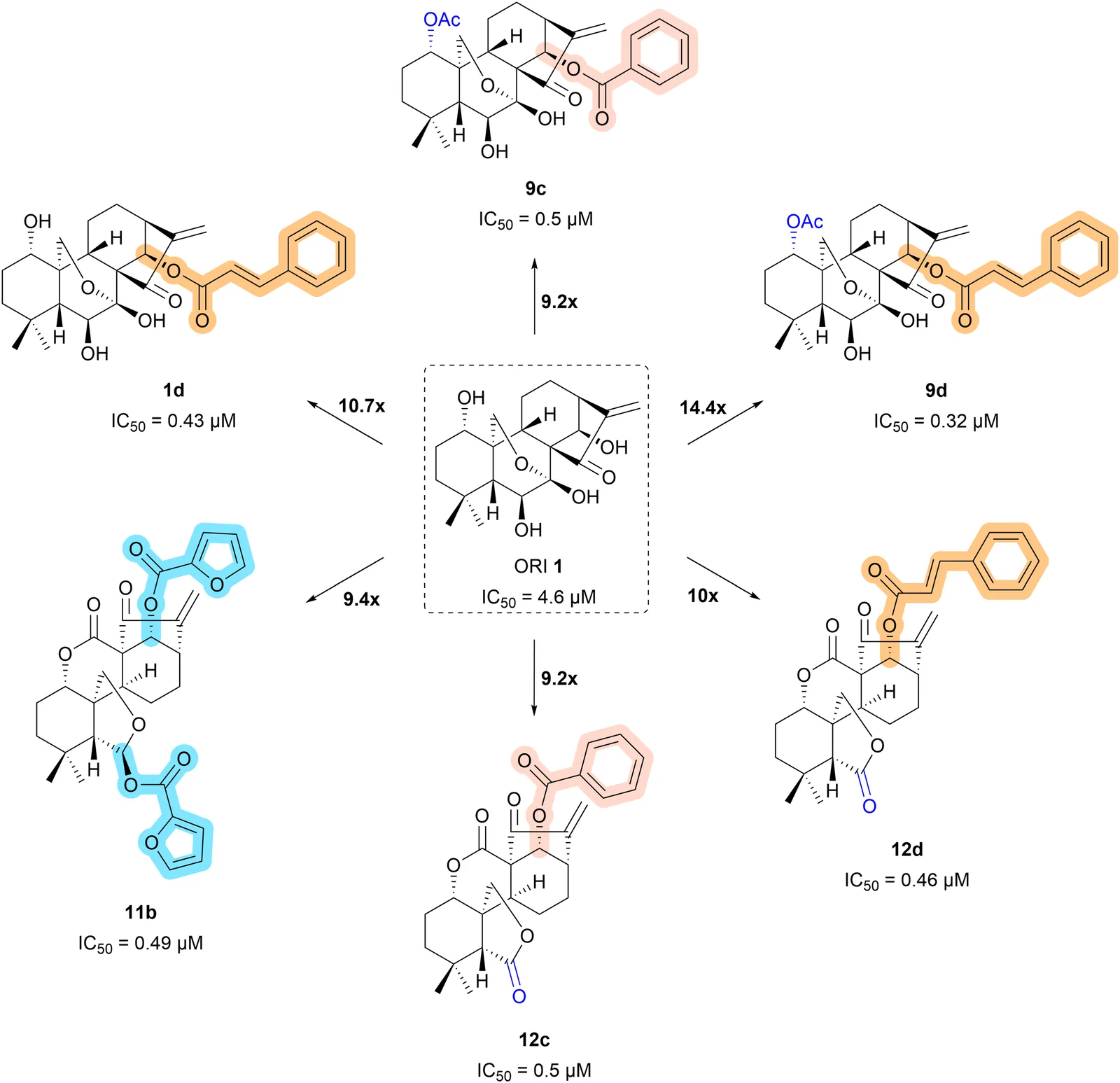
Schematic representation of the main SAR conclusions for the antiproliferative activity of *O*-acylated derivatives in SW620 cells. The SAR was established based on IC_50_ values. Aromatic and heteroaromatic substitutions exhibited higher antiproliferative potency.

The antiproliferative activities of ORI 1 and its derivatives were evaluated in the CRC cell line SW620 using the MTT assay after 72 h of treatment, with cisplatin as the reference drug. IC_50_ values were determined from the dose–response curves and are presented in Table 1. The main SAR conclusions of *O*-acylated derivatives are presented in Fig. 2. In agreement with previously reported literature, the introduction of aromatic and heteroaromatic acyl substituents conferred the greatest potency improvements.^12,30,32^ It should be noted, however, that none of these references focus on the clinical investigation of ORI 1 derivatives.

**Table 1:** IC_50_ values of ORI 1, its derivatives, and cisplatin in SW620 colorectal cancer cells^a^

Compound	Cell line/IC_50_^b^ (μM)
	SW620
ORI 1	4.6 ± 0.1
1a	1.9 ± 0.2
1b	1.8 ± 0.2
1c	0.53 ± 0.04
1d	0.43 ± 0.03
2	0.7 ± 0.1
3	2.3 ± 0.2
3a	2.4 ± 0.1
3b	1.2 ± 0.1
3c	1.2 ± 0.1
3d	0.5 ± 0.1
4	9.7 ± 0.6
6	6.8 ± 0.7
9	1.6 ± 0.2
9a	0.9 ± 0.1
9b	1.0 ± 0.1
9c	0.5 ± 0.1
9d	0.32 ± 0.02
10	4.2 ± 0.3
11	16.8 ± 0.5
11a	1.6 ± 0.2
11b	0.49 ± 0.02
12	5.1 ± 0.4
12a	1.3 ± 0.1
12b	1.0 ± 0.1
12c	0.5 ± 0.1
12d	0.46 ± 0.03
Cisplatin	1.4 ± 0.5^c^ (ref. 53)

a After 72 h of incubation with increasing concentrations of each compound, IC_50_ values were determined using the MTT assay. Data are expressed as mean ± SD from three independent experiments, each performed in technical triplicate.

b IC_50_ values represent the compound concentration necessary to inhibit 50% of cell viability.

c IC_50_ value was previously obtained by our research group using the same method.

ORI 1 exhibited moderate antiproliferative activity (IC_50_ = 4.6 μM). All directly *O*-acylated derivatives exhibited enhanced potency relative to the parent compound, with IC_50_ values ranging from 0.43 to 1.9 μM. The benzoate (1c, IC_50_ = 0.53 μM) and cinnamate (1d, IC_50_ = 0.43 μM) derivatives were more potent than the diacetate (1a, IC_50_ = 1.9 μM) and furoate derivatives (1b, IC_50_ = 1.8 μM). Compound 2, a tetraacetate derivative featuring the opening of the C7–C20 hemiacetal ring, also displayed enhanced activity (IC_50_ = 0.7 μM), indicating that *O*-acylation can improve potency even within structurally modified scaffolds.

A-ring oxidation of ORI 1 afforded compound 3 (IC_50_ = 2.3 μM), which exhibited improved antiproliferative activity relative to the parent compound. Subsequent C14 *O*-acylation of this oxidized scaffold led to a different structure–activity relationship from that observed for derivatives of the parent compound. The acetate derivative did not further enhance potency (3a, IC_50_ = 2.4 μM), whereas the furoate (3b) and benzoate (3c) derivatives displayed IC_50_ values of 1.2 μM. The cinnamate derivative 3d exhibited the highest potency within this subset (IC_50_ = 0.5 μM). A comparison with the corresponding derivatives obtained directly from ORI 1 indicates that this oxidation step does not systematically enhance the activity across the series. Compound 3b was slightly more potent than its non-oxidized counterpart 1b. However, for the benzoate and cinnamate derivatives, the compounds obtained directly from ORI 1 (1c and 1d) remained more active than their oxidized analogs (3c and 3d). These findings suggest that although A-ring oxidation improves the baseline activity of the scaffold, the nature of the C14 acyl substituent plays a more decisive role in determining the antiproliferative potency. In contrast, the spirolactone-type derivative 4 exhibited reduced activity (IC_50_ = 9.7 μM), and the rearranged derivative 6 was also less active than ORI 1 (IC_50_ = 6.8 μM).

The A-ring acetylated scaffold (compound 9) displayed an IC_50_ value of 1.6 μM, exhibiting improved antiproliferative activity compared to both ORI 1 and oxidized scaffold 3. Subsequent C14 *O*-acylation afforded compounds 9a–9d, among which the cinnamate derivative 9d emerged as the most potent compound in the series (IC_50_ = 0.32 μM). The furoate (9b, IC_50_ = 1.0 μM) and benzoate (9c, IC_50_ = 0.5 μM) derivatives also showed enhanced activity relative to their corresponding analogs derived directly from ORI 1 (1b–1c) or from the oxidized scaffold 3 (3b–3c), suggesting that A-ring acetylation may favorably influence subsequent C14 functionalization. The terminal alkynyl derivative 9a also exhibited submicromolar potency (IC_50_ = 0.9 μM). In contrast, rearranged compound 10 (IC_50_ = 4.2 μM) displayed activity comparable to that of the parent compound, consistent with the reduced potency observed for the related rearranged compound 6.

Transformation of ORI 1 into the corresponding enmein-type compound 11 resulted in a significant loss of antiproliferative activity (IC_50_ = 16.8 μM). However, subsequent *O*-acylation at the C6 and C14 positions restored potency, with the diacetate 11a displaying an IC_50_ of 1.6 μM and the difuroate 11b showing substantially enhanced activity (IC_50_ = 0.49 μM). The oxidation of compound 11 afforded compound 12 (IC_50_ = 5.1 μM). Further C14 *O*-acylation improved potency, particularly for the benzoate (12c, IC_50_ = 0.5 μM) and cinnamate (12d, IC_50_ = 0.46 μM) derivatives, whereas the pentynoate (12a, IC_50_ = 1.3 μM) and furoate (12b, IC_50_ = 1.0 μM) derivatives showed more moderate activity. Overall, these findings indicate that both scaffold modification and the nature of the substituent jointly influence antiproliferative activity.

Based on their activity in SW620 cells, selected derivatives were further evaluated in additional CRC cell lines, HCT116 and HT29 (Table 2). The chosen compounds represent the most active substituent classes identified in the initial screening, namely, the furoate 9b, benzoate 9c, cinnamate 9d, and difuroate enmein-type 11b derivatives. The chosen cell lines are genetically distinct: HCT116 is a microsatellite instability-high (MSI-H), whereas SW620 and HT29 are microsatellite-stable (MSS), and they differ in key oncogenic alterations, including *KRAS* and *TP53* mutation status. Testing the selected derivatives under these conditions enabled us to assess their activities in genetically distinct CRC cell lines.

**Table 2:** IC_50_ values of ORI 1, its derivatives (9b–9d and 11b), and cisplatin in HCT116 and HT29 colorectal cancer cell lines, and in the non-tumoral human colon epithelial cell line (NCM460)^a^

Compound	Cell line/IC_50_^b^ (μM)		
	Colorectal cancer		Non-tumoral
	HCT116	HT29	NCM460
ORI 1	5.9 ± 0.3	12.6 ± 0.3	7.6 ± 0.8
9b	1.1 ± 0.1	1.8 ± 0.2	N.D.
9c	1.0 ± 0.1	1.3 ± 0.2	N.D.
9d	0.57 ± 0.02	0.7 ± 0.1	0.38 ± 0.02
11b	0.8 ± 0.1	1.3 ± 0.2	1.9 ± 0.2
Cisplatin	21.1 ± 1.3^c^ (ref. 54)	6.1^d^ (ref. 55)	N.D.

a After 72 h of incubation with increasing concentrations of each compound, IC_50_ values were determined using the MTT assay. Data are expressed as mean ± SD from three independent experiments, each performed in technical triplicate. N.D.: not determined.

b IC_50_ values represent the compound concentration necessary to inhibit 50% of cell viability.

c IC_50_ value was previously obtained by our research group using the same method.

d IC_50_ value was acquired from literature by applying the same method.

Across both additional cell lines, compound 9d displayed the highest potency, whereas 11b remained the most active furoate derivative and the second most potent compound overall. Therefore, to determine the selectivity of the two most promising derivatives (9d and 11b), they were evaluated in the normal colon epithelial cell line NCM460 (Table 2). Although 9d was the most potent compound, it exhibited limited selectivity (SI = 1.19), which was even lower than that of ORI 1 (SI = 1.65). In contrast, compound 11b showed improved selectivity (SI = 3.88), indicating a more favorable balance between anticancer potency and cytotoxicity toward normal cells.

**Fig. 3:**
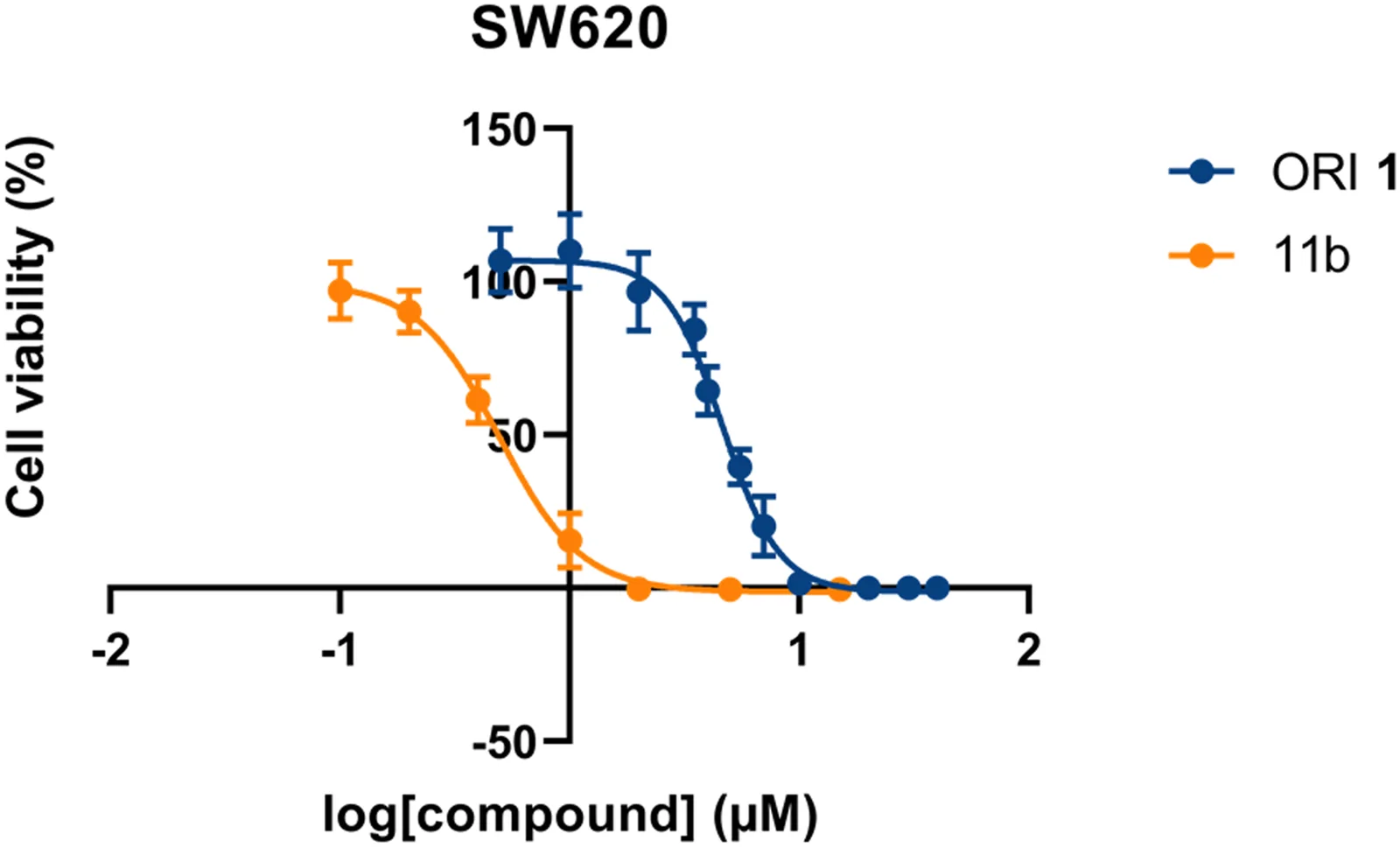
Dose–response curves of ORI 1 (IC_50_ = 4.6 μM) and compound 11b (IC_50_ = 0.49 μM) in SW620 cells after 72 h of treatment. Cell viability was assessed using the MTT assay. Data are expressed as mean ± SD from three independent experiments, each performed in technical triplicate.

Based on these results, compound 11b was selected for further mechanistic studies in SW620 cells because it exhibited the highest potency in this cell line (Fig. 3). Moreover, SW620 cells are MSS and represent the predominant CRC subtype that is treated with conventional chemotherapy.

Preliminary *in silico* ADME predictions were performed using the SwissADME platform, and the detailed reports for ORI 1 and compound 11b are provided in the SI. The data show that compound 11b has increased lipophilicity compared to ORI 1 (consensus log *P*_o/w_ = 3.61 *vs.* 0.64).

Regarding the alkynyl-containing derivatives 9a and 12a, they may be suitable for probe development and target identification studies. While the present work focuses on compound 11b, developing these alkyne-functionalized derivatives into active probes, aligned with the activity-based protein profiling (ABPP) platforms reported by Cravatt and co-workers,^56^ could allow chemoproteomic studies to map cellular targets in CRC cells.

#### Analysis of cell apoptosis

Apoptosis is a regulated form of programmed cell death, and its evasion is a hallmark of cancer that contributes to tumor progression and therapeutic resistance. Many anticancer agents exert part of their cytotoxic effect by activating apoptotic pathways.^57^ To investigate whether the antiproliferative effects of compound 11b were associated with apoptosis induction, phosphatidylserine (PS) externalization and membrane integrity were evaluated by Annexin V-FITC/propidium iodide (PI) staining followed by flow cytometry. In viable cells, PS is confined to the inner leaflet of the plasma membrane, whereas during early apoptosis, it becomes exposed on the cell surface and binds to Annexin V. PI is excluded from viable and early apoptotic cells but enters cells with compromised membrane integrity, characteristic of late apoptosis or necrosis, where it intercalates with DNA. This approach allows for discrimination among viable (Annexin V−/PI−), early apoptotic (Annexin V+/PI−), and late apoptotic/necrotic (Annexin V+/PI+) populations.^58^

**Fig. 4:**
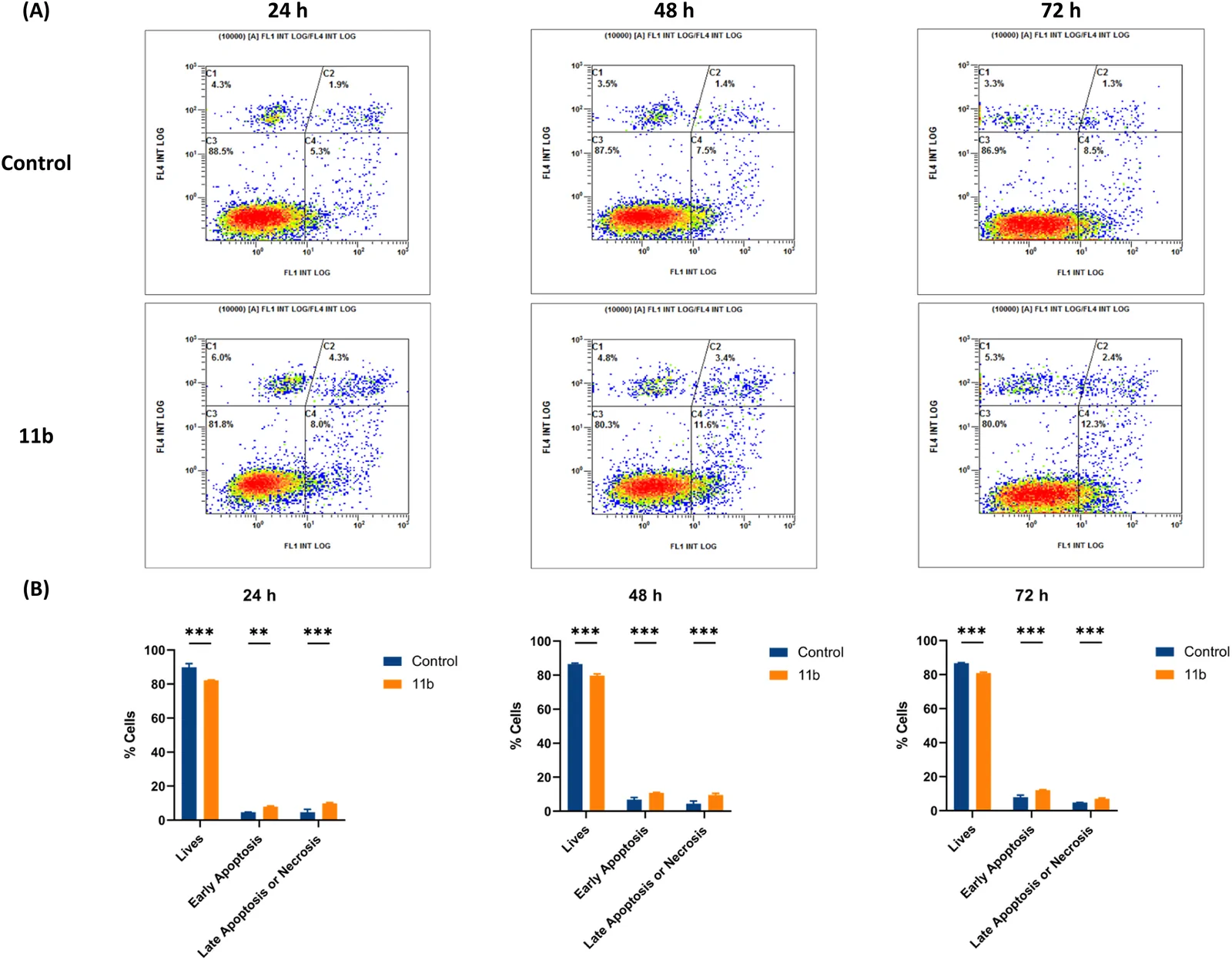
Apoptosis analysis of SW620 cells untreated or treated with compound 11b at its IC_50_ concentration for 24, 48, and 72 h, followed by Annexin V-FITC/PI staining and flow cytometry. (A) Representative dot plots illustrating the distribution of live cells (lower left quadrant, C3), early apoptotic cells (lower right quadrant, C4), and late apoptotic/necrotic cells (upper quadrants, C1 + C2). (B) Quantitative analysis showing the percentage of viable, early apoptotic, and late apoptotic/necrotic populations. Data are expressed as mean ± SD from three independent experiments, each performed in technical triplicate. Statistical differences between control and treated groups were evaluated using multiple *t*-tests (one independent *t*-test per row). *p* < 0.01 (**), *p* < 0.001 (***).

SW620 cells were treated with compound 11b at its IC_50_ concentration for 24, 48, and 72 h, followed by Annexin V-FITC/PI staining and flow cytometric analysis. At 24 h, a modest increase in both early apoptotic and late apoptotic/necrotic cell populations was observed compared with control cells. This trend persisted at 48 and 72 h, although the magnitude of the effect remained limited throughout the time course. Despite the fact that a statistically significant reduction in the viable cell population was observed at all time points, only a small fraction of cells underwent apoptosis upon treatment with compound 11b, showing that apoptosis is not the predominant mechanism underlying its antiproliferative effects in SW620 cells (Fig. 4).

#### Analysis of cell cycle and growth kinetics

The cell cycle is a coordinated process that regulates cellular growth and division, and its disruption is a hallmark of cancer initiation and progression. Many anticancer agents induce cell cycle arrest or alter its progression. Therefore, cell cycle distribution analysis is commonly used to elucidate the mechanisms underlying the antiproliferative effects of novel compounds.^59,60^

As apoptosis induction by compound 11b was relatively modest in SW620 cells, we investigated whether its antiproliferative activity was associated with alterations in cell cycle progression. For this purpose, SW620 cells were exposed to compound 11b at its IC_50_ concentration for 24, 48, and 72 h. Cells were subsequently fixed and permeabilized with cold 70% ethanol (v/v), stained with propidium iodide (PI) in the presence of RNase A, and analyzed by flow cytometry to determine DNA content and cell cycle phase distribution.

**Fig. 5:**
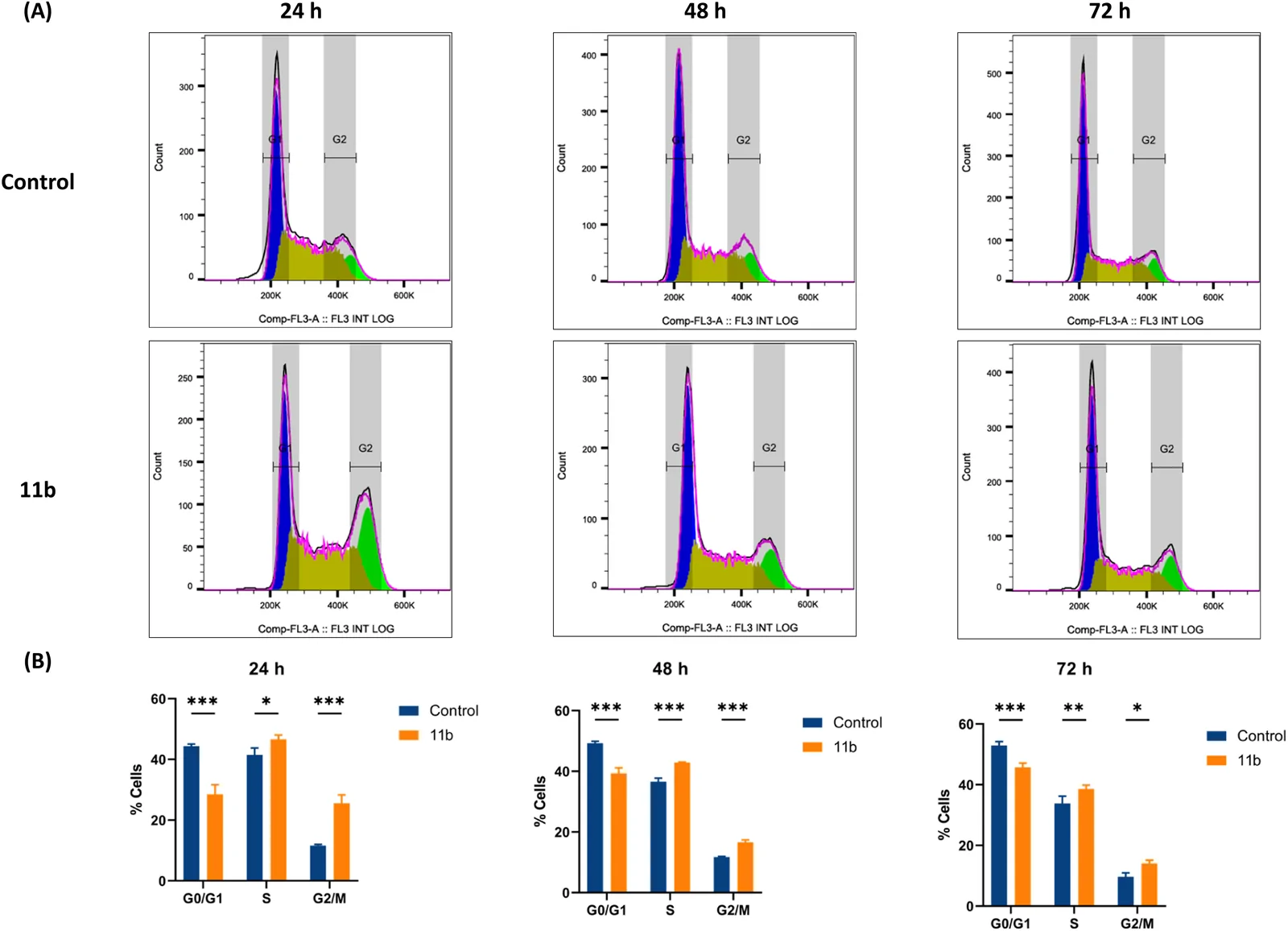
Cell cycle analysis of SW620 cells untreated or treated with compound 11b at its IC_50_ concentration for 24, 48, and 72 h. At each time point, cells were fixed and permeabilized with cold 70% ethanol (v/v), stained with propidium iodide (PI) in the presence of RNase A, and DNA content was analyzed by flow cytometry. (A) Representative histograms showing the distribution of cells across the G0/G1 (blue area), S (yellow area), and G2/M (green area) phases. (B) Quantitative analysis of the percentage of cells in each cell cycle phase. Data are expressed as mean ± SD from three independent experiments, each performed in technical triplicate. Statistical differences between control and treated groups were evaluated using two-way ANOVA (Sidak's *post hoc* comparison). *p* < 0.05 (*), *p* < 0.01 (**), *p* < 0.001 (***).

As shown in Fig. 5, treatment with compound 11b induced an accumulation of cells in the S and G2/M phases after 24 h, with a particularly pronounced increase in the G2/M population. Specifically, the proportion of cells in G2/M increased from 11.6% in the control cells to 25.6% upon treatment. This pattern persisted at later time points. At 48 and 72 h, cells exposed to compound 11b continued to display higher proportions in the S and G2/M phases than control cells, indicating a sustained perturbation of cell cycle progression.

**Fig. 6:**
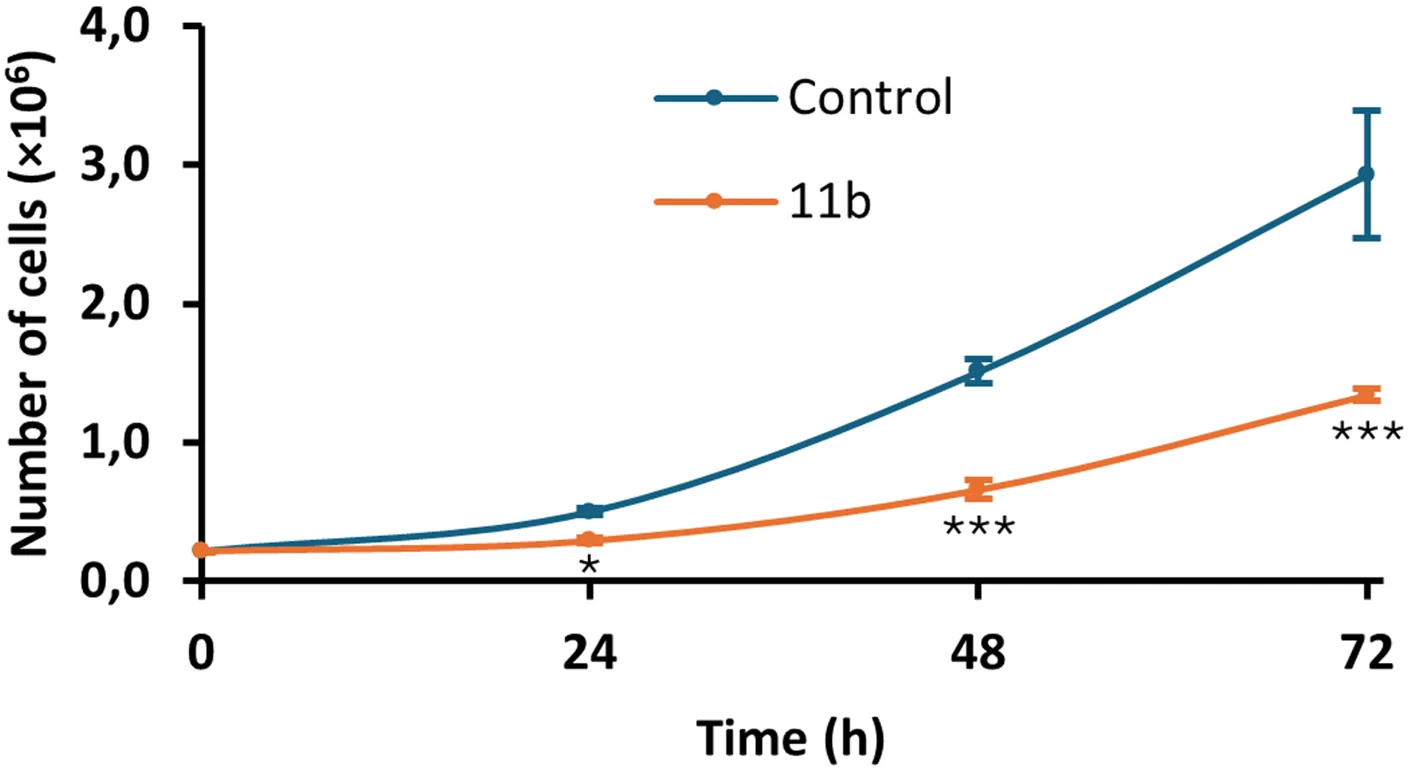
Time-dependent growth curves of SW620 cells untreated or treated with compound 11b at its IC_50_ concentration for 24, 48 and 72 h. At each time point, cells were collected and viable cells were counted. Data are expressed as mean ± SD from three independent experiments, each performed in technical triplicate. Statistical differences between control and treated groups were evaluated using multiple *t*-tests (one independent *t*-test per row). *p* < 0.05 (*), *p* < 0.001 (***).

To analyze the impact of compound 11b on cell proliferation, the population doubling time was determined in SW620 cells (Fig. 6). Cell doubling time is commonly used as an indicator of the cell growth rate and was calculated by dividing the natural logarithm of 2 by the growth rate.^61^ Control cells displayed a doubling time of 27.0 ± 1.73 h over the 0–72 h interval, consistent with the highly proliferative phenotype of this metastatic CRC cell line. To fully characterize the effect of compound 11b, the population doubling time was calculated for two distinct time intervals (0–24 h and 24–72 h). During the first 24 h of treatment, compound 11b markedly increased the doubling time to 81.4 h, indicating a pronounced early antiproliferative effect. At later time points (24–72 h), treated cells exhibited a shorter doubling time (31.5 ± 1.31 h), although it remained longer than that of control cells, indicating sustained growth inhibition. This time-dependent analysis was performed to capture the early proliferative changes observed in the cell cycle analysis, where the most pronounced accumulation of cells in the G2/M phase occurred at 24 h of treatment.

Overall, SW620 cells treated with compound 11b exhibited a slower growth rate over time than untreated cells, as reflected by the increased population doubling time.

**Fig. 7:**
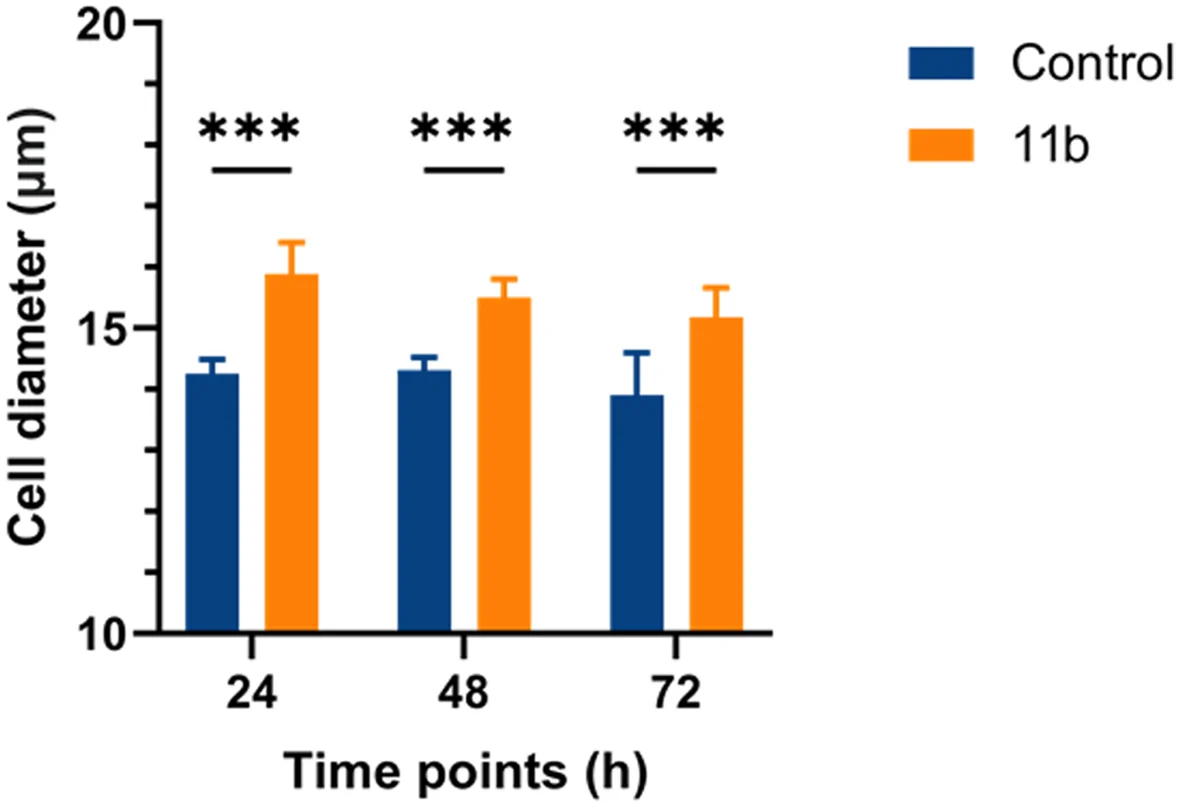
Cell diameter analysis of SW620 cells untreated or treated with compound 11b at its IC_50_ concentration for 24, 48 and 72 h. Cell diameter (μm) was measured at each time point. Data are expressed as mean ± SD from three independent experiments, each performed in technical triplicate. Statistical differences between control and treated groups were evaluated using multiple *t*-tests (one independent *t*-test per row). *p* < 0.001 (***).

To further evaluate the effect of 11b on cellular growth, changes in cell diameter were monitored over time. Cell diameter reflects cellular growth status, and increases in cell size have been associated with growth arrest and metabolic adaptation in cancer cells.^62,63^ Therefore, measuring the cell diameter can provide additional information regarding the biological effects of compound 11b. As shown in Fig. 7, treated SW620 cells exhibited larger diameters (μm) than control cells at 24, 48, and 72 h. This increase is consistent with the accumulation of cells in the G2/M phase, during which they reach their maximum size prior to mitosis.

These results, together with the prolonged doubling time, support the G2/M arrest observed.

#### Recovery assay

Since the most pronounced cell cycle arrest was observed at 24 h of treatment, a recovery assay was conducted to evaluate whether this effect was reversible. SW620 cells were treated with compound 11b at its IC_50_ concentration for 24 h. After treatment, the medium containing the compound was removed, cells were washed with DPBS, and fresh, compound-free medium was added. Cells were then allowed to recover for an additional 24 and 48 h, after which viable cells were collected and counted.

**Fig. 8:**
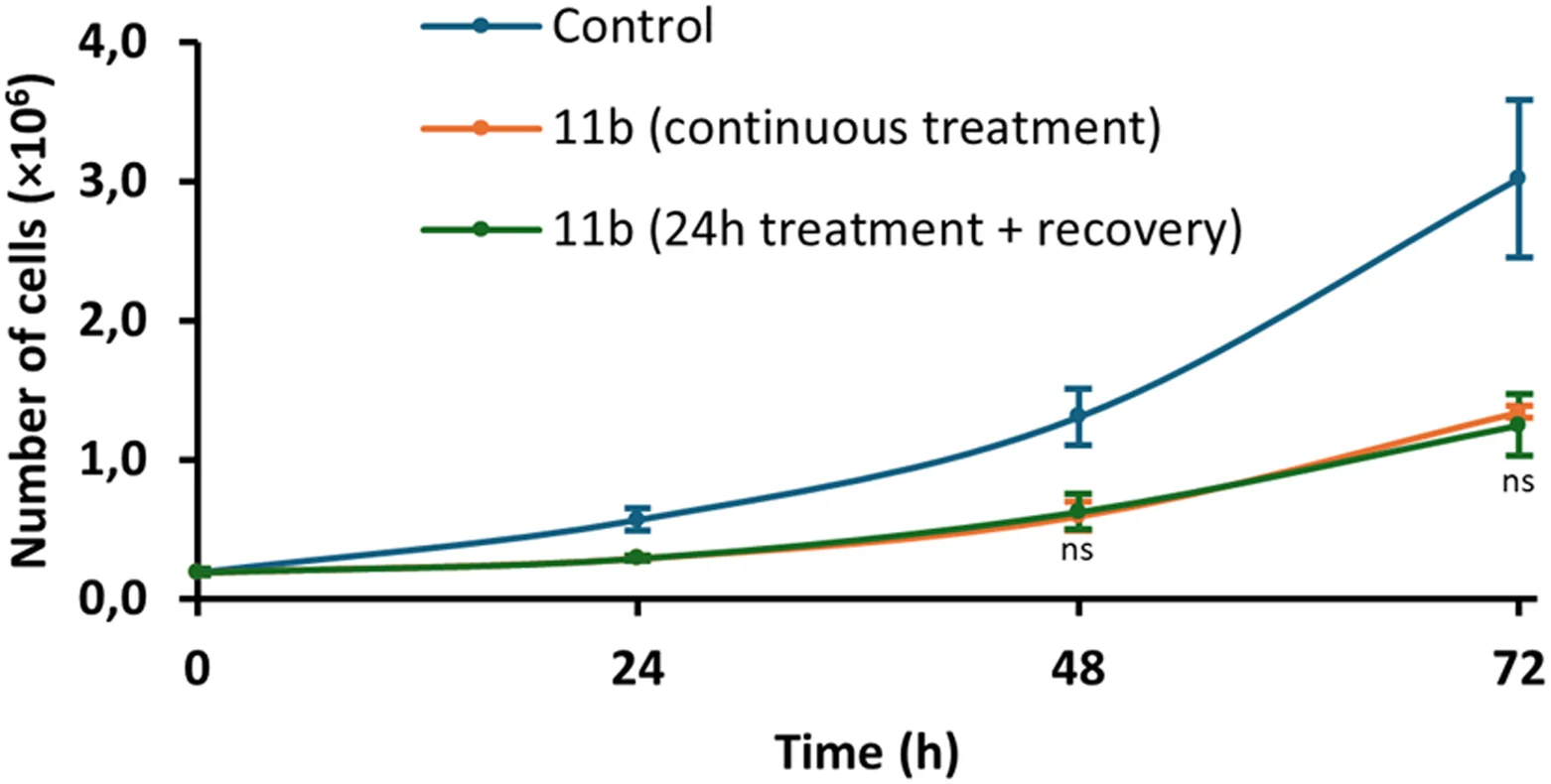
Time-dependent growth curves of SW620 cells untreated, continuously treated with 11b at its IC_50_ concentration, or treated with 11b at its IC_50_ concentration for 24 h, followed by recovery in compound-free medium for an additional 24 and 48 h. At each time point, cells were collected and viable cells were counted. Data are expressed as mean ± SD from three independent experiments, each performed in technical triplicate. Statistical differences between continuously treated and recovery-treated cells were evaluated using multiple *t*-tests (one independent *t*-test per row). Ns, not significant.

As shown in Fig. 8, SW620 cells failed to resume proliferation following compound removal. These results indicate that a 24 h exposure to compound 11b leads to a sustained inhibition of cell proliferation.

#### Analysis of ROS generation

Reactive oxygen species (ROS) are highly reactive molecules produced as byproducts of cellular metabolism and play an essential role in maintaining intracellular redox homeostasis. Dysregulation of ROS levels is associated with cancer development and progression.^64^ In cancer biology, ROS play a dual role. Moderate increases in ROS can act as signaling mediators that promote cell survival and proliferation. In contrast, excessive ROS accumulation leads to oxidative stress, resulting in cellular damage and cell death. Conversely, reduced intracellular ROS levels may disrupt redox-regulated signaling pathways and impair the proliferative capacity of cancer cells.^65,66^ Therefore, the modulation of cellular redox homeostasis represents an important mechanism through which several anticancer agents exert their biological effects.^67,68^

**Fig. 9:**
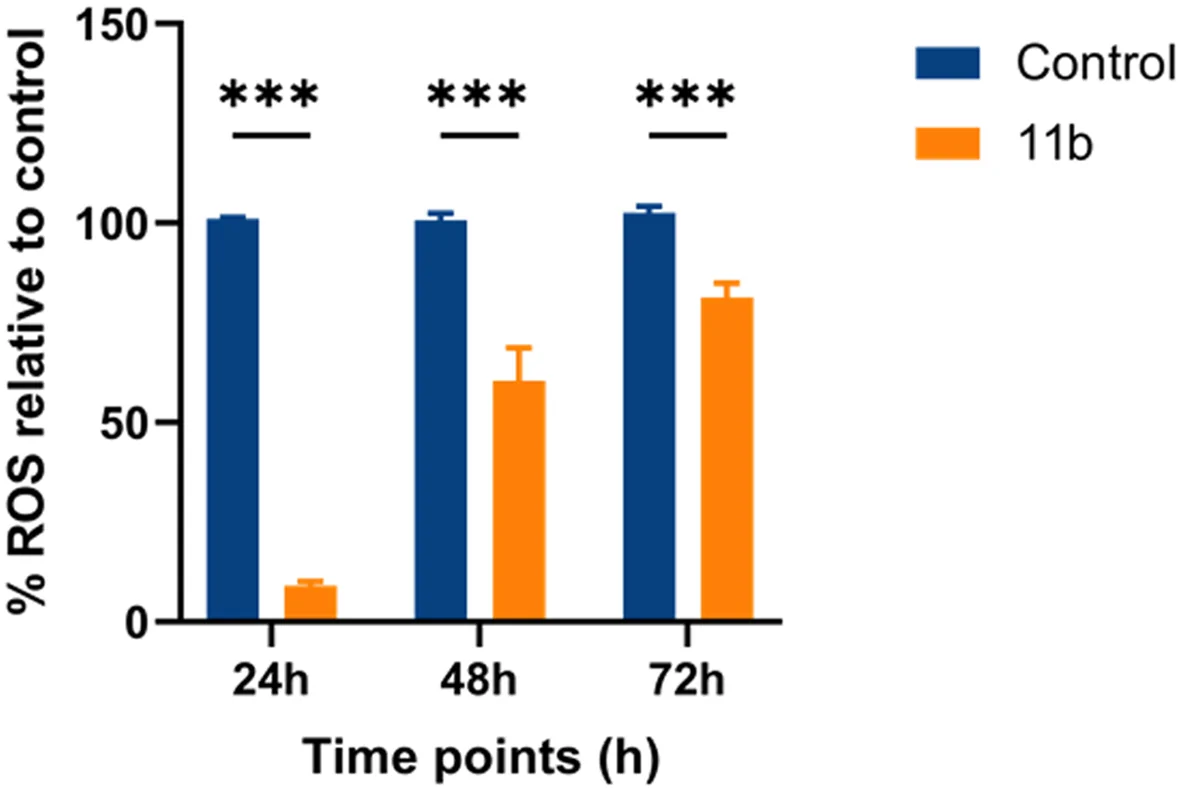
Intracellular ROS levels in SW620 cells untreated or treated with compound 11b at its IC_50_ concentration for 24, 48, and 72 h. ROS levels were quantified using the H_2_DCFDA fluorescent probe and normalized to cell number. Data are expressed as mean ± SD from three independent experiments, each performed in technical triplicate. Statistical differences between control and treated groups were evaluated using multiple *t*-tests (one independent *t*-test per row). *p* < 0.001 (***).

The effect of compound 11b on intracellular ROS levels was investigated using the fluorescent probe 2′,7′-dichlorodihydrofluorescein diacetate (H_2_DCFDA). SW620 cells were treated with 11b at its IC_50_ concentration, and ROS production was assessed at 24, 48, and 72 h, following a previously reported method from our research group.^69^ ROS fluorescence signals were normalized to the cell number to account for potential differences in cell density and viability. As shown in Fig. 9, treatment with 11b significantly reduced intracellular ROS levels compared to the control, with the strongest effect observed at 24 h. At later time points, ROS levels gradually increased but remained lower than those of the control cells, suggesting partial redox adaptation over time.

These findings indicate that compound 11b exerts a pronounced antioxidant effect in SW620 cells, which may contribute to the observed cell cycle arrest and reduced proliferative capacity.

#### Western blot analysis of cell cycle and signaling-related proteins

Cell cycle, cell proliferation, and ROS analyses revealed that compound 11b exerts its most pronounced antiproliferative effects during the first 24 h of treatment. Consequently, all Western blot experiments were performed at the 24 h time point.

**Fig. 10:**
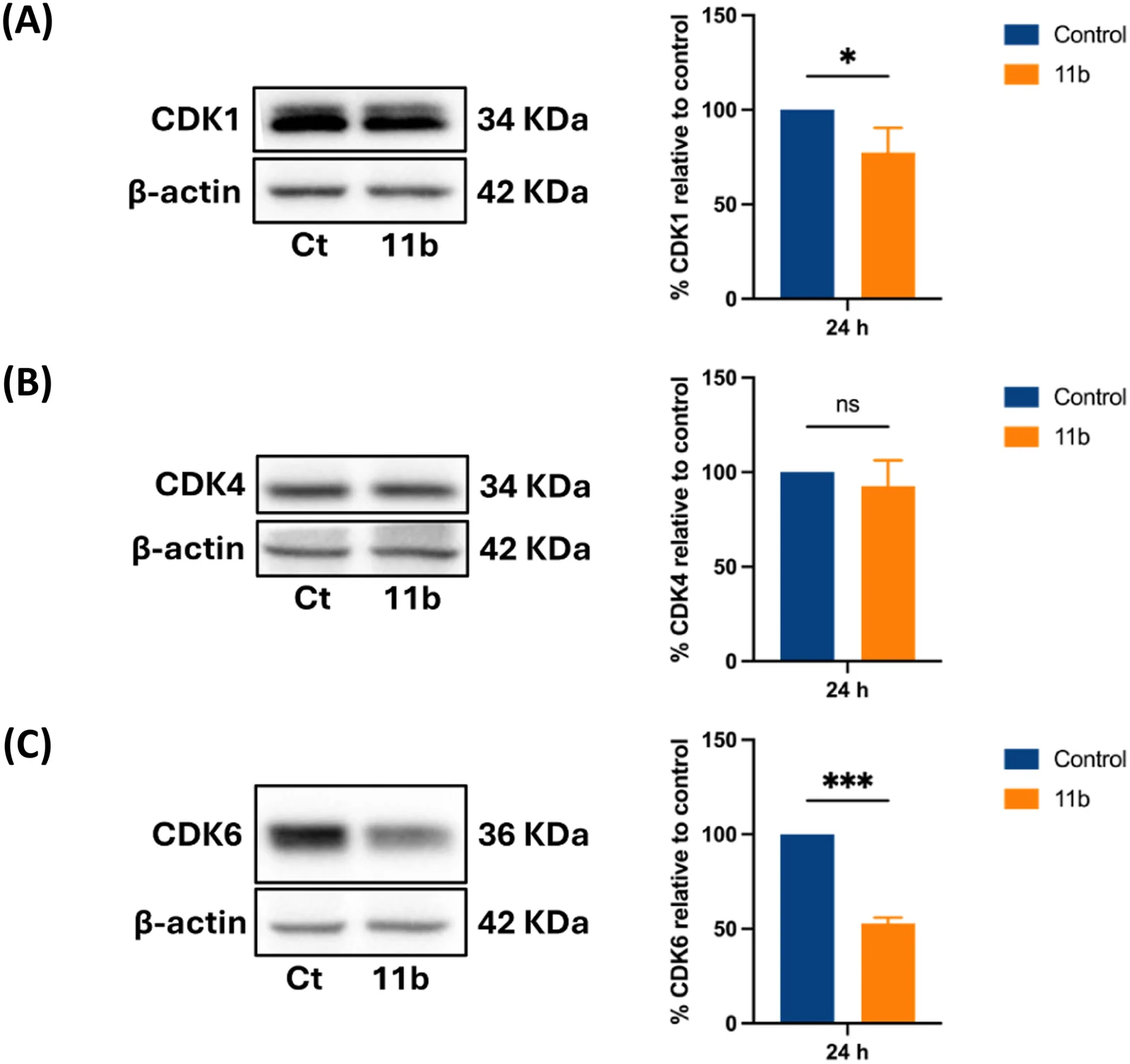
Expression levels of (A) CDK1, (B) CDK4, and (C) CDK6 in SW620 cells untreated or treated with compound 11b at its IC_50_ concentration for 24 h. Protein levels were analyzed by Western blot, with β-actin used as a loading control. Band intensities were quantified and normalized to β-actin. Bar graphs represent the relative protein expression levels expressed as percentage of control. Data are expressed as mean ± SD from three independent experiments. Statistical analysis was performed using Student's *t*-test. *p* < 0.05 (*), *p* < 0.001 (***), ns, not significant.

Cell cycle progression is regulated by cyclin-dependent kinases (CDKs) and their cyclin partners, which orchestrate transitions between specific cell cycle phases. To explore the molecular mechanisms underlying the cell cycle arrest induced by compound 11b, the expression levels of CDK1, CDK4, and CDK6 were examined by Western blotting. CDK1 serves as a critical regulator of the G2/M transition, whereas CDK4 and CDK6 primarily regulate progression through the G1 phase and entry into the S phase.^59^ As shown in Fig. 10, treatment with compound 11b decreased CDK1 expression to 77% of the control level, whereas CDK4 expression remained unaffected. In addition, a marked decrease in CDK6 expression to 53% of the control level was also observed. Given the established role of CDK6 in G1 phase progression, this effect is unlikely to be directly associated with the predominant G2/M arrest, although it may be consistent with the additional perturbation observed in the S phase. Instead, the downregulation of CDK6 may reflect a broader antiproliferative effect of the compound on cell cycle regulatory pathways.

Cell proliferation and survival are regulated by intracellular signaling pathways, among which the PI3K/Akt/mTOR axis plays a central role in coordinating cellular growth, metabolism, and cell cycle progression. Dysregulation of this pathway is frequently observed in cancer and contributes to uncontrolled proliferation and chemoresistance.^70,71^ In addition, the transcription factor c-Myc regulates the expression of numerous genes involved in these processes, further promoting tumor progression.^72^ To investigate whether the antiproliferative effects of compound 11b were associated with alterations in these signaling pathways, the protein levels and phosphorylation status of Akt and mTOR, as well as the expression of c-Myc, were analyzed by Western blotting.

**Fig. 11:**
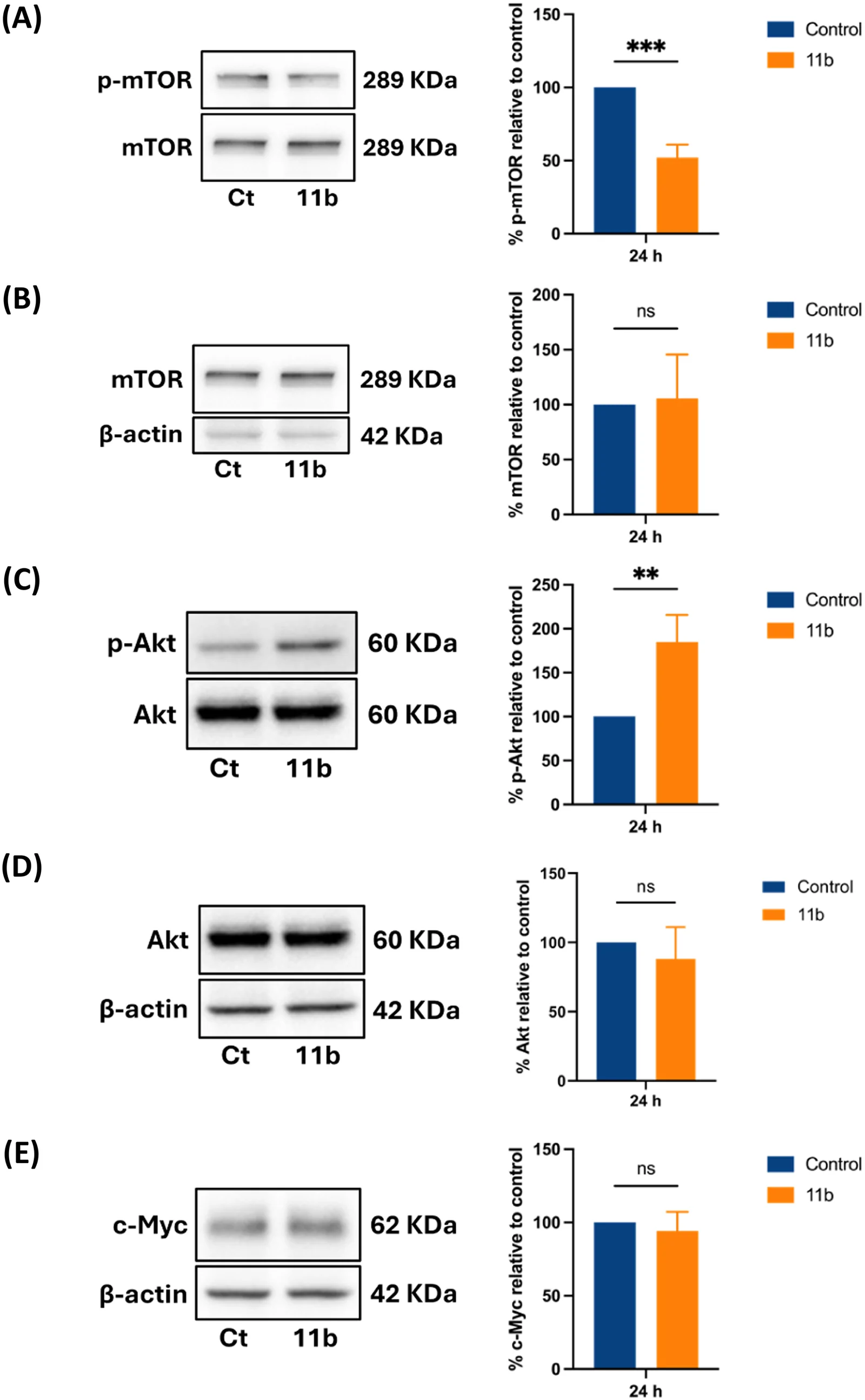
Expression levels of (A) p-mTOR (Ser2448), (B) total mTOR, (C) p-Akt (Ser473), (D) total Akt, and (E) c-Myc in SW620 cells untreated or treated with compound 11b at its IC_50_ concentration for 24 h. Protein levels were analyzed by Western blot, with β-actin used as a loading control. Band intensities were quantified and normalized to β-actin. Bar graphs represent the relative protein expression levels expressed as percentage of control. Data are expressed as mean ± SD from three independent experiments. Statistical analysis was performed using Student's *t*-test. *p* < 0.01 (**), *p* < 0.001 (***), ns, not significant.

As shown in Fig. 11, treatment with 11b led to a marked reduction in mTOR phosphorylation at Ser2448, with p-mTOR levels decreasing to approximately 52% of those observed in the control cells, whereas total mTOR expression remained unchanged. These findings indicates suppression of mTOR activation without affecting the overall abundance of the protein. In contrast, Akt phosphorylation at Ser473 was significantly increased following treatment, whereas the total Akt expression remained stable. This divergence between enhanced upstream Akt activation and reduced downstream mTOR activity suggests the involvement of compensatory signaling mechanisms, a phenomenon frequently reported upon inhibition of mTOR pathway output.^73–75^ In addition, the expression of c-Myc remained unchanged, suggesting that compound 11b does not significantly affect the transcriptional regulators associated with global proliferative signaling.

## Experimental

### Chemistry

#### General

All reagents and solvents were obtained from Sigma-Aldrich (St. Louis, MO, USA) or VWR Portugal (Carnaxide, Portugal) and, when necessary, were subjected to standard drying procedures prior to use. ORI 1 was purchased from Sigma-Aldrich (St. Louis, MO, USA). Reaction progress was monitored by thin-layer chromatography (TLC) on aluminum-backed silica gel 60 plates with fluorescent indicator F254 (Kieselgel 60 HF254, Merck Co., Rahway, NJ, USA). Preparative TLC was performed on glass plates coated with silica gel 60 (Kieselgel 60G, Merck Co., Rahway, NJ, USA). Melting points were determined on a Büchi® B-540 apparatus (Büchi®, Flawil, Switzerland) and are reported uncorrected. Infrared (IR) spectra were recorded using a PerkinElmer Spectrum 400 FT-IR/FT-NIR spectrometer (PerkinElmer, Waltham, MA, USA). Structural characterization was conducted by nuclear magnetic resonance (NMR) spectroscopy, including one-dimensional (^1^H, ^13^C, and ^19^F) and two-dimensional (COSY, NOESY, HSQC, HMBC) experiments. NMR spectra were acquired on a Bruker Avance III 400 MHz spectrometer (Bruker, Billerica, MA, USA) in CDCl_3_ or acetone-d6, operating at 400 MHz for ^1^H and 100 MHz for ^13^C. Chemical shifts (*δ*) are reported in ppm relative to residual solvent signals, and coupling constants (*J*) are expressed in Hz. Compound names were generated according to IUPAC nomenclature guidelines, using ChemDraw Professional 22.0.0.22 (PerkinElmer, Shelton, CT, USA). Mass spectrometry (MS) analyses were performed using a Thermo Scientific Finnigan LXQ Linear Ion Trap mass spectrometer (Thermo Fisher Scientific, Waltham, MA, USA) equipped with an electrospray ionization (ESI) source. Spectra were acquired in positive ion mode at 5 kV, using a sheath gas flow of 5 U and a capillary temperature of 250 °C. Elemental analysis was carried out using a TruSpec 630-200-200 CHNS analyzer (Leco Corporation, St. Joseph, MI, USA). Hydrate forms, when present, were assigned based on elemental analysis data, using calculated values for the corresponding anhydrous and hydrate formulations. The presence of water was supported by signals observed in the ^1^H NMR spectra.

Compounds 1a,^76^1c and 1d,^77^2,^78^3 and 5,^79^3a and 11a,^80^3c, 3d and 9d,^81^4, 7–9, 11 and 12,^41^10,^52^12c,^82^ and 12d^83^ have been previously reported and were synthesized as described in the literature, with minor modifications where noted. Experimental procedures and ^1^H/^13^C NMR data for these compounds are provided in the SI.

#### (1S,4aR,5S,6S,6aR,9S,11aS,11bS,14R)-1,5,6-Trihydroxy-4,4-dimethyl-8-methylene-7-oxododecahydro-1*H*-6,11b-(epoxymethano)-6a,9-methanocyclohepta[*α*]naphthalen-14-yl furan-2-carboxylate (1b)

To a solution of ORI 1 (60 mg, 0.165 mmol) in anhydrous dichloromethane (DCM, 3 mL) cooled in an ice bath, were added 2-furoic acid (2 equiv), 1-ethyl-3-(3-dimethylaminopropyl)carbodiimide (EDCI) (2 equiv) and DMAP (2 equiv). The reaction mixture was stirred in the ice bath for 15 min, after which the cooling bath was removed, and the mixture was allowed to warm to room temperature. Upon complete consumption of the starting material after 3 h, as monitored by TLC, the reaction mixture was diluted with aqueous HCl (5%, 20 mL) and extracted with DCM (3 × 30 mL). The combined organic layers were sequentially washed with water (45 mL), aqueous NaHCO_3_ (10%, 45 mL), and brine (45 mL). The organic phase was dried over anhydrous Na_2_SO_4_, filtered, and concentrated under reduced pressure to afford the crude product. Purification by preparative TLC and subsequent recrystallization yielded the desired derivative as a white solid (yield 38%). MP: 247.8–249.3 °C (ethyl acetate/petroleum ether). IR (ATR) *v*_max_: 3351, 3140, 2932, 1712, 1649, 1570, 1179, 1059 cm^−1^. ^1^H NMR (400 MHz, CDCl_3_) *δ* 7.55 (dd, *J* = 1.8, 0.8 Hz, 1H), 7.23 (dd, *J* = 3.5, 0.8 Hz, 1H), 6.51 (dd, *J* = 3.6, 1.7 Hz, 1H), 6.22–6.15 (m, 2H, 6-OH and 17′-H̲), 5.97 (d, *J* = 1.5 Hz, 1H, 14-H̲), 5.51 (d, *J* = 1.0 Hz, 1H, 17″-H̲), 4.71 (s, 1H, 7-OH̲), 4.35 (dd, *J* = 10.5, 1.6 Hz, 1H, 20′-H̲), 4.12 (dd, *J* = 10.6, 1.9 Hz, 1H, 20″-H̲), 3.83 (dd, *J* = 10.2, 6.4 Hz, 1H, 6-H̲), 3.55 (dd, *J* = 11.0, 5.9 Hz, 1H, 1-H̲), 3.43 (d, *J* = 10.0 Hz, 1H, 13-H̲), 1.15 (s, 3H, 18-CH̲_3_), 1.14 (s, 3H, 19-CH̲_3_). ^13^C NMR (100 MHz, CDCl_3_) *δ* 206.6 (C15), 156.7, 149.5 (C16), 147.0, 143.6, 120.2 (C17), 119.3, 112.3, 96.3 (C7), 78.2 (C14), 73.8 (C6), 73.5 (C1), 63.5, 62.0, 60.1, 54.5, 41.3, 41.1, 38.6, 33.8, 32.5 (C18), 30.5, 30.1, 21.5 (C19), 19.7. ESI-MS *m*/*z*: 481.3 [M + Na]^+^, anal. calcd for C_25_H_30_O_8_: C, 65.5; H, 6.60. Found: C, 65.4; H, 6.28%.

#### (4aR,5S,6S,6aR,9S,11aS,11bS,14R)-5,6-Dihydroxy-4,4-dimethyl-8-methylene-1,7-dioxododecahydro-1*H*-6,11b-(epoxymethano)-6a,9-methanocyclohepta[*α*]naphthalen-14-yl furan-2-carboxylate (3b)

Compound 3b was synthesized following the procedure used for compound 1b. The product was purified by preparative TLC followed by recrystallization to afford a white solid (yield 89%). MP: 152.5–154.4 °C (ethyl acetate/petroleum ether). IR (ATR) *v*_max_: 3375, 2936, 1707, 1642, 1577, 1471, 1294, 1051 cm^−1^. ^1^H NMR (400 MHz, CDCl_3_) *δ* 7.56 (dd, *J* = 1.8, 0.8 Hz, 1H), 7.20 (dd, *J* = 3.6, 0.9 Hz, 1H), 6.51 (dd, *J* = 3.5, 1.7 Hz, 1H), 6.29 (d, *J* = 1.4 Hz, 1H, 17′-H̲), 5.97 (d, *J* = 1.3 Hz, 1H, 14-H̲), 5.64 (d, *J* = 1.3 Hz, 1H, 17″-H̲), 5.45 (d, *J* = 11.3 Hz, 1H, 6-OH̲), 4.57 (s, 1H, 7-OH̲), 4.34 (dd, *J* = 10.7, 1.5 Hz, 1H, 20′-H̲), 4.09 (dd, *J* = 10.7, 1.8 Hz, 1H, 20″-H̲), 3.85 (dd, *J* = 11.3, 8.5 Hz, 1H, 6-H̲), 3.38 (dd, *J* = 9.1, 1.6 Hz, 1H, 13-H̲), 1.24 (s, 3H, 18-CH̲_3_), 1.05 (s, 3H, 19-CH̲_3_). ^13^C NMR (100 MHz, CDCl_3_) *δ* 211.6 (C1), 204.8 (C15), 156.7, 149.1 (C16), 147.0, 143.7, 122.2 (C17), 119.3, 112.2, 97.1 (C7), 76.6 (C14), 73.1 (C6), 65.0, 61.4, 60.6, 50.9, 48.6, 41.2, 38.7, 35.9, 32.9, 30.6 (C18), 30.1, 23.3 (C19), 19.0. ESI-MS *m*/*z*: 456.9 [M + H]^+^, anal. calcd for C_25_H_28_O_8_: C, 65.8; H, 6.18. Found: C, 65.4; H, 5.81%.

#### (4aR,5R,6aR,9S,11aS,11bS,14R)-14-Hydroxy-4,4-dimethyl-8-methyleneoctahydro-1*H*-5,11b-(epoxymethano)-6a,9-methanocyclohepta[*α*]naphthalene-1,6,7(2*H*,8*H*)-trione (6)

To a solution of compound 5 (60 mg, 0.149 mmol) in anhydrous dichloromethane (DCM, 3 mL) cooled in an ice bath, was added Deoxo-Fluor (6 equiv). The reaction mixture was stirred in the ice bath for 1 h. Upon complete consumption of the starting material, as monitored by TLC, the reaction mixture was diluted with water (20 mL) and extracted with DCM (3 × 30 mL). The combined organic layers were sequentially washed with water (45 mL), aqueous NaHCO_3_ (10%, 45 mL), and brine (45 mL). The organic phase was dried over anhydrous Na_2_SO_4_, filtered, and concentrated under reduced pressure to afford the crude product. Purification by preparative TLC and subsequent recrystallization yielded the desired derivative as a white to yellowish solid (yield 92%). MP: 102.2–104.1 °C (ethyl acetate/petroleum ether). IR (ATR) *v*_max_: 3434, 2939, 1739, 1699, 1645, 1264, 1069, 1045 cm^−1^. ^1^H NMR (400 MHz, CDCl_3_) *δ* 6.19 (d, *J* = 1.0 Hz, 1H, 17′-H̲), 5.61 (d, *J* = 1.2 Hz, 1H, 17″-H̲), 4.62 (d, *J* = 1.5 Hz, 1H, 14-H̲), 4.32 (s, 1H, 6-H̲), 4.17 (s, 2H, 20-CH̲_2_), 3.18–3.06 (m, 1H, 13-H̲), 1.23 (s, 3H, 18-CH̲_3_), 1.11 (s, 3H, 19-CH̲_3_). ^13^C NMR (100 MHz, CDCl_3_) *δ* 209.9 (C1), 206.1 (C7), 202.1 (C15), 149.3 (C16), 121.0 (C17), 82.4 (C6), 73.7 (C14), 69.5, 64.0, 59.5, 58.4, 45.2, 43.4, 40.4, 36.1, 31.5, 31.2 (C18), 29.5, 23.5 (C19), 19.9. ESI-MS *m*/*z*: 367.2 [M + Na]^+^, anal. calcd for C_20_H_24_O_5_·1.0H_2_O: C, 66.3; H, 7.23. Found: C, 66.4; H, 6.61%.

#### (1S,4aR,5S,6S,6aR,9S,11aS,11bS,14R)-1-Acetoxy-5,6-dihydroxy-4,4-dimethyl-8-methylene-7-oxododecahydro-1*H*-6,11b-(epoxymethano)-6a,9-methanocyclohepta[*α*]naphthalen-14-yl pent-4-ynoate (9a)

Compound 9a was synthesized following the procedure used for compound 1b, except that 4-pentynoic acid was employed instead. The product was purified by preparative TLC followed by recrystallization to afford a white solid (yield 69%). MP: 178.5–180.0 °C (ethyl acetate/petroleum ether). IR (ATR) *v*_max_: 3274, 2935, 1722, 1645, 1372, 1244, 1167, 1063 cm^−1^. ^1^H NMR (400 MHz, CDCl_3_) *δ* 6.18 (d, *J* = 1.2 Hz, 1H, 17′-H̲), 6.10 (d, *J* = 10.6 Hz, 1H, 6-OH̲), 5.90 (d, *J* = 1.5 Hz, 1H, 14-H̲), 5.53 (d, *J* = 1.1 Hz, 1H, 17″-H̲), 4.65 (dd, *J* = 11.2, 5.6 Hz, 1H, 1-H̲), 4.30 (dd, *J* = 10.6, 1.6 Hz, 1H, 20′-H̲), 4.21 (dd, *J* = 10.7, 1.3 Hz, 1H, 20″-H̲), 4.03 (s, 1H, 7-OH̲), 3.79 (dd, *J* = 10.6, 6.7 Hz, 1H, 6-H̲), 3.19 (d, *J* = 10.0 Hz, 1H, 13-H̲), 2.02 (s, 3H, OCOCH̲_3_), 1.98 (t, *J* = 2.5 Hz, 1H, –C≡CH̲), 1.15 (s, 6H, 18-CH̲_3_ and 19-CH̲_3_). ^13^C NMR (100 MHz, CDCl_3_) *δ* 206.1 (C15), 170.4, 169.9 (OC̲OCH_3_), 149.4 (C16), 120.6 (C17), 96.0 (C7), 81.9 (–C̲≡CH), 76.2 (C14), 75.4 (C1), 74.1 (C6), 69.5 (–C≡C̲H), 63.5, 61.6, 60.1, 53.8, 41.2, 39.8, 38.2, 33.7, 33.6, 32.4 (C18), 30.2, 25.2, 21.58 (C19), 21.53 (OCOC̲H_3_), 18.1, 14.2. ESI-MS *m*/*z*: 487.0 [M + H]^+^, anal. calcd for C_27_H_34_O_8_·0.8C_6_H_12_: C, 68.9; H, 7.93. Found: C, 69.0; H, 7.28%.

#### (1S,4aR,5S,6S,6aR,9S,11aS,11bS,14R)-1-Acetoxy-5,6-dihydroxy-4,4-dimethyl-8-methylene-7-oxododecahydro-1*H*-6,11b-(epoxymethano)-6a,9-methanocyclohepta[*α*]naphthalen-14-yl furan-2-carboxylate (9b)

Compound 9b was synthesized following the procedure used for compound 1b. The product was purified by preparative TLC followed by recrystallization to afford a white solid (yield 44%). MP: 147.3–149.0 °C (ethyl acetate/petroleum ether). IR (ATR) *v*_max_: 3545, 3365, 2936, 1718, 1644, 1472, 1296, 1048 cm^−1^. ^1^H NMR (400 MHz, CDCl_3_) *δ* 7.56 (dd, *J* = 1.8, 0.8 Hz, 1H), 7.25 (dd, *J* = 3.6, 0.9 Hz, 1H), 6.53 (dd, *J* = 3.6, 1.7 Hz, 1H), 6.23–6.16 (m, 2H, 6-OH̲ and 17′-H̲), 5.93 (d, *J* = 1.5 Hz, 1H, 14-H̲), 5.53 (d, *J* = 1.1 Hz, 1H, 17″-H̲), 4.73 (s, 1H, 7-OH̲), 4.69 (dd, *J* = 11.3, 5.6 Hz, 1H, 1-H̲), 4.35–4.19 (m, 2H, 20-CH̲_2_), 3.85 (dd, *J* = 10.1, 6.2 Hz, 1H, 6-H̲), 3.43 (d, *J* = 10.0 Hz, 1H, 13-H̲), 2.04 (s, 3H, OCOCH̲_3_), 1.16 (s, 3H, 18-CH̲_3_), 1.15 (s, 3H, 19-CH̲_3_). ^13^C NMR (100 MHz, CDCl_3_) *δ* 206.3 (C15), 170.0 (OC̲OCH_3_), 156.7, 149.0 (C16), 147.1, 143.4, 120.6 (C17), 119.5, 112.4, 96.2 (C7), 77.9 (C14), 75.4 (C1), 73.6 (C6), 63.6, 61.6, 60.5, 53.6, 40.8, 39.8, 38.1, 33.6, 32.3, 30.2 (C18), 25.2, 21.6 (C19), 21.4 (OCOC̲H_3_), 17.9. ESI-MS *m*/*z*: 523.3 [M + Na]^+^, anal. calcd for C_27_H_32_O_9_: C, 64.8; H, 6.44. Found: C, 64.5; H, 6.39%.

#### (1S,4aR,5S,6S,6aR,9S,11aS,11bS,14R)-1-Acetoxy-5,6-dihydroxy-4,4-dimethyl-8-methylene-7-oxododecahydro-1*H*-6,11b-(epoxymethano)-6a,9-methanocyclohepta[*α*]naphthalen-14-yl benzoate (9c)

Compound 9c was synthesized following the procedure used for compound 1c (see SI). The product was purified by preparative TLC followed by recrystallization to afford a white solid (yield 55%). MP: 188.3–190.0 °C (ethyl acetate/petroleum ether). IR (ATR) *v*_max_: 3347, 2936, 1713, 1643, 1599, 1274, 1241, 1061 cm^−1^. ^1^H NMR (400 MHz, CDCl_3_) *δ* 7.97–7.92 (m, 2H, 2 × Ar–H̲), 7.61–7.55 (m, 1H, Ar–H̲), 7.46–7.41 (m, 2H, 2 × Ar–H̲), 6.22 (d, *J* = 1.2 Hz, 1H, 17′-H̲), 6.16 (d, *J* = 10.6 Hz, 1H, 6-OH̲), 6.09 (d, *J* = 1.5 Hz, 1H, 14-H̲), 5.53 (d, *J* = 1.1 Hz, 1H, 17″-H̲), 4.69 (dd, *J* = 11.3, 5.6 Hz, 1H, 1-H̲), 4.36 (dd, *J* = 10.6, 1.6 Hz, 1H, 20′-H̲), 4.23 (dd, *J* = 10.5, 1.4 Hz, 1H, 20″-H̲), 3.82 (dd, *J* = 10.5, 6.6 Hz, 1H, 6-H̲), 3.33 (d, *J* = 9.9 Hz, 1H, 13-H̲), 2.05 (s, 3H, OCOCH̲_3_), 1.17 (s, 3H, 18-CH̲_3_), 1.15 (s, 3H, 19-CH̲_3_). ^13^C NMR (100 MHz, CDCl_3_) *δ* 206.4 (C15), 169.9 (OC̲OCH_3_), 165.2, 149.6 (C16), 133.6 (Ar–C̲), 129.7 (2 × Ar–C̲), 129.3 (Ar–C̲), 128.7 (2 × Ar–C̲), 120.5 (C17), 96.1 (C7), 77.2 (C14), 75.4 (C1), 74.1 (C6), 63.6, 61.8, 60.3, 53.9, 41.3, 39.8, 38.2, 33.6, 32.4 (C18), 30.3, 25.2, 21.6 (C19 and OCOC̲H_3_), 18.1. ESI-MS *m*/*z*: 533.3 [M + Na]^+^, anal. calcd for C_29_H_34_O_8_·0.7H_2_O: C, 66.6; H, 6.82. Found: C, 66.6; H, 6.09%.

#### (3S,3aR,6aS,8aR,11S,13aS,13bS,14R)-4,4-Dimethyl-10-methylene-8,9-dioxododecahydro-1*H*,8*H*-8a,11-methanocyclohepta[*c*]furo[3,4-*e*]chromene-3,14-diyl bis(furan-2-carboxylate) (11b)

Compound 11b was synthesized following the procedure used for compound 1b. The product was purified by preparative TLC followed by recrystallization to afford a white solid (yield 89%). MP: 205.7–207.3 °C (ethyl acetate/petroleum ether). IR (ATR) *v*_max_: 2944, 1757, 1717, 1646, 1577, 1471, 1292, 1171 cm^−1^. ^1^H NMR (400 MHz, CDCl_3_) *δ* 7.58 (dd, *J* = 1.8, 0.8 Hz, 1H), 7.49 (dd, *J* = 1.8, 0.8 Hz, 1H), 7.19 (dd, *J* = 3.6, 0.8 Hz, 1H), 7.12 (dd, *J* = 3.6, 0.8 Hz, 1H), 6.52 (dd, *J* = 3.5, 1.7 Hz, 1H), 6.48 (dd, *J* = 3.5, 1.7 Hz, 1H), 6.39 (s, 1H, 6-H̲), 6.29 (d, *J* = 1.2 Hz, 1H, 17′-H̲), 5.89 (d, *J* = 1.4 Hz, 1H, 14-H̲), 5.64 (d, *J* = 1.1 Hz, 1H, 17″-H̲), 4.66 (dd, *J* = 11.5, 5.8 Hz, 1H, 1-H̲), 4.22 (d, *J* = 9.7 Hz, 1H, 20′-H̲), 4.09 (d, *J* = 9.6 Hz, 1H, 20″-H̲), 3.36 (dd, *J* = 9.2, 1.5 Hz, 1H, 13-H̲), 1.13 (s, 3H, 18-CH̲_3_), 1.08 (s, 3H, 19-CH̲_3_). ^13^C NMR (100 MHz, CDCl_3_) *δ* 197.6 (C15), 165.9 (C7), 157.7, 156.9, 147.7 (C16), 147.2, 146.3, 144.5, 143.3, 120.5 (C17), 119.9, 118.5, 112.18, 112.05, 101.9 (C6), 75.8 (C1), 75.3, 74.2 (C14), 59.8, 53.6, 49.8, 48.1, 40.8, 37.0, 32.9 (C18), 31.4, 30.2, 23.2, 23.0 (C19), 19.8. ESI-MS *m*/*z*: 573.2 [M + Na]^+^, anal. calcd for C_30_H_30_O_10_: C, 65.5; H, 5.49. Found: C, 65.4; H, 5.26%.

#### (3aR,6aS,8aR,11S,13aS,13bS,14R)-4,4-Dimethyl-10-methylene-3,8,9-trioxododecahydro-1*H*,8*H*-8a,11-methanocyclohepta[*c*]furo[3,4-*e*]chromen-14-yl pent-4-ynoate (12a)

Compound 12a was synthesized following the procedure used for compound 9a. The product was purified by preparative TLC followed by recrystallization to afford a white solid (yield 87%). MP: 155.2–157.2 °C (ethyl acetate/petroleum ether). IR (ATR) *v*_max_: 3281, 2947, 1751, 1721, 1646, 1154, 1048, 1022 cm^−1^. ^1^H NMR (400 MHz, CDCl_3_) *δ* 6.29 (d, *J* = 1.3 Hz, 1H, 17′-H̲), 5.75 (d, *J* = 1.4 Hz, 1H, 14-H̲), 5.65 (d, *J* = 1.2 Hz, 1H, 17″-H̲), 4.60 (dd, *J* = 11.6, 5.7 Hz, 1H, 1-H̲), 4.36 (d, *J* = 10.1 Hz, 1H, 20′-H̲), 4.05 (d, *J* = 10.2 Hz, 1H, 20″-H̲), 3.21 (dd, *J* = 9.4, 1.4 Hz, 1H, 13-H̲), 1.96 (t, *J* = 2.6 Hz, 1H, –C≡CH̲), 1.23 (s, 3H, 18-CH̲_3_), 1.09 (s, 3H, 19-CH̲_3_). ^13^C NMR (100 MHz, CDCl_3_) *δ* 197.0 (C15), 175.3 (C6), 171.6, 166.1 (C7), 146.8 (C16), 121.7 (C17), 82.2 (–C̲≡CH), 74.5 (C1), 73.5 (C14), 71.4, 69.1 (–C≡C̲H), 59.4, 50.8, 47.4, 45.9, 40.2, 36.4, 33.12, 33.07, 32.3 (C18), 29.5, 23.6 (C19), 23.0, 19.2, 14.0. ESI-MS *m*/*z*: 441.1 [M + H]^+^, anal. calcd for C_25_H_28_O_7_: C, 68.2; H, 6.41. Found: C, 68.1; H, 6.52%.

#### (3aR,6aS,8aR,11S,13aS,13bS,14R)-4,4-Dimethyl-10-methylene-3,8,9-trioxododecahydro-1*H*,8*H*-8a,11-methanocyclohepta[*c*]furo[3,4-*e*]chromen-14-yl furan-2-carboxylate (12b)

Compound 12b was synthesized following the procedure used for compound 1b. The product was purified by preparative TLC followed by recrystallization to afford a white solid (yield 48%). MP: 281.1–282.9 °C (ethyl acetate/petroleum ether). IR (ATR) *v*_max_: 2954, 1754, 1715, 1640, 1579, 1478, 1297, 1168 cm^−1^. ^1^H NMR (400 MHz, CDCl_3_) *δ* 7.59 (dd, *J* = 1.8, 0.8 Hz, 1H), 7.19 (dd, *J* = 3.5, 0.8 Hz, 1H), 6.49 (dd, *J* = 3.6, 1.7 Hz, 1H), 6.31 (d, *J* = 1.3 Hz, 1H, 17′-H̲), 5.86 (d, *J* = 1.4 Hz, 1H, 14-H̲), 5.67 (d, *J* = 1.1 Hz, 1H, 17″-H̲), 4.64 (dd, *J* = 11.5, 5.7 Hz, 1H, 1-H̲), 4.39 (d, *J* = 10.2 Hz, 1H, 20′-H̲), 4.10 (d, *J* = 10.2 Hz, 1H, 20″-H̲), 3.35 (dd, *J* = 9.5, 1.5 Hz, 1H, 13-H̲), 1.24 (s, 3H, 18-CH̲_3_), 1.09 (s, 3H, 19-CH̲_3_). ^13^C NMR (100 MHz, CDCl_3_) *δ* 197.1 (C15), 175.3 (C6), 165.4 (C7), 157.7, 147.3, 146.8 (C16), 143.2, 121.7 (C17), 120.0, 112.1, 74.5 (C1), 73.8 (C14), 71.4, 59.4, 50.8, 47.7, 46.1, 40.5, 36.4, 33.1, 32.3 (C18), 29.5, 23.6 (C19), 23.1, 19.2. ESI-MS *m*/*z*: 477.2 [M + Na]^+^, anal. calcd for C_25_H_26_O_8_·0.2C_6_H_12_: C, 66.7; H, 6.07. Found: C, 66.5; H, 5.64%.

### Biology

#### Materials

All reagents were of analytical or cell culture grade. Gibco (Thermo Fisher Scientific, Waltham, MA, USA) supplied Dulbecco's modified Eagle medium (DMEM; Cat. No. 11966-025), Penicillin/Streptomycin solution (Cat. No. 15140-122), Fetal Bovine Serum (Cat. No. A5256701), 0.05% trypsin–EDTA (Cat. No. 25300-062), and 0.4% Trypan Blue solution (Cat. No. 15250-061). Thermo Fisher Scientific (Waltham, MA, USA) also provided DMSO (Cat. No. BP231-100), Pierce™ BCA Protein Assay reagents A (Cat. No. 23228) and B (Cat. No. 1859078), BSA standard ampules (Cat. No. 23209), and Spectra™ Multicolor Broad Range Protein Ladder (Cat. No. 26634). Additional DMSO (≥99.9%; Cat. No. D/4121/PB15) was obtained from Fisher Scientific. BioWest (Nuaillé, France) supplied Ham's F-12 medium without l-glutamine (Cat. No. L0136). Linus, represented by Cultek (Madrid, Spain) provided Dulbecco's phosphate-buffered saline (DPBS) without calcium and magnesium (Cat. No. L0615). Sigma-Aldrich (St. Louis, MO, USA) supplied d-(+)-glucose solution (Cat. No. G8769-100ML), Triton™ X-100 (Cat. No. T9284), Trizma® base (Cat. No. T1503), sodium dodecyl sulfate (SDS; Cat. No. L3771), sodium deoxycholate (Cat. No. D6750), Tween® 20 (Cat. No. P9416), H_2_DCFDA (Cat. No. D6883), propidium iodide (Cat. No. P4864), and TEMED (Cat. No. T9281). PanReac AppliChem (Barcelona, Spain) supplied thiazolyl blue tetrazolium bromide (MTT; Cat. No. A2231) and 30% acrylamide solution (29:1; Cat. No. A4983). Elabscience Biotechnology Inc. (Houston, TX, USA) provided Annexin V-FITC (Cat. No. E-CK-A111). Bio-Rad Laboratories (Hercules, CA, USA) supplied ammonium persulfate (APS; Cat. No. 1610700). Roche Diagnostics (Rotkreuz, Switzerland) provided RNase A (Cat. No. 10109142001). Merck Millipore (Darmstadt, Germany) supplied protease inhibitor cocktail III (Cat. No. 535140), phosphatase inhibitor cocktail IV (Cat. No. 524628), PVDF membranes (Immobilon®-P; Cat. No. IPVH00010), and the Immobilon® Western Chemiluminescent HRP Substrate Kit (Cat. No. WBKLS0050). Skimmed milk powder was commercially sourced (Nestlé, Vevey, Switzerland). Primary antibodies against CDK1 (sc-54), CDK4 (sc-260) and CDK6 (sc-177) were obtained from Santa Cruz Biotechnology (Dallas, TX, USA). Antibodies against total mTOR (7C10), phospho-mTOR (Ser2448; D9C2), total Akt (Cat. No. 9272), and phospho-Akt (Ser473; D9E) were purchased from Cell Signaling Technology (Danvers, MA, USA). The anti-actin antibody (Cat. No. 691001) was obtained from MP Biomedicals (Santa Ana, CA, USA). The anti-c-Myc antibody (Y69), HRP-conjugated anti-rabbit (Cat. No. ab6721) and anti-mouse (Cat. No. ab6728) secondary antibodies were supplied by Abcam (Cambridge, UK).

#### Compound

All compounds were stored as dry powders at −20 °C. Stock solutions were prepared in dimethyl sulfoxide (DMSO) and diluted with the appropriate culture medium to obtain the desired final concentrations. In all treatment conditions, the percentage of DMSO in the culture medium was kept at or below 0.5% (v/v).

#### Cell culture

SW620 (ATCC CCL-227), HT29 (ATCC HTB-38), and HCT116 (ATCC CCL-247) cell lines were obtained from the American Type Culture Collection (ATCC, Manassas, VA, USA). NCM460 cells (RRID:CVCL_0460) were acquired from INCELL Corporation (San Antonio, TX, USA). SW620 and NCM460 cells were grown in glucose-free DMEM supplemented with 12.5 mM d-glucose, 5% fetal bovine serum (FBS), and 1% penicillin–streptomycin (P/S) solution. HT29 cells were maintained in the same basal medium containing 12.5 mM d-glucose and 1% P/S, but were supplemented with 10% FBS. HCT116 cells were cultured in a 1 : 1 mixture of glucose-free DMEM and Ham's F-12 medium supplemented with 11.25 mM d-glucose, 10% FBS, and 1% P/S. All cultures were maintained at 37 °C in a humidified incubator with 5% CO_2_. Experiments were performed using subconfluent monolayers.

#### Cell viability assay

Cell viability was determined using the MTT assay. Cells were seeded in 96-well plates at the following densities: SW620 (3000 cells per well), HCT116 (1900 cells per well), HT29 (3500 cells per well), and NCM460 (6000 cells per well). After 24 h to allow attachment, the medium was replaced with fresh culture medium containing increasing concentrations of the test compounds or vehicle (DMSO). After 72 h of treatment, the culture medium was aspirated, and 100 μL of a freshly prepared 1 : 1 solution of MTT (1 mg mL^−1^ in PBS, filtered) and serum-free medium was added to each well. Plates were incubated for 1 h at 37 °C. The MTT-containing solution was then removed, and the resulting formazan crystals were solubilized with 100 μL DMSO per well. Absorbance was measured at 570 nm using a Multiskan SkyHigh microplate reader (Thermo Fisher Scientific, Waltham, MA, USA). IC_50_ values were determined using GraphPad Prism 10 (GraphPad Software, San Diego, CA, USA) and are presented as the mean ± standard deviation (SD) from at least three independent experiments.

#### Apoptosis assay

SW620 cells were seeded in 6-well plates at a density of 1.25 × 10^5^ cells per well and allowed to attach for 24 h at 37 °C. Cells were then treated with either fresh culture medium (control) or compound 11b at its previously determined IC_50_ concentration for 24, 48, or 72 h. At each time point, both floating and attached cells were collected. Adherent cells were gently detached by trypsinization, washed with DPBS, and combined with floating cells collected by centrifugation. The resulting cell pellets were resuspended in 95 μL of binding buffer (10 mM HEPES/NaOH, pH 7.4, 140 mM NaCl, 2.5 mM CaCl_2_) and stained with 3 μL of Annexin V-FITC for 30 min at room temperature in the absence of light. After incubation, 500 μL of binding buffer and 10 μL of propidium iodide (PI; 1 mg mL^−1^) were added immediately before analysis. Samples were analyzed using a Gallios flow cytometer (Beckman Coulter, Brea, CA, USA) with 488 nm excitation, and at least 1 × 10^4^ events were acquired per sample.

#### Cell cycle assay

SW620 cells were seeded in 6-well plates and treated as described for the apoptosis assay. After centrifugation, cells were resuspended in 500 μL of cold DPBS. While gently vortexing, the suspension was slowly added dropwise to 4.5 mL of cold 70% ethanol (v/v) for fixation. Samples were kept at −20 °C for a minimum of 4 h. Fixed cells were pelleted, washed with cold DPBS to remove residual ethanol, and resuspended in 350 μL of DPBS containing RNase A (10 μL of a 10 mg mL^−1^ stock solution). Following incubation for 1 h at 37 °C under gentle agitation, 10 μL of propidium iodide (PI; 1 mg mL^−1^) was added immediately before acquisition. Flow cytometry analysis was performed using a Gallios flow cytometer (Beckman Coulter, Brea, CA, USA) with 488 nm excitation. At least 1 × 10^4^ events were recorded per sample. Data processing and cell cycle modeling were performed using the FlowJo software (FlowJo LLC, BD Biosciences, Ashland, OR, USA).

#### Doubling time and cell diameter

SW620 cells were seeded in 6-well plates at a density of 1.25 × 10^5^ cells per well and allowed to attach for 24 h at 37 °C. Cells were then treated with either fresh culture medium (control) or compound 11b at its previously determined IC_50_ concentration for 24, 48, or 72 h. At the start of the experiment (0 h), cells were trypsinized, washed with DPBS, and collected by centrifugation. The resulting cell pellets were resuspended in DPBS containing trypan blue, and cell number, viability, and cell diameter were determined using an Invitrogen Countess II FL Automated Cell Counter (Thermo Fisher Scientific, Waltham, MA, USA). The same procedure was performed at each treatment time point (24, 48, and 72 h) to establish cell growth curves and monitor proliferation over time.

#### Recovery assay

This assay was performed following the same protocol used for the doubling time analysis, with slight modifications. SW620 cells were seeded in 6-well plates and exposed to compound 11b at its IC_50_ concentration for 24 h. After this treatment period, the medium containing the compound was removed, cells were washed with DPBS, and fresh compound-free medium was added to allow cell recovery. Cells were then maintained for an additional 24 and 48 h. At each time point (0, 24, 48, and 72 h), cells were collected, stained with trypan blue, and counted using an Invitrogen Countess II FL Automated Cell Counter (Thermo Fisher Scientific, Waltham, MA, USA) to assess viable cell number. Cell growth curves were determined as previously described.

#### ROS determination assay

SW620 cells were seeded in black, clear-bottom 96-well plates at a density of 3000 cells per well and treated with compound 11b at its previously determined IC_50_ concentration for 24, 48, and 72 h. After treatment, cells were washed once with warm DPBS and incubated with 20 μM 2′,7′-dichlorodihydrofluorescein diacetate (H_2_DCFDA) prepared in DPBS containing 5.5 mM d-glucose for 45 min at 37 °C in a humidified atmosphere with 5% CO_2_. After this incubation, the probe solution was replaced with DPBS supplemented with 5.5 mM d-glucose, and cells were further incubated for 1 h. Fluorescence was then recorded using a FLUOstar OPTIMA microplate reader (BMG LABTECH GmbH, Ortenberg, Germany) at excitation/emission wavelengths of 485/535 nm. Measured ROS levels were normalized to cell number to correct for variations in cell density and viability.

#### Total protein extraction from cultured cells

SW620 cells were seeded in 6-well plates at a density of 1.25 × 10^5^ cells per well and allowed to attach for 24 h at 37 °C. Cells were then treated with either fresh culture medium (control) or compound 11b at its previously determined IC_50_ concentration for 24 h. after treatment, cells were washed with ice-cold DPBS and lysed for 20 min at 4 °C in RIPA buffer containing 50 mM Tris–HCl (pH 8.0), 150 mM NaCl, 0.1% (w/v) SDS, 1% Triton™ X-100, and 12 mM sodium deoxycholate, supplemented with 1% protease inhibitor cocktail and 0.5% phosphatase inhibitor cocktail. Cells were scraped, sonicated, and centrifuged at 12 000 × *g* for 20 min at 4 °C. Supernatants were collected, and protein concentrations were quantified using the Pierce™ BCA protein assay kit, with bovine serum albumin as the standard.

#### Western blot analysis

Equal amounts of protein (14 μg) were separated on 12% SDS-polyacrylamide gel electrophoresis (SDS-PAGE) and subsequently transferred onto PVDF membranes. Membranes were blocked for 1 h at room temperature in PBS containing 0.1% Tween-20 (PBS-T) supplemented with 5% (w/v) skim milk. After blocking, membranes were incubated overnight at 4 °C with the appropriate primary antibodies against CDK1, CDK4, CDK6, mTOR, phospho-mTOR (Ser2448), Akt, phospho-Akt (Ser473) or c-Myc. For β-actin detection, membranes were incubated with the primary antibody for 30 min at room temperature. Following primary antibody incubation, membranes were washed with PBS-T and then incubated for 1 h at room temperature with the corresponding HRP-conjugated secondary antibodies (anti-mouse or anti-rabbit). After extensive washing with PBS-T, immunoreactive bands were detected using the Immobilon™ Western Chemiluminescent HRP Substrate and visualized with an iBright™ Imaging System (Invitrogen™, Thermo Fisher Scientific, Waltham, MA, USA).

#### Data analysis and statistical methods

All experimental conditions were performed in technical triplicates, and each experiment was independently repeated at least three times. Statistical analyses were conducted using GraphPad Prism 10 (GraphPad Software, San Diego, CA, USA). Comparisons between two groups were performed using unpaired Student's *t*-tests, while analyses involving multiple groups were carried out using two-way ANOVA followed by Sidak's *post hoc* test or multiple unpaired *t*-tests (one per row), as appropriate. Data are presented as mean ± SD, and statistical significance was defined as *p* < 0.05 (*), *p* < 0.01 (**), and *p* < 0.001 (***).

## Conclusions

In conclusion, we successfully designed and synthesized a series of ORI 1 derivatives and evaluated their antiproliferative activities in colorectal cancer (CRC) cell lines. The main structural modification involved the functionalization of the C14 hydroxyl group, and most derivatives exhibited enhanced antiproliferative activity compared to the parent compound. Structure–activity relationship (SAR) analysis revealed that aromatic and heteroaromatic substitutions positively contributed to the antiproliferative potency. Among the synthesized compounds, difuroate enmein-type compound 11b emerged as the most interesting, displaying a favorable balance between anticancer activity and cytotoxicity toward normal colon cells. Treatment with this compound resulted in a marked reduction in SW620 cell proliferation, accompanied by an increased doubling time and significant accumulation in the S and G2/M phases of the cell cycle. Mechanistic studies further revealed that 11b reduced intracellular ROS levels, indicating modulation of redox homeostasis. In parallel, Western blot analysis showed attenuation of mTOR signaling and reduced expression of CDK1 and CDK6, consistent with the cell cycle arrest observed by flow cytometry. Collectively, these findings identify compound 11b as a potential candidate for the development of novel anticancer agents targeting CRC. Further studies are warranted to elucidate the precise molecular mechanisms underlying its anticancer activity and to evaluate its therapeutic potential in more advanced preclinical models.

## Author contributions

Pedro J. M. Sobral: conceptualization, methodology, validation, formal analysis, investigation, data curation, writing – original draft preparation, visualization. Rita I. Oliveira: methodology, formal analysis, investigation, data curation, writing – original draft preparation, visualization. Marta Cascante: conceptualization, methodology, funding acquisition, resources, supervision, validation, writing – review and editing. Silvia Marin: conceptualization, methodology, funding acquisition, resources, supervision, validation, writing – review and editing. Jorge A. R. Salvador: conceptualization, methodology, funding acquisition, resources, supervision, validation, writing – review and editing, project administration.

## Conflicts of interest

There are no conflicts to declare.

## Supplementary Material

MD-017-D6MD00432F-s001

## Data Availability

Raw data files supporting the Western blot experiments are available at Zenodo at https://doi.org/10.5281/zenodo.20416310. Supplementary information (SI): selected spectroscopic data, *in silico* ADME prediction reports, and dose-response curves. See DOI: https://doi.org/10.1039/d6md00432f.
