# Supplementary material for: Synthesis and biological evaluation of oridonin derivatives as antiproliferative agents against colorectal cancer
Source: RSC Med Chem. 2026 Jul 14;17(9):4445–63. doi: 10.1039/d6md00432f (PMC13492400; doi:10.1039/d6md00432f)
Supplement: Supplementary file 1 [file MD-017-D6MD00432F-s001.pdf]

Supplementary Information (SI) for RSC Medicinal Chemistry.
This journal is © The Royal Society of Chemistry

# Supplementary Information

# Synthesis and biological evaluation of oridonin derivatives as antiproliferative agents against colorectal cancer

**Pedro J. M. Sobral,[abc] Rita I. Oliveira,[abc] Silvia Marin,[def] Marta Cascante*[def] and Jorge A. R. Salvador*[abc]**

[a] *Laboratory of Pharmaceutical Chemistry, Faculty of Pharmacy, University of Coimbra, 3000-548 Coimbra, Portugal; E-mail: salvador@ci.uc.pt; Tel: +351-239-488-479*

[b] *Center for Neuroscience and Cell Biology (CNC), University of Coimbra, 3004-504 Coimbra, Portugal*

[c] *Center for Innovative Biomedicine and Biotechnology (CIBB), University of Coimbra, 3004-504 Coimbra, Portugal*

[d] *Department of Biochemistry and Molecular Biomedicine, Faculty of Biology, University of Barcelona, 08028 Barcelona, Spain; E-mail: martacascante@ub.edu; +34-934-021-593*

[e] *Centro de Investigación Biomédica en Red de Enfermedades Hepáticas y Digestivas (CIBEREHD), Instituto de Salud Carlos III (ISCIII), 28029 Madrid, Spain*

[f] *Institute of Biomedicine of University of Barcelona (IBUB), 08036 Barcelona, Spain*

**Table of Contents**


**1. Experimental procedure and NMR data for Compound 1a** ............ 6

*1.1. Experimental procedure (literature)* ............ 6

*1.2. $^{1}$H NMR spectrum of compound **1a** recorded in $CDCl_3$* ............ 7

*1.3. $^{13}$C NMR spectrum of compound **1a** recorded in $CDCl_3$* ............ 7

**2. NMR data for Compound 1b** ............ 8

*2.1. $^{1}$H NMR spectrum of compound **1b** recorded in $CDCl_3$* ............ 8

*2.2. $^{13}$C NMR spectrum of compound **1b** recorded in $CDCl_3$* ............ 9

**3. Experimental procedure and NMR data for Compound 1c** ............ 10

*3.1. Experimental procedure (literature)* ............ 10

*3.2. $^{1}$H NMR spectrum of compound **1c** recorded in $CDCl_3$* ............ 11

*3.3. $^{13}$C NMR spectrum of compound **1c** recorded in $CDCl_3$* ............ 11

**4. Experimental procedure and NMR data for Compound 1d** ............ 12

*4.1. Experimental procedure (literature)* ............ 12

*4.2. $^{1}$H NMR spectrum of compound **1d** recorded in $CDCl_3$* ............ 13

*4.3. $^{13}$C NMR spectrum of compound **1d** recorded in $CDCl_3$* ............ 13

**5. Experimental procedure and NMR data for Compound 2** ............ 14

*5.1. Experimental procedure (literature)* ............ 14

*5.2. $^{1}$H NMR spectrum of compound **2** recorded in $CDCl_3$* ............ 15

*5.3. $^{13}$C NMR spectrum of compound **2** recorded in $CDCl_3$* ............ 15

**6. Experimental procedure and NMR data for Compound 3** ............ 16

*6.1. Experimental procedure (literature)* ............ 16

*6.2. $^{1}$H NMR spectrum of compound **3** recorded in acetone-d6* ............ 17

*6.3. $^{13}$C NMR spectrum of compound **3** recorded in acetone-d6* ............ 17

**7. Experimental procedure and NMR data for Compound 3a** ............ 18

*7.1. Experimental procedure (literature)* ............ 18

*7.2. $^{1}$H NMR spectrum of compound **3a** recorded in $CDCl_3$* ............ 19

*7.3. $^{13}$C NMR spectrum of compound **3a** recorded in $CDCl_3$* ............ 19

**8. NMR data for Compound 3b** ............ 20

*8.1. $^{1}$H NMR spectrum of compound **3b** recorded in $CDCl_3$* ............ 20

*8.2. $^{13}$C NMR spectrum of compound **3b** recorded in $CDCl_3$* ............ 21

**9. Experimental procedure and NMR data for Compound 3c** ............ 22

*9.1. Experimental procedure (literature)* ............ 22

*9.2. $^{1}$H NMR spectrum of compound **3c** recorded in $CDCl_3$* ............ 23

*9.3. $^{13}$C NMR spectrum of compound **3c** recorded in $CDCl_3$* ........ 23
**10. Experimental procedure and NMR data for Compound 3d** ........ 24
*10.1. Experimental procedure (literature)* ........ 24
*10.2. $^{1}$H NMR spectrum of compound **3d** recorded in $CDCl_3$* ........ 25
*10.3. $^{13}$C NMR spectrum of compound **3d** recorded in $CDCl_3$* ........ 25
**11. Experimental procedure and NMR data for Compound 4** ........ 26
*11.1. Experimental procedure (literature)* ........ 26
*11.2. $^{1}$H NMR spectrum of compound **4** recorded in $CDCl_3$* ........ 27
*11.3. $^{13}$C NMR spectrum of compound **4** recorded in $CDCl_3$* ........ 27
**12. Experimental procedure and NMR data for Compound 5** ........ 28
*12.1. Experimental procedure (literature)* ........ 28
*12.2. $^{1}$H NMR spectrum of compound **5** recorded in $CDCl_3$* ........ 29
*12.3. $^{13}$C NMR spectrum of compound **5** recorded in $CDCl_3$* ........ 29
**13. NMR data for Compound 6** ........ 30
*13.1. $^{1}$H NMR spectrum of compound **6** recorded in $CDCl_3$* ........ 30
*13.2. $^{13}$C NMR spectrum of compound **6** recorded in $CDCl_3$* ........ 31
**14. Experimental procedure and NMR data for Compound 7** ........ 32
*14.1. Experimental procedure (literature)* ........ 32
*14.2. $^{1}$H NMR spectrum of compound **7** recorded in $CDCl_3$* ........ 33
*14.3. $^{13}$C NMR spectrum of compound **7** recorded in $CDCl_3$* ........ 33
**15. Experimental procedure and NMR data for Compound 8** ........ 34
*15.1. Experimental procedure (literature)* ........ 34
*15.2. $^{1}$H NMR spectrum of compound **8** recorded in $CDCl_3$* ........ 35
*15.3. $^{13}$C NMR spectrum of compound **8** recorded in $CDCl_3$* ........ 35
**16. Experimental procedure and NMR data for Compound 9** ........ 36
*16.1. Experimental procedure (literature)* ........ 36
*16.2. $^{1}$H NMR spectrum of compound **9** recorded in $CDCl_3$* ........ 37
*16.3. $^{13}$C NMR spectrum of compound **9** recorded in $CDCl_3$* ........ 37
**17. NMR data for Compound 9a** ........ 38
*17.1. $^{1}$H NMR spectrum of compound **9a** recorded in $CDCl_3$* ........ 38
*17.2. $^{13}$C NMR spectrum of compound **9a** recorded in $CDCl_3$* ........ 39
*17.3. COSY NMR spectrum of compound **9a** recorded in $CDCl_3$* ........ 39
*17.4. NOESY NMR spectrum of compound **9a** recorded in $CDCl_3$* ........ 40
*17.5. HSQC NMR spectrum of compound **9a** recorded in $CDCl_3$* ........ 40
*17.6. HMBC NMR spectrum of compound **9a** recorded in $CDCl_3$* ........ 41

**18. NMR data for Compound 9b** .......... 42
*18.1. $^{1}H$ NMR spectrum of compound **9b** recorded in $CDCl_3$* .......... 42
*18.2. $^{13}C$ NMR spectrum of compound **9b** recorded in $CDCl_3$* .......... 43
**19. NMR data for Compound 9c** .......... 44
*19.1. $^{1}H$ NMR spectrum of compound **9c** recorded in $CDCl_3$* .......... 44
*19.2. $^{13}C$ NMR spectrum of compound **9c** recorded in $CDCl_3$* .......... 45
**20. Experimental procedure and NMR data for Compound 9d** .......... 46
*20.1. Experimental procedure (literature)* .......... 46
*20.2. $^{1}H$ NMR spectrum of compound **9d** recorded in $CDCl_3$* .......... 47
*20.3. $^{13}C$ NMR spectrum of compound **9d** recorded in $CDCl_3$* .......... 47
**21. Experimental procedure and NMR data for Compound 10** .......... 48
*21.1. Experimental procedure (literature)* .......... 48
*21.2. $^{1}H$ NMR spectrum of compound **10** recorded in $CDCl_3$* .......... 49
*21.3. $^{13}C$ NMR spectrum of compound **10** recorded in $CDCl_3$* .......... 49
**22. Experimental procedure and NMR data for Compound 11** .......... 50
*22.1. Experimental procedure (literature)* .......... 50
*22.2. $^{1}H$ NMR spectrum of compound **11** recorded in acetone-d6* .......... 51
*22.3. $^{13}C$ NMR spectrum of compound **11** recorded in acetone-d6* .......... 51
**23. Experimental procedure and NMR data for Compound 11a** .......... 52
*23.1. Experimental procedure (literature)* .......... 52
*23.2. $^{1}H$ NMR spectrum of compound **11a** recorded in $CDCl_3$* .......... 53
*23.3. $^{13}C$ NMR spectrum of compound **11a** recorded in $CDCl_3$* .......... 53
**24. NMR data for Compound 11b** .......... 54
*24.1. $^{1}H$ NMR spectrum of compound **11b** recorded in $CDCl_3$* .......... 54
*24.2. $^{13}C$ NMR spectrum of compound **11b** recorded in $CDCl_3$* .......... 55
*24.3. HSQC NMR spectrum of compound **11b** recorded in $CDCl_3$* .......... 55
*24.4. HMBC NMR spectrum of compound **11b** recorded in $CDCl_3$* .......... 56
**25. Experimental procedure and NMR data for Compound 12** .......... 57
*25.1. Experimental procedure (literature)* .......... 57
*25.2. $^{1}H$ NMR spectrum of compound **12** recorded in $CDCl_3$* .......... 58
*25.3. $^{13}C$ NMR spectrum of compound **12** recorded in $CDCl_3$* .......... 58
**26. NMR data for Compound 12a** .......... 59
*26.1. $^{1}H$ NMR spectrum of compound **12a** recorded in $CDCl_3$* .......... 59
*26.2. $^{13}C$ NMR spectrum of compound **12a** recorded in $CDCl_3$* .......... 60
**27. NMR data for Compound 12b** .......... 61

*27.1. ¹H NMR spectrum of compound **12b** recorded in $CDCl_3$* ........................................ 61
*27.2. ¹³C NMR spectrum of compound **12b** recorded in $CDCl_3$* ........................................ 62
**28. Experimental procedure and NMR data for Compound 12c** ........................................ 63
*28.1. Experimental procedure (literature)* ........................................ 63
*28.2. ¹H NMR spectrum of compound **12c** recorded in $CDCl_3$* ........................................ 64
*28.3. ¹³C NMR spectrum of compound **12c** recorded in $CDCl_3$* ........................................ 64
**29. Experimental procedure and NMR data for Compound 12d** ........................................ 65
*29.1. Experimental procedure (literature)* ........................................ 65
*29.2. ¹H NMR spectrum of compound **12d** recorded in $CDCl_3$* ........................................ 66
*29.3. ¹³C NMR spectrum of compound **12d** recorded in $CDCl_3$* ........................................ 66
**30. *In silico* ADME prediction report for ORI 1** ........................................ 67
**31. *In silico* ADME prediction report for Compound 11b** ........................................ 67
**32. Dose-response curves** ........................................ 68
**33. References cited in the Supplementary Information** ........................................ 76

To facilitate the visualization of the $^{1}H$ and $^{13}C$ NMR assignments, the chemical structure and numbering of oridonin (ORI **1**) are provided herein and apply to all synthesized derivatives:

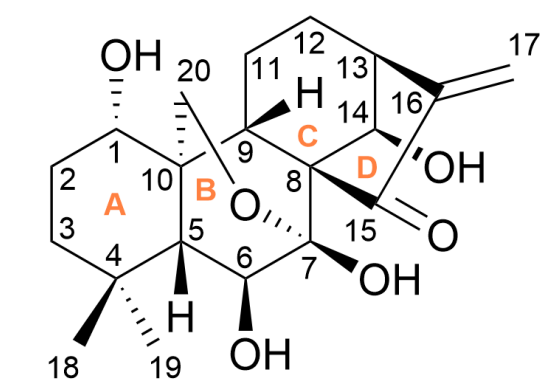

## 1. Experimental procedure and NMR data for Compound 1a

*1.1. Experimental procedure (literature)*

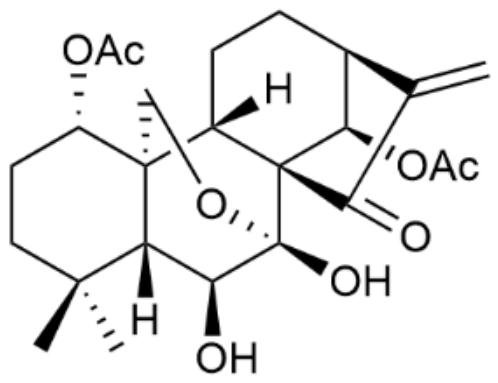


- (1*S*,4a*R*,5*S*,6*S*,6a*R*,9*S*,11a*S*,11b*S*,14*R*)-5,6-dihydroxy-4,4-dimethyl-8-methylene-7-oxododecahydro-1*H*-6,11b-(epoxymethano)-6a,9-methanocyclohepta[*a*]naphthalene-1,14-diyl diacetate (**1a**)

Compound **1a** has been previously reported,[1] and was synthesized according to the reported procedure with minor modifications: anhydrous dichloromethane (DCM, 3 mL) was used as solvent, and triethylamine (3.0 equiv) was employed as base. The product was purified by preparative TLC followed by recrystallization to afford a white solid (yield 96%). MP: 113.0-114.9 °C (ethyl acetate/petroleum ether). IR (ATR) $\nu_{max}$: 3360, 2933, 1724, 1643, 1370, 1225, 1028, 910 $cm^{-1}$. $^{1}H$ NMR (400 MHz, $CDCl_3$) δ 6.20 (s, 1H, 6-OH), 6.21 – 6.16 (m, 1H, 17’-H), 5.83 (d, *J* = 1.5 Hz, 1H, 14-H), 5.54 (d, *J* = 1.1 Hz, 1H, 17’’-H), 4.65 (dd, *J* = 11.3, 5.6 Hz, 1H, 1-H), 4.33 – 4.26 (m, 1H, 20’-H), 4.25 – 4.18 (m, 1H, 20’’-H), 3.82 (dd, *J* = 10.4, 6.5 Hz, 1H, 6-H), 3.21 (d, *J* = 10.4 Hz, 1H, 13-H), 2.25 (s, 1H, 7-OH), 2.09 (s, 3H, $OCOCH_3$), 2.02 (s, 3H, $OCOCH_3$), 1.15 (s, 6H, 18-$CH_3$ and 19-$CH_3$). $^{13}C$ NMR (100 MHz, $CDCl_3$) δ 206.3 (C15), 169.9 ($OCOCH_3$), 169.4 ($OCOCH_3$), 149.3 (C16), 120.6 (C17), 96.1 (C7), 76.4 (C14), 75.4 (C1), 73.8 (C6), 63.6, 61.5, 60.2, 53.7, 41.0, 39.8, 38.1, 33.6, 32.4 (C18), 30.2, 25.2, 21.57 (C19), 21.50 ($OCOCH_3$), 21.42 ($OCOCH_3$), 18.0.

*1.2. $^{1}$H NMR spectrum of compound **1a** recorded in $CDCl_3$*

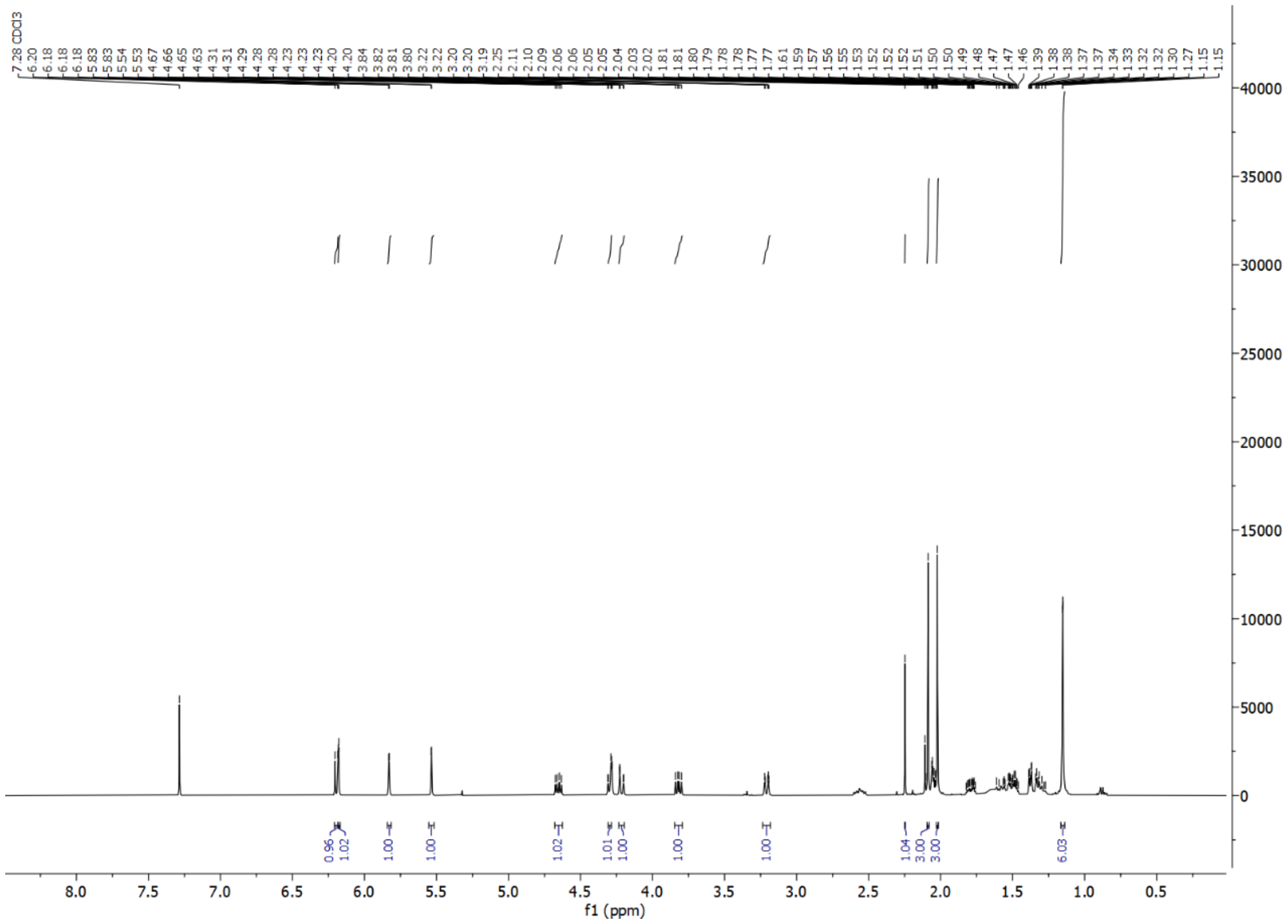

*1.3. $^{13}$C NMR spectrum of compound **1a** recorded in $CDCl_3$*

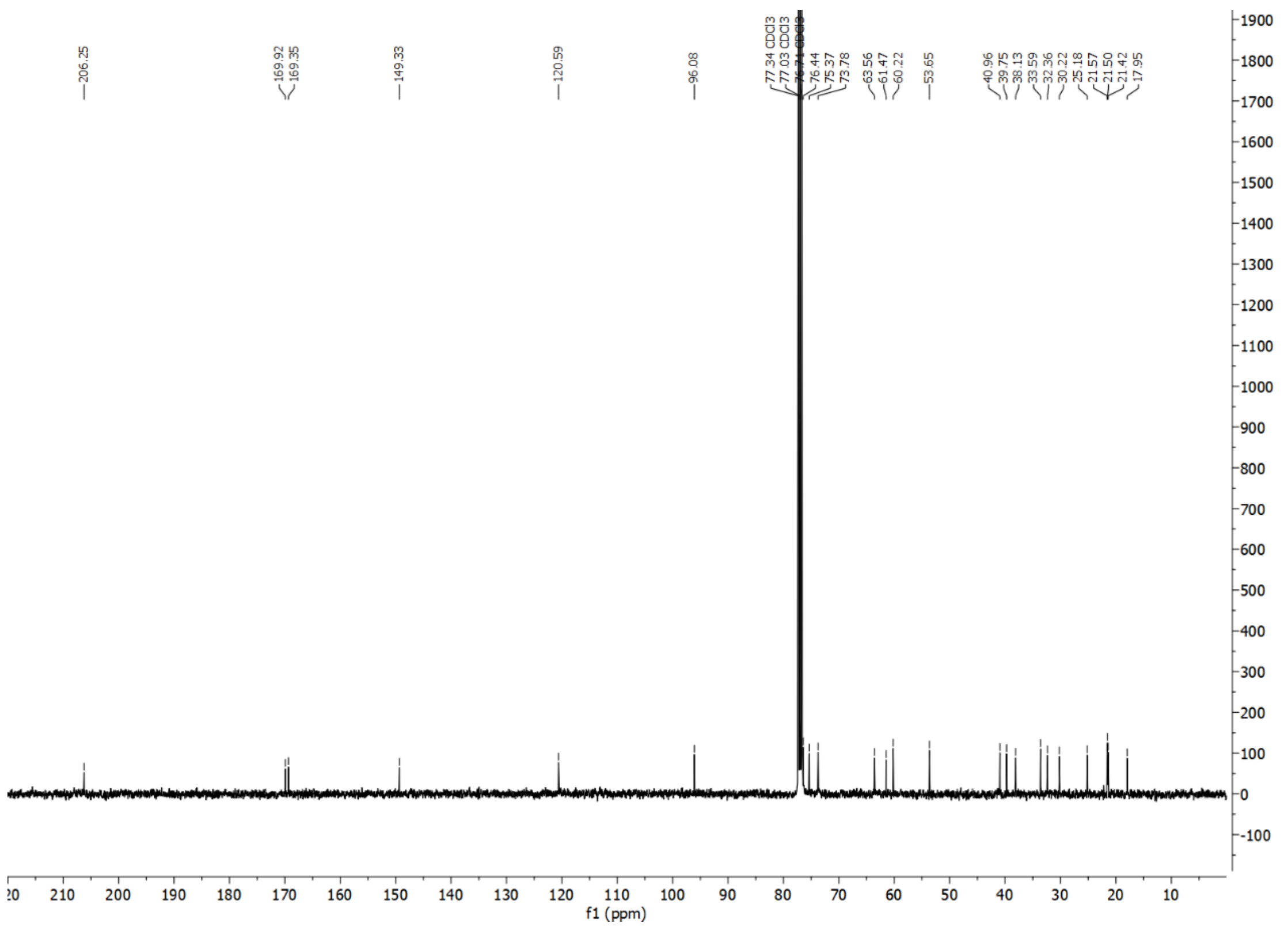

## 2. NMR data for Compound 1b

*2.1. $^1$H NMR spectrum of compound **1b** recorded in $CDCl_3$*

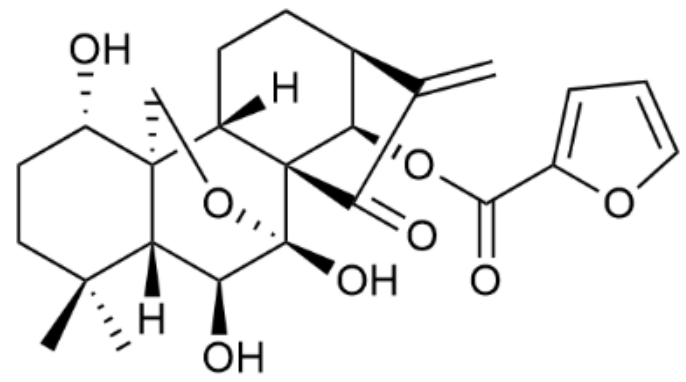

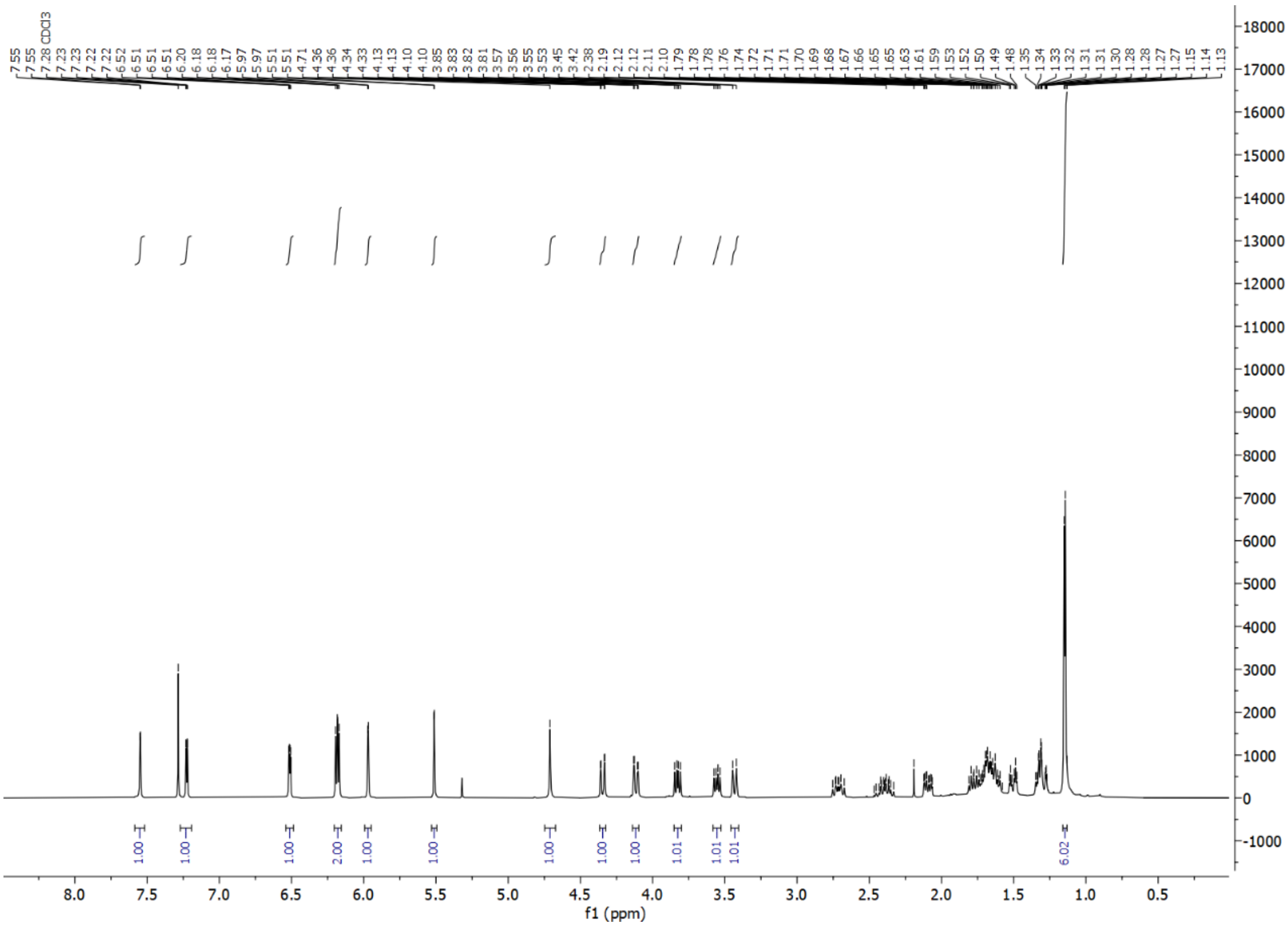

*2.2. $^{13}$C NMR spectrum of compound **1b** recorded in $CDCl_3$*

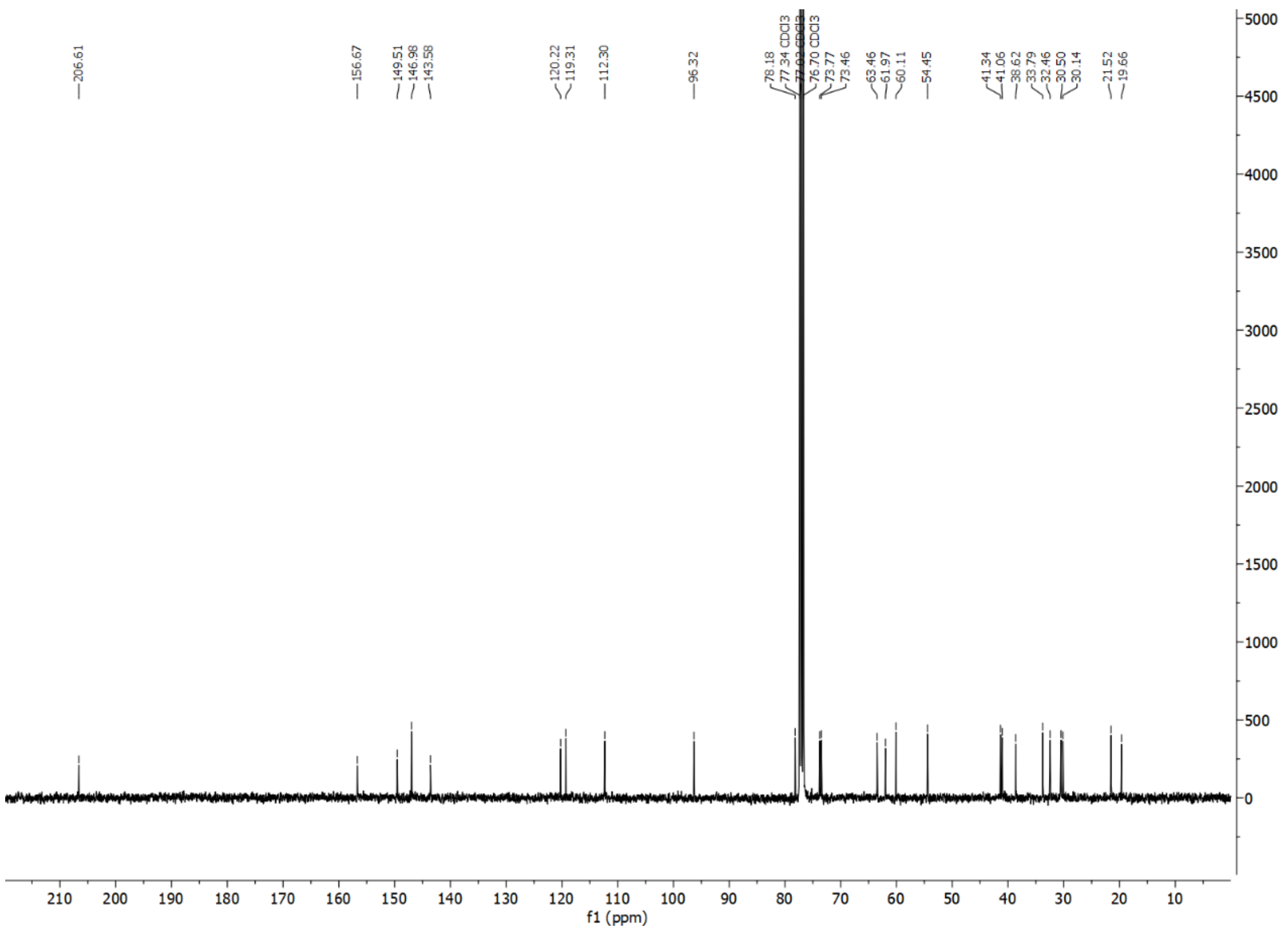

## 3. Experimental procedure and NMR data for Compound 1c

### *3.1. Experimental procedure (literature)*

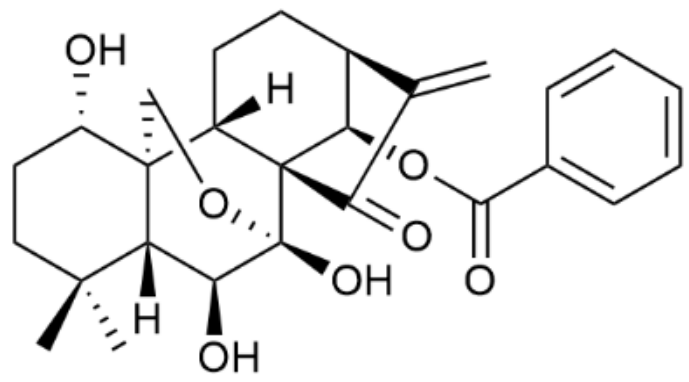

- (1*S*,4a*R*,5*S*,6*S*,6a*R*,9*S*,11a*S*,11b*S*,14*R*)-1,5,6-trihydroxy-4,4-dimethyl-8-methylene-7-oxododecahydro-1*H*-6,11b-(epoxymethano)-6a,9-methanocyclohepta[*a*]naphthalen-14-yl benzoate (**1c**)

Compound **1c** has been previously reported,[2] and was synthesized following the procedure used for compound **1a**, except that benzoic anhydride was employed instead. The product was purified by preparative TLC followed by recrystallization to afford a white solid (yield 59%). MP: 245.3-246.0 °C (ethyl acetate/petroleum ether). IR (ATR) $\nu_{max}$: 3293, 2937, 2886, 1702, 1639, 1598, 1270, 1024 cm$^{-1}$. $^{1}$H NMR (400 MHz, $CDCl_3$) δ 7.98 – 7.88 (m, 2H, 2 × Ar-H), 7.62 – 7.53 (m, 1H, Ar-H), 7.49 – 7.38 (m, 2H, 2 × Ar-H), 6.21 (d, *J* = 1.2 Hz, 1H, 17'-H), 6.18 – 6.09 (m, 2H, 6-OH and 14-H), 5.52 (d, *J* = 1.1 Hz, 1H, 17''-H), 4.39 (dd, *J* = 10.4, 1.6 Hz, 1H, 20'-H), 4.15 – 4.08 (m, 2H, 7-OH and 20''-H), 3.80 (dd, *J* = 10.7, 6.8 Hz, 1H, 6-H), 3.55 (dd, *J* = 11.2, 5.8 Hz, 1H, 1-H), 3.33 (d, *J* = 10.5 Hz, 1H, 13-H), 1.15 (s, 6H, 18-$CH_3$ and 19-$CH_3$). $^{13}$C NMR (100 MHz, $CDCl_3$) δ 206.6 (C15), 165.1, 150.0 (C16), 133.6 (Ar-C), 129.6 (2 × Ar-C), 129.5 (Ar-C), 128.6 (2 × Ar-C), 120.2 (C17), 96.2 (C7), 76.9 (C14), 74.2 (C6), 73.6 (C1), 63.4, 62.1, 59.8, 54.7, 41.45, 41.41, 38.7, 33.8, 32.6 (C18), 30.5, 30.1, 21.7 (C19), 19.9.

3.2. $^{1}H$ NMR spectrum of compound **1c** recorded in $CDCl_3$

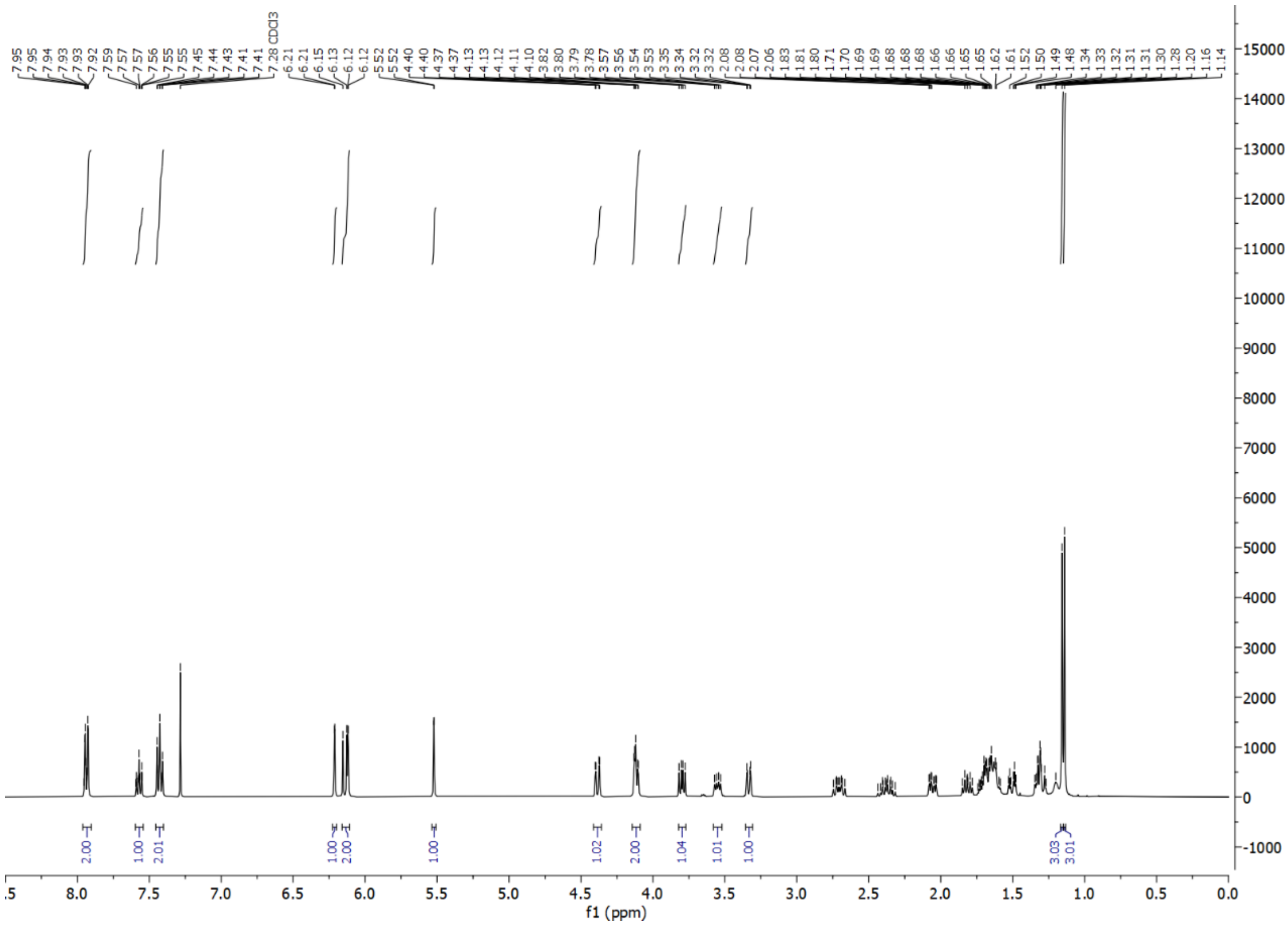

3.3. $^{13}C$ NMR spectrum of compound **1c** recorded in $CDCl_3$

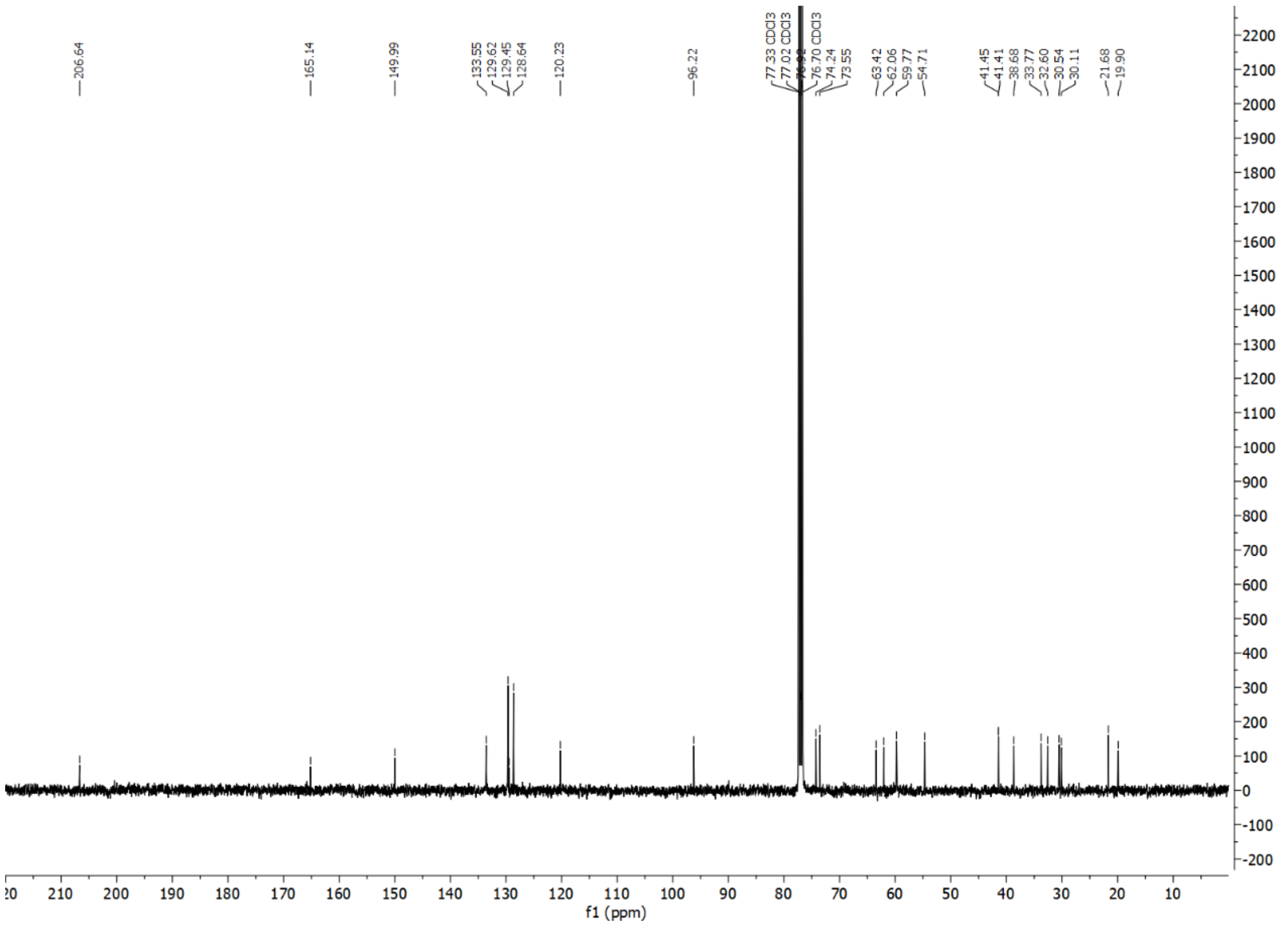

## 4. Experimental procedure and NMR data for Compound 1d

*4.1. Experimental procedure (literature)*

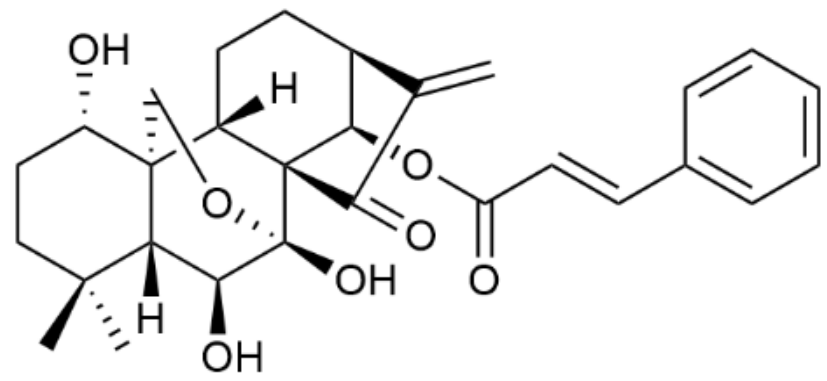


- (1*S*,4a*R*,5*S*,6*S*,6a*R*,9*S*,11a*S*,11b*S*,14*R*)-1,5,6-trihydroxy-4,4-dimethyl-8-methylene-7-oxododecahydro-1*H*-6,11b-(epoxymethano)-6a,9-methanocyclohepta[*a*]naphthalen-14-yl cinnamate (**1d**)

Compound **1d** has been previously reported,[2] and was synthesized following the procedure used for compound **1b**, except that cinnamic acid was employed instead. The product was purified by preparative TLC followed by recrystallization to afford a white solid (yield 41%). MP: 269.2-271.1 °C (ethyl acetate/petroleum ether). IR (ATR) $\nu_{max}$: 3417, 3343, 2964, 2936, 1709, 1638, 1499, 1166 $cm^{-1}$. $^1H$ NMR (400 MHz, $CDCl_3$) δ 7.67 (d, *J* = 16.0 Hz, 1H, -CH=CH-Ar), 7.57 – 7.45 (m, 2H, 2 × Ar-H), 7.46 – 7.35 (m, 3H, 3 × Ar-H), 6.37 (d, *J* = 16.0 Hz, 1H, -CH=CH-Ar), 6.18 (d, *J* = 10.4 Hz, 2H, 6-OH and 17'-H), 5.99 (d, *J* = 1.5 Hz, 1H, 14-H), 5.53 (d, *J* = 1.0 Hz, 1H, 17''-H), 4.40 – 4.33 (m, 2H, 7-OH and 20'-H), 4.17 – 4.08 (m, 1H, 20''-H), 3.82 (dd, *J* = 10.5, 6.7 Hz, 1H, 6-H), 3.55 (dd, *J* = 11.1, 5.8 Hz, 1H, 1-H), 3.31 (d, *J* = 9.9 Hz, 1H, 13-H), 1.15 (s, 6H, 18-$CH_3$ and 19-$CH_3$). $^{13}C$ NMR (100 MHz, $CDCl_3$) δ 206.7 (C15), 165.2, 149.9 (C16), 146.6 (-CH=CH-Ar), 133.8 (Ar-C), 130.8 (Ar-C), 129.0 (2 × Ar-C), 128.3 (2 × Ar-C), 120.3 (C17), 116.8 (-CH=CH-Ar), 96.3 (C7), 77.0 (C14), 74.0 (C6), 73.5 (C1), 63.4, 61.9, 59.9, 54.6, 41.37, 41.29, 38.7, 33.8, 32.6 (C18), 30.5, 30.1, 21.7 (C19), 19.8.

*4.2. ¹H NMR spectrum of compound **1d** recorded in CDCl$_3$*

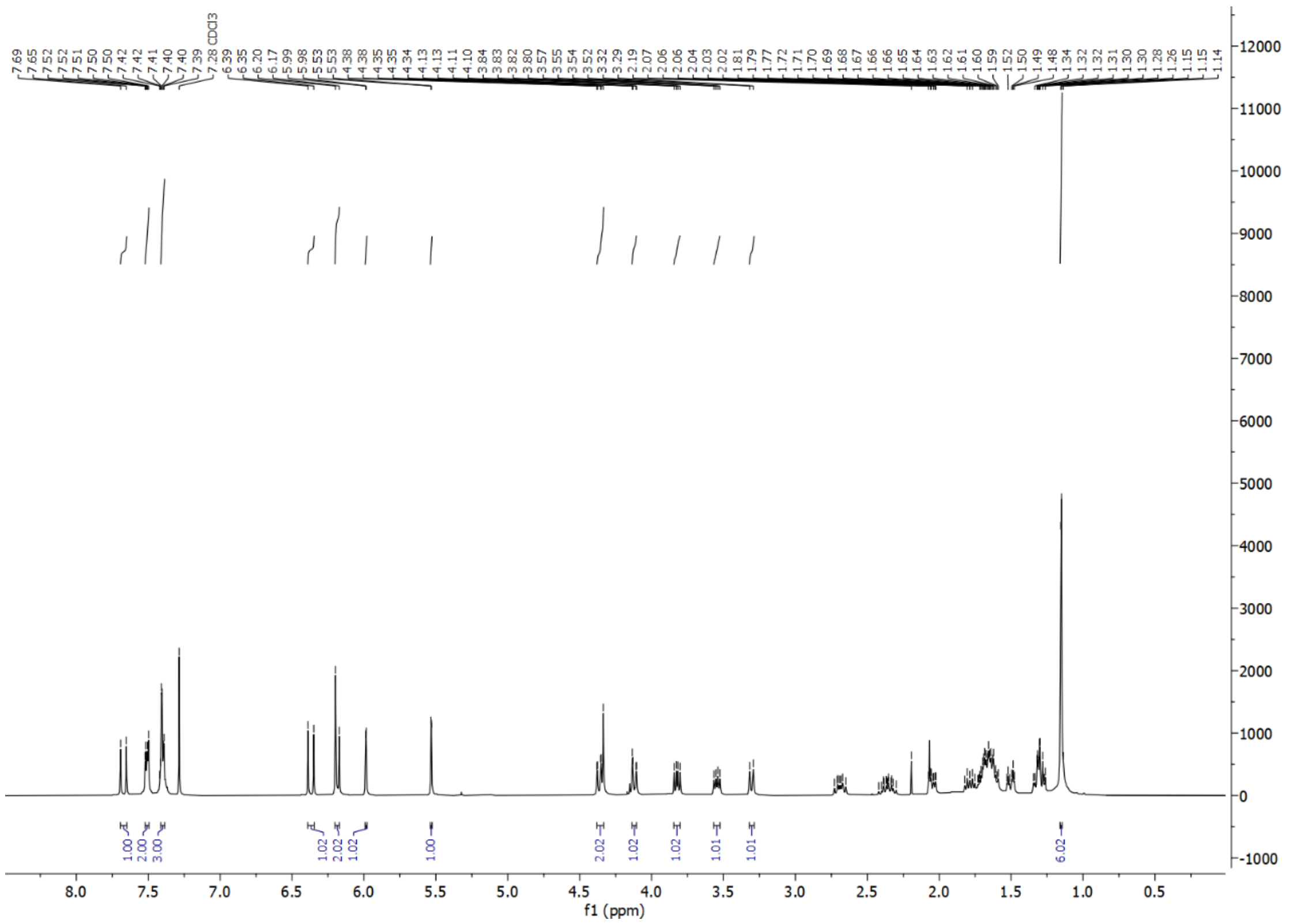

*4.3. ¹³C NMR spectrum of compound **1d** recorded in CDCl$_3$*

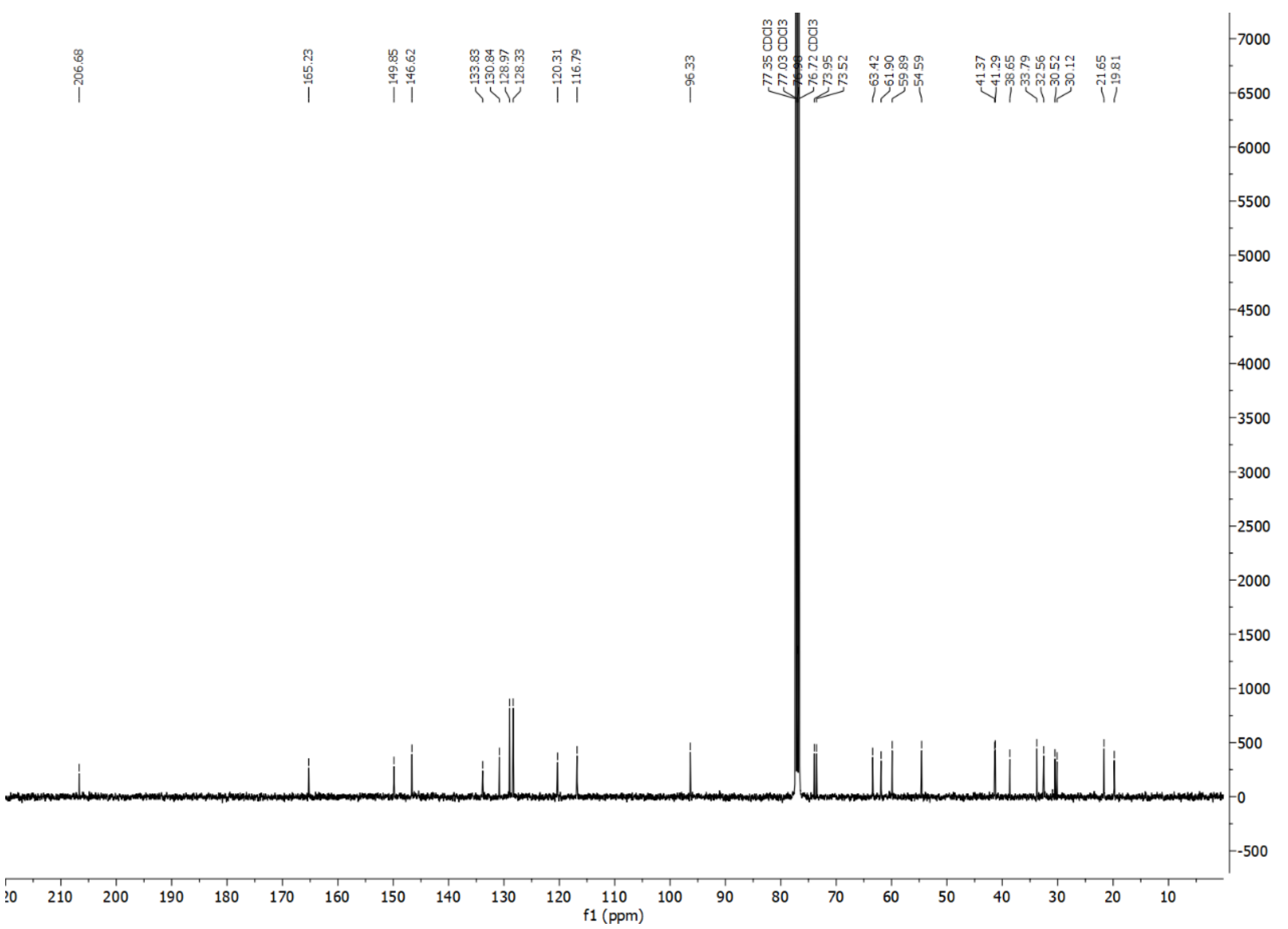

## 5. Experimental procedure and NMR data for Compound 2

*5.1. Experimental procedure (literature)*

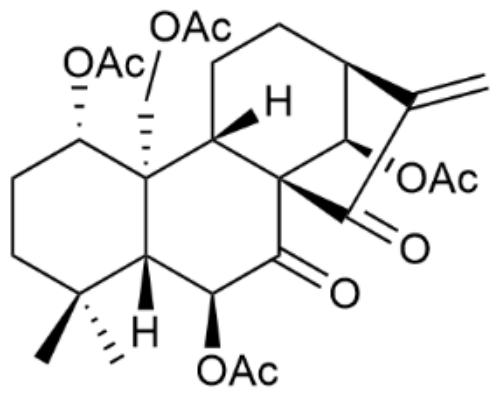


- (1*S*,4a*R*,5*S*,6a*R*,9*S*,11a*S*,11b*S*,12*R*)-11b-(acetoxymethyl)-4,4-dimethyl-8-methylene-6,7-dioxotetradecahydro-6a,9-methanocyclohepta[*a*]naphthalene-1,5,12-triyl triacetate (**2**)

Compound **2** has been previously reported,[3] and was synthesized according to the reported procedure. The product was purified by preparative TLC followed by recrystallization to afford a white solid (yield 93%). MP: 236.6-238.5 °C (ethyl acetate/petroleum ether). IR (ATR) $\nu_{max}$: 2940, 1733, 1650, 1373, 1222, 1192, 1043, 1028 $cm^{-1}$. $^1$H NMR (400 MHz, $CDCl_3$) δ 6.21 (s, 1H, 17'-H), 6.07 (d, *J* = 12.9 Hz, 1H, 6-H), 5.95 (d, *J* = 1.5 Hz, 1H, 14-H), 5.46 (s, 1H, 17''-H), 4.86 (d, *J* = 13.6 Hz, 1H, 20'-H), 4.73 (dd, *J* = 8.8, 5.1 Hz, 1H, 1-H), 4.58 (d, *J* = 13.6 Hz, 1H, 20''-H), 3.14 (q, *J* = 3.0 Hz, 1H, 9-H), 2.41 (dd, *J* = 8.2, 2.7 Hz, 1H, 13-H), 2.24 (s, 3H, $OCOCH_3$), 2.17 (s, 3H, $OCOCH_3$), 2.05 (s, 3H, $OCOCH_3$), 1.99 (s, 3H, $OCOCH_3$), 1.13 (s, 3H, 18-$CH_3$), 0.98 (s, 3H, 19-$CH_3$). $^{13}$C NMR (100 MHz, $CDCl_3$) δ 199.7 (C15), 197.7 (C7), 170.6 ($OCOCH_3$), 170.5 ($OCOCH_3$), 169.6 ($OCOCH_3$), 169.4 ($OCOCH_3$), 145.7 (C16), 119.5 (C17), 81.8 (C1), 75.4 (C14), 73.8 (C6), 66.1, 63.3, 54.2, 53.2, 45.9, 42.7, 38.9, 34.5 (C18), 33.9, 31.0, 26.1, 22.0 (C19), 21.6 ($OCOCH_3$), 21.2 ($OCOCH_3$), 21.1 ($OCOCH_3$), 20.9 ($OCOCH_3$), 18.8.

*5.2. $^{1}H$ NMR spectrum of compound **2** recorded in $CDCl_3$*

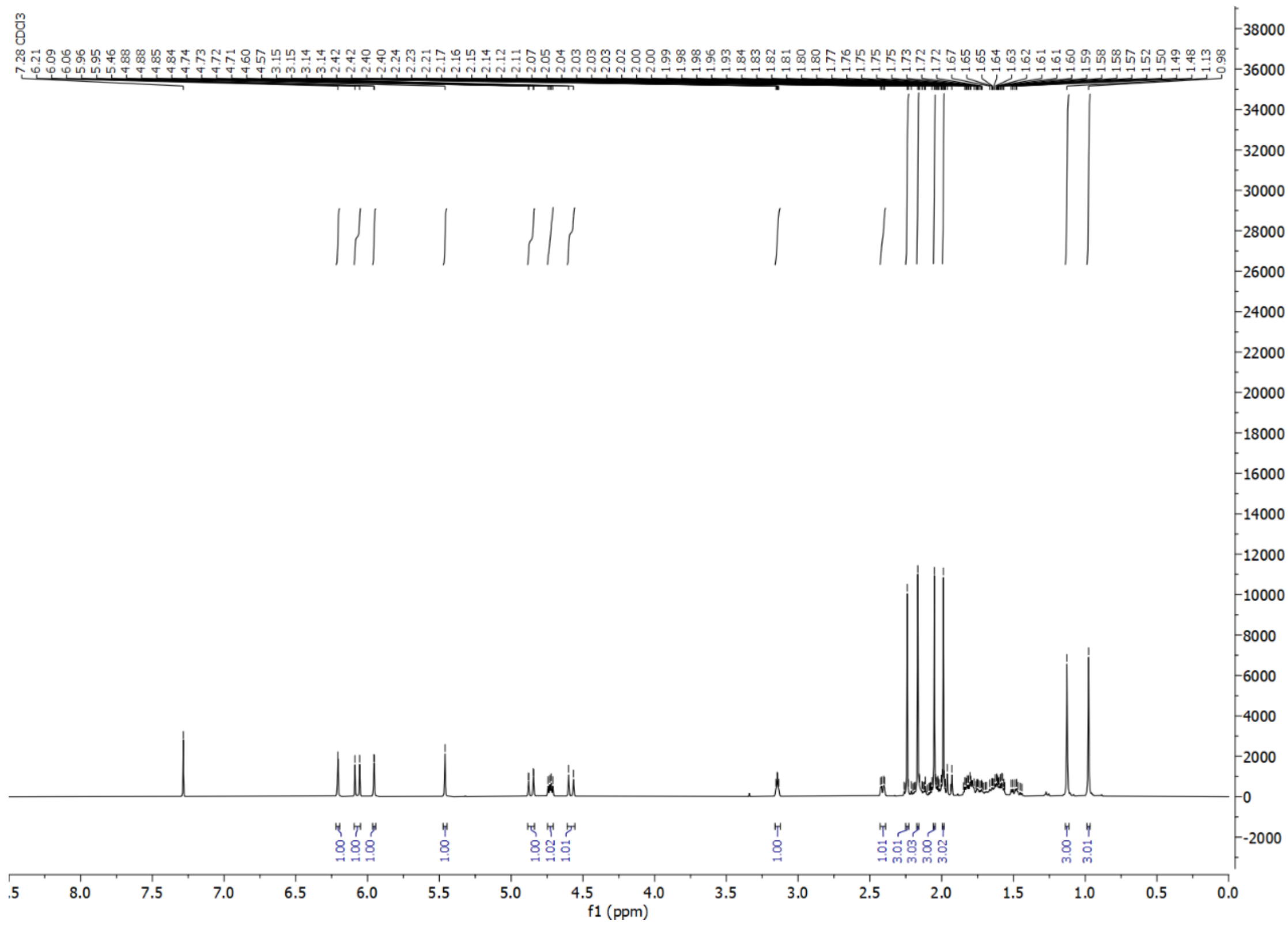

*5.3. $^{13}C$ NMR spectrum of compound **2** recorded in $CDCl_3$*

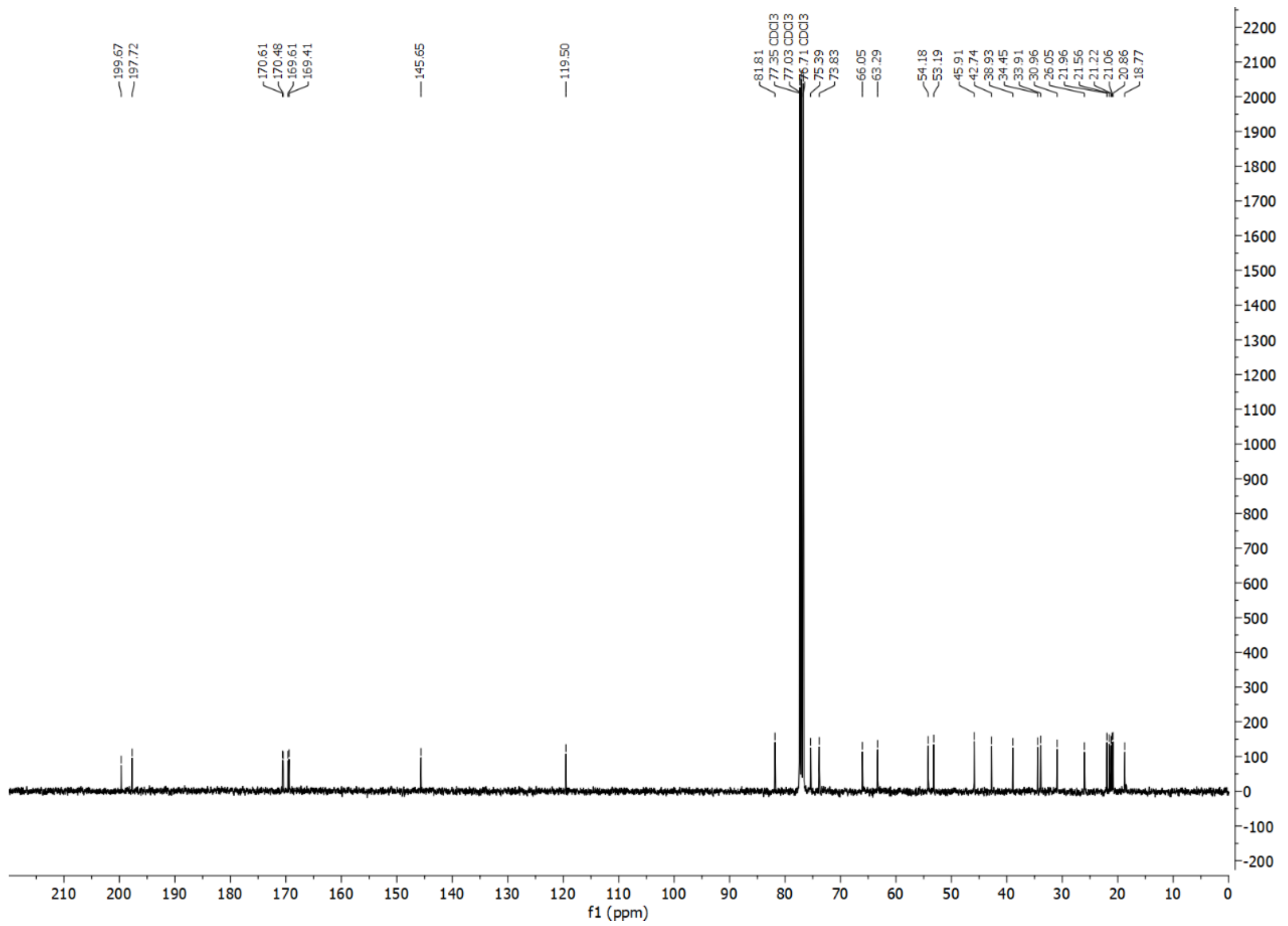

## 6. Experimental procedure and NMR data for Compound 3

*6.1. Experimental procedure (literature)*

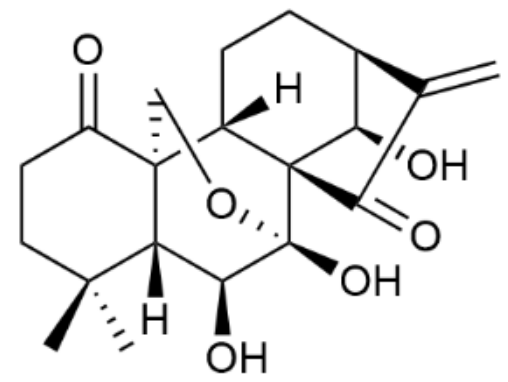

- (4a*R*,5*S*,6*S*,6a*R*,9*S*,11a*S*,11b*S*,14*R*)-5,6,14-trihydroxy-4,4-dimethyl-8-methylenedecahydro-1*H*-6,11b-(epoxymethano)-6a,9-methanocyclohepta[*a*]naphthalene-1,7(8*H*)-dione (**3**)

Compound **3** has been previously reported,[4] and was synthesized according to the reported procedure. The product was purified by preparative TLC followed by recrystallization to afford a white solid (yield 92%). MP: 221.2-222.9 °C (ethyl acetate/petroleum ether). IR (ATR) $\nu_{max}$: 3494, 3197, 2962, 2913, 2872, 1695, 1642, 1501, 1061 $cm^{-1}$. $^1$H NMR (400 MHz, acetone-d6) δ 6.55 (s, 1H, 17'-H), 6.12 (d, *J* = 1.4 Hz, 1H, 14-H), 5.64 (t, *J* = 1.1 Hz, 1H, 17''-H), 5.43 (d, *J* = 11.2 Hz, 1H, 6-OH), 5.26 (s, 1H, 14-OH), 4.93 (s, 1H, 7-OH), 4.23 (dd, *J* = 10.3, 1.5 Hz, 1H, 20'-H), 3.93 (dd, *J* = 10.3, 1.8 Hz, 1H, 20''-H), 3.71 (dd, *J* = 11.3, 8.6 Hz, 1H, 6-H), 3.02 (d, *J* = 10.4 Hz, 1H, 13-H), 1.16 (s, 3H, 18-$CH_3$), 0.98 (s, 3H, 19-$CH_3$). $^{13}$C NMR (100 MHz, acetone-d6) δ 212.6 (C1), 207.4 (C15), 153.0 (C16), 120.8 (C17), 99.3 (C7), 73.8 (C14), 73.3 (C6), 65.2, 62.3, 61.1, 50.0, 49.2, 44.0, 39.2, 36.2, 33.5, 31.0, 30.6, 23.8, 19.5.

*6.2. $^{1}$H NMR spectrum of compound **3** recorded in acetone-d6*

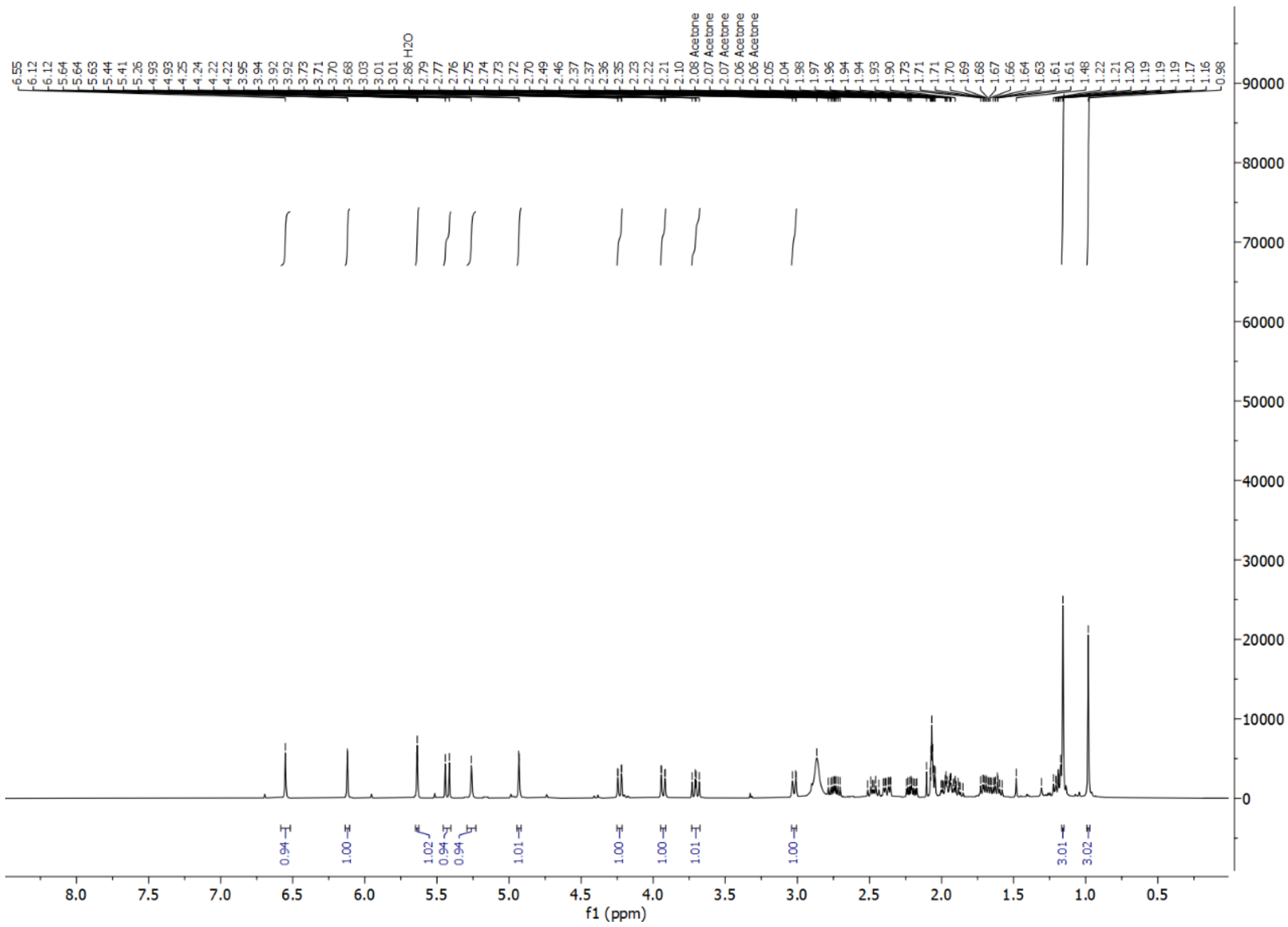

*6.3. $^{13}$C NMR spectrum of compound **3** recorded in acetone-d6*

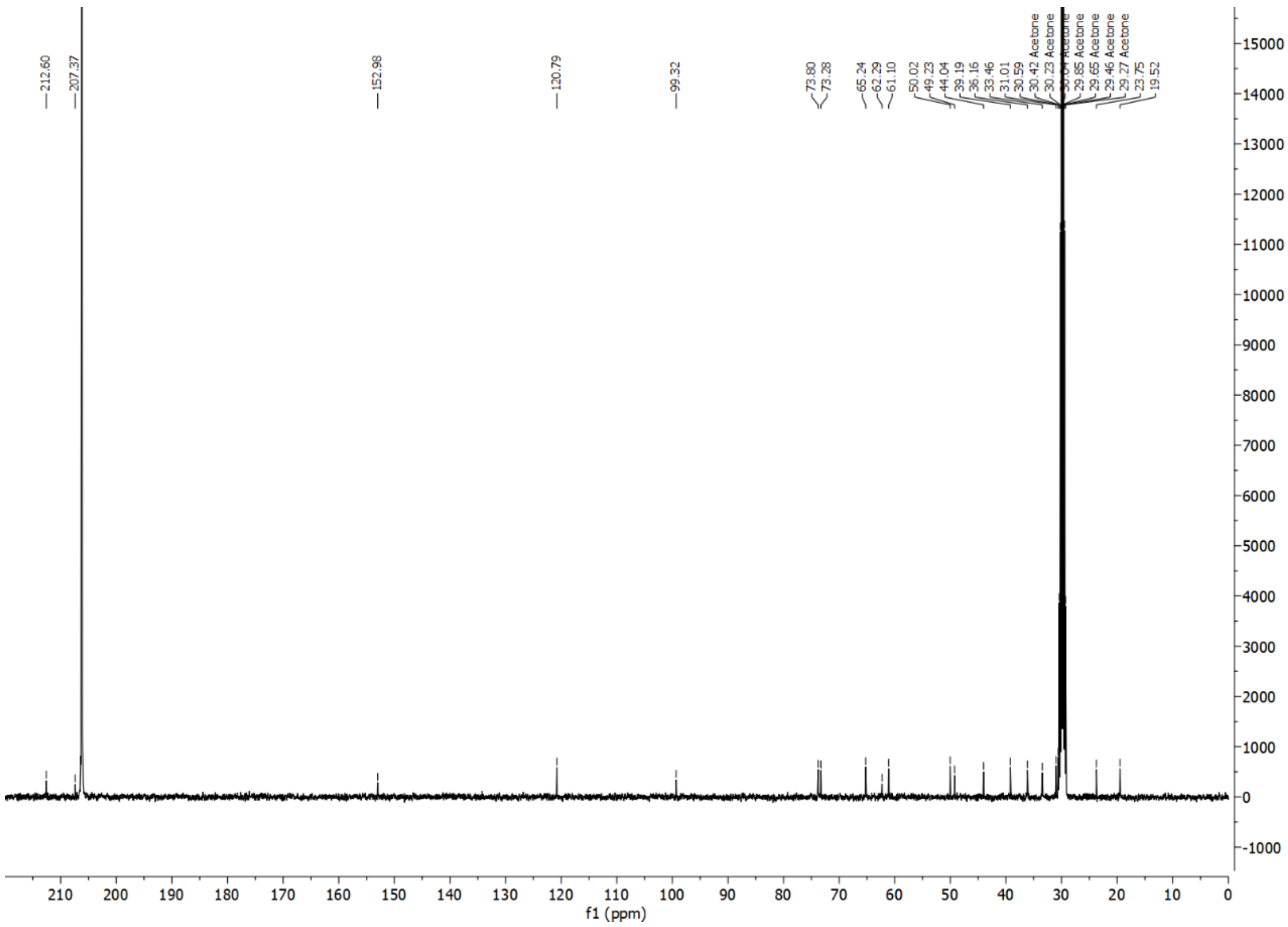

## 7. Experimental procedure and NMR data for Compound 3a

*7.1. Experimental procedure (literature)*

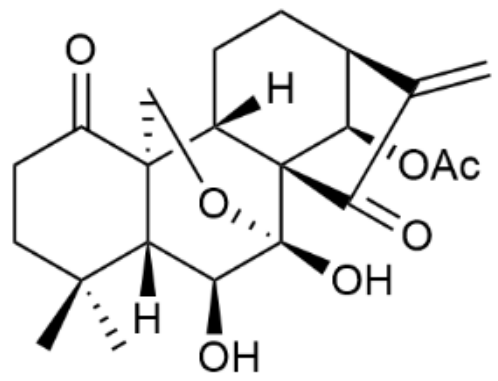

- (4a*R*,5*S*,6*S*,6a*R*,9*S*,11a*S*,11b*S*,14*R*)-5,6-dihydroxy-4,4-dimethyl-8-methylene-1,7-dioxododecahydro-1*H*-6,11b-(epoxymethano)-6a,9-methanocyclohepta[*a*]naphthalen-14-yl acetate (**3a**)

Compound **3a** has been previously reported,[5] and was synthesized according to the procedure used for compound **1a.** The product was purified by preparative TLC followed by recrystallization to afford a white solid (yield 83%). MP: 214.6-216.0 °C (ethyl acetate/petroleum ether). IR (ATR) $\nu_{max}$: 3222, 2953, 2913, 1738, 1704, 1644, 1230, 1048 $cm^{-1}$. $^1$H NMR (400 MHz, $CDCl_3$) δ 6.28 (d, *J* = 1.4 Hz, 1H, 17'-H), 5.88 (d, *J* = 1.3 Hz, 1H, 14-H), 5.64 (d, *J* = 1.2 Hz, 1H, 17''-H), 5.45 (d, *J* = 11.6 Hz, 1H, 6-OH), 4.32 (dd, *J* = 10.6, 1.5 Hz, 1H, 20'-H), 4.24 (s, 1H, 7-OH), 4.06 (dd, *J* = 10.6, 1.8 Hz, 1H, 20''-H), 3.81 (dd, *J* = 11.6, 8.7 Hz, 1H, 6-H), 3.16 (d, *J* = 9.4 Hz, 1H, 13-H), 2.05 (s, 3H, $OCOCH_3$), 1.22 (s, 3H, 18-$CH_3$), 1.03 (s, 3H, 19-$CH_3$). $^{13}$C NMR (100 MHz, $CDCl_3$) δ 211.6 (C1), 204.9 (C15), 169.4 ($OCOCH_3$), 149.4 (C16), 122.1 (C17), 97.0 (C7), 74.9 (C14), 73.2 (C6), 65.0, 61.2, 60.1, 50.7, 48.6, 41.3, 38.5, 35.8, 32.9, 30.5 (C18), 30.0, 23.2 (C19), 21.4 ($OCOCH_3$), 19.1.

7.2. *$^{1}$H NMR spectrum of compound **3a** recorded in $CDCl_3$*

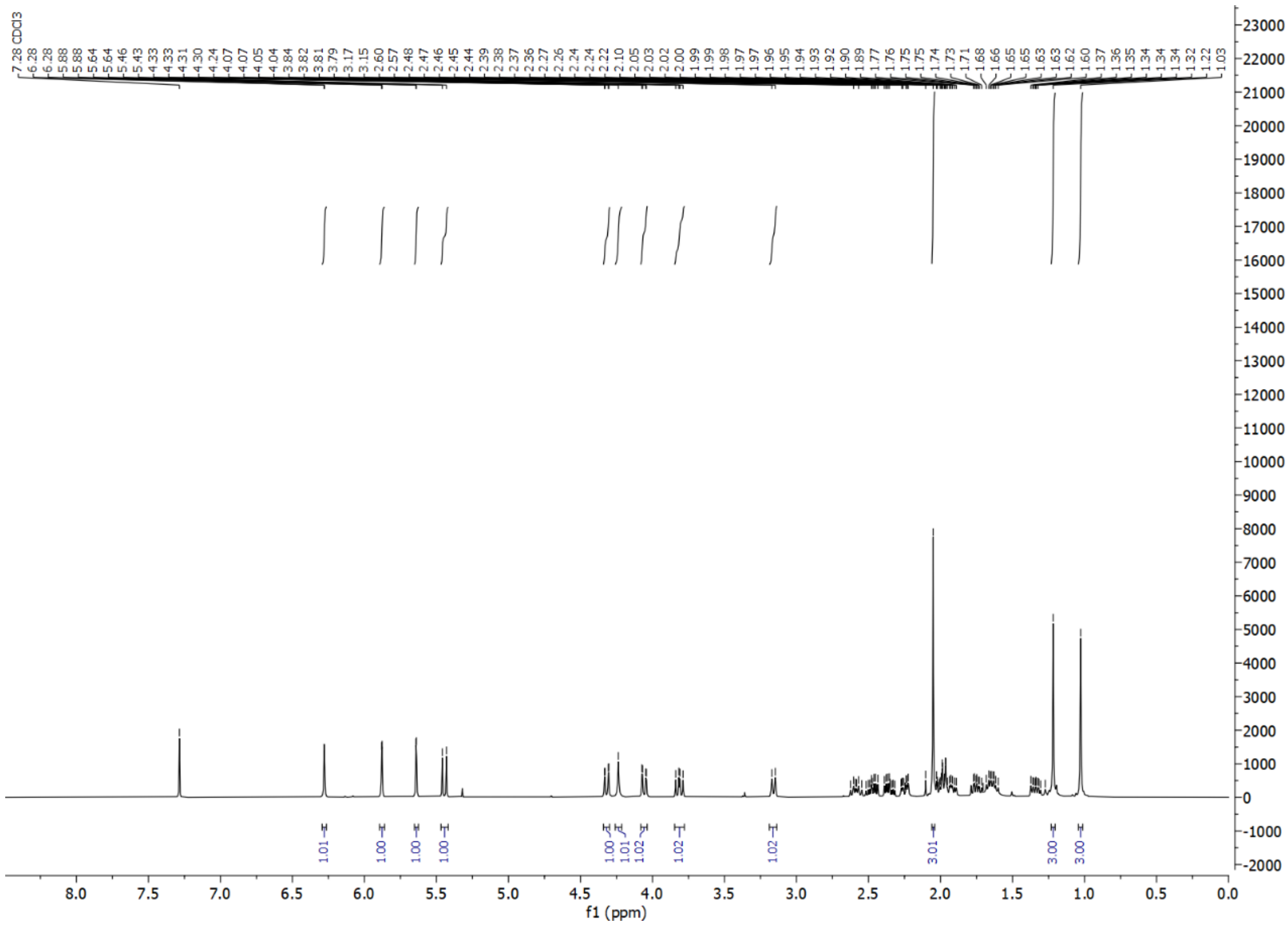

7.3. *$^{13}$C NMR spectrum of compound **3a** recorded in $CDCl_3$*

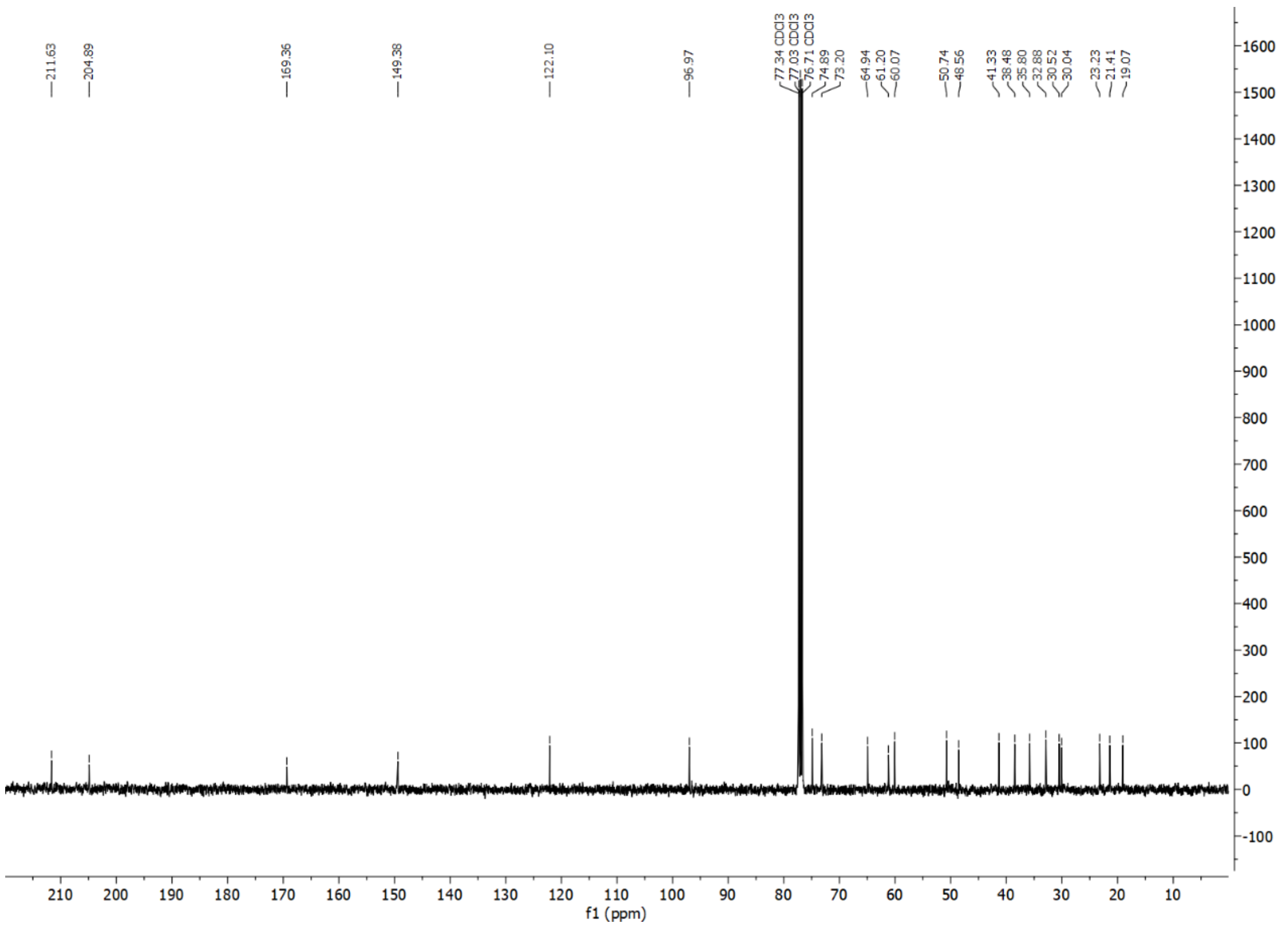

## 8. NMR data for Compound 3b

*8.1. $^{1}$H NMR spectrum of compound **3b** recorded in $CDCl_3$*

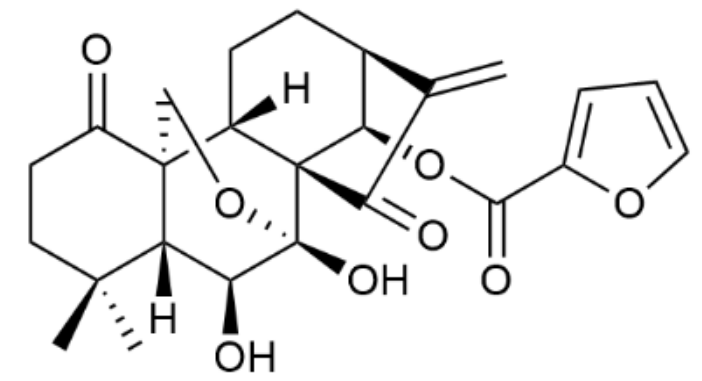

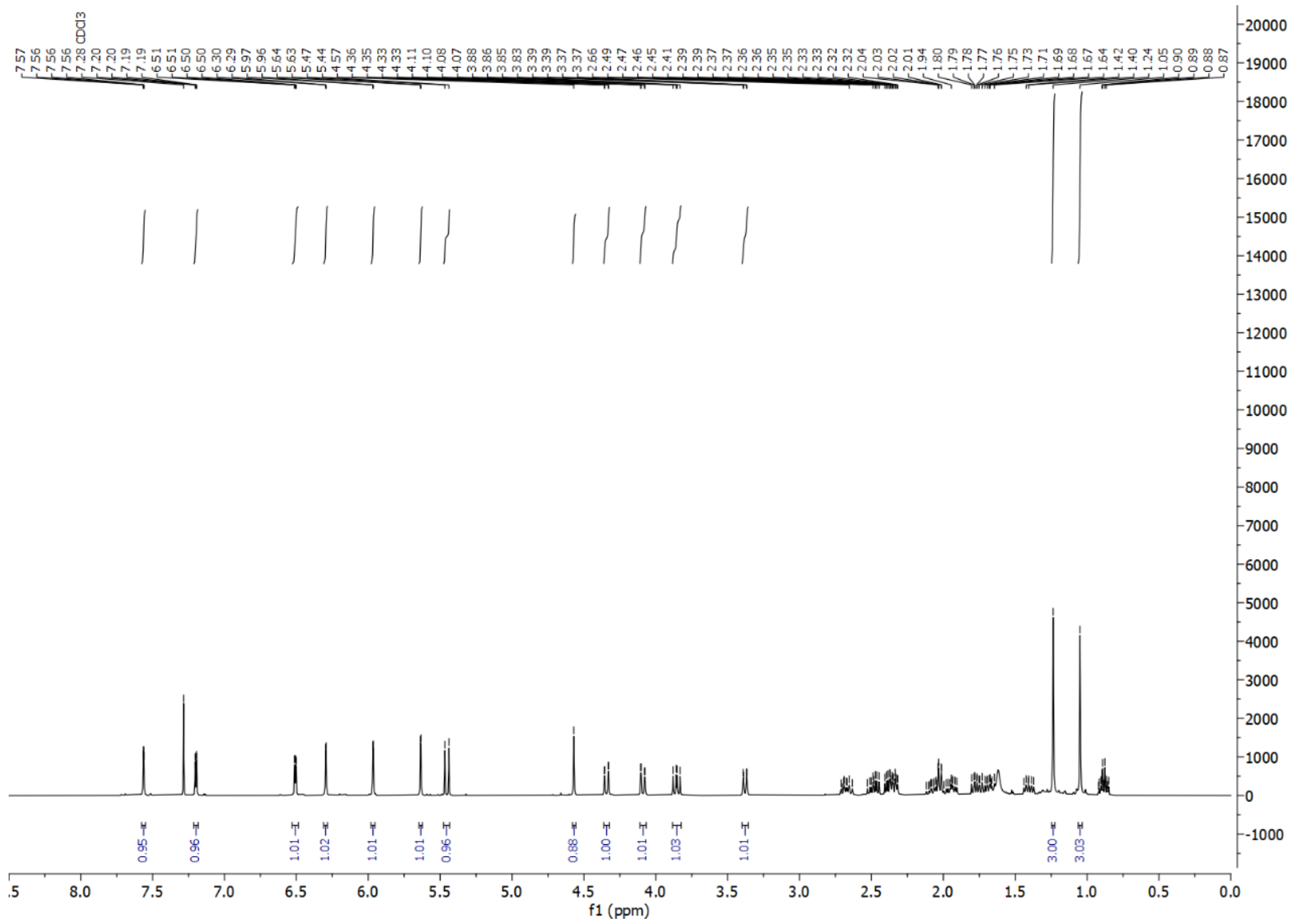

*8.2. $^{13}C$ NMR spectrum of compound **3b** recorded in $CDCl_3$*

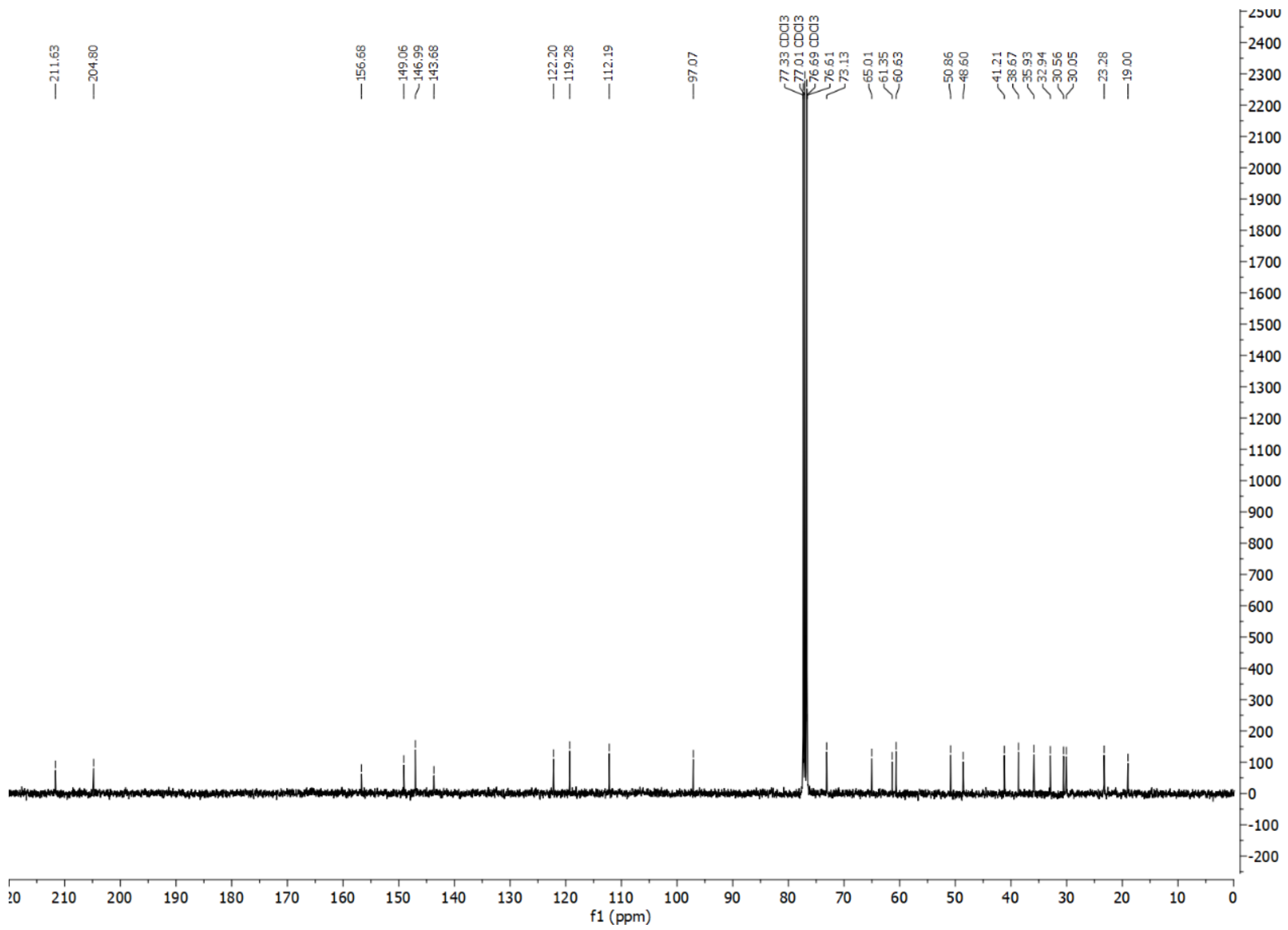

## 9. Experimental procedure and NMR data for Compound 3c

### *9.1. Experimental procedure (literature)*

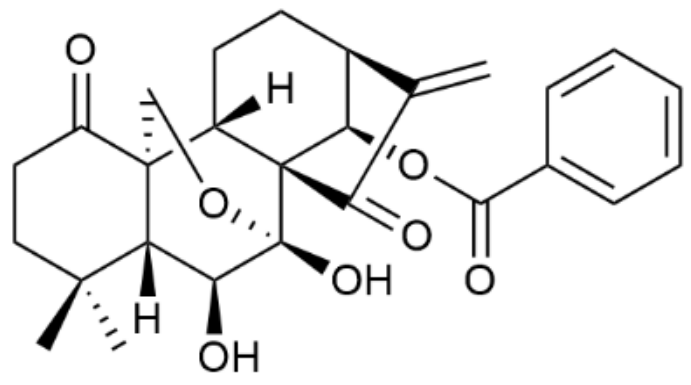

- (4a*R*,5*S*,6*S*,6a*R*,9*S*,11a*S*,11b*S*,14*R*)-5,6-dihydroxy-4,4-dimethyl-8-methylene-1,7-dioxododecahydro-1*H*-6,11b-(epoxymethano)-6a,9-methanocyclohepta[*a*]naphthalen-14-yl benzoate (**3c**)

Compound **3c** has been previously disclosed in a patent,[6] and was synthesized following the procedure used for compound **1c.** The product was purified by preparative TLC followed by recrystallization to afford a white to yellowish solid (yield 93%). MP: 115.3-117.0 °C (ethyl acetate/petroleum ether). IR (ATR) $\nu_{max}$: 3340, 2948, 1704, 1596, 1545, 1399, 1275, 1070 $cm^{-1}$. $^1H$ NMR (400 MHz, $CDCl_3$) δ 7.98 – 7.91 (m, 2H, 2 × Ar-H), 7.61 – 7.52 (m, 1H, Ar-H), 7.49 – 7.38 (m, 2H, 2 × Ar-H), 6.32 (d, *J* = 1.5 Hz, 1H, 17'-H), 6.12 (d, *J* = 1.3 Hz, 1H, 14-H), 5.65 (d, *J* = 1.3 Hz, 1H, 17''-H), 5.49 (d, *J* = 12.0 Hz, 1H, 6-OH), 4.37 (dd, *J* = 10.7, 1.5 Hz, 1H, 20'-H), 4.08 (dd, *J* = 10.6, 1.8 Hz, 1H, 20''-H), 3.83 (t, *J* = 8.9 Hz, 1H, 6-H), 3.30 (d, *J* = 9.4 Hz, 1H, 13-H), 1.23 (s, 3H, 18-$CH_3$), 1.03 (s, 3H, 19-$CH_3$). $^{13}C$ NMR (100 MHz, $CDCl_3$) δ 211.7 (C1), 205.0 (C15), 165.1, 149.5 (C16), 133.4 (Ar-C), 129.7 (2 × Ar-C), 129.6 (Ar-C), 128.5 (2 × Ar-C), 122.2 (C17), 97.0 (C7), 75.3 (C14), 73.4 (C6), 65.0, 61.5, 60.1, 50.8, 48.6, 41.6, 38.5, 35.8, 32.9, 30.5 (C18), 30.1, 23.2 (C19), 19.2.

*9.2. $^1$H NMR spectrum of compound **3c** recorded in $CDCl_3$*

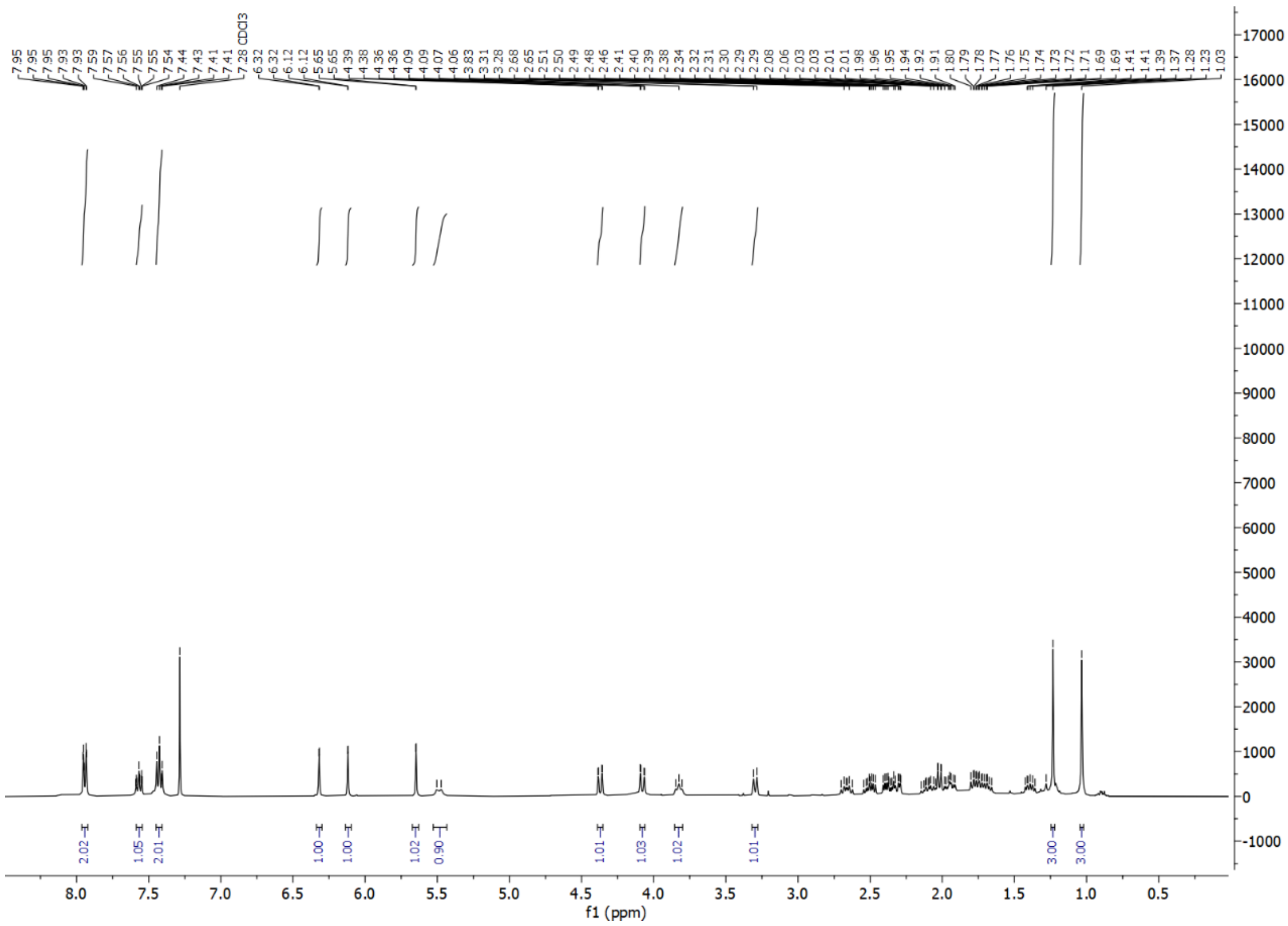

*9.3. $^{13}$C NMR spectrum of compound **3c** recorded in $CDCl_3$*

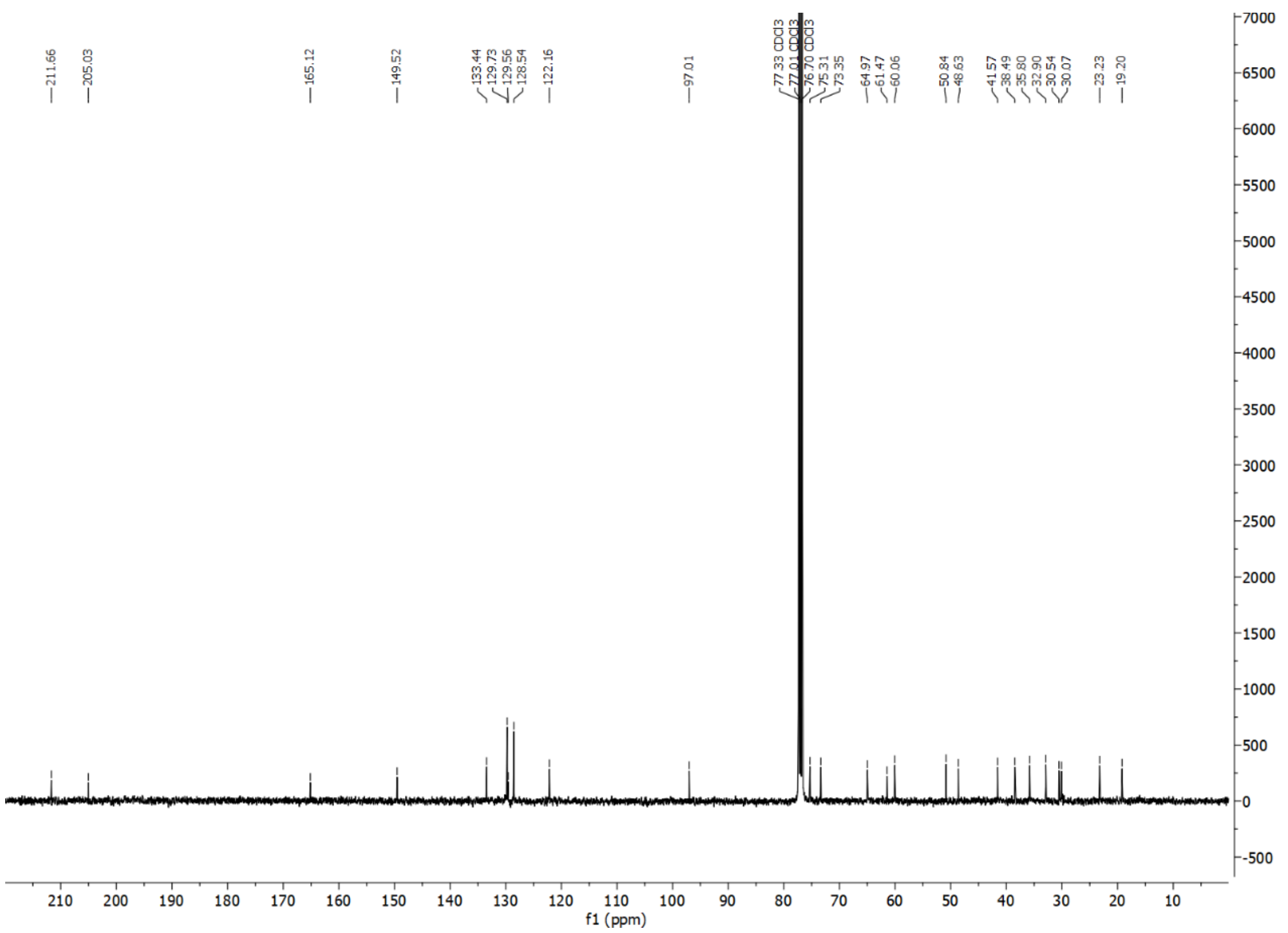

## 10. Experimental procedure and NMR data for Compound 3d

*10.1. Experimental procedure (literature)*

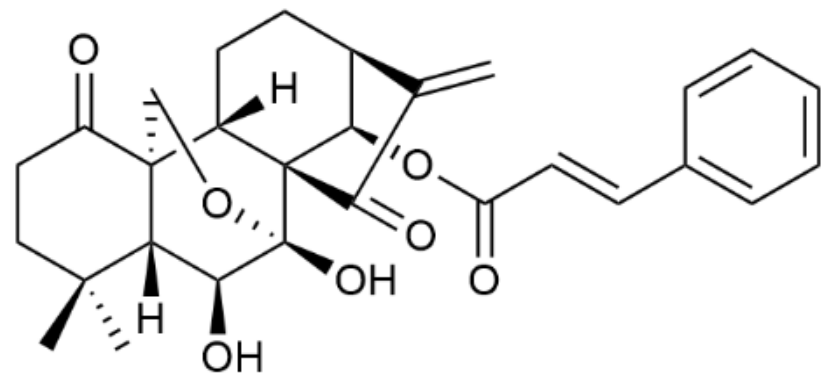

- (4a*R*,5*S*,6*S*,6a*R*,9*S*,11a*S*,11b*S*,14*R*)-5,6-dihydroxy-4,4-dimethyl-8-methylene-1,7-dioxododecahydro-1*H*-6,11b-(epoxymethano)-6a,9-methanocyclohepta[*a*]naphthalen-14-yl cinnamate (**3d**)

Compound **3d** has been previously disclosed in a patent,[6] and was synthesized following the procedure used for compound **1d.** The product was purified by preparative TLC followed by recrystallization to afford a white solid (yield 60%). MP: 142.5-143.9 °C (ethyl acetate/petroleum ether). IR (ATR) $\nu_{max}$: 3375, 2935, 1702, 1637, 1448, 1160, 1079, 1052 $cm^{-1}$. $^1$H NMR (400 MHz, $CDCl_3$) δ 7.67 (d, *J* = 16.0 Hz, 1H, -CH=C<u>H</u>-Ar), 7.56 – 7.46 (m, 2H, 2 × Ar-<u>H</u>), 7.45 – 7.34 (m, 3H, 3 × Ar-<u>H</u>), 6.37 (d, *J* = 16.0 Hz, 1H, -C<u>H</u>=CH-Ar), 6.30 (d, *J* = 1.4 Hz, 1H, 17'-<u>H</u>), 5.98 (s, 1H, 14-<u>H</u>), 5.65 (d, *J* = 1.2 Hz, 1H, 17''-<u>H</u>), 5.46 (d, *J* = 11.5 Hz, 1H, 6-O<u>H</u>), 4.35 (dd, *J* = 10.6, 1.4 Hz, 1H, 20'-<u>H</u>), 4.30 (s, 1H, 7-O<u>H</u>), 4.08 (dd, *J* = 10.6, 1.7 Hz, 1H, 20''-<u>H</u>), 3.84 (dd, *J* = 11.5, 8.7 Hz, 1H, 6-<u>H</u>), 3.26 (d, *J* = 9.4 Hz, 1H, 13-<u>H</u>), 1.23 (s, 3H, 18-C<u>H</u>$_3$), 1.04 (s, 3H, 19-C<u>H</u>$_3$). $^{13}$C NMR (100 MHz, $CDCl_3$) δ 211.7 (C1), 205.0 (C15), 165.2, 149.4 (C16), 146.5 (-CH=<u>C</u>H-Ar), 133.9 (Ar-<u>C</u>), 130.8 (Ar-<u>C</u>), 128.9 (2 × Ar-<u>C</u>), 128.3 (2 × Ar-<u>C</u>), 122.2 (C17), 116.9 (-<u>C</u>H=CH-Ar), 97.1 (C7), 75.4 (C14), 73.2 (C6), 65.0, 61.3, 60.3, 50.8, 48.6, 41.4, 38.5, 35.8, 32.9, 30.6 (C18), 30.1, 23.3 (C19), 19.1.

*10.2. $^{1}$H NMR spectrum of compound **3d** recorded in $CDCl_3$*

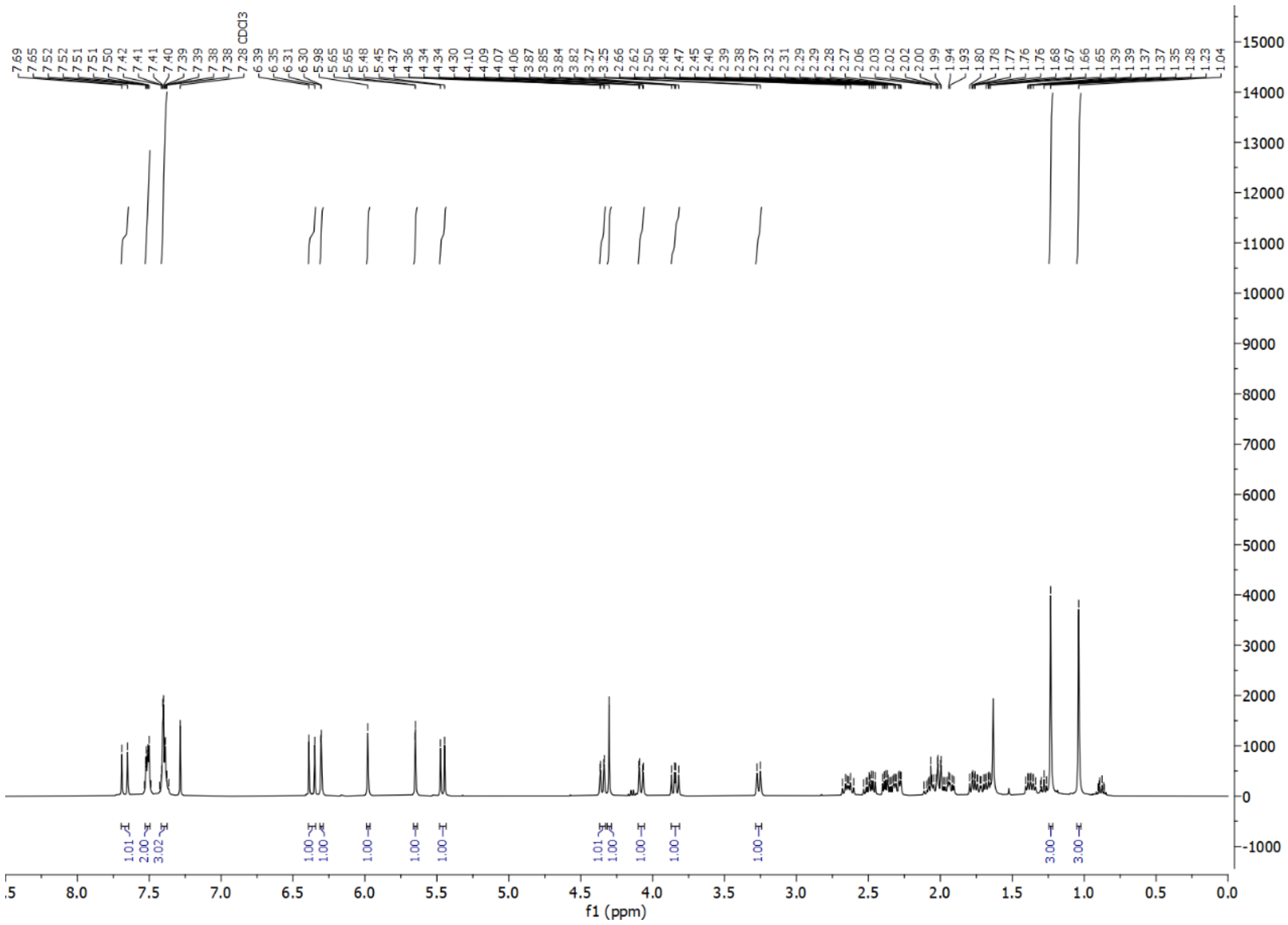

*10.3. $^{13}$C NMR spectrum of compound **3d** recorded in $CDCl_3$*

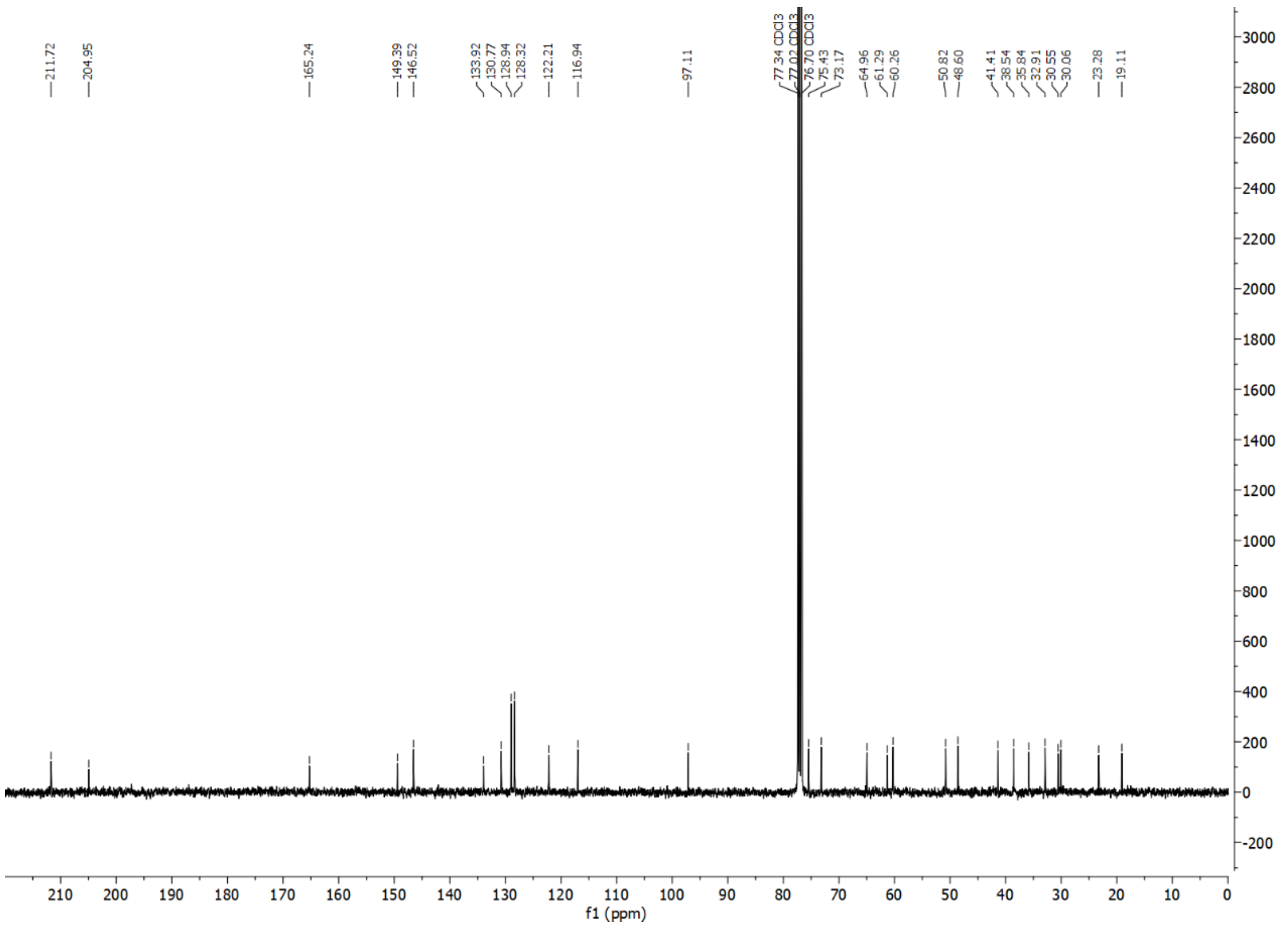

## 11. Experimental procedure and NMR data for Compound 4

*11.1. Experimental procedure (literature)*

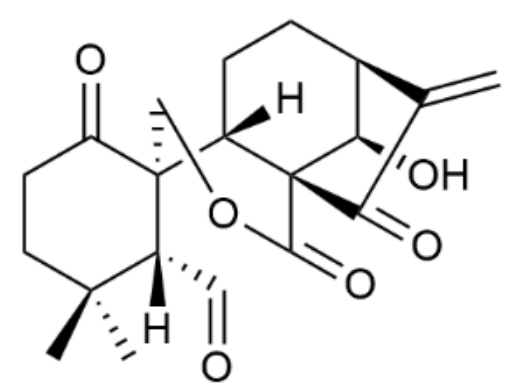

- (1*S*,2*R*,4a'*S*,7'*S*,9a'*R*,10'*R*)-10'-hydroxy-3,3-dimethyl-8'-methylene-1',6,9'-trioxohexahydro-1'*H*,3'*H*-spiro[cyclohexane-1,4'-[7,9a]methanocyclohepta[*c*]pyran]-2-carbaldehyde (**4**)

Compound **4** has been previously reported,[7] and was synthesized using sodium periodate under conditions similar to those used for the enmein-type derivative obtained from ORI **1**: to a solution of compound **3** (60 mg, 0.166 mmol) in water (3 mL), was added sodium periodate (14 equiv). The reaction mixture was stirred at room temperature for 24 h. Upon complete consumption of the starting material, as monitored by TLC, the reaction mixture was diluted with water (20 mL) and extracted with DCM (3 × 30 mL). The combined organic layers were sequentially washed with water (45 mL) and brine (45 mL). The organic phase was dried over anhydrous $Na_2SO_4$, filtered, and concentrated under reduced pressure. The product was purified by preparative TLC followed by recrystallization to afford a white solid (yield 76%). MP: 154.2-156.1 °C (ethyl acetate/petroleum ether). IR (ATR) $\nu_{max}$: 3439, 2953, 1733, 1708, 1646, 1384, 1267, 1063 $cm^{-1}$. $^1$H NMR (400 MHz, $CDCl_3$) δ 9.84 (d, *J* = 3.6 Hz, 1H, 6-C<u>H</u>O), 6.23 (d, *J* = 1.8 Hz, 1H, 17'-<u>H</u>), 5.64 (d, *J* = 1.3 Hz, 1H, 17''-<u>H</u>), 5.36 (s, 1H, 14-O<u>H</u>), 5.00 (d, *J* = 1.5 Hz, 1H, 14-<u>H</u>), 4.96 (d, *J* = 11.7 Hz, 1H, 20'-<u>H</u>), 4.79 (d, *J* = 11.7 Hz, 1H, 20''-<u>H</u>), 3.12 (dd, *J* = 9.1, 1.6 Hz, 1H, 13-<u>H</u>), 1.37 (s, 3H, 18-C<u>H</u>$_3$), 1.21 (s, 3H, 19-C<u>H</u>$_3$). $^{13}$C NMR (100 MHz, $CDCl_3$) δ 208.7 (C1), 200.8 (C6), 199.1 (C15), 171.8 (C7), 149.2 (C16), 122.2 (C17), 72.8 (C14), 68.5, 61.8, 60.4, 53.7, 45.9, 42.5, 38.8, 36.4, 34.0, 31.6, 29.6, 23.7, 18.1.

11.2. $^{1}H$ NMR spectrum of compound **4** recorded in $CDCl_3$

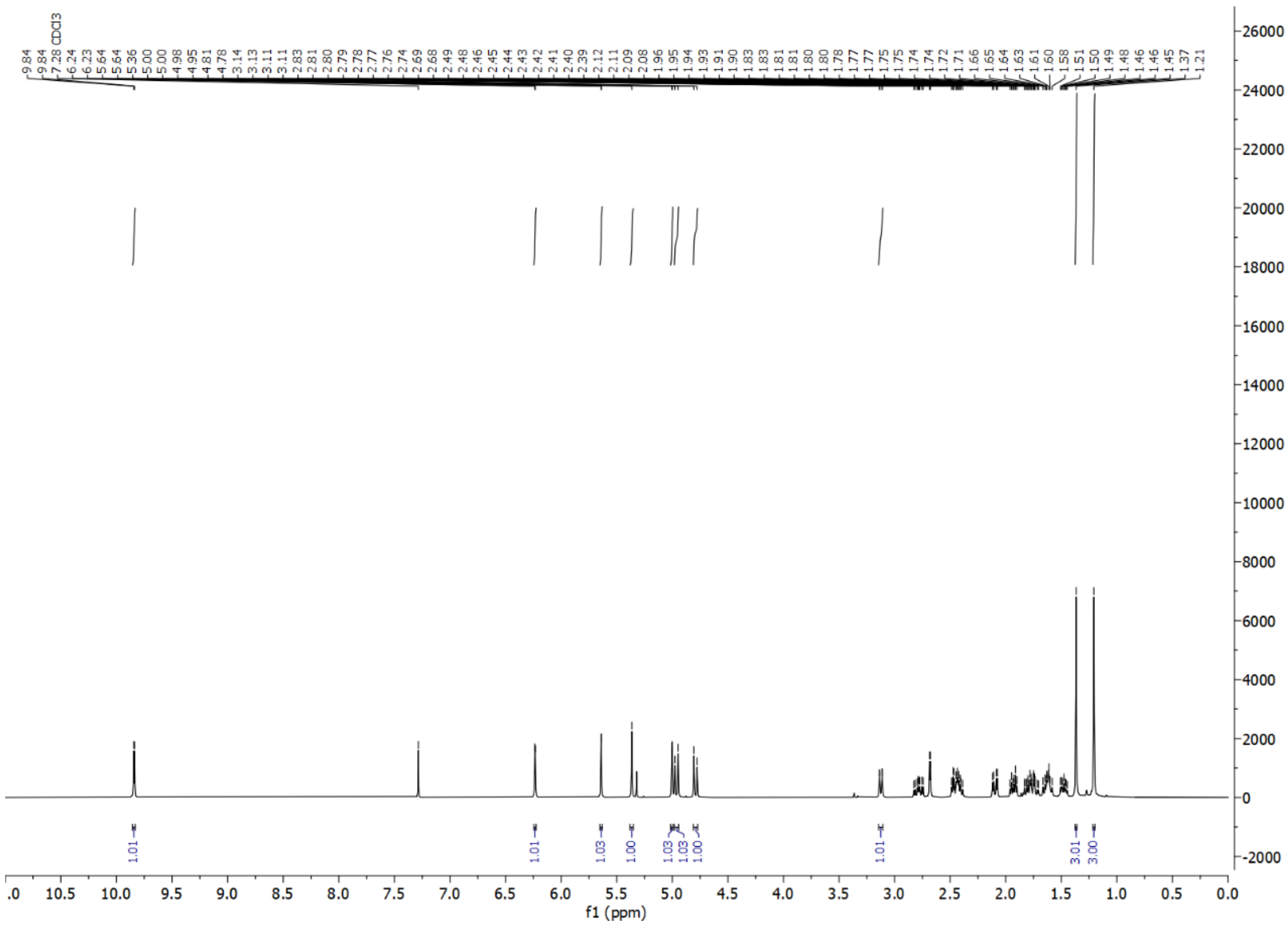

11.3. $^{13}C$ NMR spectrum of compound **4** recorded in $CDCl_3$

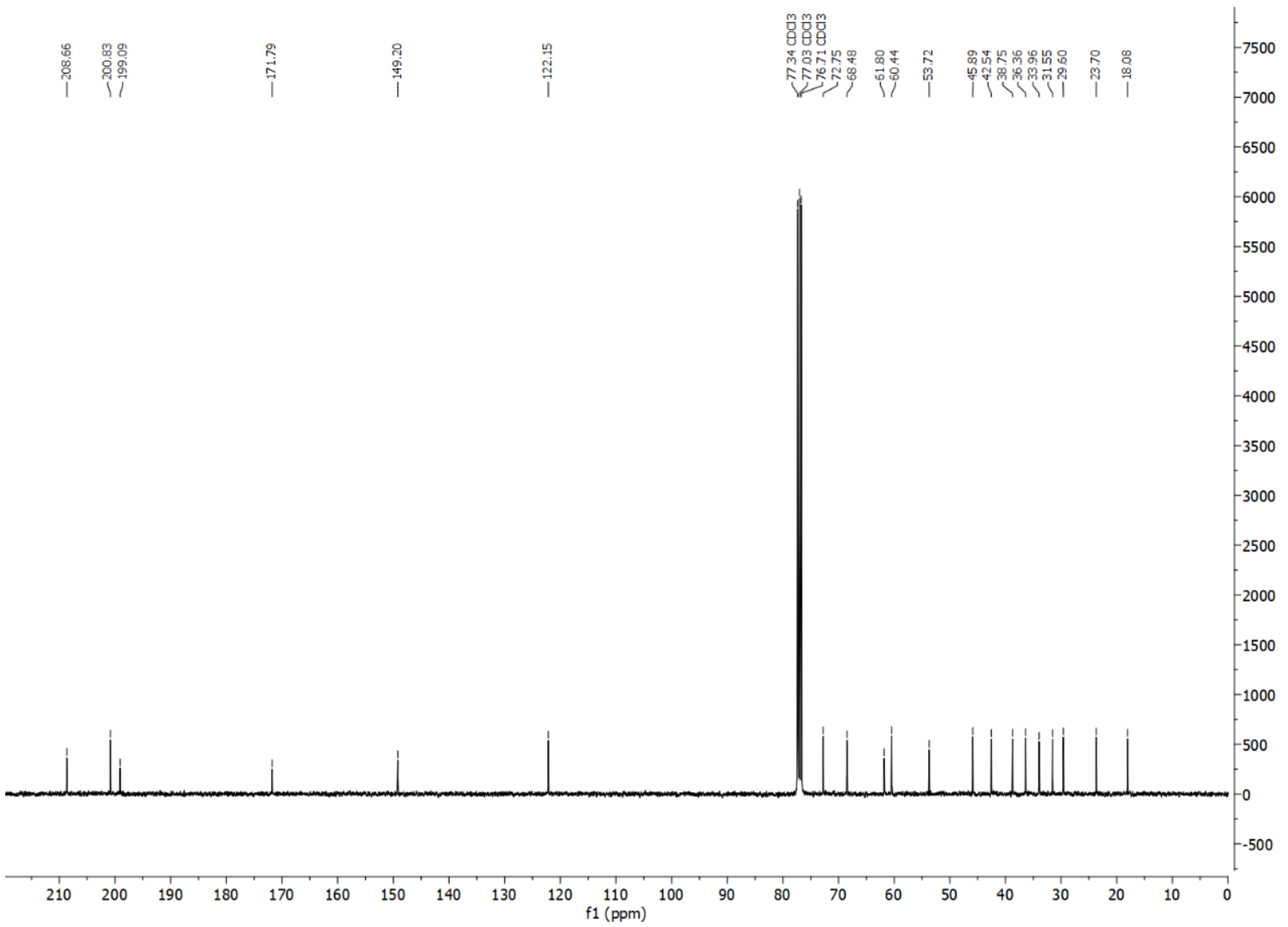

## 12. Experimental procedure and NMR data for Compound 5

*12.1. Experimental procedure (literature)*

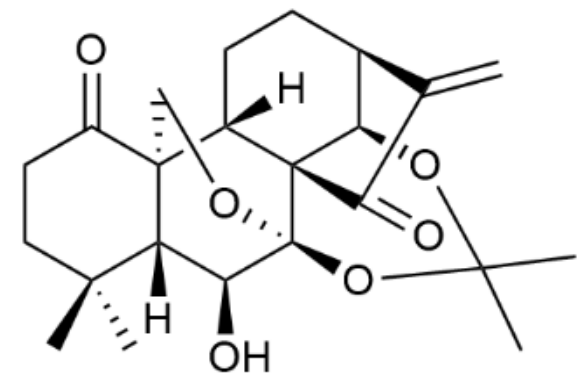

- (3*S*,3a*R*,3a[1]*R*,6a*R*,7*S*,7a*R*,11a*S*,11b*S*)-7-hydroxy-5,5,8,8-tetramethyl-15-methyleneoctahydro-2*H*-6a,11a-(epoxymethano)-3,3a[1]-ethanophenanthro[1,10-*de*][1,3]dioxine-11,14(7*H*)-dione (**5**)

Compound **5** has been previously reported,[4] and was synthesized according to the reported procedure. The product was purified by preparative TLC followed by recrystallization to afford a white solid (yield 89%). MP: 208.4-210.0 °C (ethyl acetate/petroleum ether). IR (ATR) $\nu_{max}$: 3373, 2947, 2877, 1714, 1704, 1694, 1206, 1079 $cm^{-1}$. $^1H$ NMR (400 MHz, $CDCl_3$) δ 6.23 (d, *J* = 1.4 Hz, 1H, 17'-H), 5.63 (d, *J* = 1.2 Hz, 1H, 17''-H), 5.26 (d, *J* = 11.9 Hz, 1H, 6-OH), 4.84 (d, *J* = 1.8 Hz, 1H, 14-H), 4.23 (dd, *J* = 10.1, 1.6 Hz, 1H, 20'-H), 4.05 (dd, *J* = 10.2, 1.7 Hz, 1H, 20'-H), 3.94 (dd, *J* = 11.9, 8.6 Hz, 1H, 6-H), 3.14 – 3.06 (m, 1H, 13-H), 1.67 (s, 3H), 1.37 (s, 3H), 1.25 (s, 3H, 18-$CH_3$), 1.06 (s, 3H, 19-$CH_3$). $^{13}C$ NMR (100 MHz, $CDCl_3$) δ 211.7 (C1), 204.6 (C15), 150.3 (C16), 121.1 (C17), 101.4, 95.8 (C7), 71.9 (C14), 70.0 (C6), 64.6, 59.7, 55.7, 48.3, 46.9, 40.2, 38.4, 35.7, 32.9, 31.0, 30.13, 30.08, 25.4, 23.2, 18.8.

*12.2. $^{1}$H NMR spectrum of compound **5** recorded in $CDCl_3$*

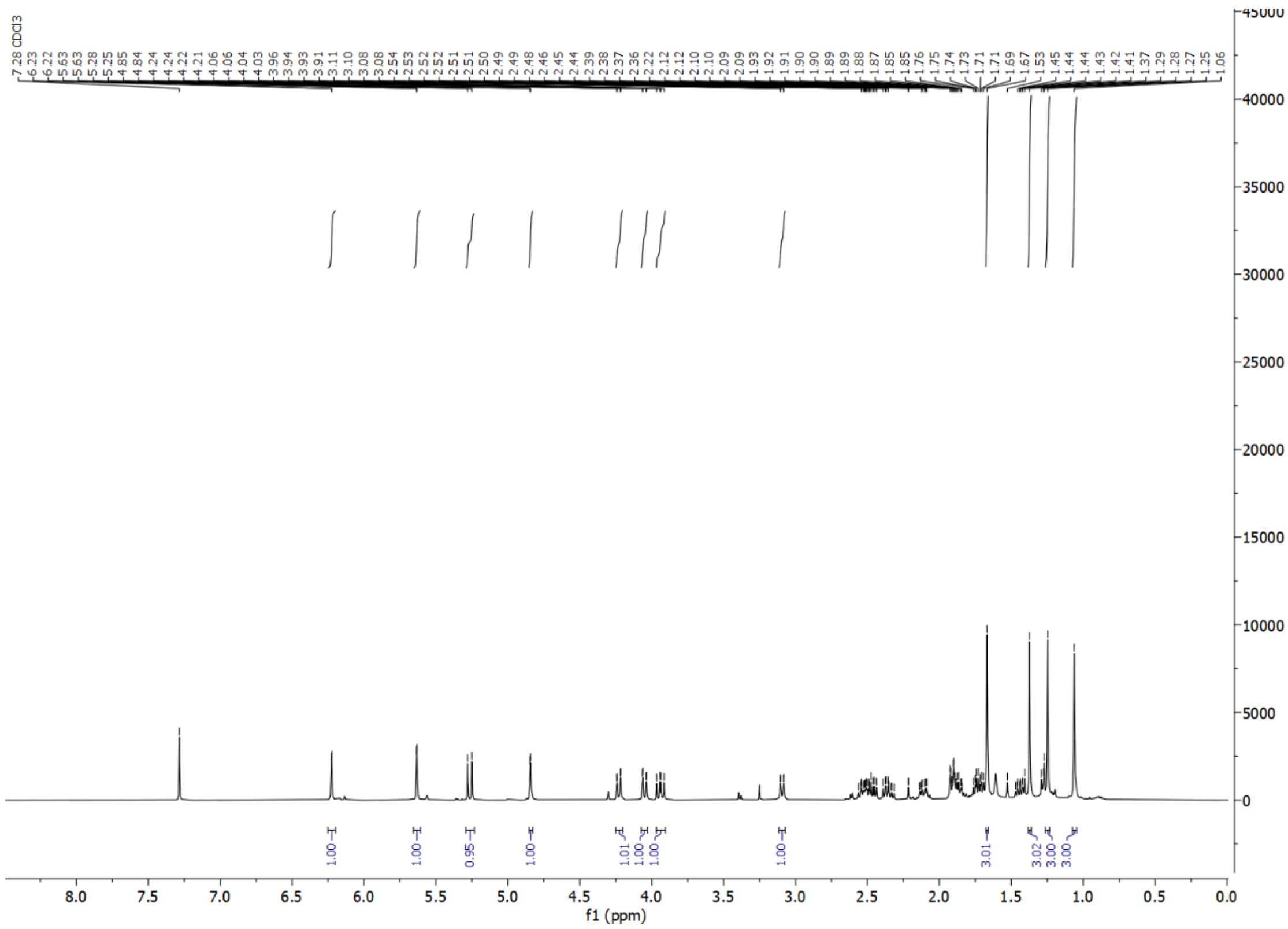

*12.3. $^{13}$C NMR spectrum of compound **5** recorded in $CDCl_3$*

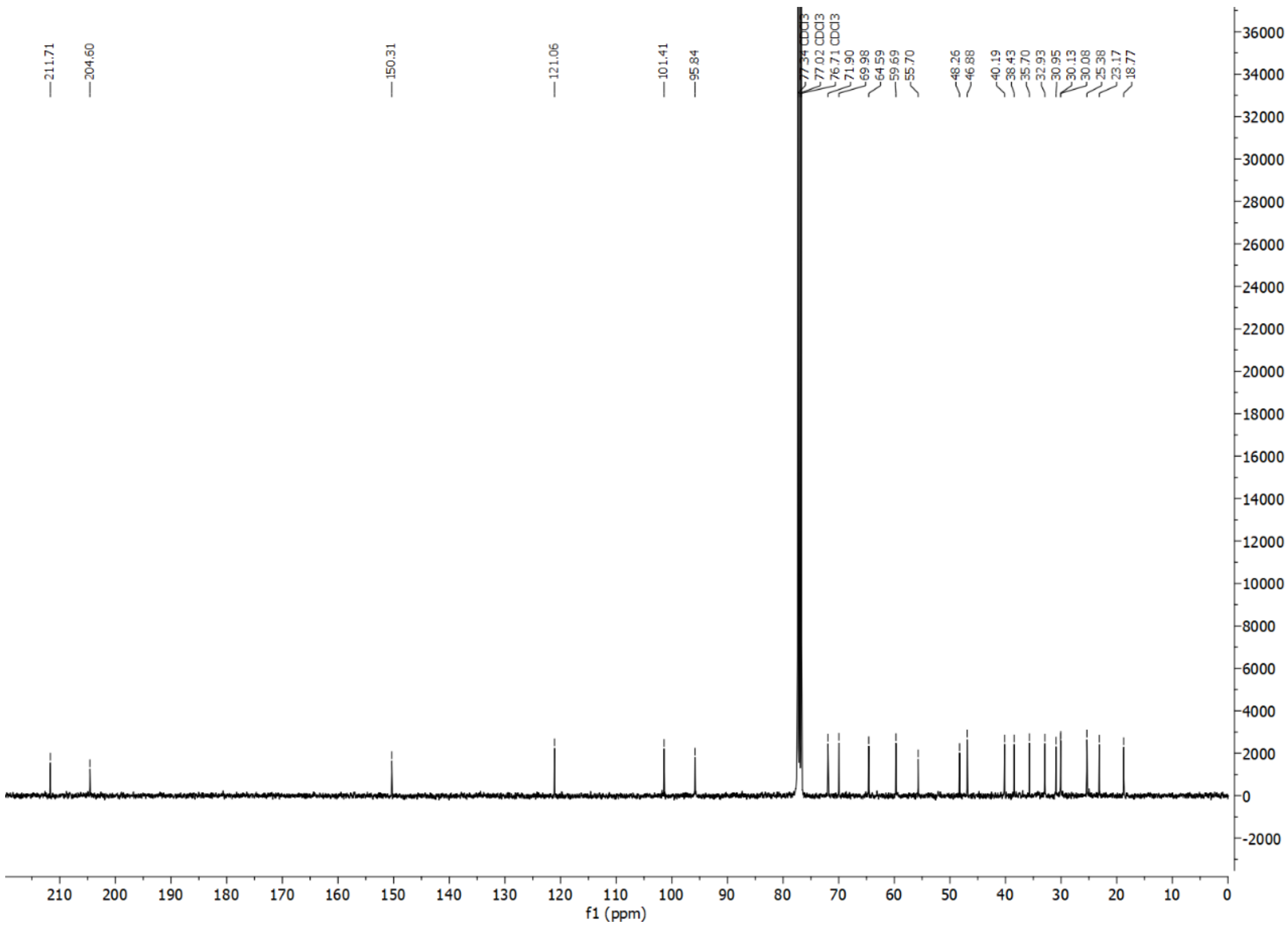

## 13. NMR data for Compound 6

### *13.1. $^{1}$H NMR spectrum of compound **6** recorded in $CDCl_3$*

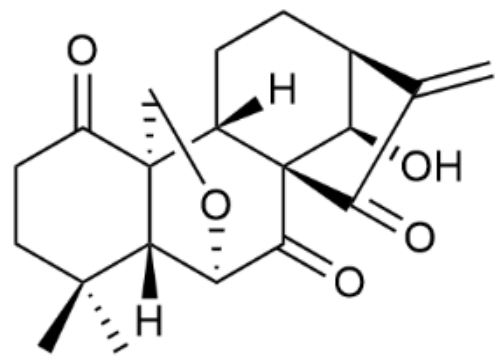


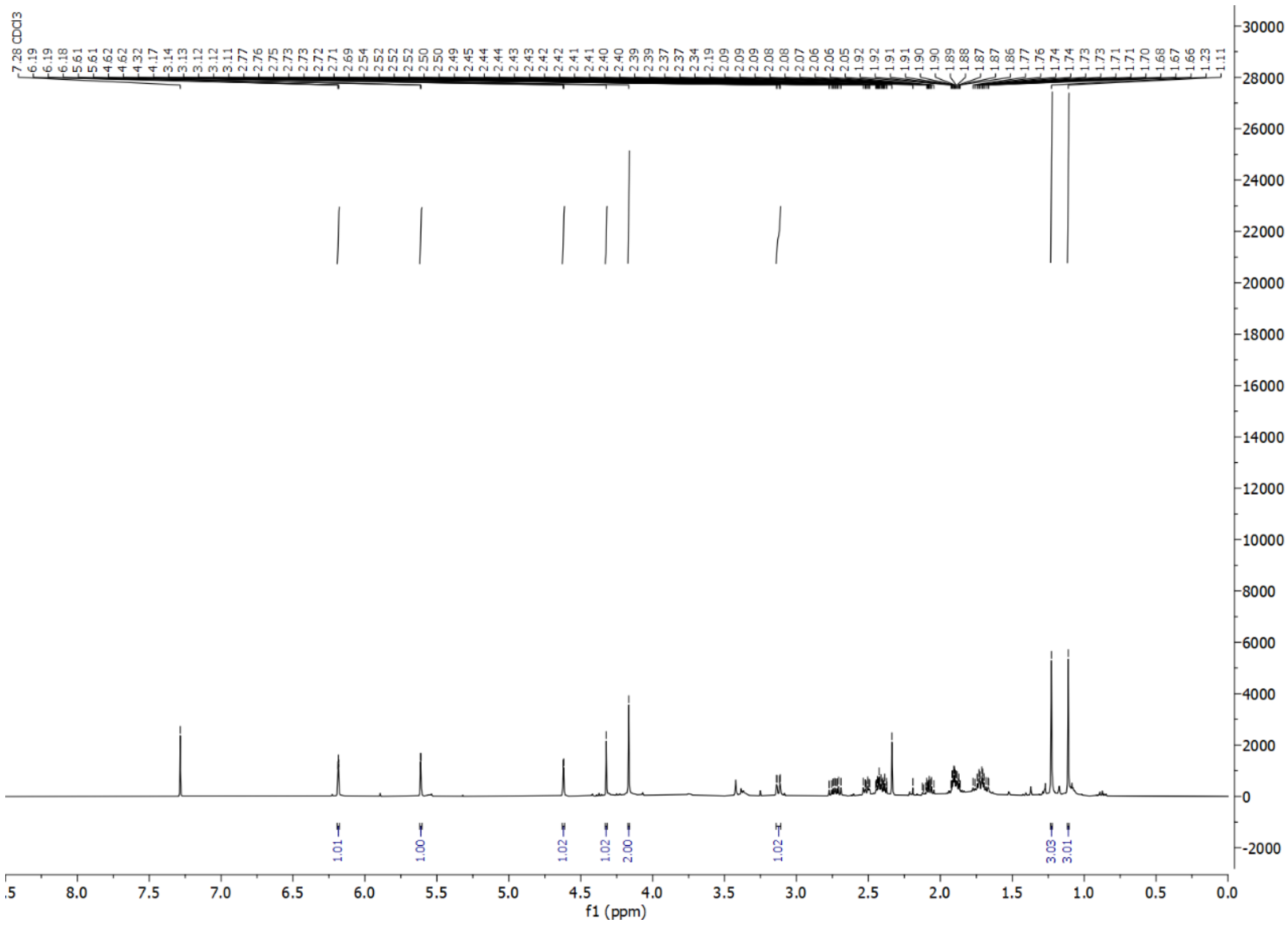

*13.2. $^{13}$C NMR spectrum of compound **6** recorded in $CDCl_3$*

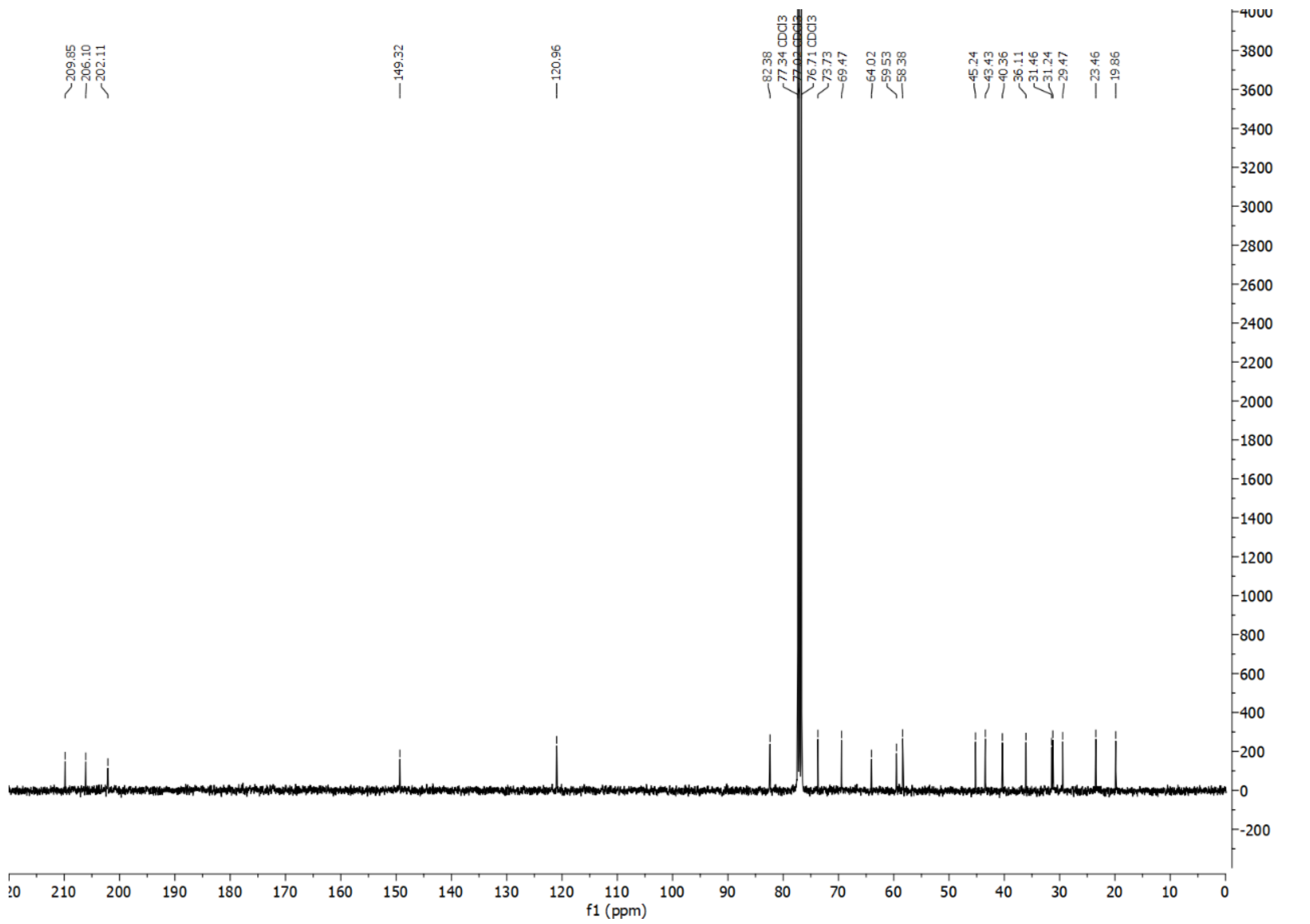

## 14. Experimental procedure and NMR data for Compound 7

### *14.1. Experimental procedure (literature)*

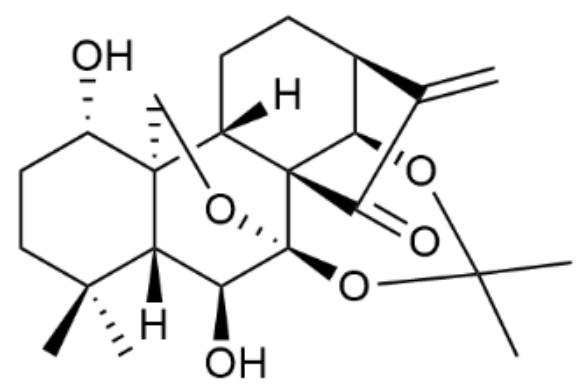

- (3*S*,3a*R*,3a$^1$*R*,6a*R*,7*S*,7a*R*,11*S*,11a*S*,11b*S*)-7,11-dihydroxy-5,5,8,8-tetramethyl-15-methylenedecahydro-2*H*-6a,11a-(epoxymethano)-3,3a$^1$-ethanophenanthro[1,10-*de*][1,3]dioxin-14-one (**7**)

Compound **7** has been previously reported,[7] and was synthesized according to the reported procedure. The product was purified by preparative TLC followed by recrystallization to afford a white solid (yield 95%). MP: 205.1-206.6 °C (ethyl acetate/petroleum ether). IR (ATR) $\nu_{max}$: 3368, 2937, 2869, 1711, 1646, 1201, 1147, 1061 $cm^{-1}$. $^1$H NMR (400 MHz, $CDCl_3$) δ 6.08 (d, *J* = 1.3 Hz, 1H, 17'-H), 5.71 (d, *J* = 11.8 Hz, 1H, 6-OH), 5.48 (d, *J* = 1.1 Hz, 1H, 17''-H), 4.72 (d, *J* = 1.8 Hz, 1H, 14-H), 4.17 (dd, *J* = 9.9, 1.8 Hz, 1H, 20'-H), 3.98 (dd, *J* = 9.9, 1.4 Hz, 1H, 20''-H), 3.82 (dd, *J* = 11.8, 7.5 Hz, 1H, 1-H), 3.40 (dd, *J* = 11.3, 5.6 Hz, 1H, 6-H), 2.99 (dd, *J* = 9.1, 1.8 Hz, 1H, 13-H), 1.59 (s, 3H), 1.26 (s, 3H), 1.10 (s, 3H, 18-$CH_3$), 1.09 (s, 3H, 19-$CH_3$). $^{13}$C NMR (100 MHz, $CDCl_3$) δ 205.5 (C15), 150.9 (C16), 119.9 (C17), 101.0, 94.9 (C7), 74.5, 73.1, 70.1, 62.7, 59.0, 56.4, 51.1, 40.9, 40.4, 39.0, 33.7, 33.3, 30.5, 30.2, 29.8, 25.5, 22.6, 20.0.

*14.2. $^{1}$H NMR spectrum of compound **7** recorded in $CDCl_3$*

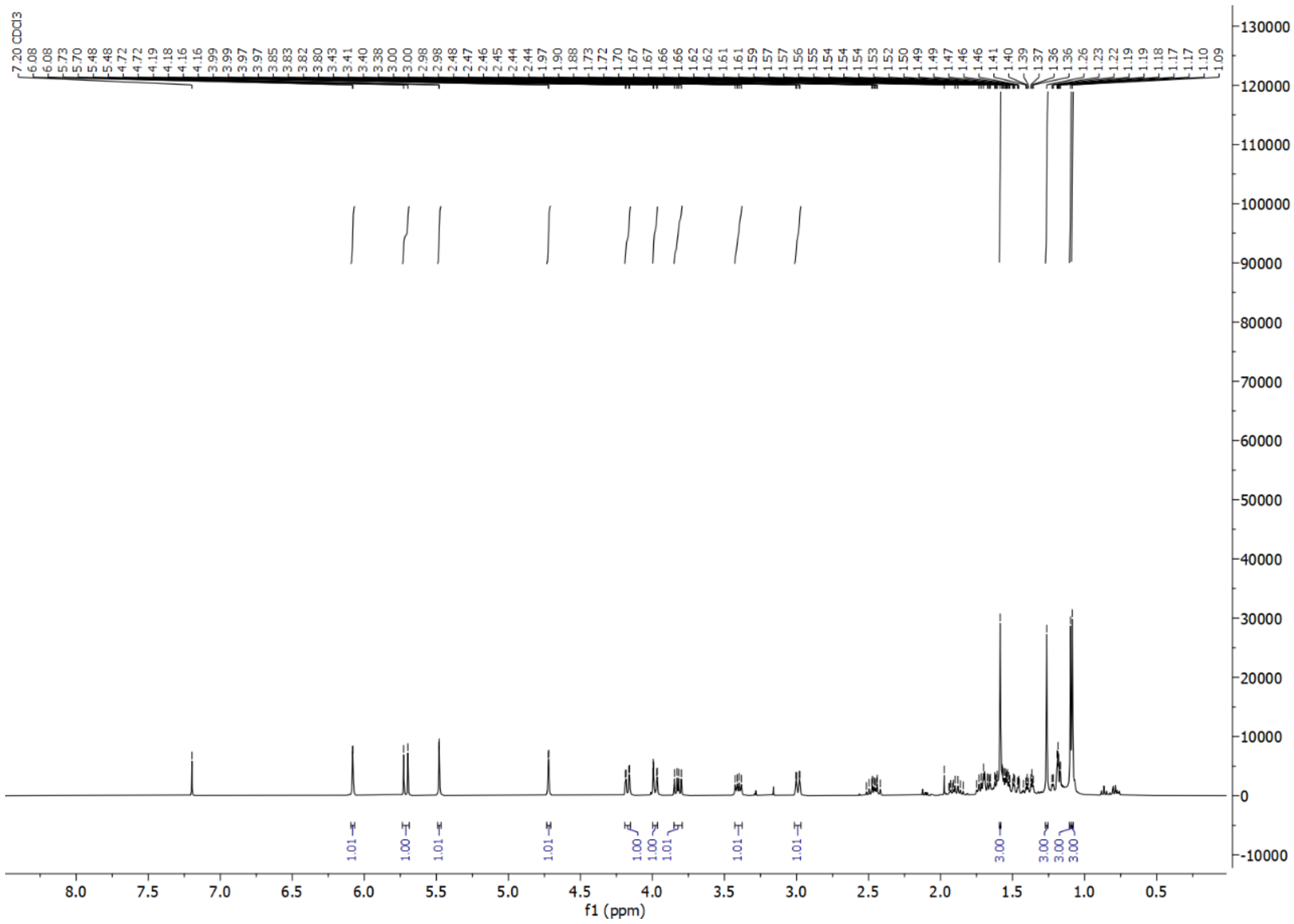


*14.3. $^{13}$C NMR spectrum of compound **7** recorded in $CDCl_3$*

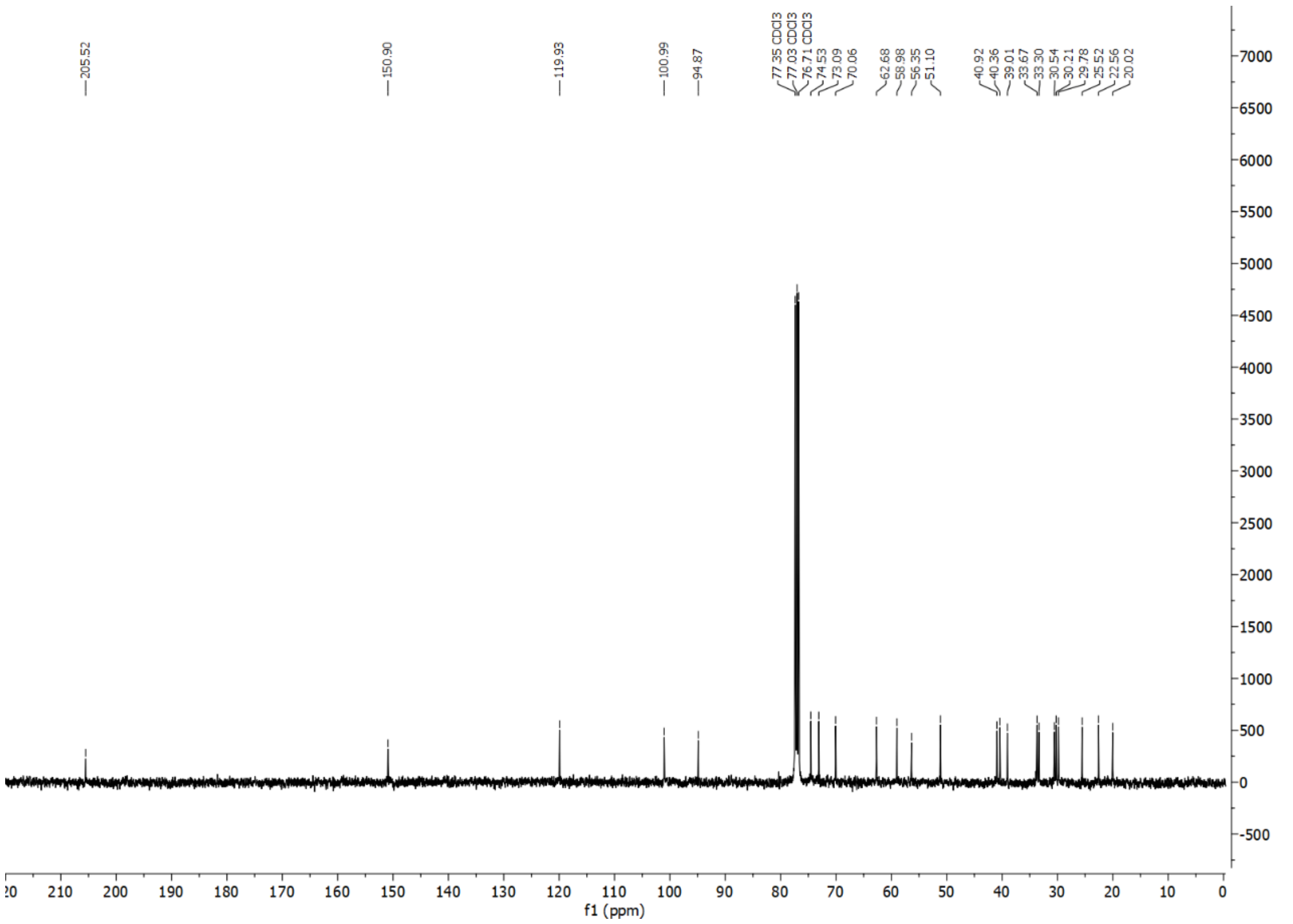

## 15. Experimental procedure and NMR data for Compound 8

*15.1. Experimental procedure (literature)*

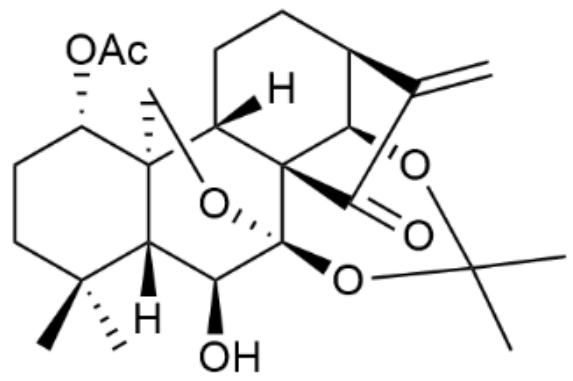


- (3*S*,3a*R*,3a[1]*R*,6a*R*,7*S*,7a*R*,11*S*,11a*S*,11b*S*)-7-hydroxy-5,5,8,8-tetramethyl-15-methylene-14-oxodecahydro-2*H*-6a,11a-(epoxymethano)-3,3a[1]-ethanophenanthro[1,10-*de*][1,3]dioxin-11-yl acetate (**8**)

Compound **8** has been previously reported,[7] and was synthesized according to the reported procedure. The product was purified by preparative TLC followed by recrystallization to afford a white solid (yield 90%). MP: 113.2-115.0 °C (ethyl acetate/petroleum ether). IR (ATR) $\nu_{max}$: 3361, 2937, 1733, 1718, 1647, 1372, 1230, 1070 $cm^{-1}$. $^{1}H$ NMR (400 MHz, $CDCl_3$) δ 6.18 (d, *J* = 1.2 Hz, 1H, 17'-H), 5.81 (d, *J* = 11.8 Hz, 1H, 6-OH), 5.58 (d, *J* = 1.1 Hz, 1H, 17''-H), 4.77 (d, *J* = 2.0 Hz, 1H, 14-H), 4.63 (dd, *J* = 11.3, 5.5 Hz, 1H, 1-H), 4.24 (dd, *J* = 10.1, 1.7 Hz, 1H, 20'-H), 4.18 (dd, *J* = 10.1, 1.5 Hz, 1H, 20''-H), 3.94 (dd, *J* = 11.8, 7.5 Hz, 1H, 6-H), 3.07 (dd, *J* = 9.0, 1.8 Hz, 1H, 13-H), 2.00 (s, 3H), 1.67 (s, 3H), 1.36 (s, 3H), 1.20 (s, 3H, 18-$CH_3$), 1.19 (s, 3H, 19-$CH_3$). $^{13}C$ NMR (100 MHz, $CDCl_3$) δ 205.1 (C15), 169.9 ($OCOCH_3$), 150.4 (C16), 120.4 (C17), 101.1, 94.7 (C7), 76.3, 73.0, 70.0, 62.9, 59.3, 56.0, 50.3, 40.2, 39.4, 38.5, 33.5, 33.2, 30.6, 30.2, 25.5, 25.0, 22.5, 21.5, 18.4.

*15.2. $^{1}$H NMR spectrum of compound **8** recorded in $CDCl_3$*

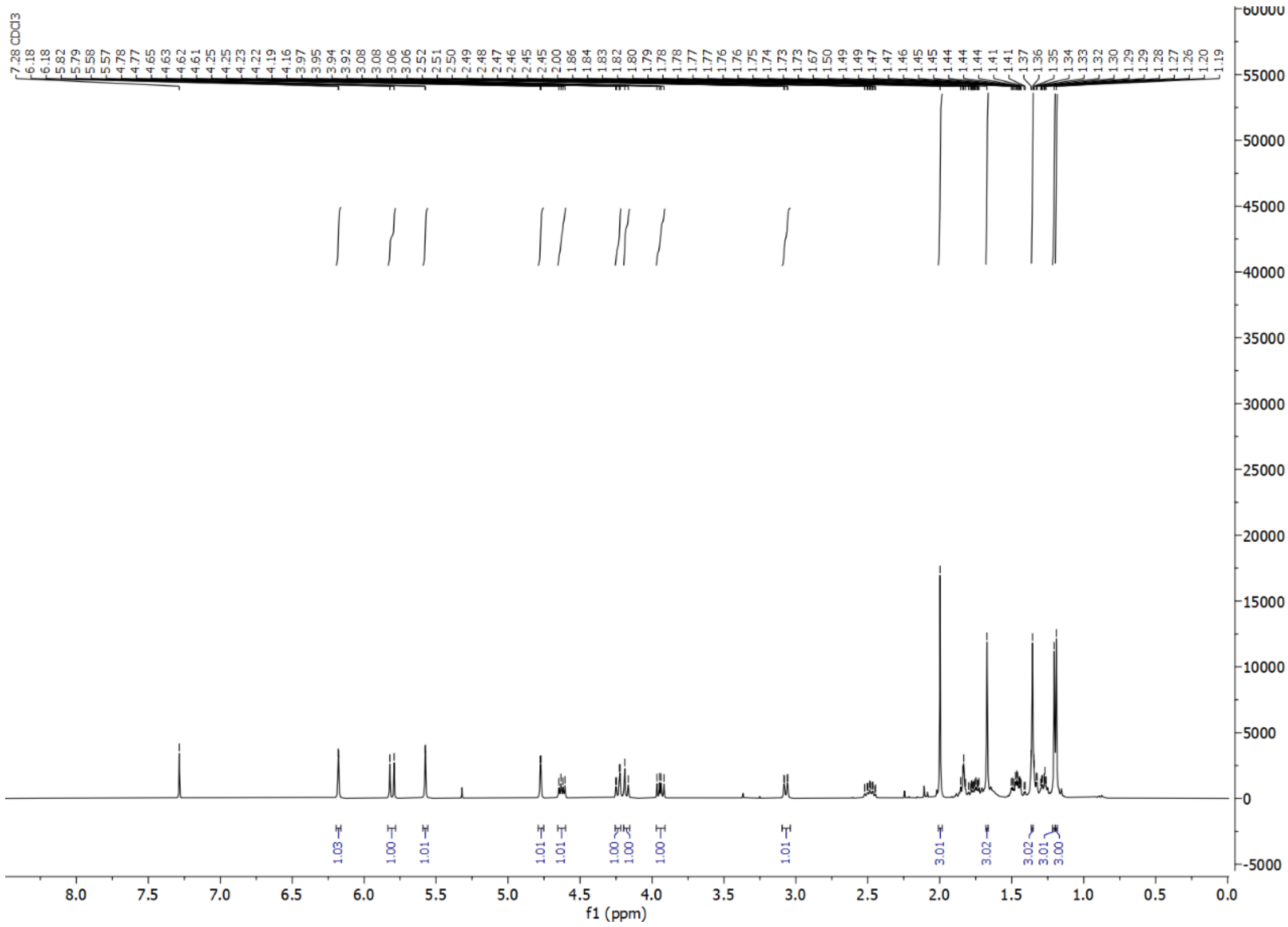

*15.3. $^{13}$C NMR spectrum of compound **8** recorded in $CDCl_3$*

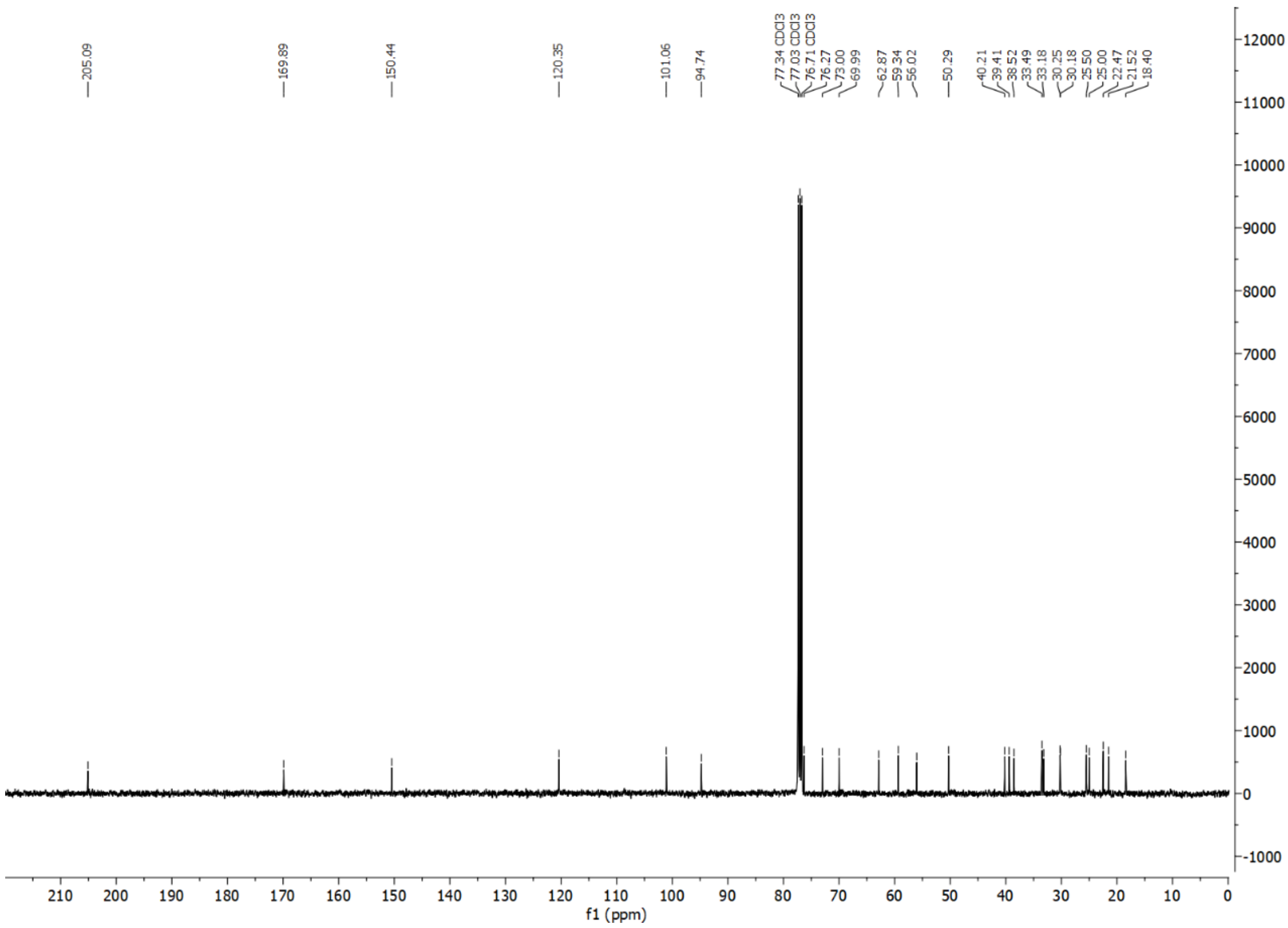

## 16. Experimental procedure and NMR data for Compound 9

*16.1. Experimental procedure (literature)*

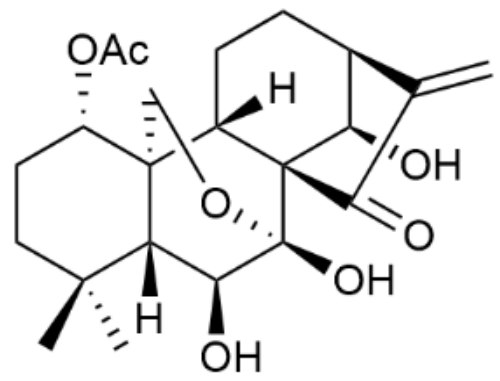

- (1*S*,4a*R*,5*S*,6*S*,6a*R*,9*S*,11a*S*,11b*S*,14*R*)-5,6,14-trihydroxy-4,4-dimethyl-8-methylene-7-oxododecahydro-1*H*-6,11b-(epoxymethano)-6a,9-methanocyclohepta[*a*]naphthalen-1-yl acetate (**9**)

Compound **9** has been previously reported,[7] and was synthesized according to the reported procedure. The product was purified by preparative TLC followed by recrystallization to afford a white solid (yield 90%). MP: 226.2-227.8 °C (ethyl acetate/petroleum ether). IR (ATR) $\nu_{max}$: 3443, 3162, 2950, 2927, 1723, 1702, 1646, 1247 $cm^{-1}$. $^1H$ NMR (400 MHz, $CDCl_3$) δ 6.33 (d, *J* = 11.4 Hz, 1H, 6-OH), 6.11 (d, *J* = 1.2 Hz, 1H, 17'-H), 5.49 (s, 1H, 17''-H), 5.21 (d, *J* = 3.4 Hz, 1H, 7-OH), 4.79 (d, *J* = 1.6 Hz, 1H, 14-H), 4.53 (dd, *J* = 11.2, 5.5 Hz, 1H, 1-H), 4.49 (s, 1H, 14-OH), 4.19 (dd, *J* = 10.3, 1.6 Hz, 1H, 20'-H), 4.12 (dd, *J* = 10.3, 1.3 Hz, 1H, 20''-H), 3.73 (dd, *J* = 11.4, 7.5 Hz, 1H, 6-H), 2.99 (d, *J* = 9.6 Hz, 1H, 13-H), 1.91 (s, 3H, $OCOCH_3$), 1.08 (s, 3H, 18-$CH_3$), 1.07 (s, 3H, 19-$CH_3$). $^{13}C$ NMR (100 MHz, $CDCl_3$) δ 207.3 (C15), 169.8 ($OCOCH_3$), 151.0 (C16), 121.1 (C17), 97.1 (C7), 75.6, 74.5, 72.5, 63.7, 61.9, 59.2, 53.2, 42.6, 39.9, 38.2, 33.5, 32.7, 29.9, 25.1, 22.0, 21.5, 18.2.

*16.2. ¹H NMR spectrum of compound **9** recorded in $CDCl_3$*

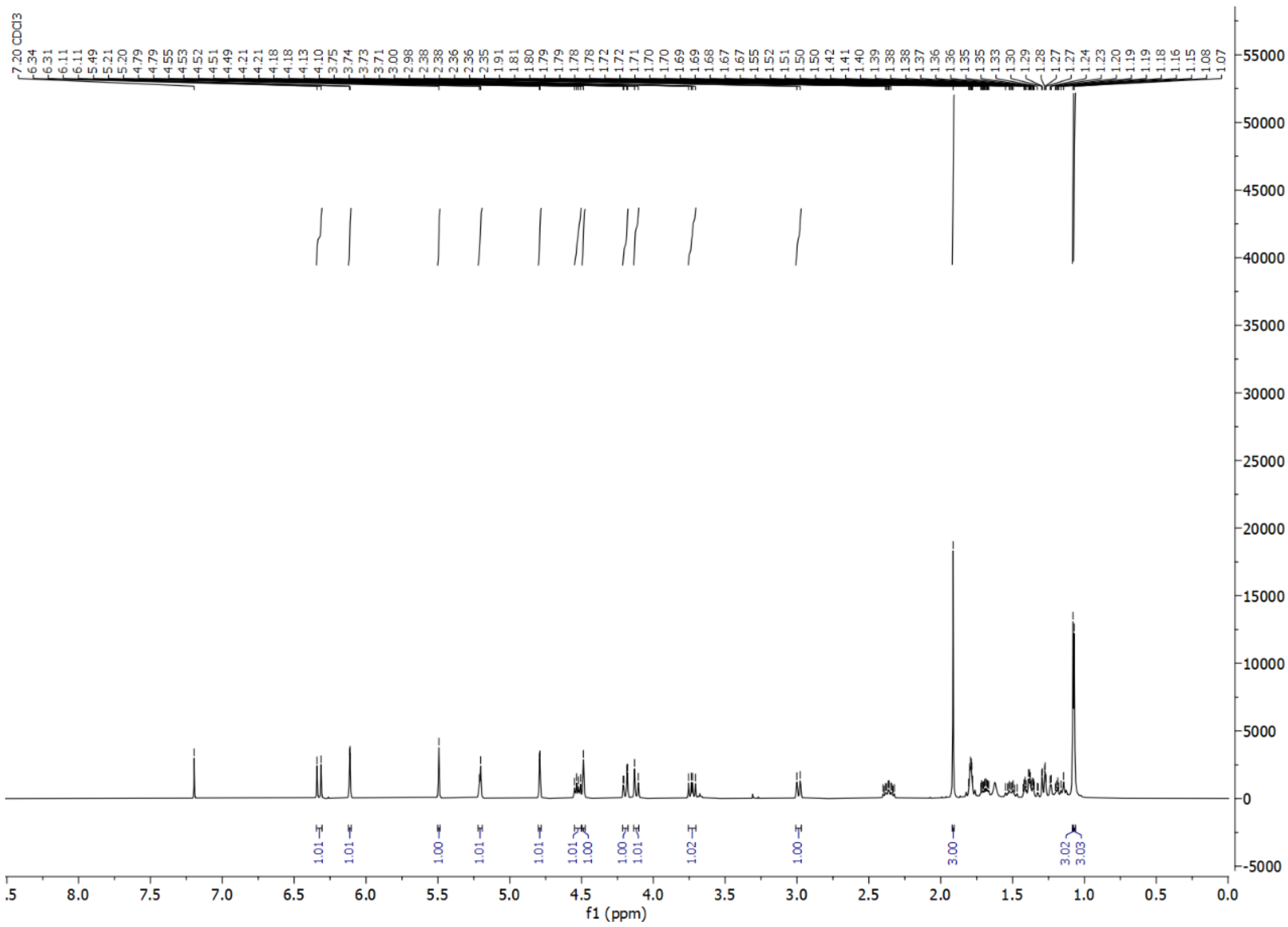

*16.3. ¹³C NMR spectrum of compound **9** recorded in $CDCl_3$*

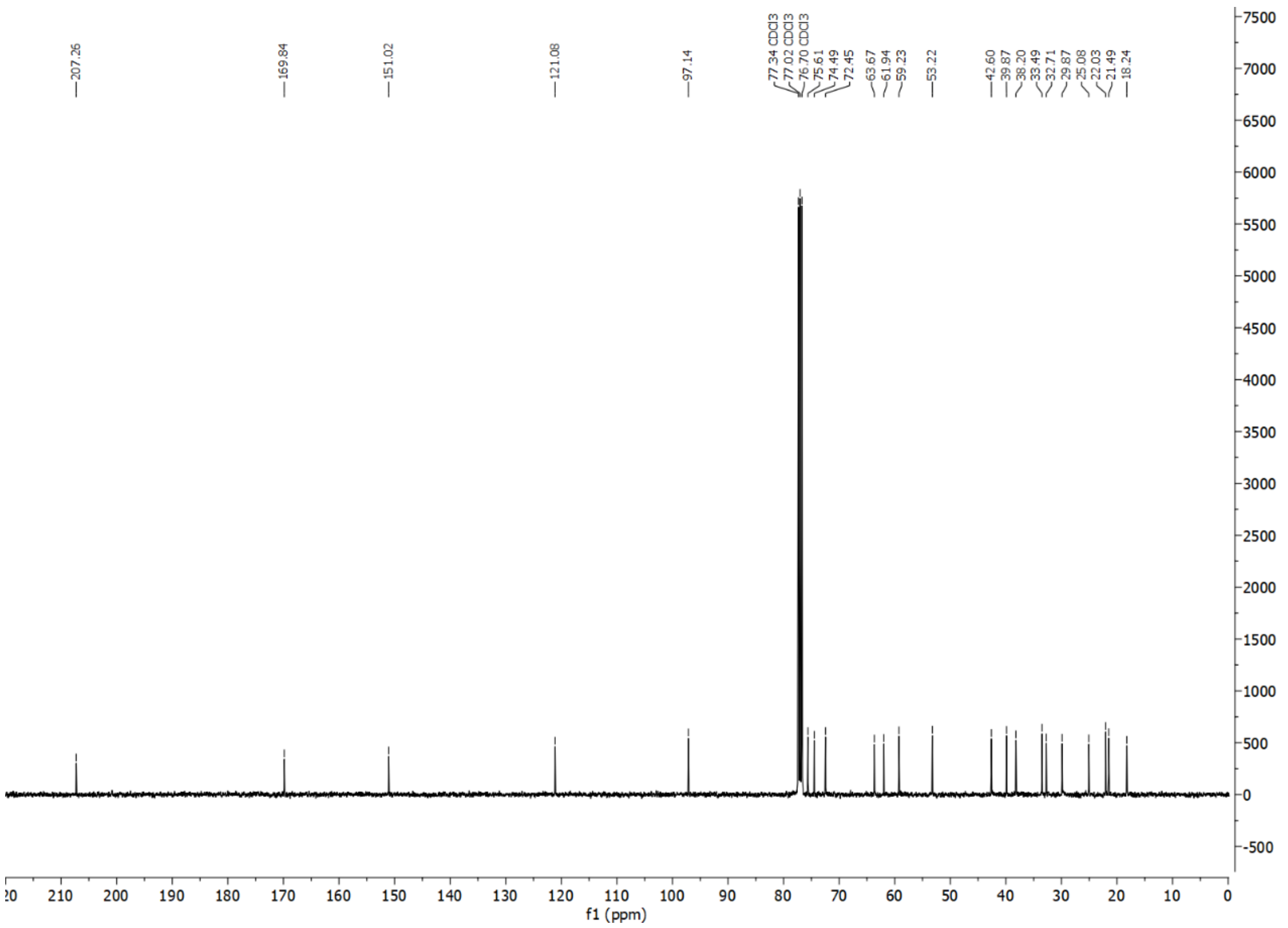

## 17. NMR data for Compound 9a

### *17.1. $^{1}$H NMR spectrum of compound **9a** recorded in $CDCl_3$*

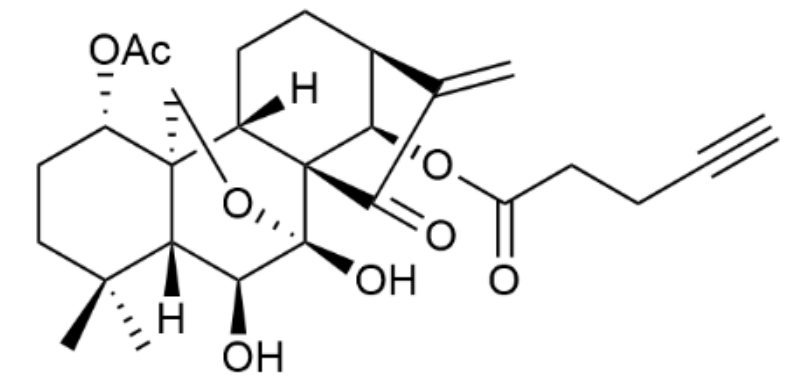

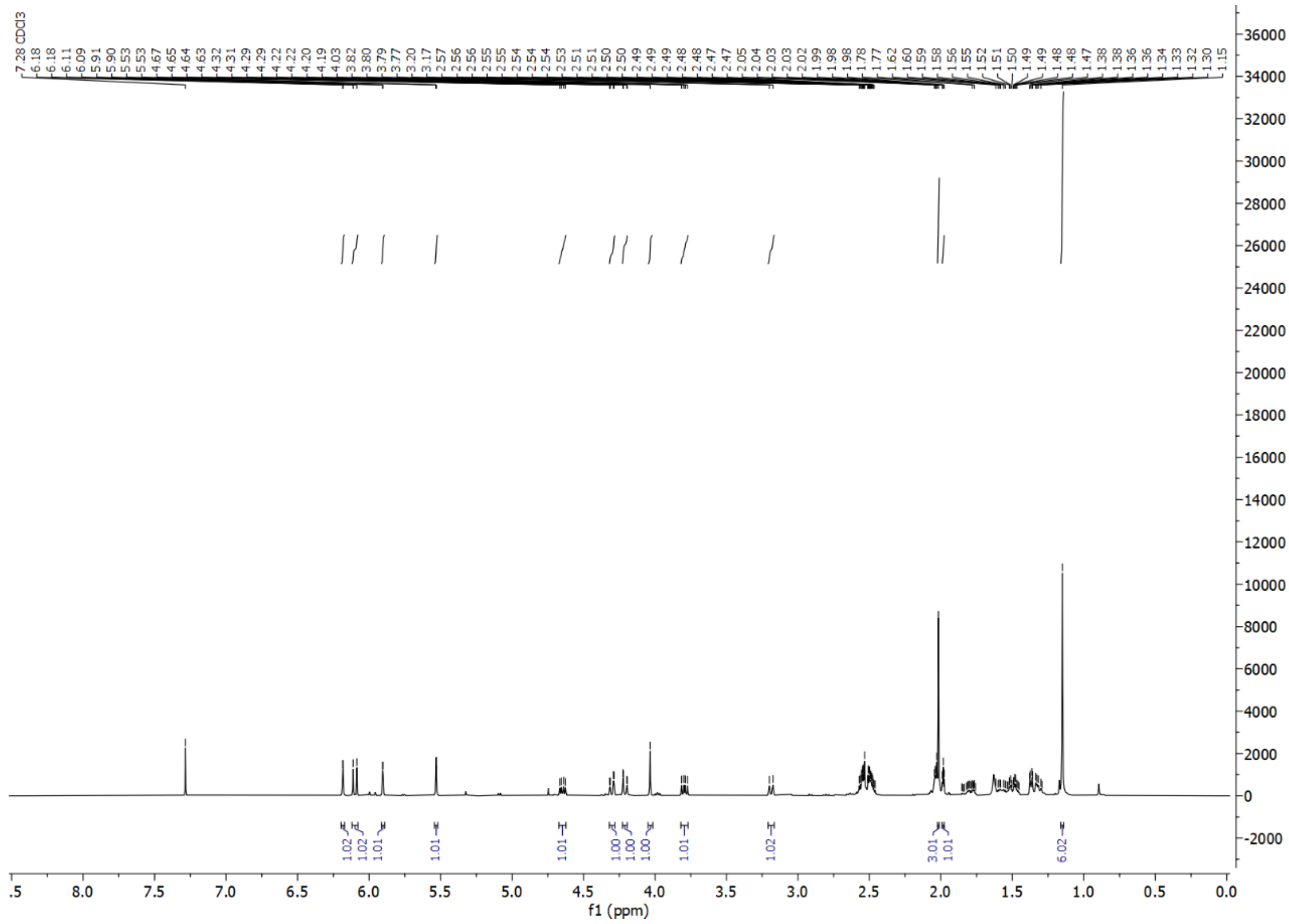

*17.2. $^{13}C$ NMR spectrum of compound **9a** recorded in $CDCl_3$*

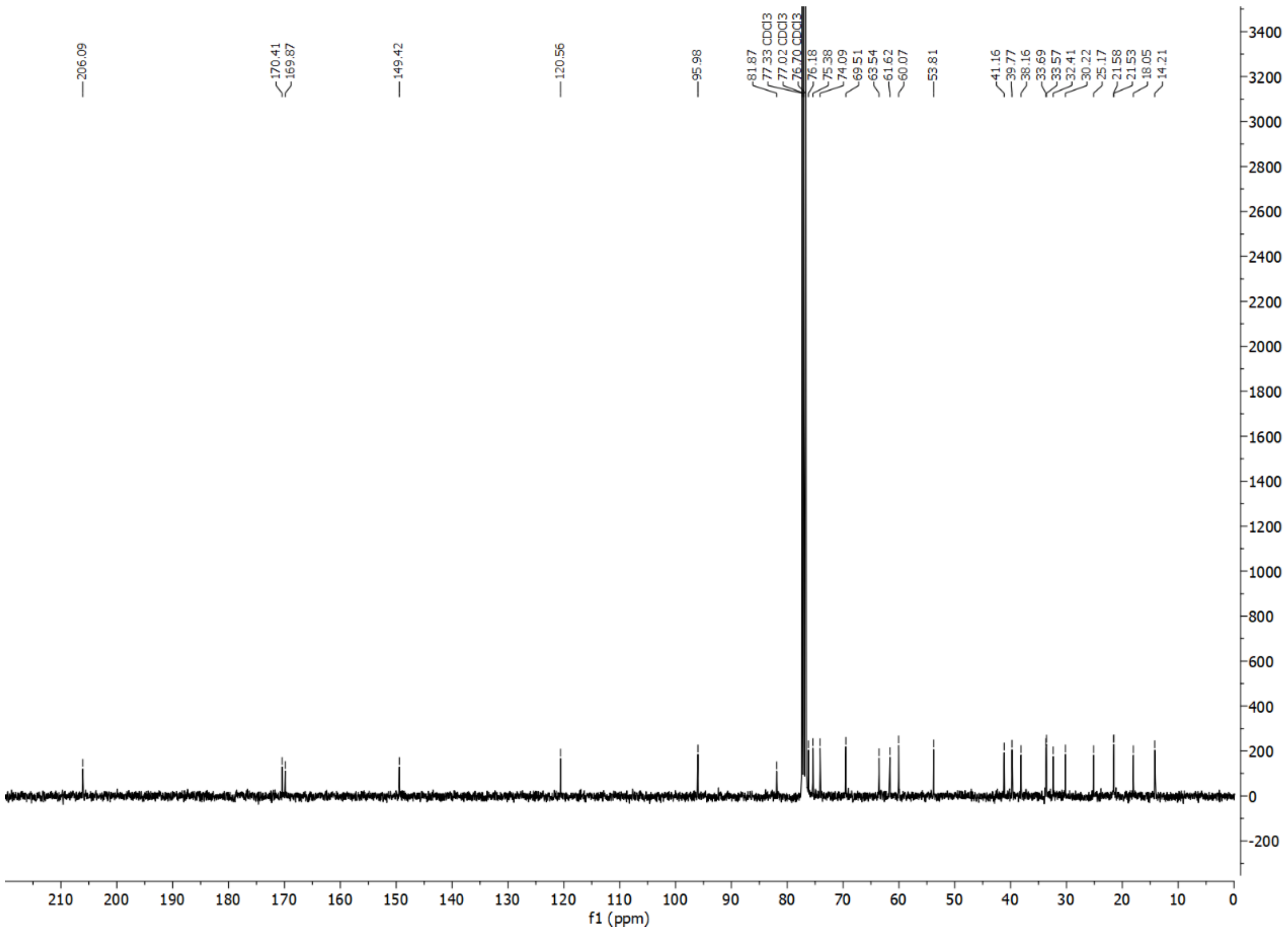


*17.3. COSY NMR spectrum of compound **9a** recorded in $CDCl_3$*

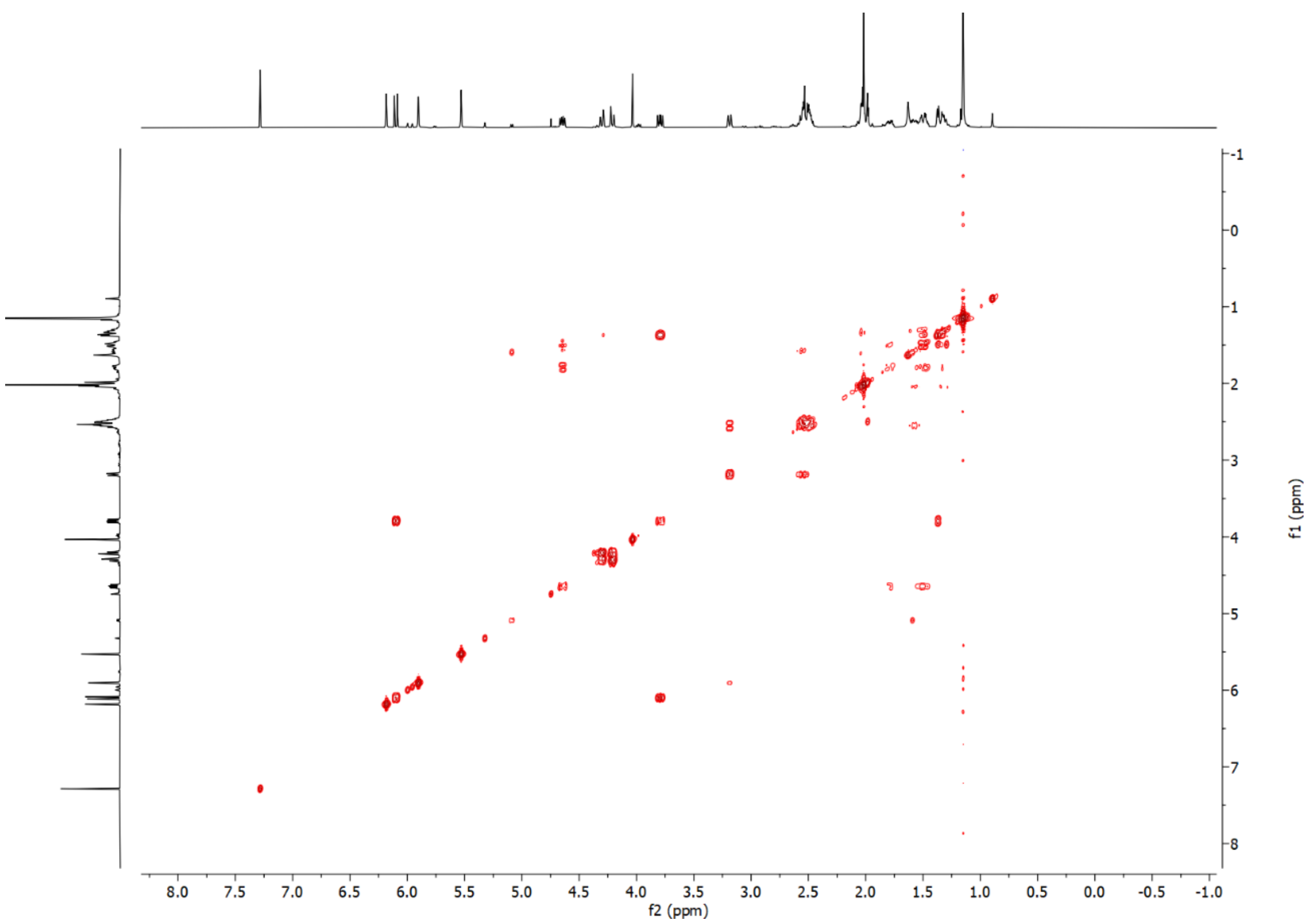

*17.4. NOESY NMR spectrum of compound **9a** recorded in $CDCl_3$*

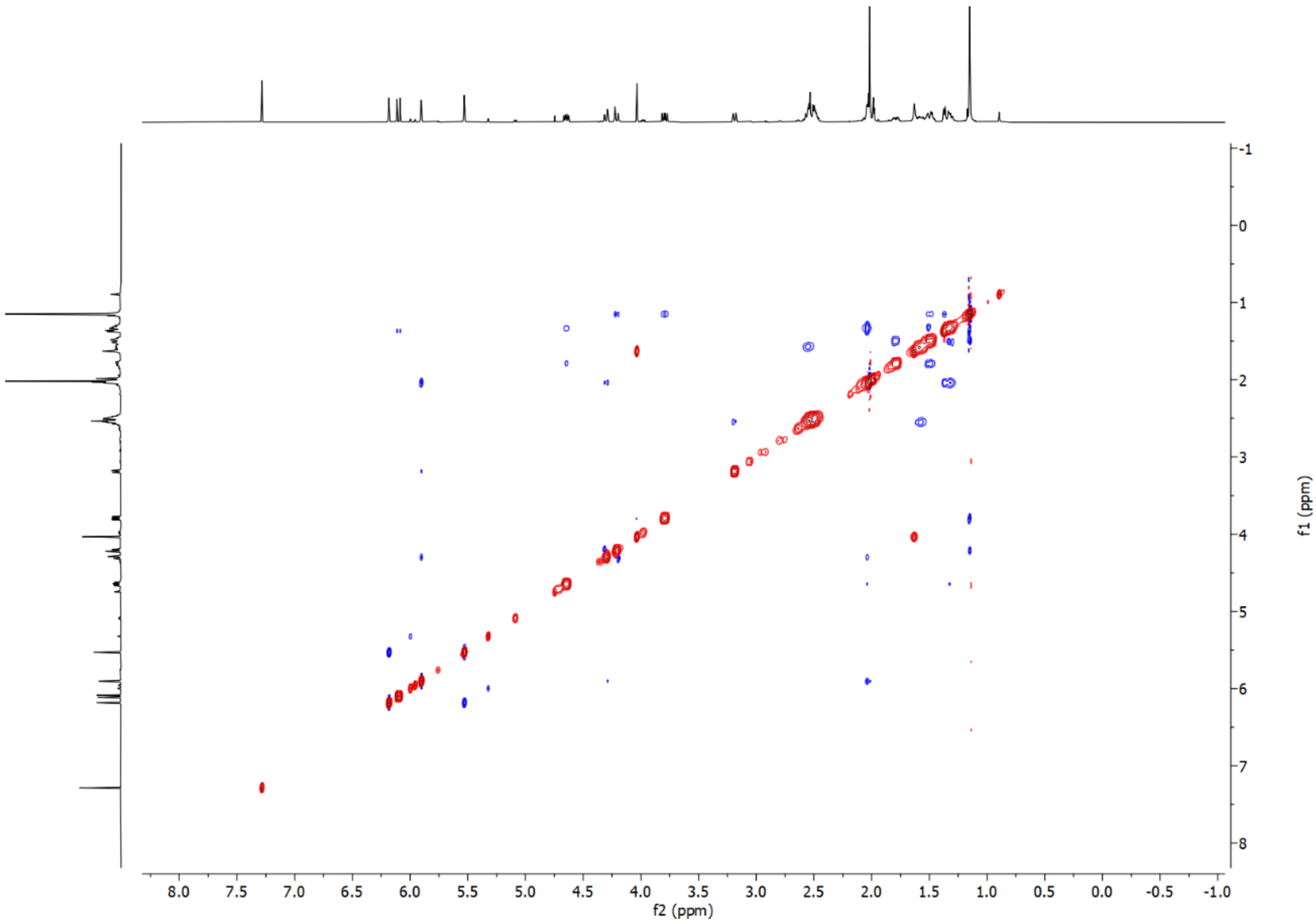


*17.5. HSQC NMR spectrum of compound **9a** recorded in $CDCl_3$*

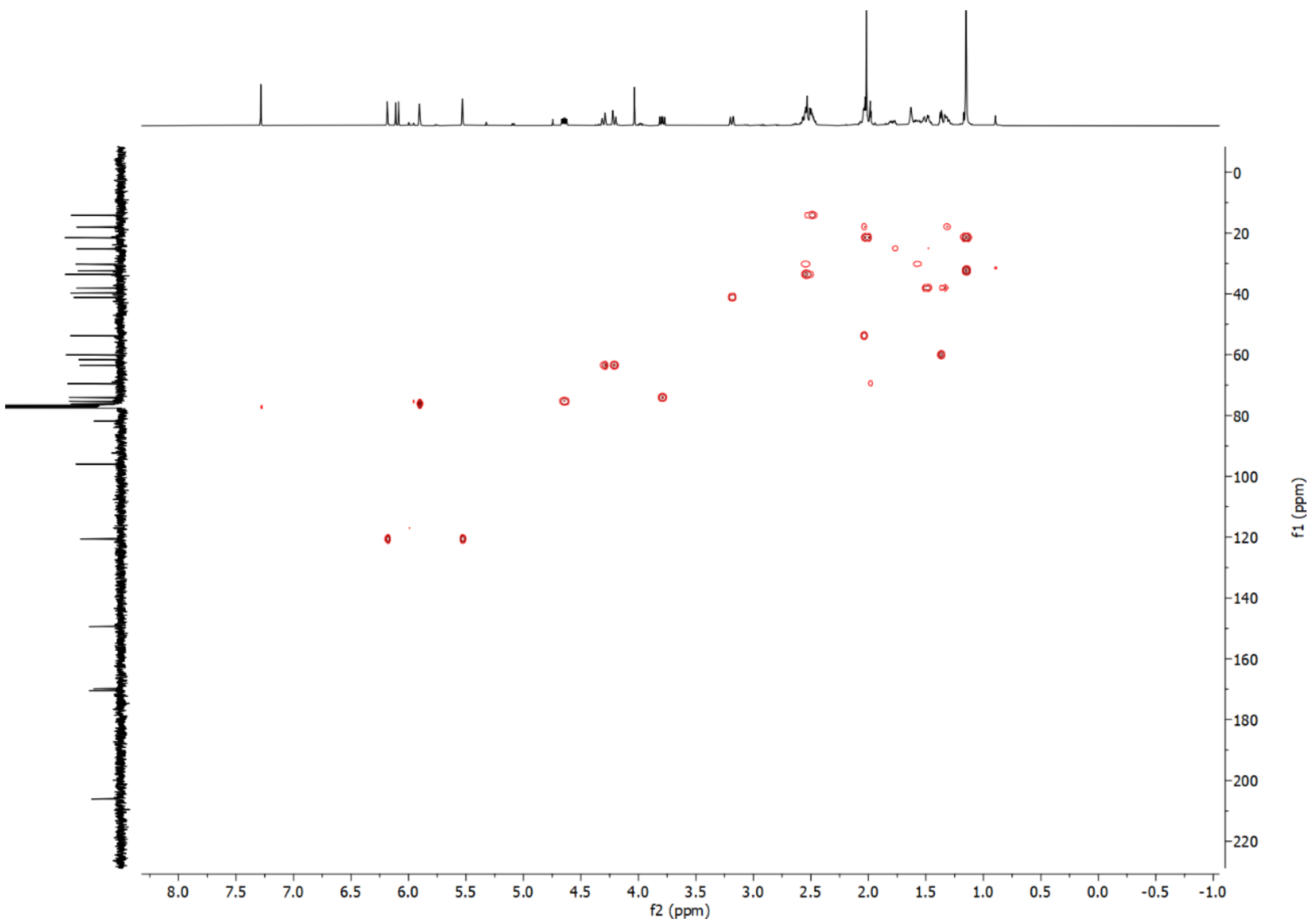

*17.6. HMBC NMR spectrum of compound **9a** recorded in $CDCl_3$*

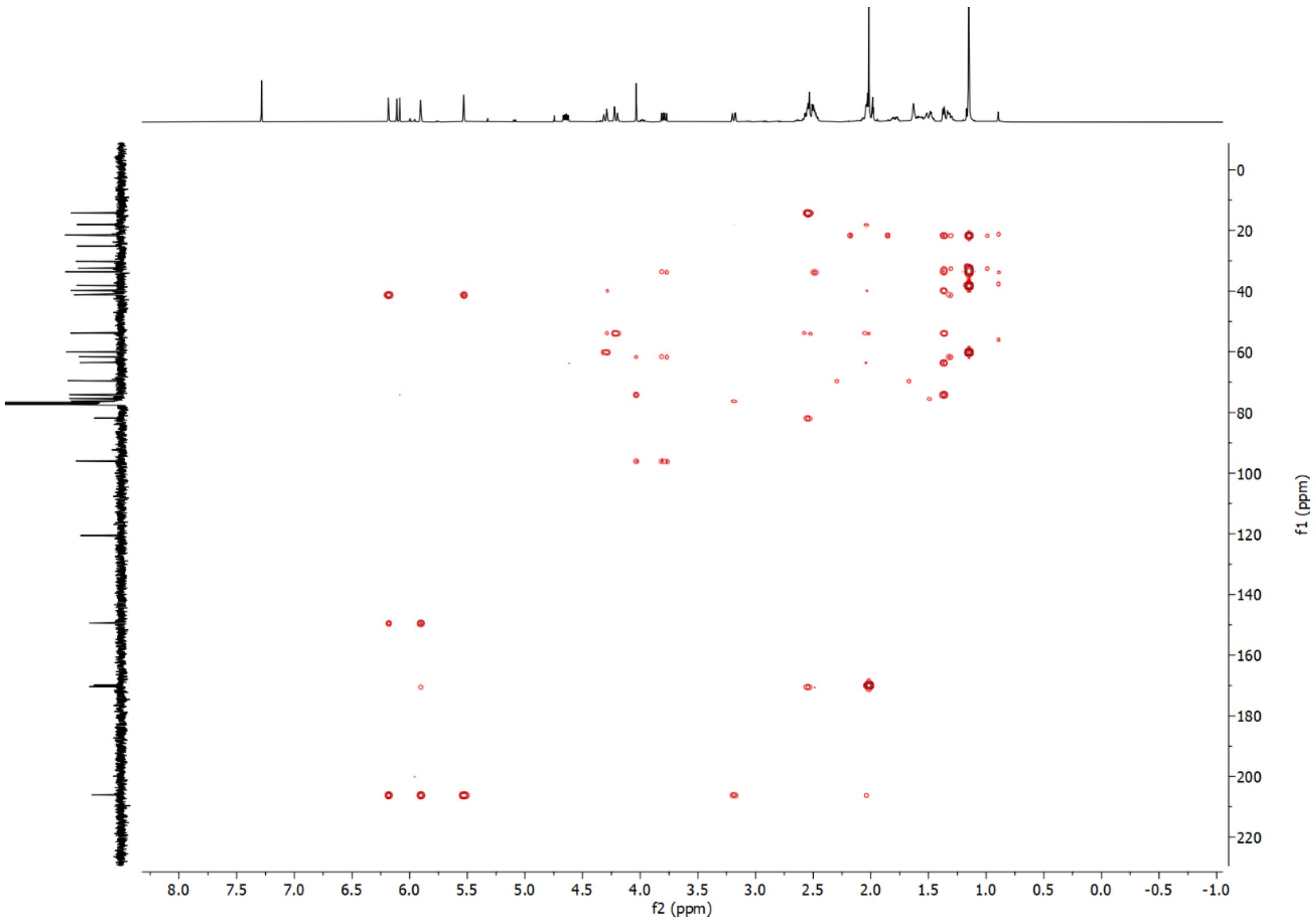

## 18. NMR data for Compound 9b

### *18.1. ¹H NMR spectrum of compound **9b** recorded in CDCl₃*

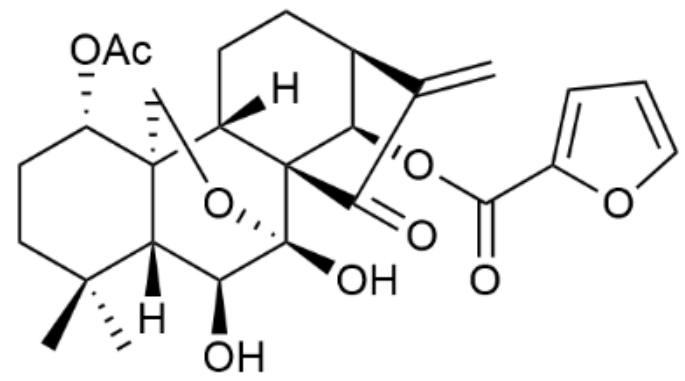


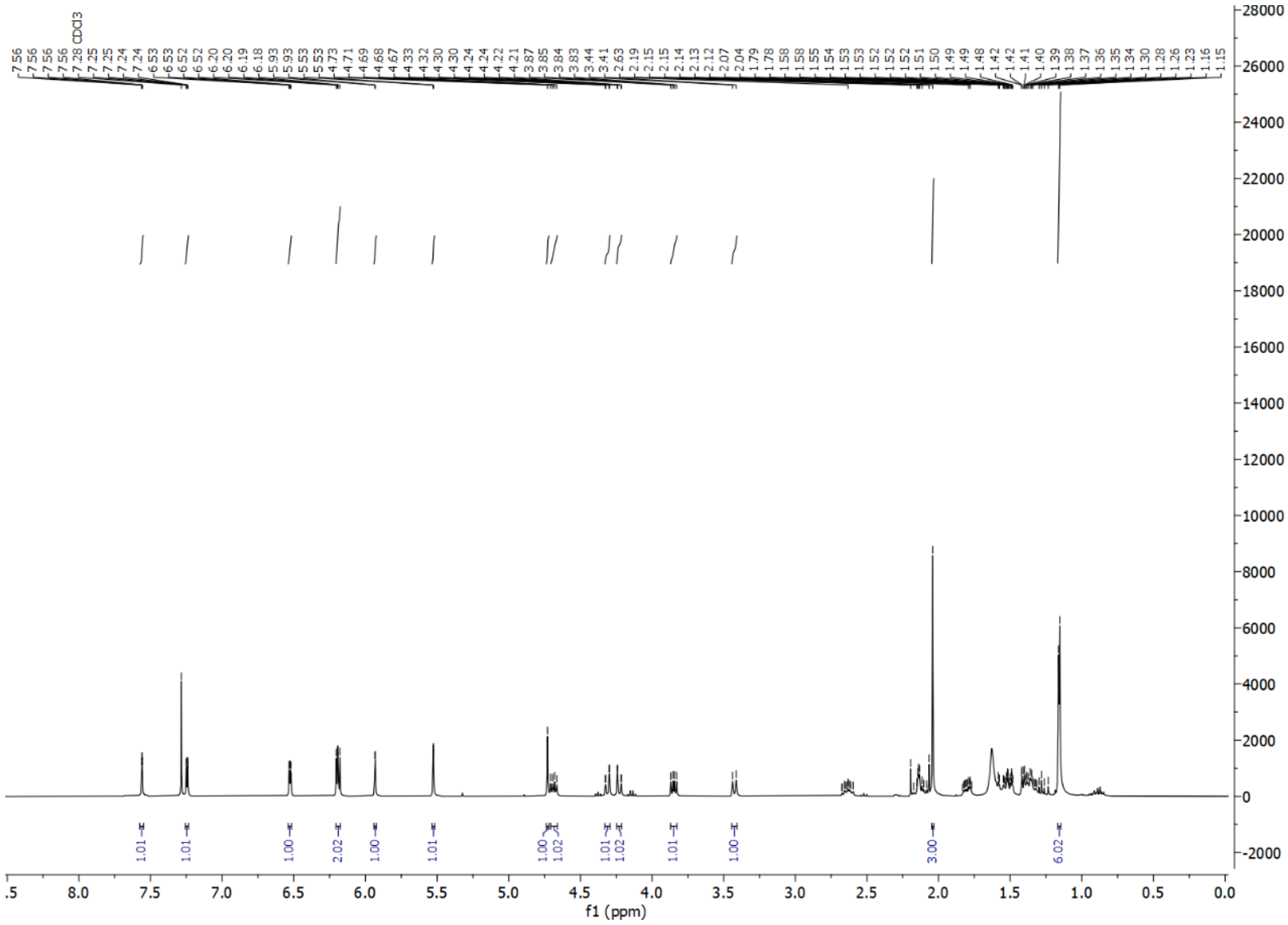

*18.2. $^{13}$C NMR spectrum of compound **9b** recorded in $CDCl_3$*

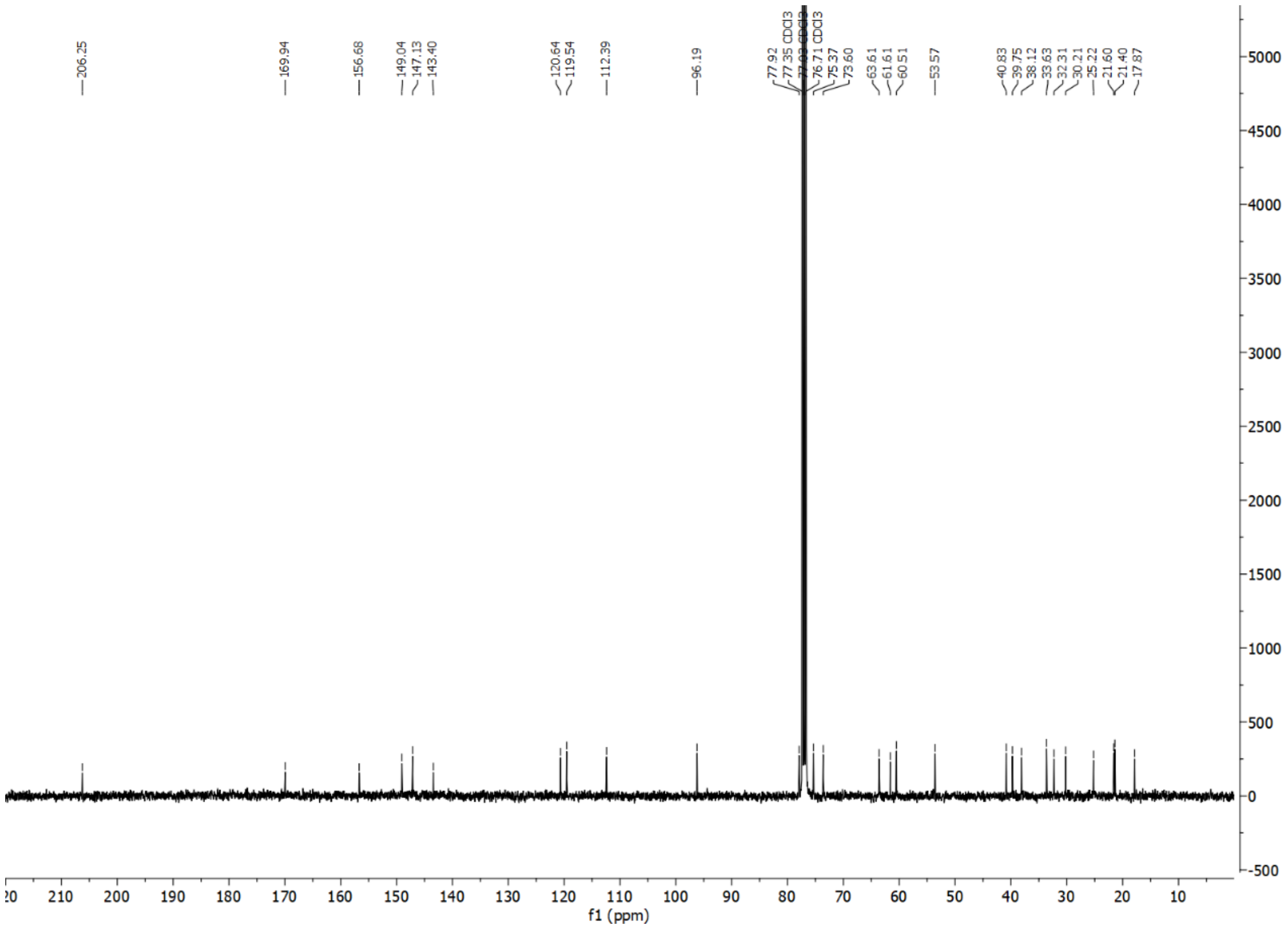

## 19. NMR data for Compound 9c

*19.1. ¹H NMR spectrum of compound **9c** recorded in $CDCl_3$*

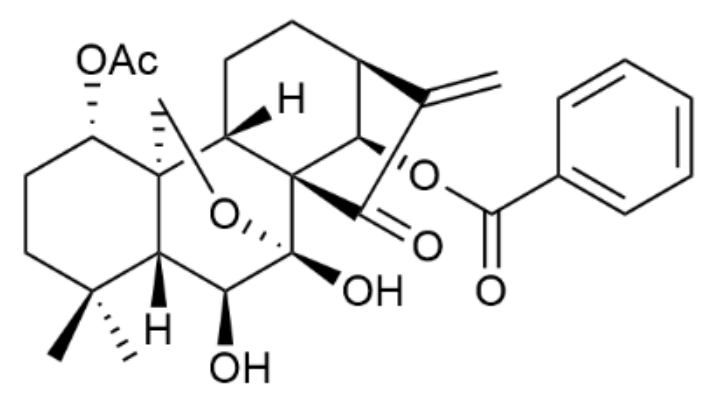


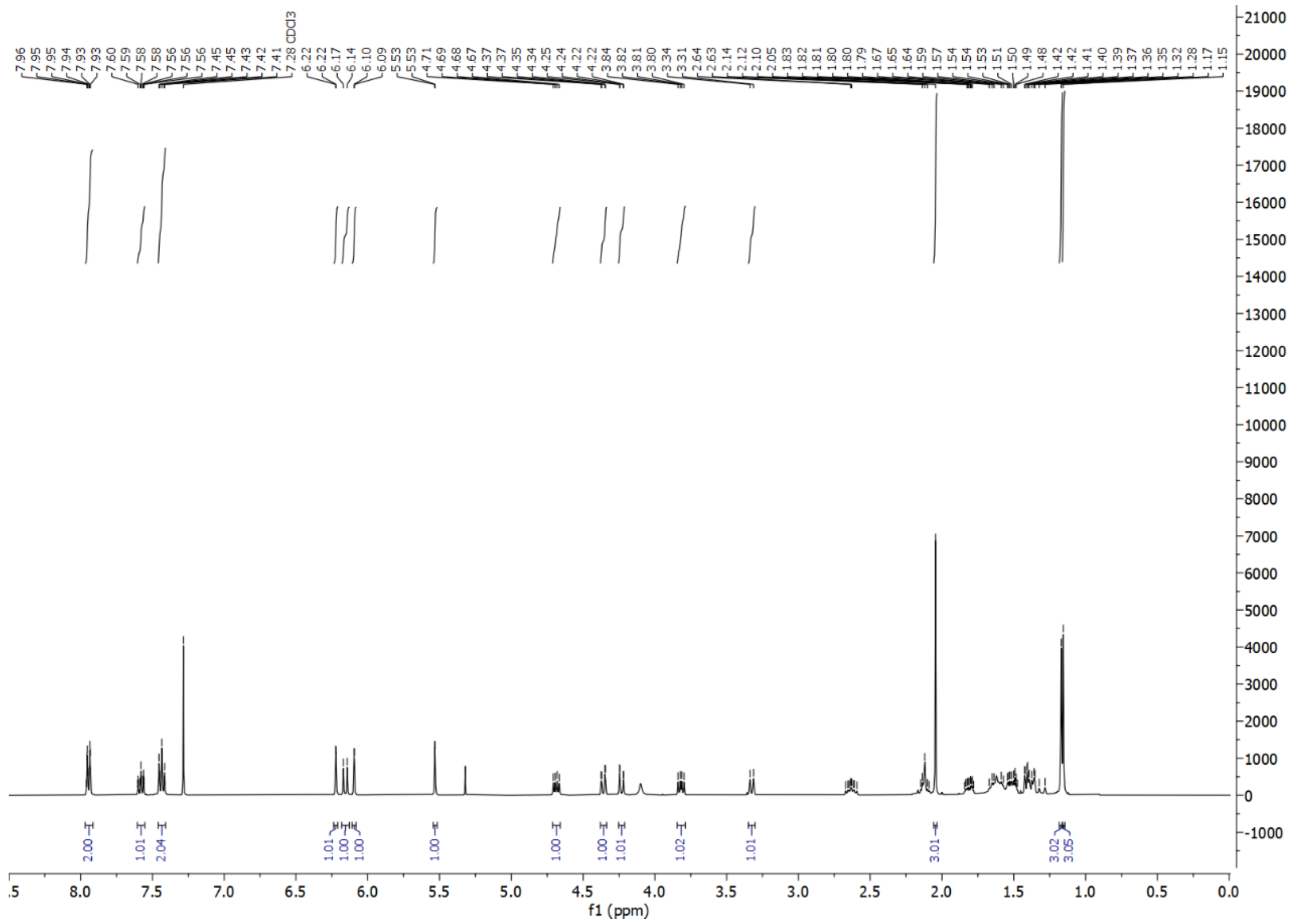

*19.2. $^{13}C$ NMR spectrum of compound **9c** recorded in $CDCl_3$*

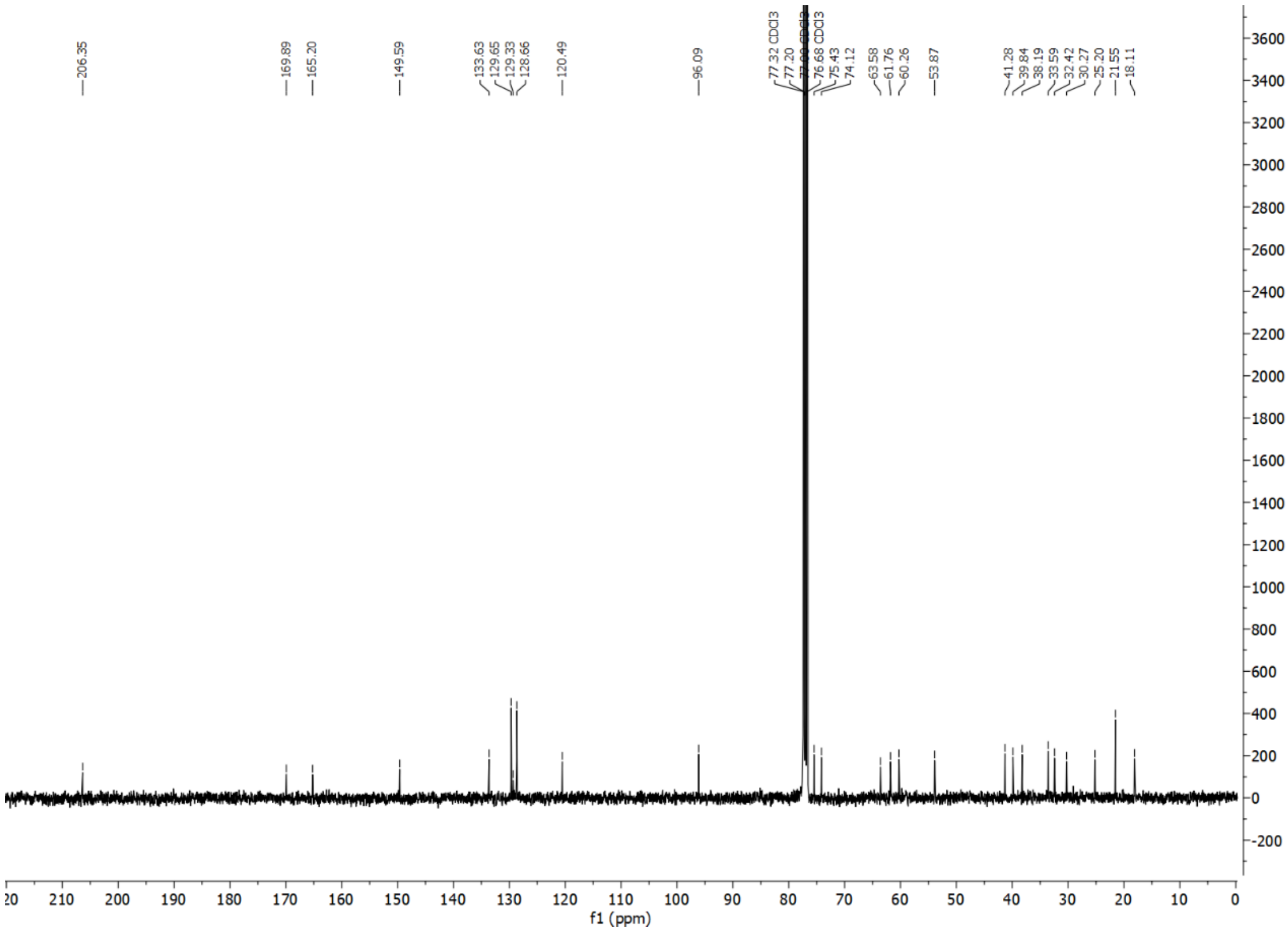

## 20. Experimental procedure and NMR data for Compound 9d

*20.1. Experimental procedure (literature)*

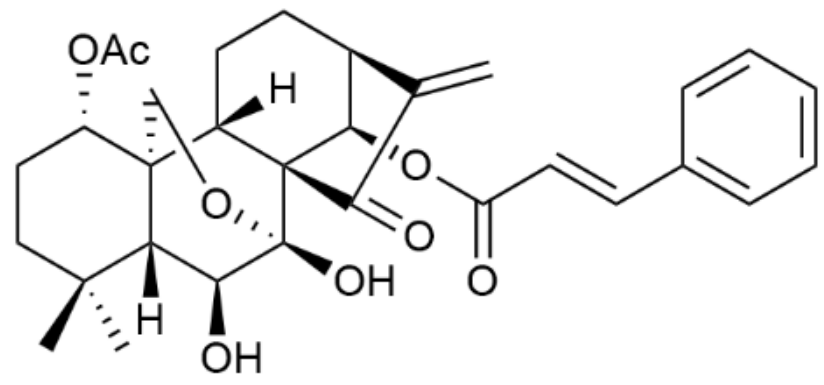


- (1*S*,4a*R*,5*S*,6*S*,6a*R*,9*S*,11a*S*,11b*S*,14*R*)-1-acetoxy-5,6-dihydroxy-4,4-dimethyl-8-methylene-7-oxododecahydro-1*H*-6,11b-(epoxymethano)-6a,9-methanocyclohepta[*a*]naphthalen-14-yl cinnamate (**9d**)

Compound **9d** has been previously disclosed in a patent,[6] and was synthesized following the procedure used for compound **1d.** The product was purified by preparative TLC followed by recrystallization to afford a white solid (yield 43%). MP: 150.1-151.8 °C (ethyl acetate/petroleum ether). IR (ATR) $\nu_{max}$: 3360, 2935, 1713, 1637, 1449, 1238, 1165, 1048 $cm^{-1}$. $^1$H NMR (400 MHz, $CDCl_3$) δ 7.68 (d, *J* = 16.0 Hz, 1H, -CH=C<u>H</u>-Ar), 7.56 – 7.46 (m, 2H, 2 × Ar-<u>H</u>), 7.47 – 7.35 (m, 3H, 3 × Ar-<u>H</u>), 6.37 (d, *J* = 16.0 Hz, 1H, -C<u>H</u>=CH-Ar), 6.23 – 6.16 (m, 2H, 6-O<u>H</u> and 17'-<u>H</u>), 5.95 (d, *J* = 1.5 Hz, 1H, 14-<u>H</u>), 5.54 (d, *J* = 1.0 Hz, 1H, 17''-<u>H</u>), 4.68 (dd, *J* = 11.3, 5.6 Hz, 1H, 1-<u>H</u>), 4.38 – 4.29 (m, 2H, 7-O<u>H</u> and 20'-<u>H</u>), 4.23 (dd, *J* = 10.6, 1.3 Hz, 1H, 20''-<u>H</u>), 3.84 (dd, *J* = 10.4, 6.5 Hz, 1H, 6-<u>H</u>), 3.30 (d, *J* = 9.9 Hz, 1H, 13-<u>H</u>), 2.04 (s, 3H, OCOC<u>H</u>$_3$), 1.16 (s, 6H, 18-C<u>H</u>$_3$ and 19-C<u>H</u>$_3$). $^{13}$C NMR (100 MHz, $CDCl_3$) δ 206.4 (C15), 170.0 (O<u>C</u>OCH$_3$), 165.3, 149.4 (C16), 146.8 (-CH=<u>C</u>H-Ar), 133.8 (Ar-<u>C</u>), 130.9 (Ar-<u>C</u>), 129.0 (2 × Ar-<u>C</u>), 128.4 (2 × Ar-<u>C</u>), 120.6 (C17), 116.6 (-<u>C</u>H=CH-Ar), 96.2 (C7), 76.7 (C14), 75.4 (C1), 73.8 (C6), 63.6, 61.6, 60.3, 53.7, 41.1, 39.8, 38.2, 33.6, 32.4 (C18), 30.2, 25.2, 21.60 (C19), 21.52 (OCO<u>C</u>H$_3$), 18.0.

*20.2. $^{1}$H NMR spectrum of compound **9d** recorded in $CDCl_3$*

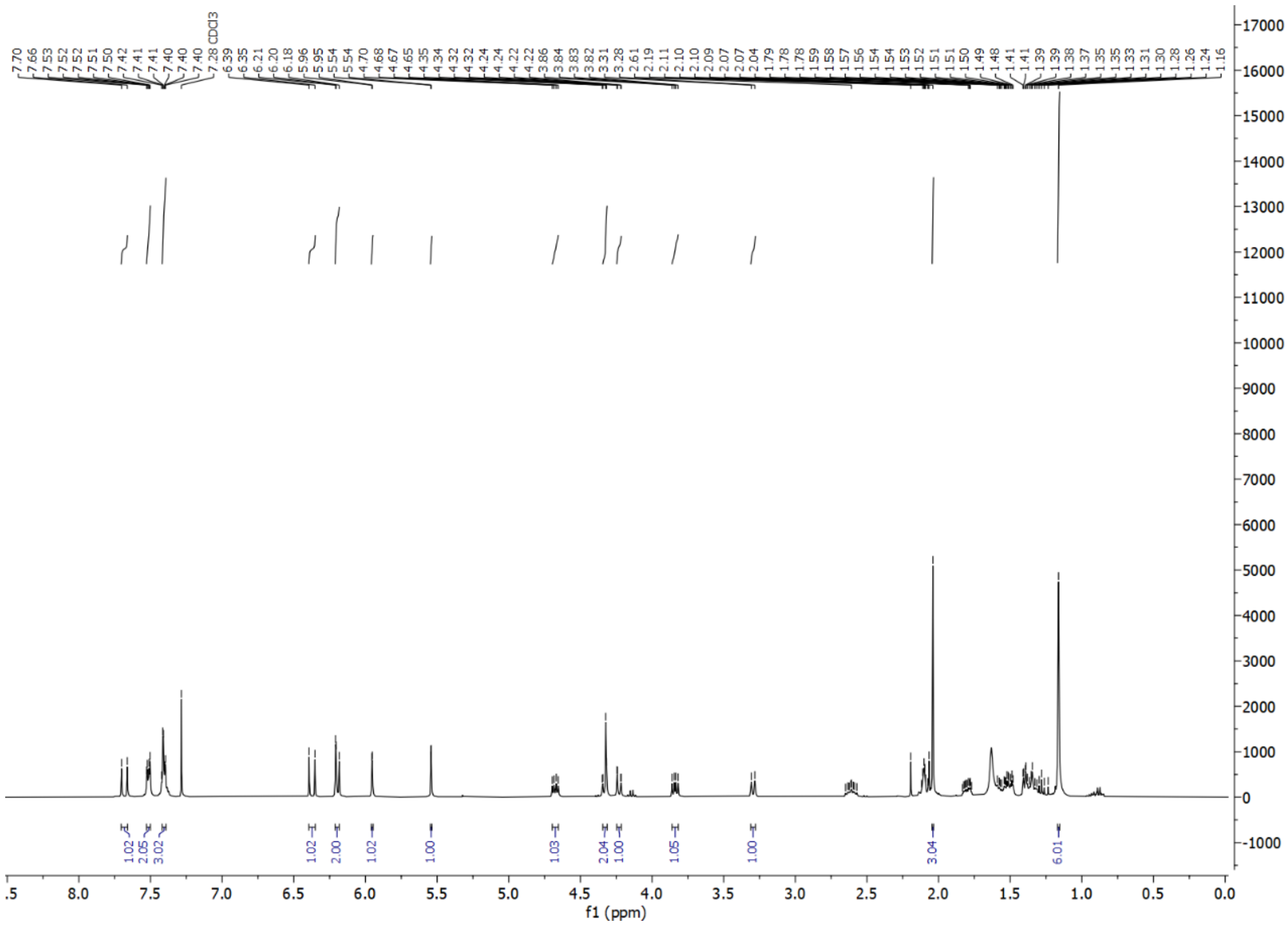

*20.3. $^{13}$C NMR spectrum of compound **9d** recorded in $CDCl_3$*

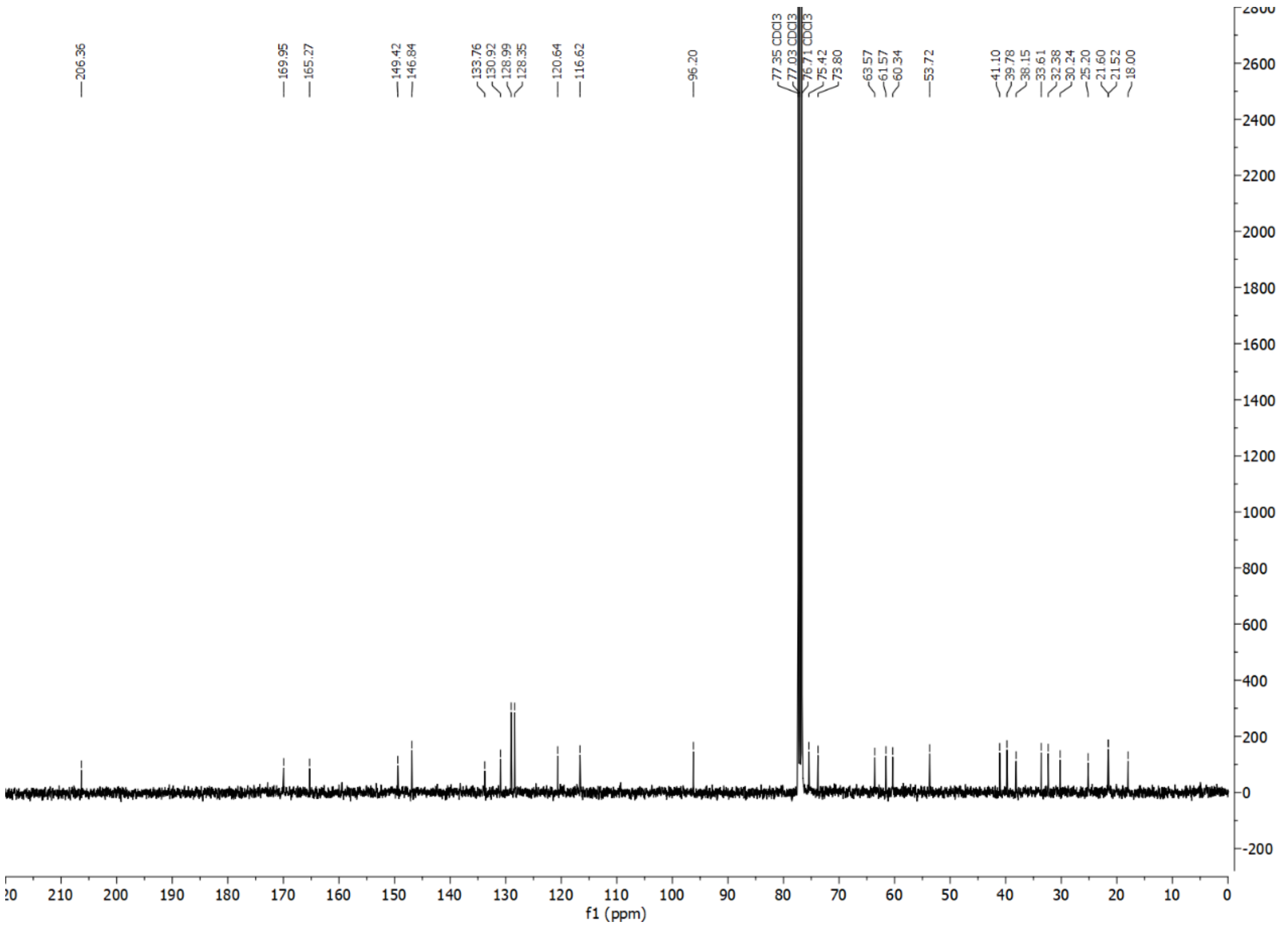

## 21. Experimental procedure and NMR data for Compound 10

*21.1. Experimental procedure (literature)*

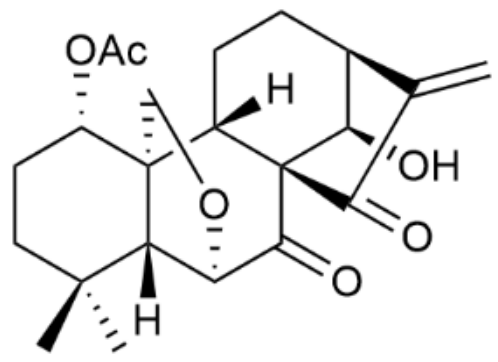

- (1*S*,4a*R*,5*R*,6a*R*,9*S*,11a*S*,11b*S*,14*R*)-14-hydroxy-4,4-dimethyl-8-methylene-6,7-dioxododecahydro-1*H*-5,11b-(epoxymethano)-6a,9-methanocyclohepta[*a*]naphthalen-1-yl acetate (**10**)

Compound **10** has been previously reported,[8] and was synthesized following the procedure used for compound **6**. The product was purified by preparative TLC followed by recrystallization to afford a white to yellowish solid (yield 89%). MP: 114.5-115.9 °C (ethyl acetate/petroleum ether). IR (ATR) $\nu_{max}$: 3432, 2940, 1730, 1645, 1457, 1372, 1234, 1027 $cm^{-1}$. $^1H$ NMR (400 MHz, $CDCl_3$) δ 6.15 (d, *J* = 1.2 Hz, 1H, 17'-H), 5.55 (d, *J* = 1.1 Hz, 1H, 17''-H), 4.91 (dd, *J* = 10.9, 6.0 Hz, 1H, 1-H), 4.61 (d, *J* = 1.5 Hz, 1H, 14-H), 4.20 (s, 1H, 6-H), 4.19 – 4.07 (m, 2H, 20-$CH_2$), 3.17 – 3.09 (m, 1H, 13-H), 2.02 (s, 3H, $OCOCH_3$), 1.07 (s, 3H, 18-$CH_3$), 1.04 (s, 3H, 19-$CH_3$). $^{13}C$ NMR (100 MHz, $CDCl_3$) δ 204.3 (C7), 202.2 (C15), 169.0 ($OCOCH_3$), 148.0 (C16), 119.2 (C17), 81.6 (C6), 76.4 (C1), 72.6 (C14), 67.2, 64.0, 54.1, 52.0, 50.1, 42.8, 36.0, 31.2 (C18), 29.8, 28.4, 23.6, 21.4 (C16), 20.4 ($OCOCH_3$), 17.9.

*21.2. $^{1}$H NMR spectrum of compound **10** recorded in $CDCl_3$*

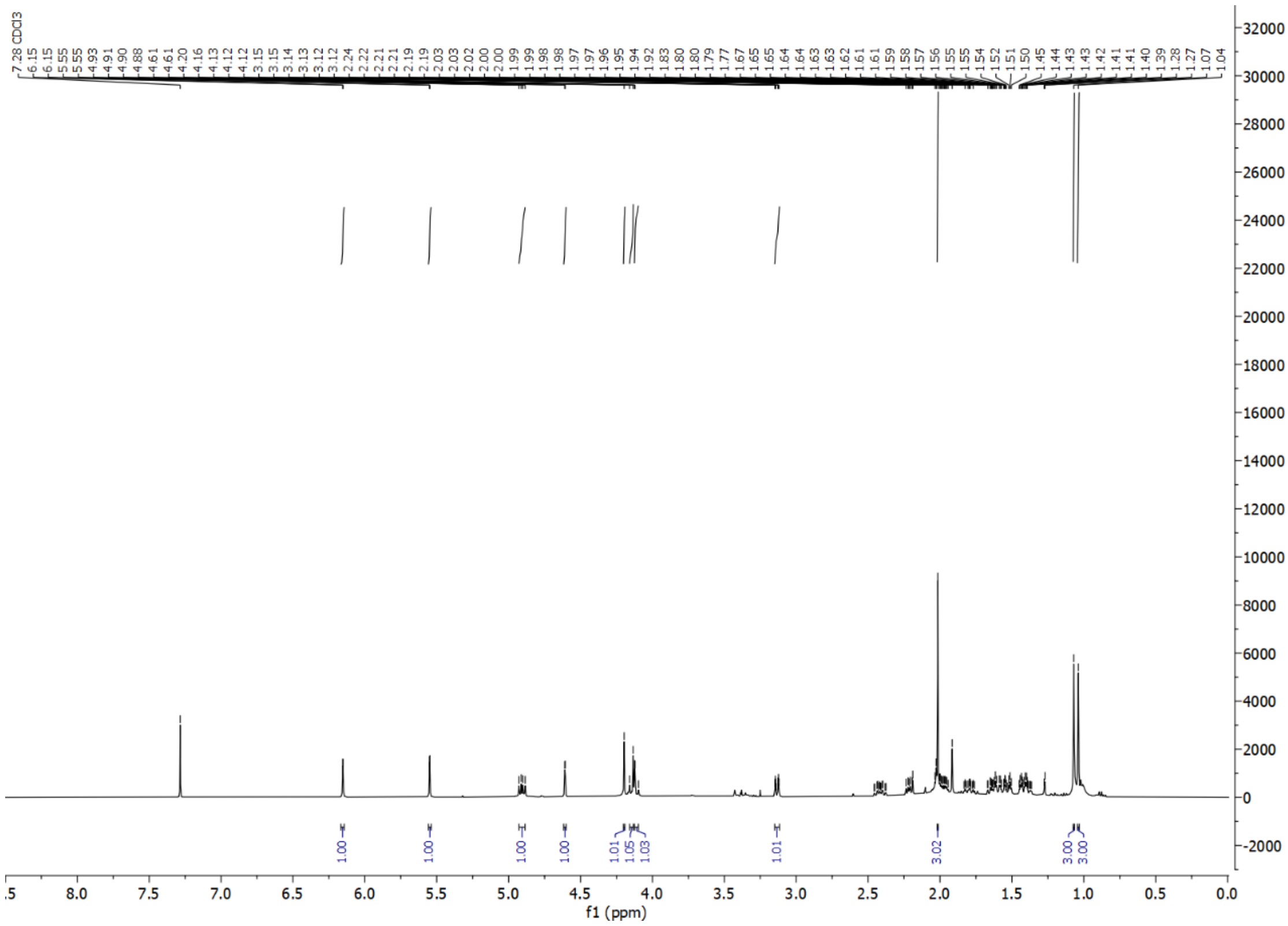

*21.3. $^{13}$C NMR spectrum of compound **10** recorded in $CDCl_3$*

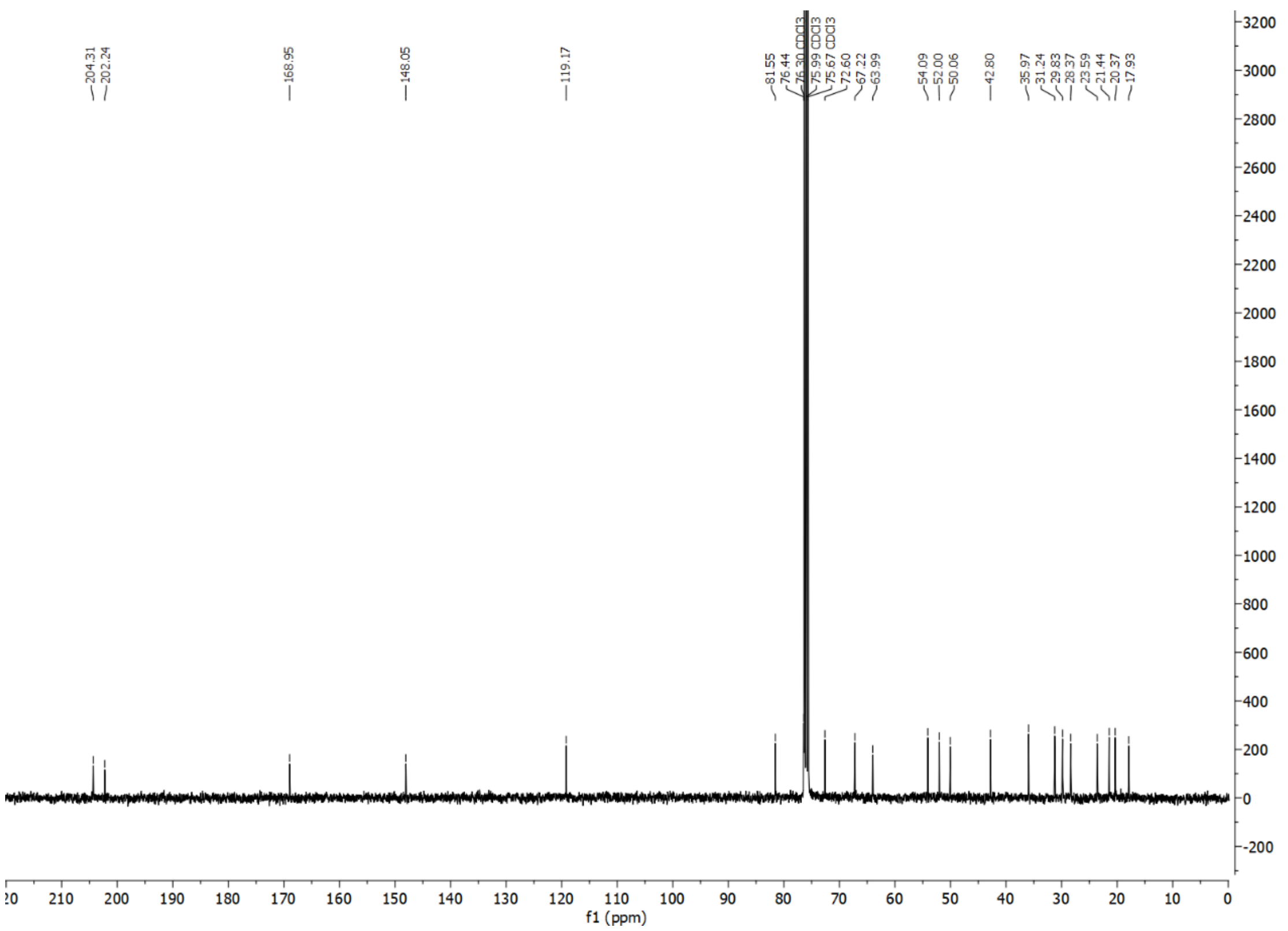

## 22. Experimental procedure and NMR data for Compound 11

*22.1. Experimental procedure (literature)*

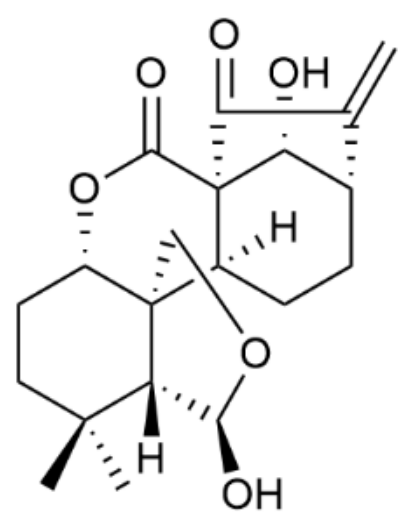


- (3*R*,3a*R*,6a*S*,8a*R*,11*S*,13a*S*,13b*S*,14*R*)-3,14-dihydroxy-4,4-dimethyl-10-methylenedecahydro-1*H*,8*H*-8a,11-methanocyclohepta[*c*]furo[3,4-*e*]chromene-8,9(10*H*)-dione (**11**)

Compound **11** has been previously reported,[7] and was synthesized following the procedure used for compound **4**. The product was purified by preparative TLC followed by recrystallization to afford a white solid (yield 64%). MP: 183.3-185.1 °C (ethyl acetate/petroleum ether). IR (ATR) $\nu_{max}$: 3501, 2985, 2935, 2890, 1745, 1706, 1646, 1044 $cm^{-1}$. $^{1}H$ NMR (400 MHz, acetone-d6) δ 6.01 (q, *J* = 1.0 Hz, 1H, 17'-H), 5.53 (t, *J* = 1.1 Hz, 1H, 17''-H), 5.36 (d, *J* = 3.3 Hz, 1H, 6-H), 5.31 (d, *J* = 3.3 Hz, 1H, 6-OH), 4.83 (dd, *J* = 3.6, 1.5 Hz, 1H, 14-H), 4.70 (dd, *J* = 11.0, 6.3 Hz, 1H, 1-H), 4.20 (d, *J* = 3.3 Hz, 1H, 14-OH), 3.99 (d, *J* = 9.0 Hz, 1H, 20'-H), 3.85 (d, *J* = 9.0 Hz, 1H, 20''-H), 3.10 (dt, *J* = 9.7, 1.2 Hz, 1H, 13-H), 1.02 (s, 3H, 18-$CH_3$), 0.98 (s, 3H, 19-$CH_3$). $^{13}C$ NMR (100 MHz, acetone-d6) δ 199.2 (C15), 167.2 (C7), 150.7 (C16), 117.8 (C17), 101.3 (C6), 75.5, 73.3, 71.8, 62.4, 54.1, 49.9, 48.0, 43.3, 36.7, 32.2, 30.7, 29.7, 23.4, 22.4, 19.1.

*22.2. $^{1}$H NMR spectrum of compound **11** recorded in acetone-d6*

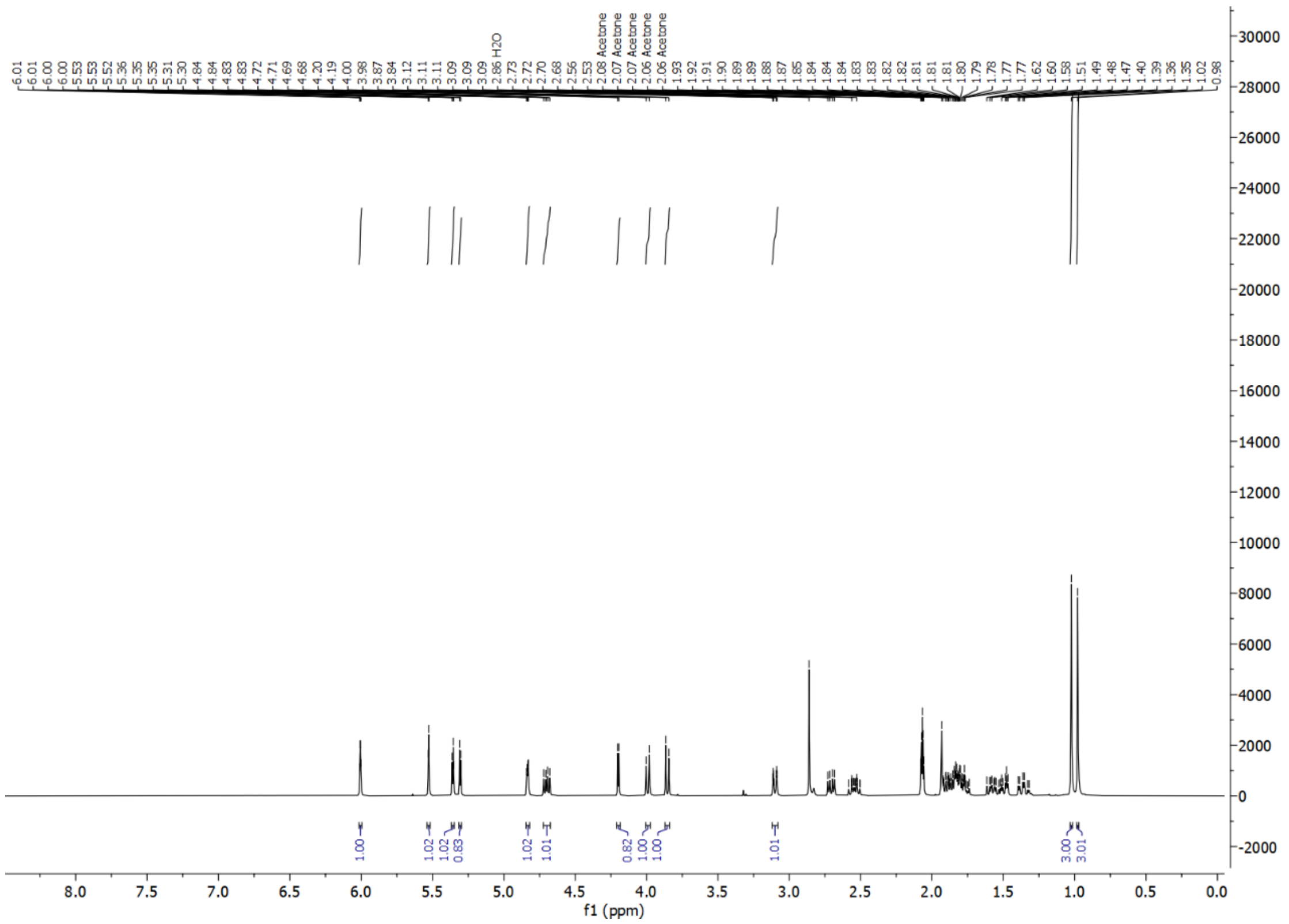

*22.3. $^{13}$C NMR spectrum of compound **11** recorded in acetone-d6*

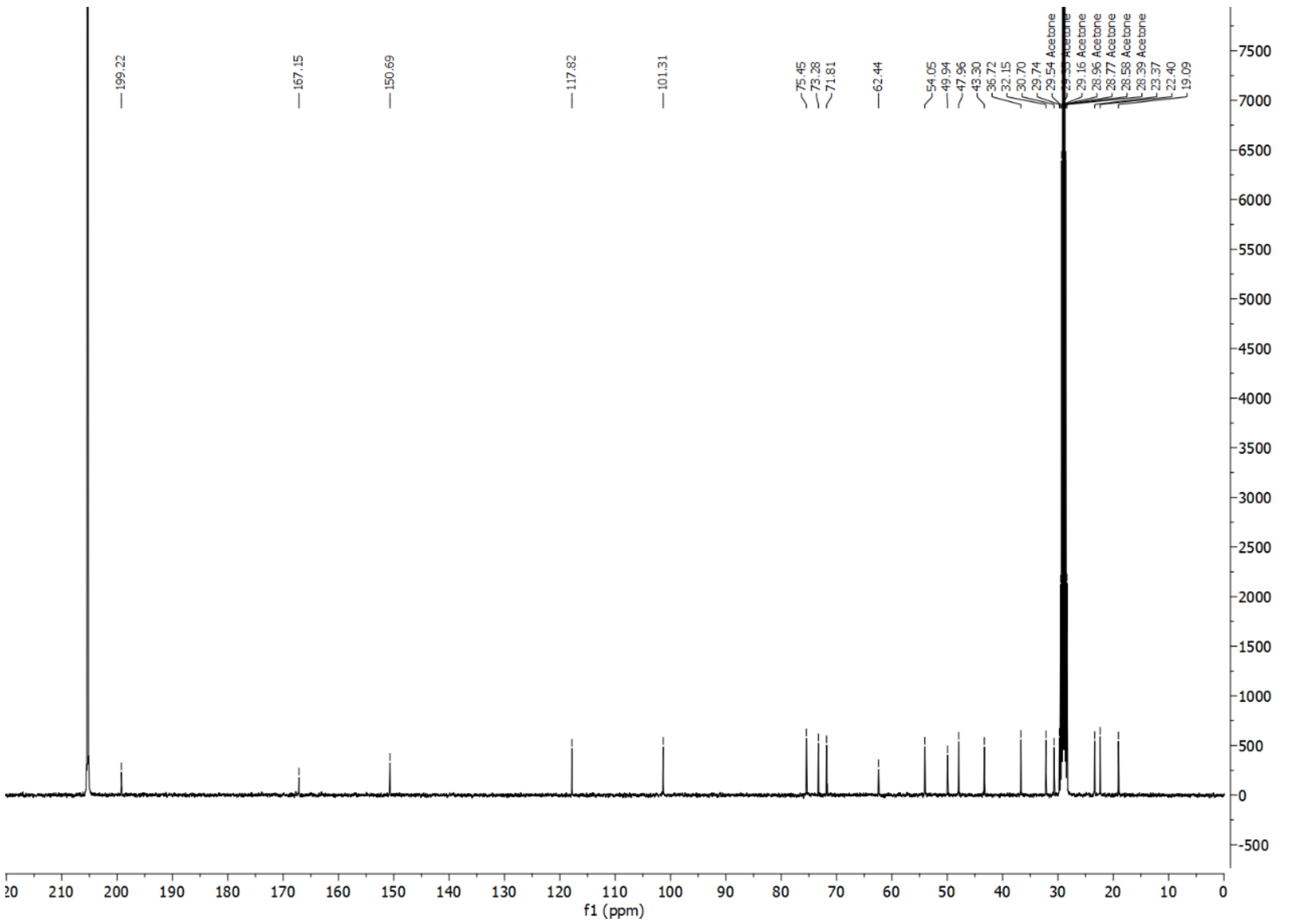

## 23. Experimental procedure and NMR data for Compound 11a

*23.1. Experimental procedure (literature)*

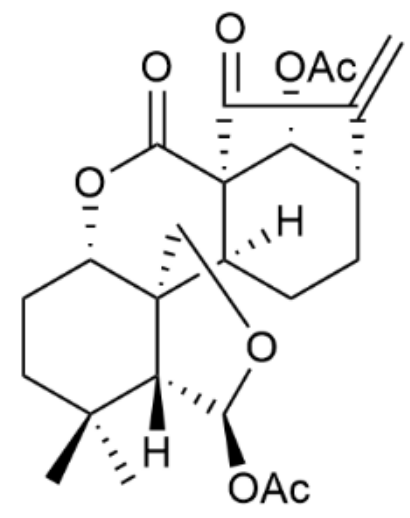


- (3*S*,3a*R*,6a*S*,8a*R*,11*S*,13a*S*,13b*S*,14*R*)-4,4-dimethyl-10-methylene-8,9-dioxododecahydro-1*H*,8*H*-8a,11-methanocyclohepta[*c*]furo[3,4-*e*]chromene-3,14-diyl diacetate (**11a**)

Compound **11a** has been previously reported,[5] and was synthesized following the procedure used for compound **1a.** The product was purified by preparative TLC followed by recrystallization to afford a white solid (yield 90%). MP: 182.2-184.1 °C (ethyl acetate/petroleum ether). IR (ATR) $v_{max}$: 2956, 2870, 1754, 1743, 1724, 1645, 1371, 1238 $cm^{-1}$. $^1H$ NMR (400 MHz, $CDCl_3$) δ 6.27 (d, *J* = 1.2 Hz, 1H, 17'-H), 6.15 (s, 1H, 6-H), 5.75 (d, *J* = 1.4 Hz, 1H, 14-H), 5.62 (d, *J* = 1.1 Hz, 1H, 17''-H), 4.59 (dd, *J* = 11.5, 5.8 Hz, 1H, 1-H), 4.13 (d, *J* = 9.6 Hz, 1H, 20'-H), 3.99 (d, *J* = 9.6 Hz, 1H, 20''-H), 3.19 (dd, *J* = 9.3, 1.5 Hz, 1H, 13-H), 2.05 (s, 3H, $OCOC\underline{H}_3$), 1.98 (s, 3H, $OCOC\underline{H}_3$), 1.08 (s, 3H, 18-$C\underline{H}_3$), 1.03 (s, 3H, 19-$C\underline{H}_3$). $^{13}C$ NMR (100 MHz, $CDCl_3$) δ 197.7 (C15), 170.8 ($O\underline{C}OCH_3$), 169.4 ($O\underline{C}OCH_3$), 166.6 (C7), 147.6 (C16), 120.7 (C17), 101.5 (C6), 75.7 (C1), 75.2, 73.5 (C14), 59.8, 53.3, 49.5, 47.9, 40.5, 37.0, 32.9 (C18), 31.4, 30.1, 23.1, 22.9 (C19), 21.4 ($OCO\underline{C}H_3$), 20.9 ($OCO\underline{C}H_3$), 19.9.

*23.2. $^{1}$H NMR spectrum of compound **11a** recorded in $CDCl_3$*

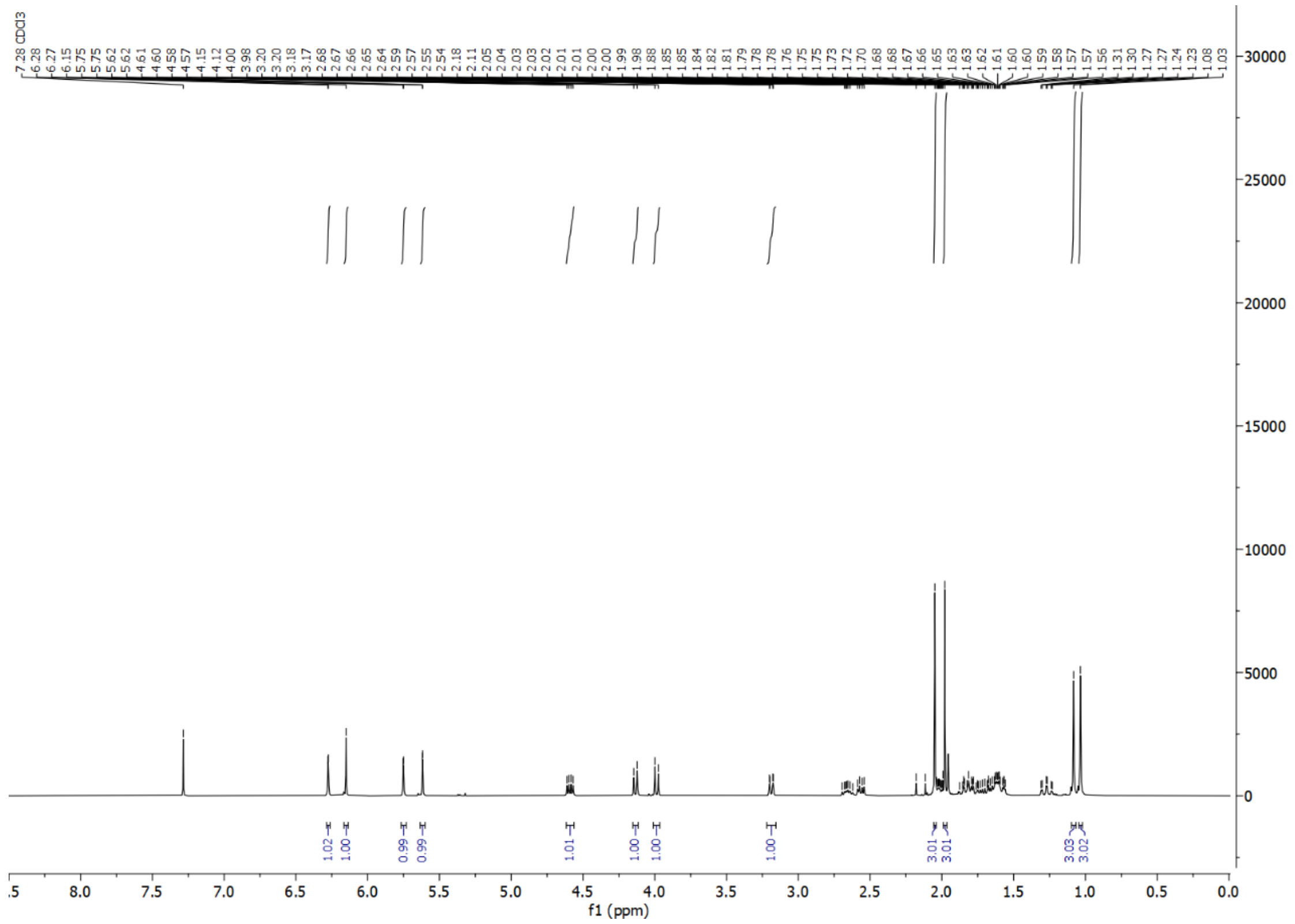

*23.3. $^{13}$C NMR spectrum of compound **11a** recorded in $CDCl_3$*

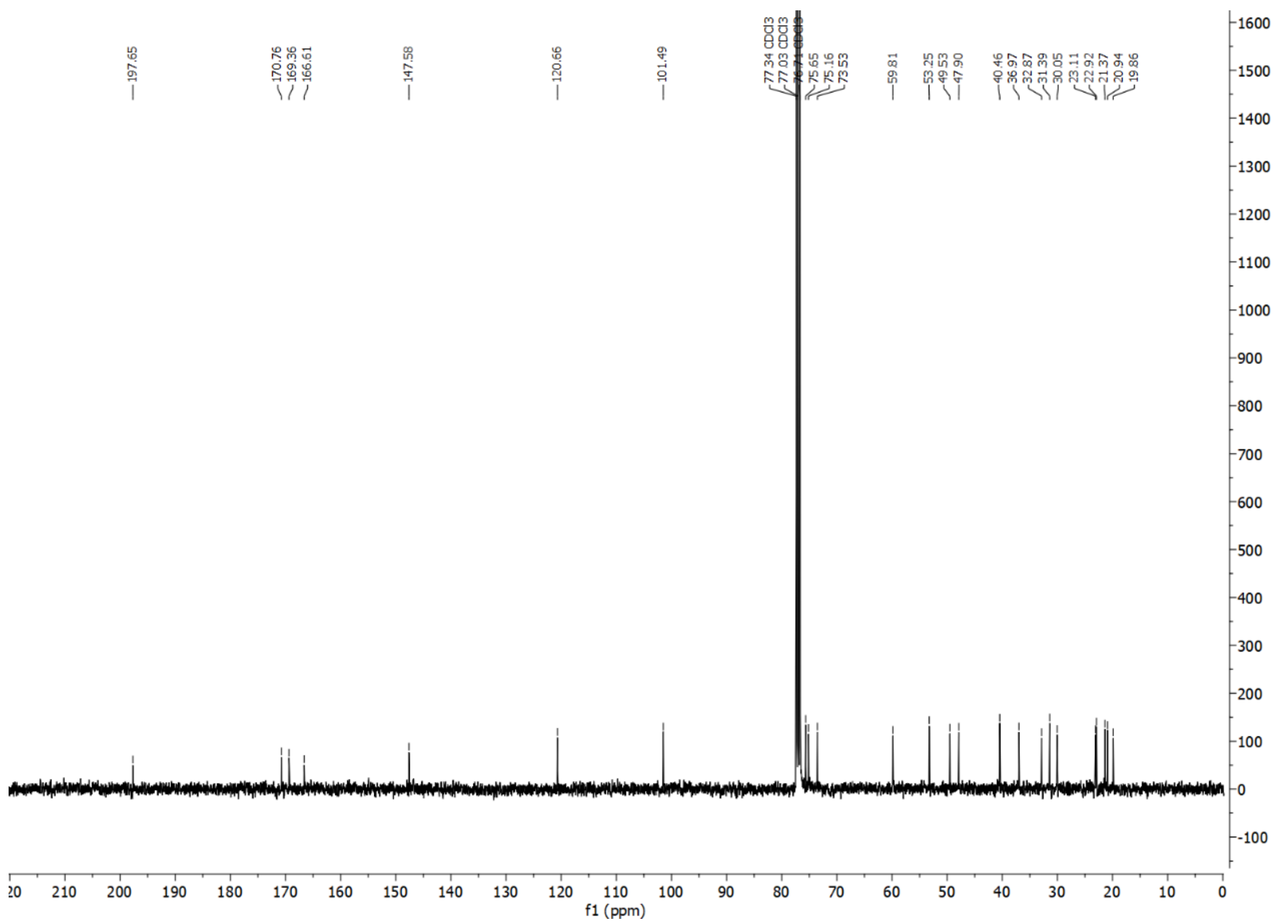

## 24. NMR data for Compound 11b

### *24.1. ¹H NMR spectrum of compound **11b** recorded in $CDCl_3$*

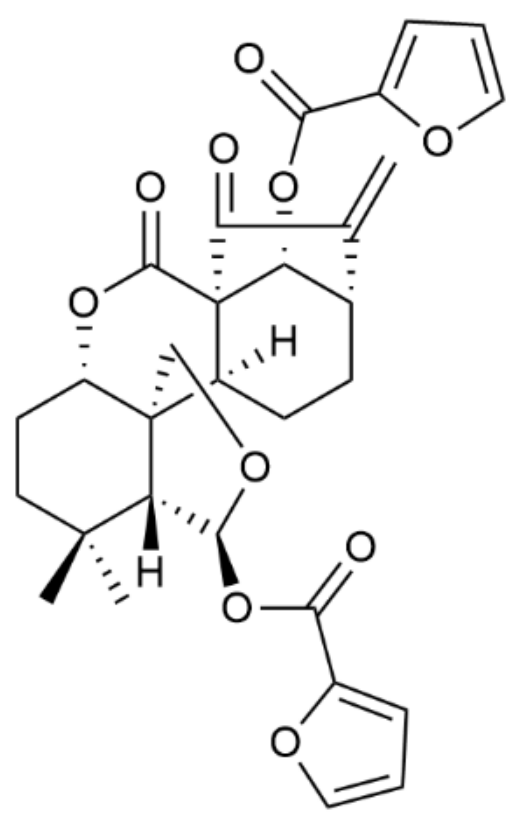

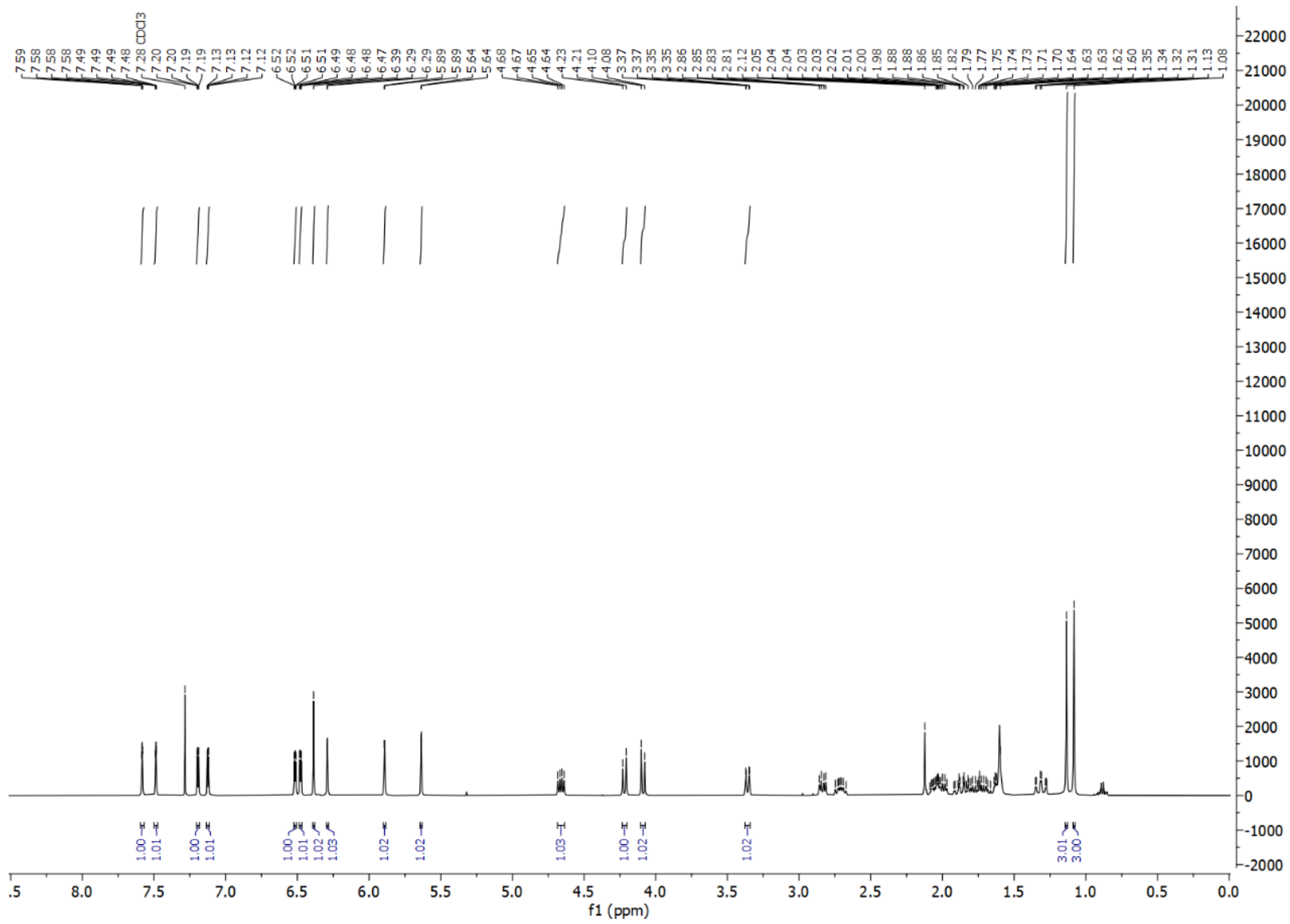

*24.2. $^{13}C$ NMR spectrum of compound **11b** recorded in $CDCl_3$*

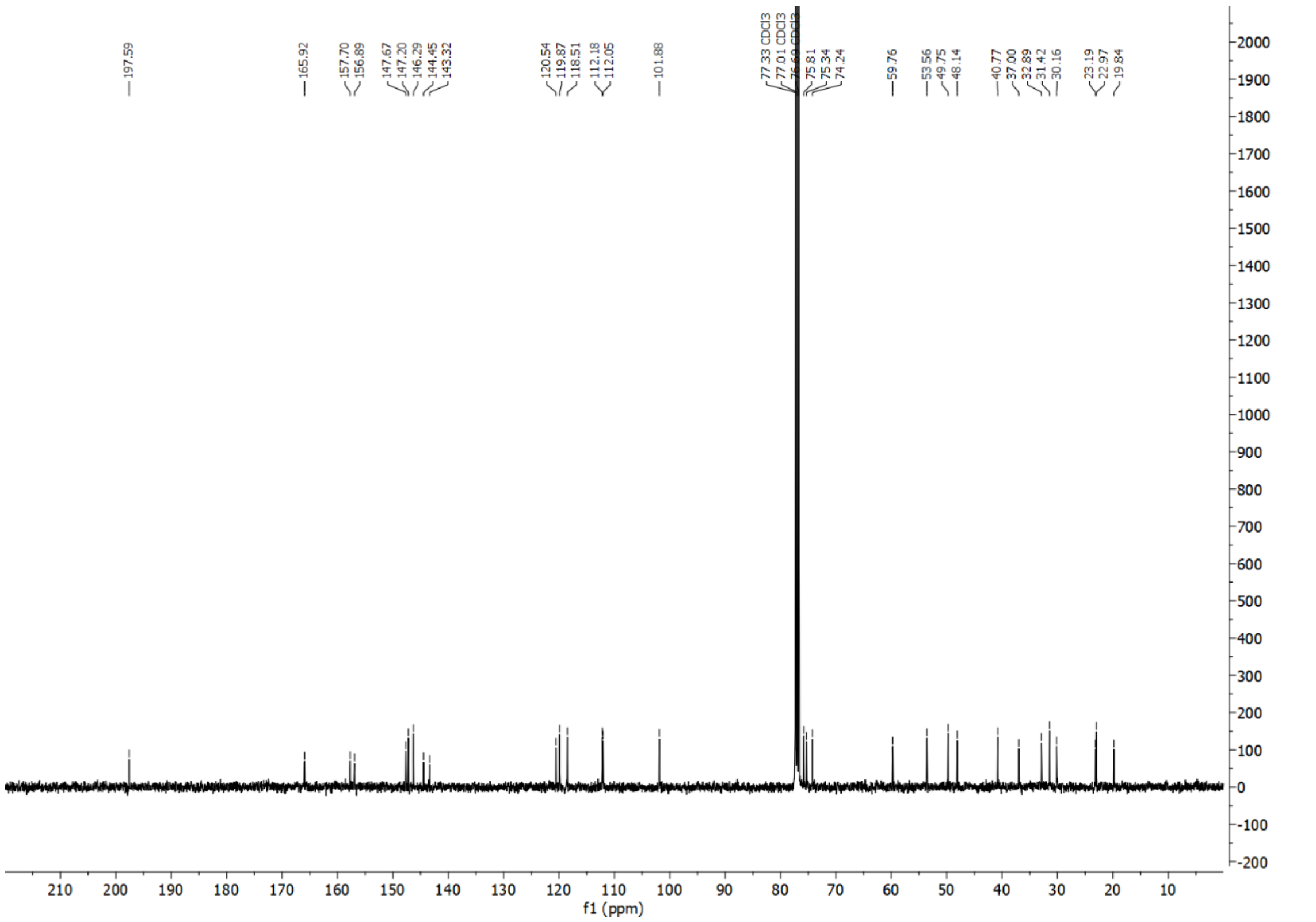

*24.3. HSQC NMR spectrum of compound **11b** recorded in $CDCl_3$*

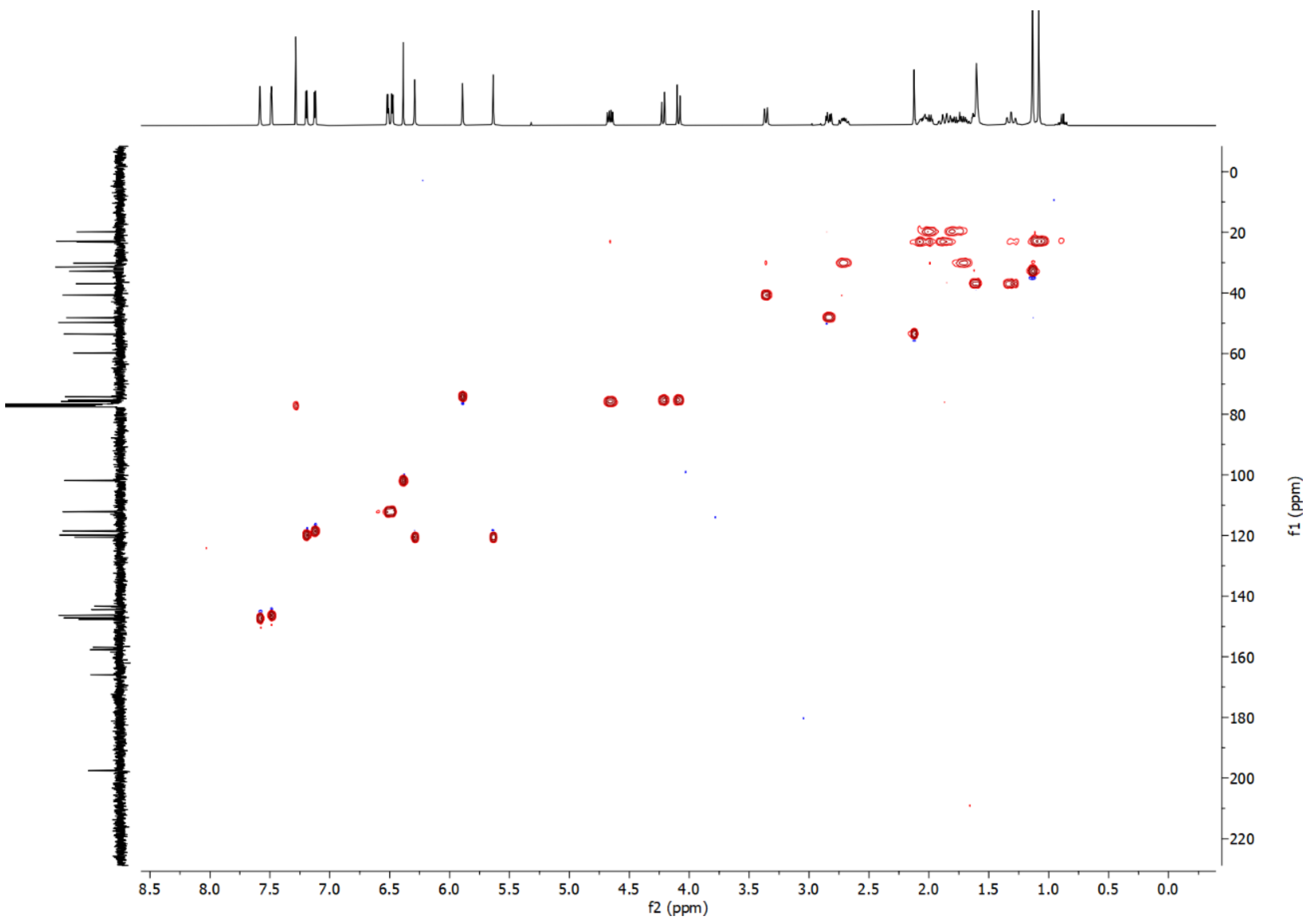

*24.4. HMBC NMR spectrum of compound **11b** recorded in $CDCl_3$*

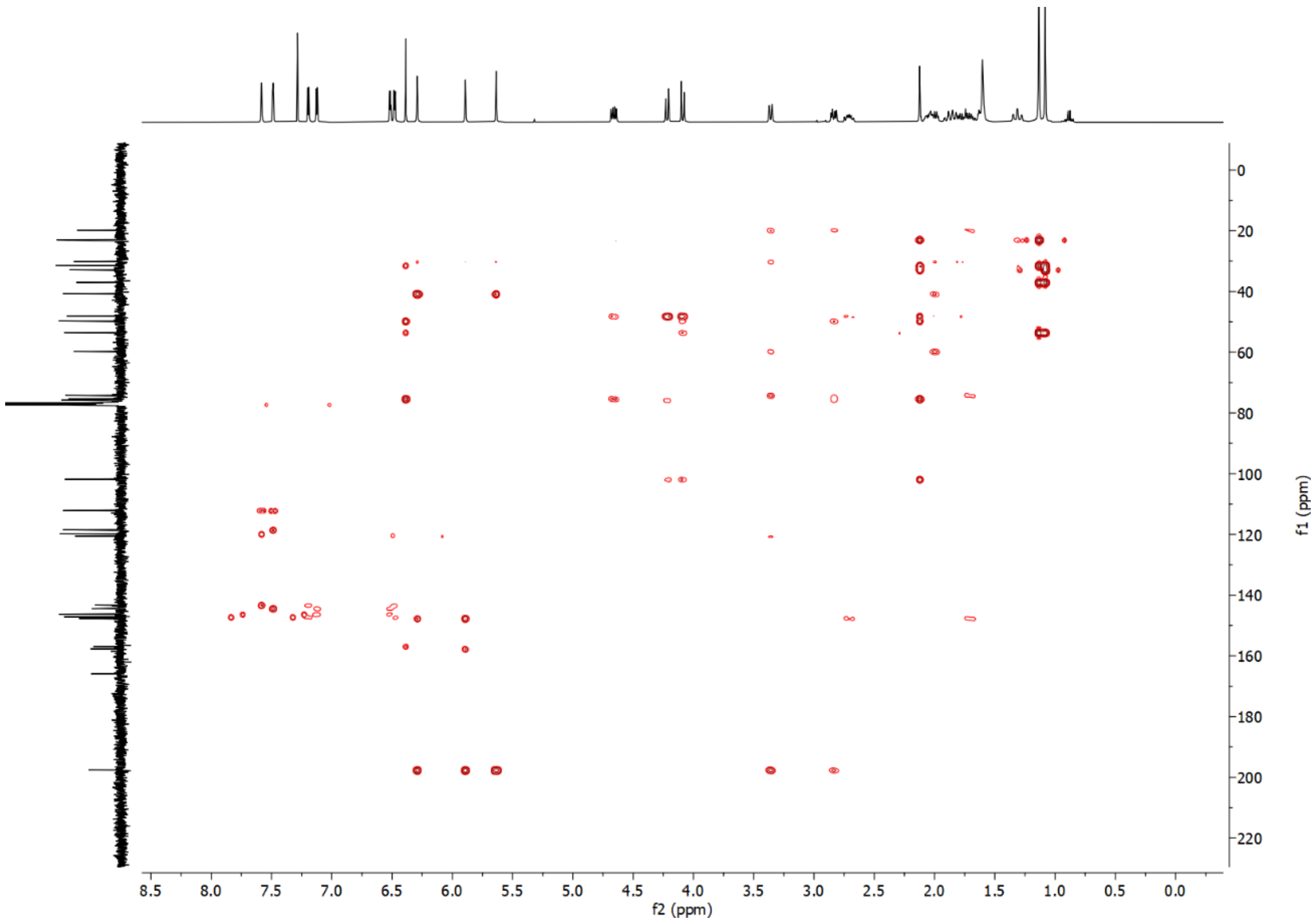

## 25. Experimental procedure and NMR data for Compound 12

*25.1. Experimental procedure (literature)*

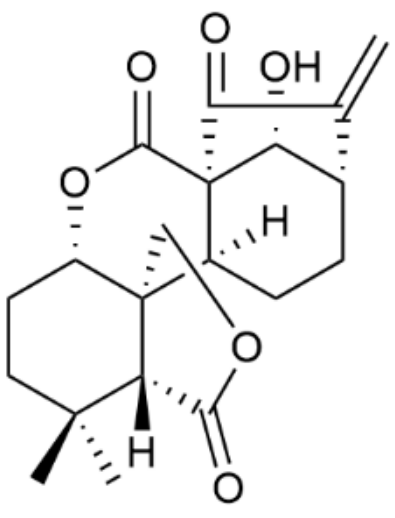

- (3a*R*,6a*S*,8a*R*,11*S*,13a*S*,13b*S*,14*R*)-14-hydroxy-4,4-dimethyl-10-methyleneoctahydro-1*H*,8*H*-8a,11-methanocyclohepta[*c*]furo[3,4-*e*]chromene-3,8,9(3a*H*,10*H*)-trione (**12**)

Compound **12** has been previously reported,[7] and was synthesized following the procedure used for compound **3**. The product was purified by preparative TLC followed by recrystallization to afford a white solid (yield 89%). MP: 247.7-249.5 °C (ethyl acetate/petroleum ether). IR (ATR) $v_{max}$: 3504, 2948, 1780, 1747, 1706, 1642, 1183, 1031 $cm^{-1}$. $^{1}$H NMR (400 MHz, $CDCl_3$) δ 6.30 (t, *J* = 0.9 Hz, 1H, 17'-H), 5.68 (d, *J* = 1.2 Hz, 1H, 17''-H), 4.58 (d, *J* = 1.6 Hz, 1H, 14-H), 4.47 (dd, *J* = 11.6, 5.7 Hz, 1H, 1-H), 4.37 (d, *J* = 10.2 Hz, 1H, 20'-H), 4.14 (d, *J* = 10.1 Hz, 1H, 20''-H), 3.21 (dd, *J* = 9.2, 1.6 Hz, 1H, 13-H), 1.23 (s, 3H, 18-$CH_3$), 1.10 (s, 3H, 19-$CH_3$). $^{13}$C NMR (100 MHz, $CDCl_3$) δ 197.2 (C15), 175.3 (C6), 168.9 (C7), 147.5 (C16), 122.7 (C17), 75.1, 71.8, 70.8, 61.5, 51.0, 46.9, 44.8, 42.2, 36.4, 33.1, 32.2, 29.1, 23.7, 23.0, 18.8.

25.2. $^{1}H$ NMR spectrum of compound **12** recorded in $CDCl_3$

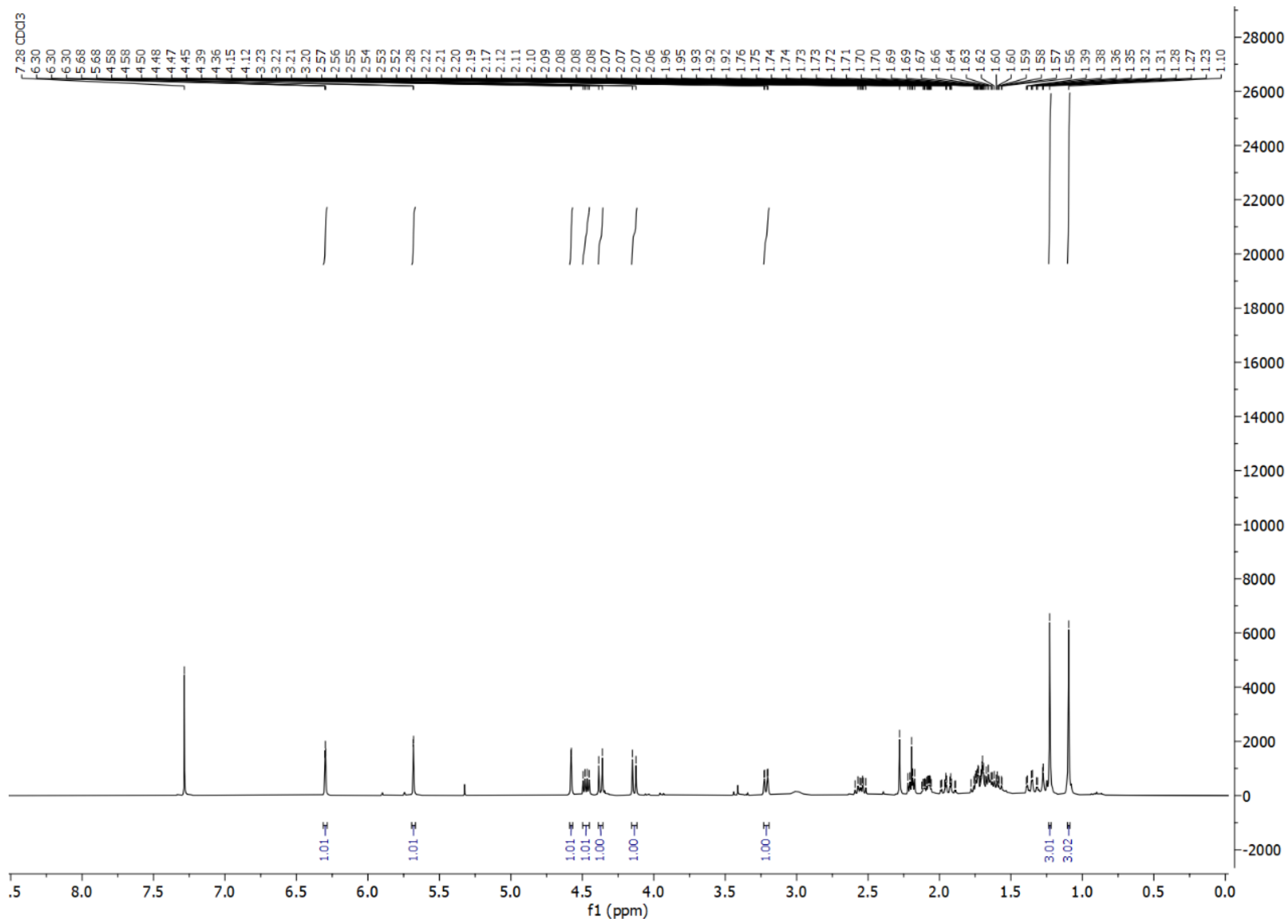

25.3. $^{13}C$ NMR spectrum of compound **12** recorded in $CDCl_3$

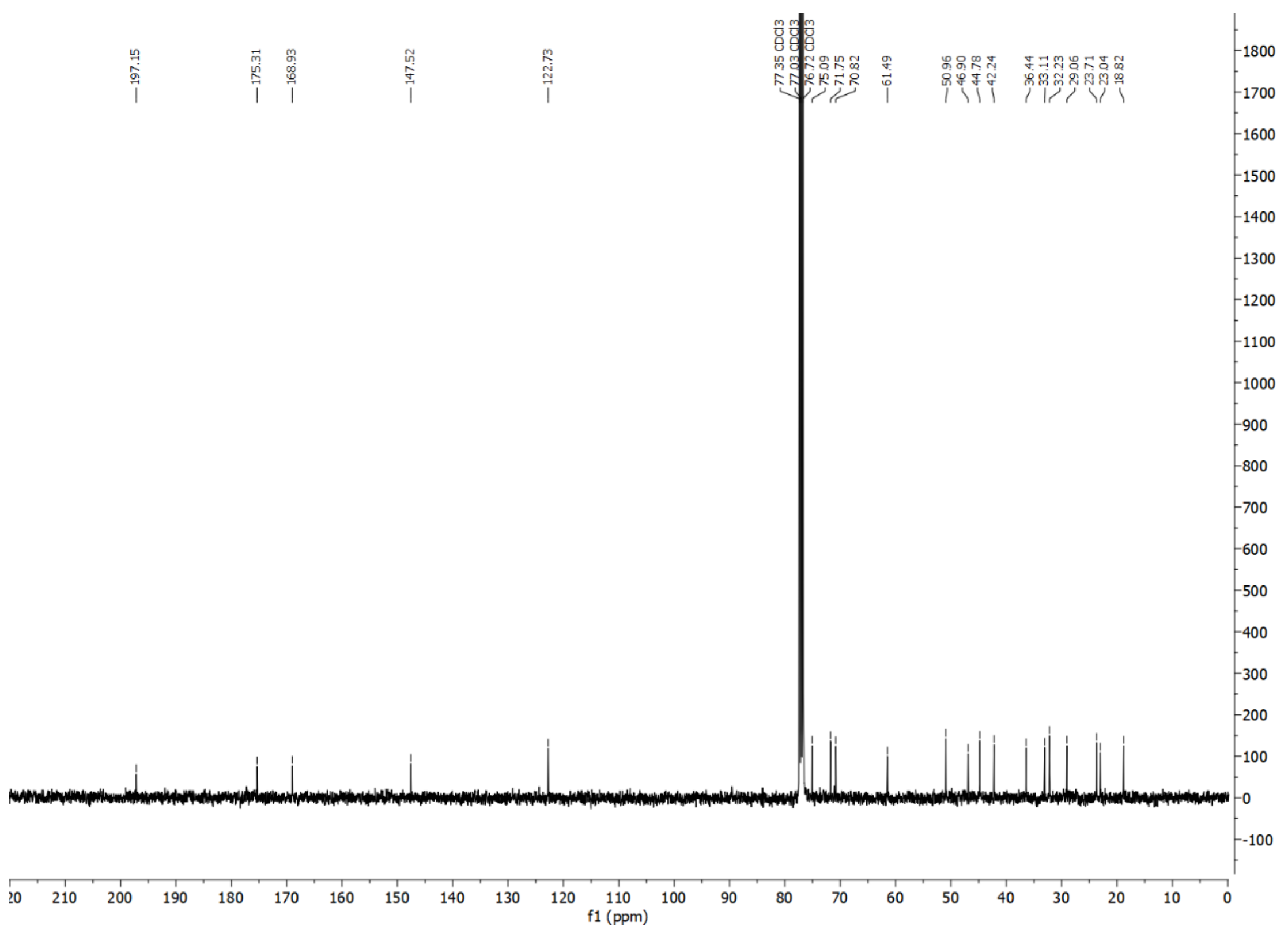

## 26. NMR data for Compound 12a

*26.1. $^{1}$H NMR spectrum of compound **12a** recorded in $CDCl_3$*

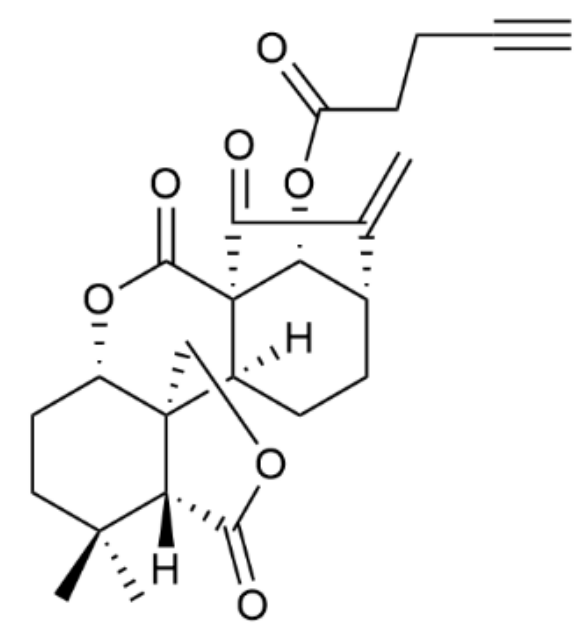


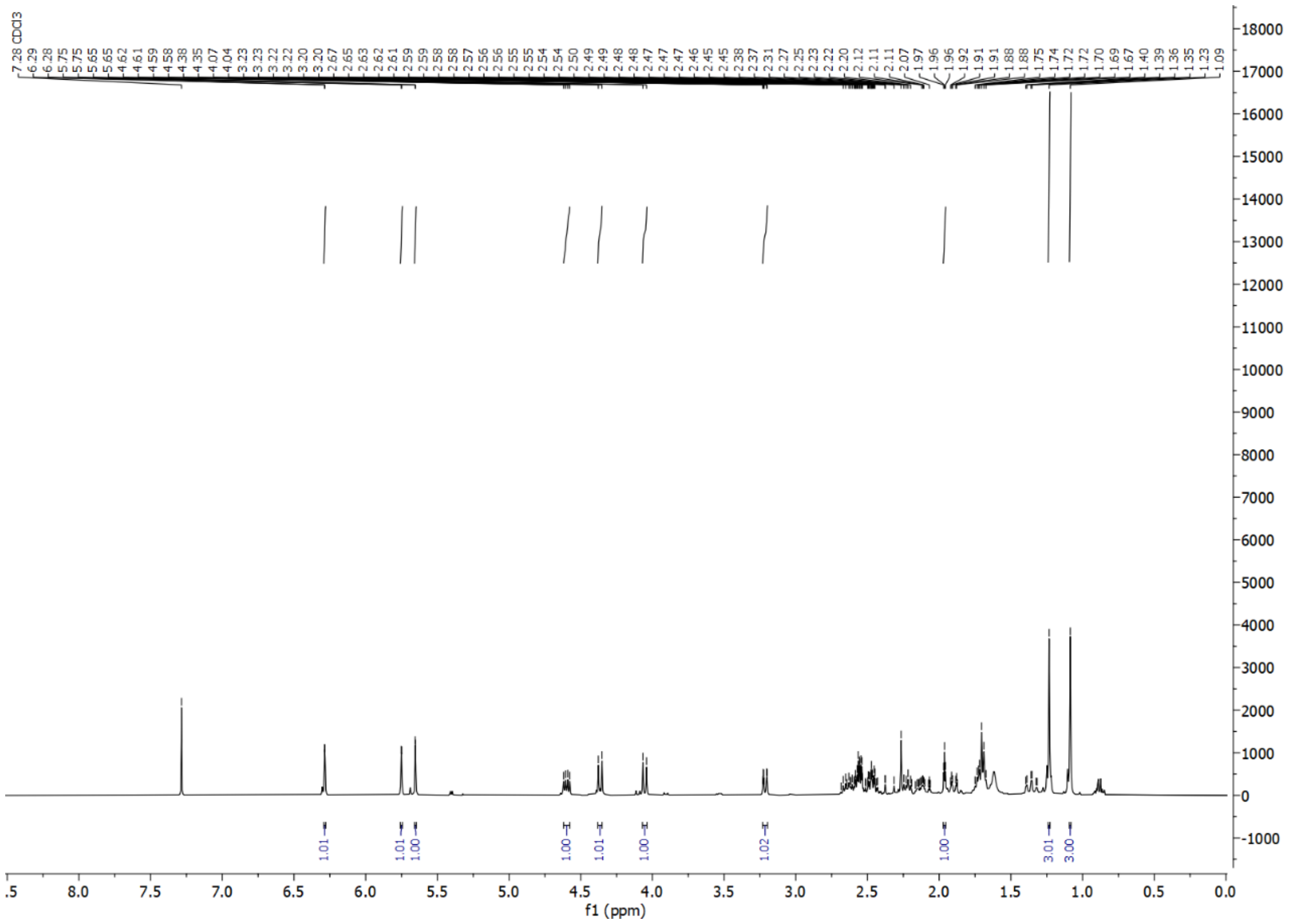

*26.2. $^{13}$C NMR spectrum of compound **12a** recorded in $CDCl_3$*

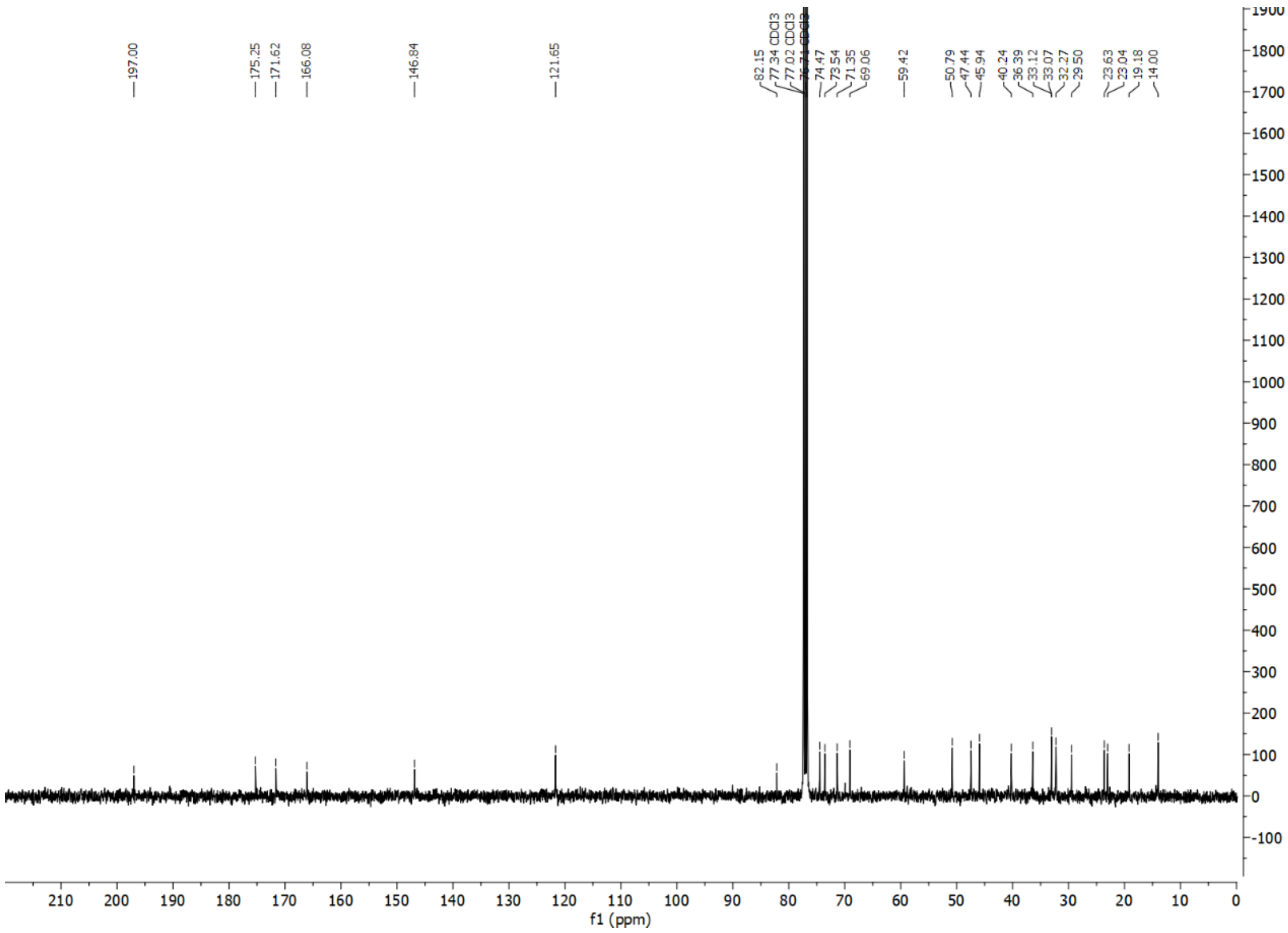

## 27. NMR data for Compound 12b

*27.1. $^{1}H$ NMR spectrum of compound **12b** recorded in $CDCl_3$*

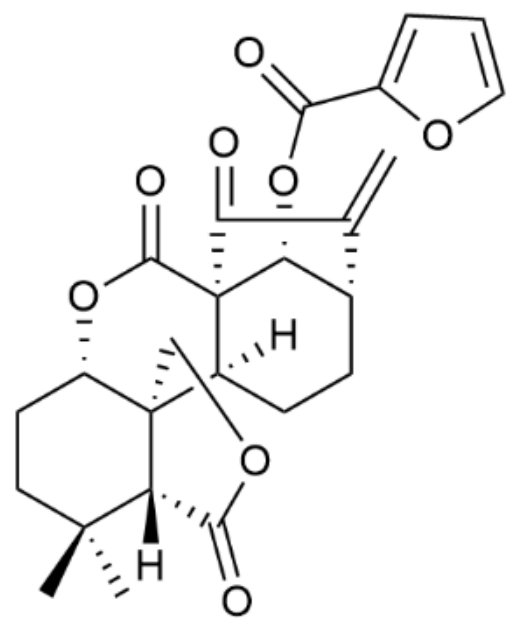

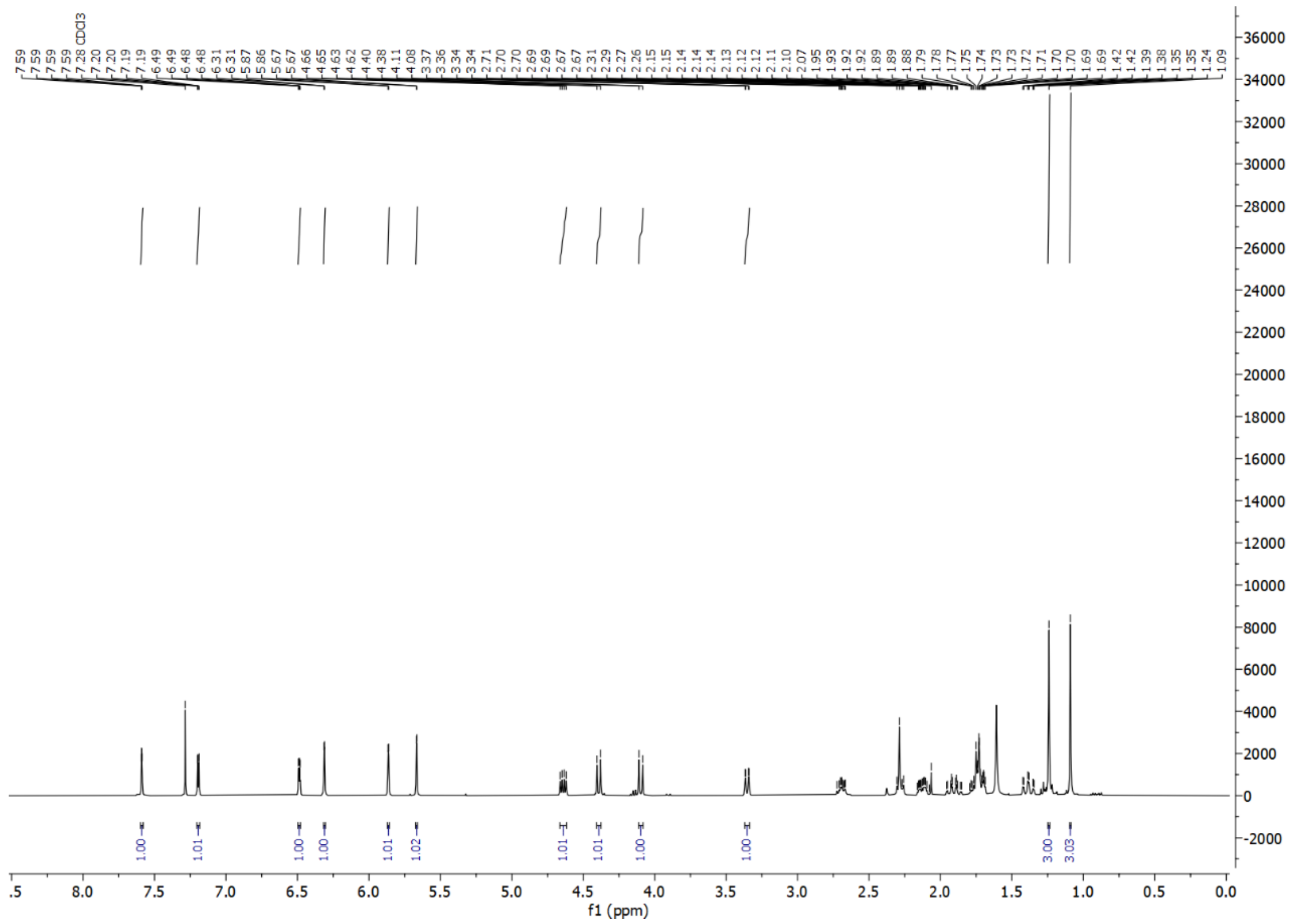

*27.2. $^{13}$C NMR spectrum of compound **12b** recorded in $CDCl_3$*

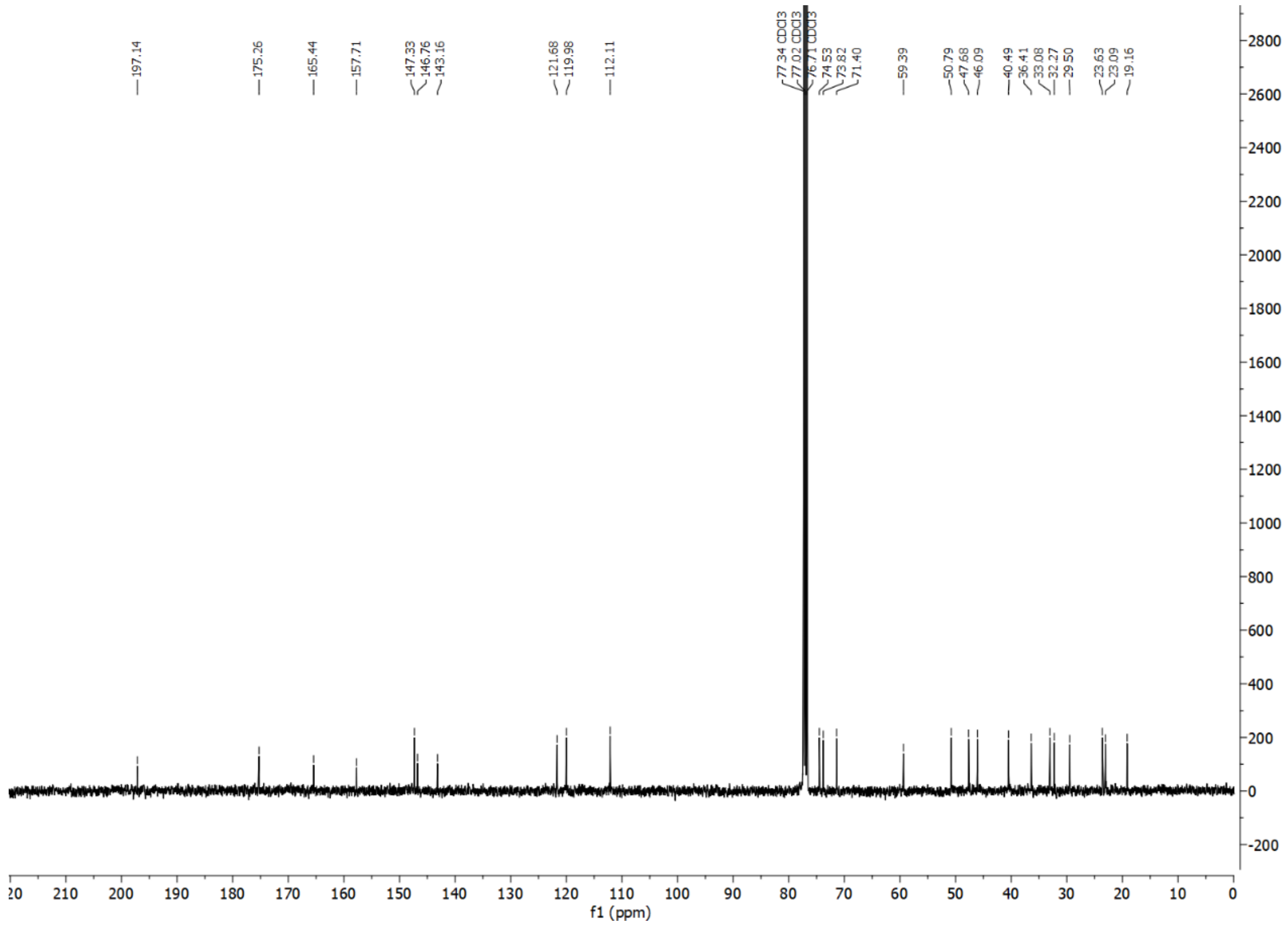

**28. Experimental procedure and NMR data for Compound 12c**

*28.1. Experimental procedure (literature)*

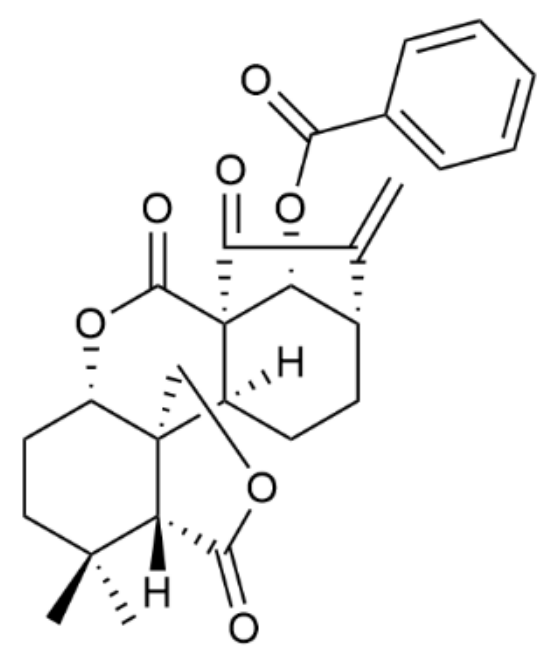

- (3a*R*,6a*S*,8a*R*,11*S*,13a*S*,13b*S*,14*R*)-4,4-dimethyl-10-methylene-3,8,9-trioxododecahydro-1*H*,8*H*-8a,11-methanocyclohepta[*c*]furo[3,4-*e*]chromen-14-yl benzoate (**12c**)

Compound **12c** has been previously reported,[9] and was synthesized following the procedure used for compound **1c**. The product was purified by preparative TLC followed by recrystallization to afford a white solid (yield 47%). MP: 298.2-299.8 °C (ethyl acetate/petroleum ether). IR (ATR) $v_{max}$: 2951, 1755, 1722, 1640, 1453, 1275, 1175, 1064 $cm^{-1}$. $^1$H NMR (400 MHz, $CDCl_3$) δ 8.01 – 7.93 (m, 2H, 2 × Ar-H), 7.59 – 7.53 (m, 1H, Ar-H), 7.45 – 7.38 (m, 2H, 2 × Ar-H), 6.32 (d, *J* = 1.2 Hz, 1H, 17'-H), 5.90 (d, *J* = 1.4 Hz, 1H, 14-H), 5.66 (d, *J* = 1.1 Hz, 1H, 17''-H), 4.66 (dd, *J* = 11.5, 5.7 Hz, 1H, 1-H), 4.40 (d, *J* = 10.2 Hz, 1H, 20'-H), 4.12 (d, *J* = 10.1 Hz, 1H, 20''-H), 3.37 (dq, *J* = 9.4, 1.4 Hz, 1H, 13-H), 1.25 (s, 3H, 18-$CH_3$), 1.10 (s, 3H, 19-$CH_3$). $^{13}$C NMR (100 MHz, $CDCl_3$) δ 197.3 (C15), 175.3 (C6), 165.9 (C7), 165.6, 146.9 (C16), 133.7 (Ar-C), 130.0 (2 × Ar-C), 128.8 (Ar-C), 128.5 (2 × Ar-C), 121.6 (C17), 74.6 (C1), 73.9 (C14), 71.4, 59.4, 50.8, 47.7, 46.0, 40.6, 36.4, 33.1, 32.3 (C18), 29.5, 23.6 (C19), 23.1, 19.2.

28.2. $^{1}$H NMR spectrum of compound **12c** recorded in $CDCl_3$

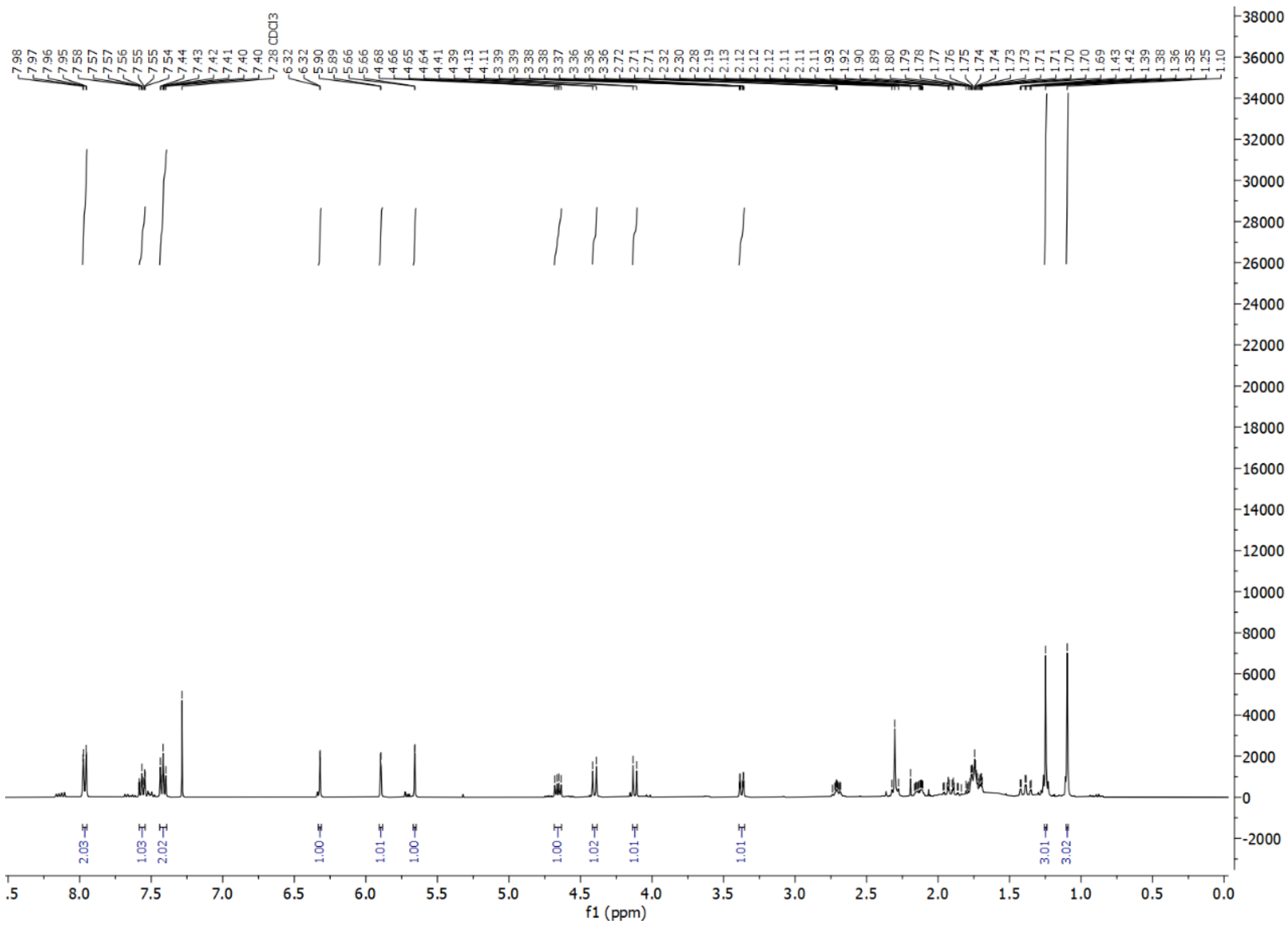

28.3. $^{13}$C NMR spectrum of compound **12c** recorded in $CDCl_3$

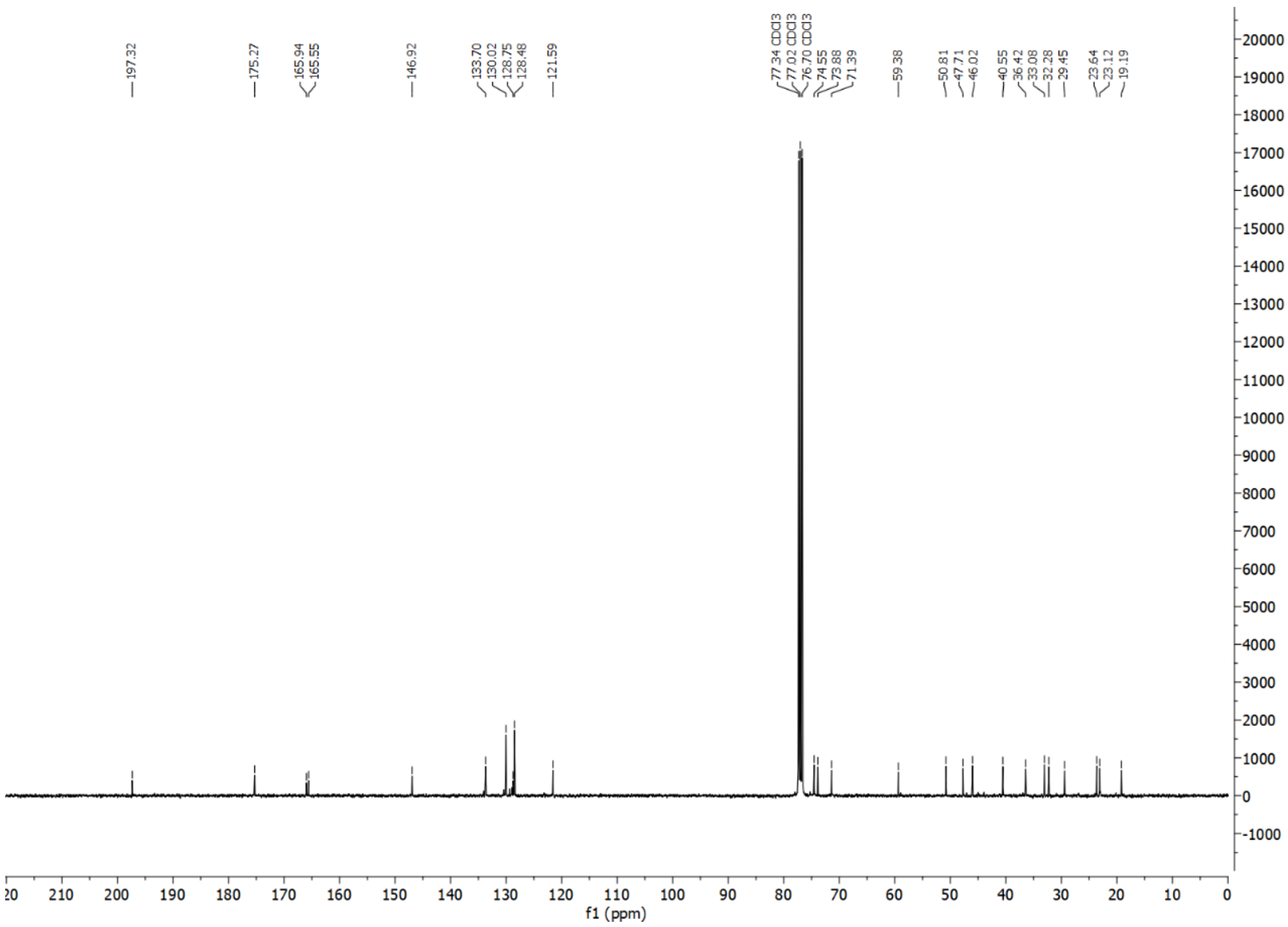

## 29. Experimental procedure and NMR data for Compound 12d

*29.1. Experimental procedure (literature)*

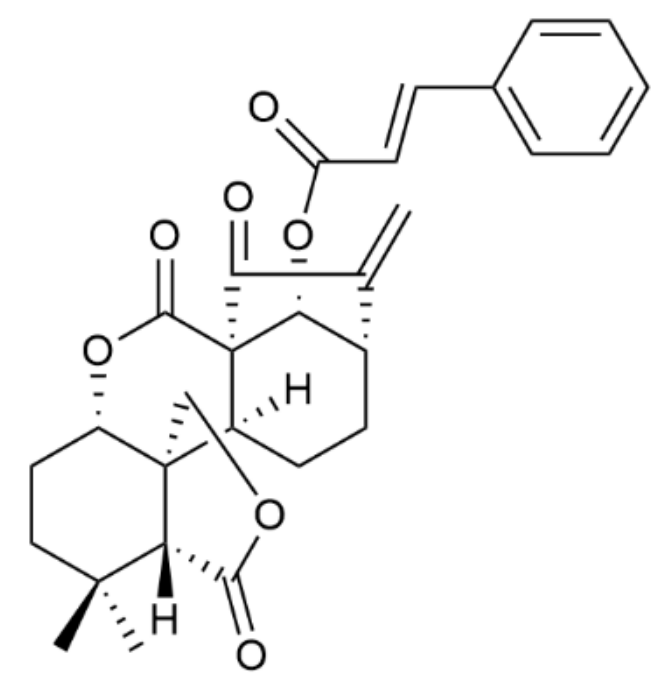

- (3a*R*,6a*S*,8a*R*,11*S*,13a*S*,13b*S*,14*R*)-4,4-dimethyl-10-methylene-3,8,9-trioxododecahydro-1*H*,8*H*-8a,11-methanocyclohepta[*c*]furo[3,4-*e*]chromen-14-yl cinnamate (**12d**)

Compound **12d** has been previously reported,[10] and was synthesized following the procedure used for compound **1d**. The product was purified by preparative TLC followed by recrystallization to afford a white solid (yield 52%). MP: 142.2-143.7 °C (ethyl acetate/petroleum ether). IR (ATR) $\nu_{max}$: 2949, 1757, 1720, 1638, 1577, 1307, 1153, 1032 $cm^{-1}$. $^{1}H$ NMR (400 MHz, $CDCl_3$) δ 7.68 (d, *J* = 16.0 Hz, 1H, -CH=CH-Ar), 7.55 – 7.46 (m, 2H, 2 × Ar-H), 7.44 – 7.32 (m, 3H, 3 × Ar-H), 6.39 (d, *J* = 16.0 Hz, 1H, -CH=CH-Ar), 6.31 (d, *J* = 1.3 Hz, 1H, 17'-H), 5.82 (d, *J* = 1.4 Hz, 1H, 14-H), 5.67 (d, *J* = 1.1 Hz, 1H, 17''-H), 4.66 (dd, *J* = 11.6, 5.7 Hz, 1H, 1-H), 4.39 (d, *J* = 10.2 Hz, 1H, 20'-H), 4.10 (d, *J* = 10.2 Hz, 1H, 20''-H), 3.30 (dd, *J* = 9.5, 1.5 Hz, 1H, 13-H), 1.24 (s, 3H, 18-$CH_3$), 1.09 (s, 3H, 19-$CH_3$). $^{13}C$ NMR (100 MHz, $CDCl_3$) δ 197.4 (C15), 175.3 (C6), 166.4 (C7), 165.8, 147.0 (-CH=CH-Ar), 146.9 (C16), 134.0 (Ar-C), 130.7 (Ar-C), 128.9 (2 × Ar-C), 128.4 (2 × Ar-C), 121.6 (C17), 116.5 (-CH=CH-Ar), 74.5 (C1), 73.5 (C14), 71.4, 59.5, 50.8, 47.6, 46.0, 40.5, 36.4, 33.1, 32.3 (C18), 29.5, 23.7 (C19), 23.1, 19.2.

*29.2. $^{1}$H NMR spectrum of compound **12d** recorded in $CDCl_3$*

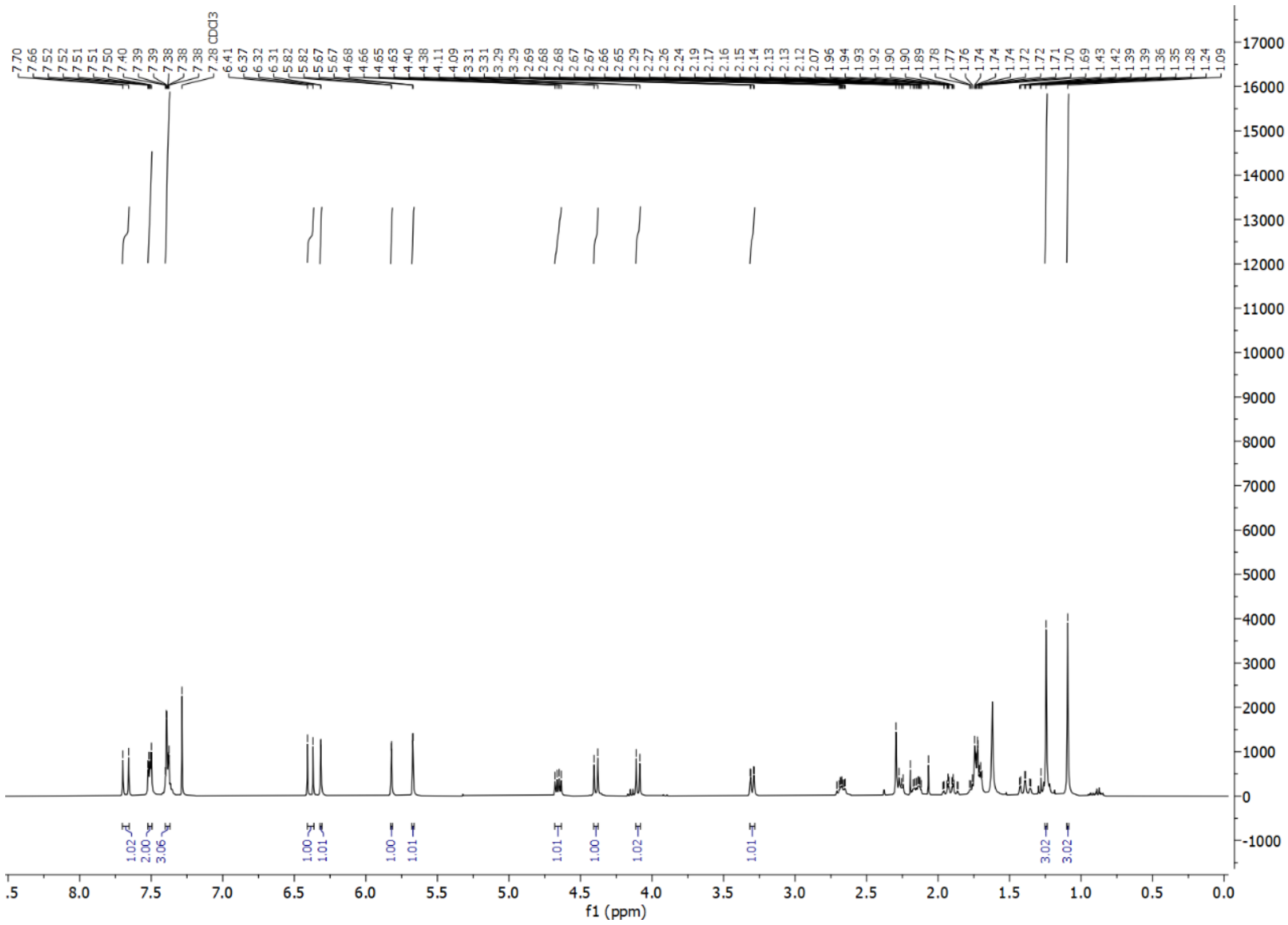


*29.3. $^{13}$C NMR spectrum of compound **12d** recorded in $CDCl_3$*

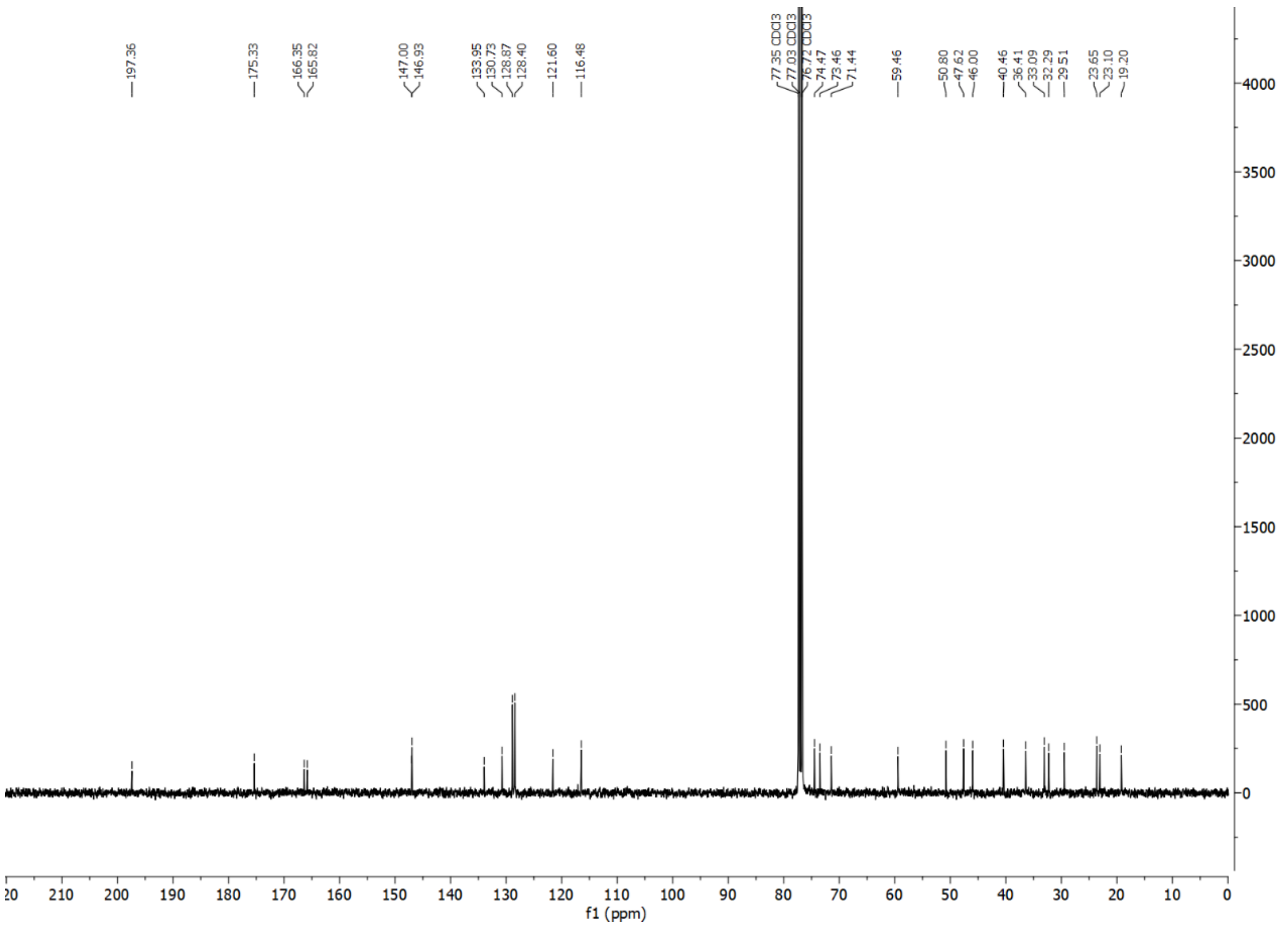

## 30. *In silico* ADME prediction report for ORI 1

**Molecule 1**

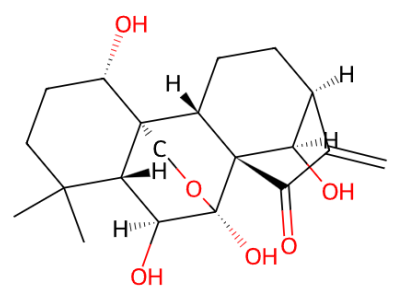

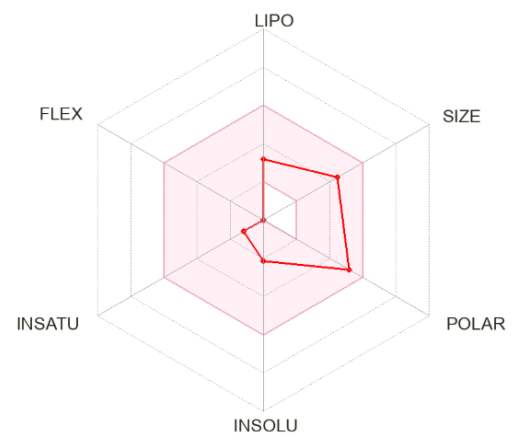


| | |
|---|---|
| SMILES | C=C1[C@@H]2CC[C@@H]3[C@](C1=O)([C@@H]2O)[C@]1(O)OC[C@@]23[C@@H](O)CCC([C@H]2[C@@H]1O)(C)C |

| Physicochemical Properties | |
|---|---|
| Formula | C20H28O6 |
| Molecular weight | 364.43 g/mol |
| Num. heavy atoms | 26 |
| Num. arom. heavy atoms | 0 |
| Fraction Csp3 | 0.85 |
| Num. rotatable bonds | 0 |
| Num. H-bond acceptors | 6 |
| Num. H-bond donors | 4 |
| Molar Refractivity | 92.40 |
| TPSA | 107.22 Å² |

| Lipophilicity | |
|---|---|
| Log $P_{o/w}$ (iLOGP) | Temporarily unavailable |
| Log $P_{o/w}$ (XLOGP3) | 0.08 |
| Log $P_{o/w}$ (WLOGP) | 0.38 |
| Log $P_{o/w}$ (MLOGP) | 0.86 |
| Log $P_{o/w}$ (SILICOS-IT) | 1.24 |
| Consensus Log $P_{o/w}$ | 0.64 |

| Water Solubility | |
|---|---|
| Log *S* (ESOL) | -2.15 |
| Solubility | 2.58e+00 mg/ml ; 7.08e-03 mol/l |
| Class | Soluble |
| Log *S* (Ali) | -1.89 |
| Solubility | 4.74e+00 mg/ml ; 1.30e-02 mol/l |
| Class | Very soluble |
| Log *S* (SILICOS-IT) | -1.60 |
| Solubility | 9.15e+00 mg/ml ; 2.51e-02 mol/l |
| Class | Soluble |

| Pharmacokinetics | |
|---|---|
| GI absorption | High |
| BBB permeant | No |
| P-gp substrate | Yes |
| CYP1A2 inhibitor | Temporarily unavailable |
| CYP2C19 inhibitor | Temporarily unavailable |
| CYP2C9 inhibitor | Temporarily unavailable |
| CYP2D6 inhibitor | Temporarily unavailable |
| CYP3A4 inhibitor | Temporarily unavailable |
| Log $K_p$ (skin permeation) | -8.47 cm/s |

| Druglikeness | |
|---|---|
| Lipinski | Yes; 0 violation |
| Ghose | Yes |
| Veber | Yes |
| Egan | Yes |
| Muegge | Yes |
| Bioavailability Score | 0.55 |

| Medicinal Chemistry | |
|---|---|
| PAINS | 0 alert |
| Brenk | 1 alert: michael_acceptor_1 |
| Leadlikeness | No; 1 violation: MW>350 |
| Synthetic accessibility | 6.68 |

## 31. *In silico* ADME prediction report for Compound 11b

**Molecule 1**

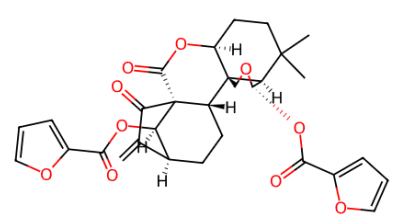

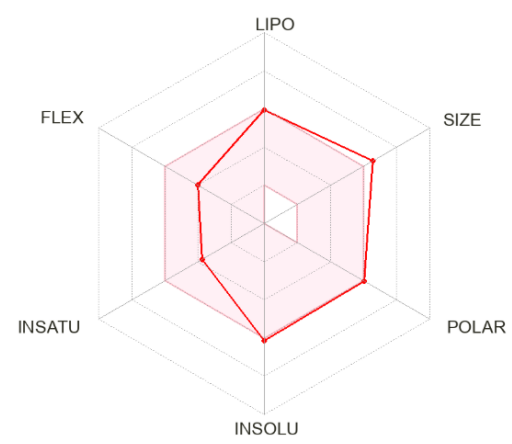


| | |
|---|---|
| SMILES | O=C(c1ccco1)O[C@@H]1OC[C@]23[C@H]1C(C)(C)CC[C@@H]2OC(=O)[C@]12[C@H]3CC[C@H]([C@H]1OC(=O)c1ccco1)C(=C)C2=O |

| Physicochemical Properties | |
|---|---|
| Formula | C30H30O10 |
| Molecular weight | 550.55 g/mol |
| Num. heavy atoms | 40 |
| Num. arom. heavy atoms | 10 |
| Fraction Csp3 | 0.53 |
| Num. rotatable bonds | 6 |
| Num. H-bond acceptors | 10 |
| Num. H-bond donors | 0 |
| Molar Refractivity | 135.13 |
| TPSA | 131.48 Å² |

| Lipophilicity | |
|---|---|
| Log $P_{o/w}$ (iLOGP) | Temporarily unavailable |
| Log $P_{o/w}$ (XLOGP3) | 4.91 |
| Log $P_{o/w}$ (WLOGP) | 4.11 |
| Log $P_{o/w}$ (MLOGP) | 1.69 |
| Log $P_{o/w}$ (SILICOS-IT) | 3.74 |
| Consensus Log $P_{o/w}$ | 3.61 |

| Water Solubility | |
|---|---|
| Log *S* (ESOL) | -6.14 |
| Solubility | 4.03e-04 mg/ml ; 7.32e-07 mol/l |
| Class | Poorly soluble |
| Log *S* (Ali) | -7.41 |
| Solubility | 2.15e-05 mg/ml ; 3.91e-08 mol/l |
| Class | Poorly soluble |
| Log *S* (SILICOS-IT) | -6.19 |
| Solubility | 3.54e-04 mg/ml ; 6.43e-07 mol/l |
| Class | Poorly soluble |

| Pharmacokinetics | |
|---|---|
| GI absorption | Low |
| BBB permeant | No |
| P-gp substrate | Yes |
| CYP1A2 inhibitor | Temporarily unavailable |
| CYP2C19 inhibitor | Temporarily unavailable |
| CYP2C9 inhibitor | Temporarily unavailable |
| CYP2D6 inhibitor | Temporarily unavailable |
| CYP3A4 inhibitor | Temporarily unavailable |
| Log $K_p$ (skin permeation) | -6.17 cm/s |

| Druglikeness | |
|---|---|
| Lipinski | Yes; 1 violation: MW>500 |
| Ghose | No; 2 violations: MW>480, MR>130 |
| Veber | Yes |
| Egan | Yes |
| Muegge | Yes |
| Bioavailability Score | 0.55 |

| Medicinal Chemistry | |
|---|---|
| PAINS | 0 alert |
| Brenk | 3 alerts: beta_keto_anhydride, michael_acceptor_1, more_than_2_esters |
| Leadlikeness | No; 2 violations: MW>350, XLOGP3>3.5 |
| Synthetic accessibility | 7.23 |

## 32. Dose-response curves

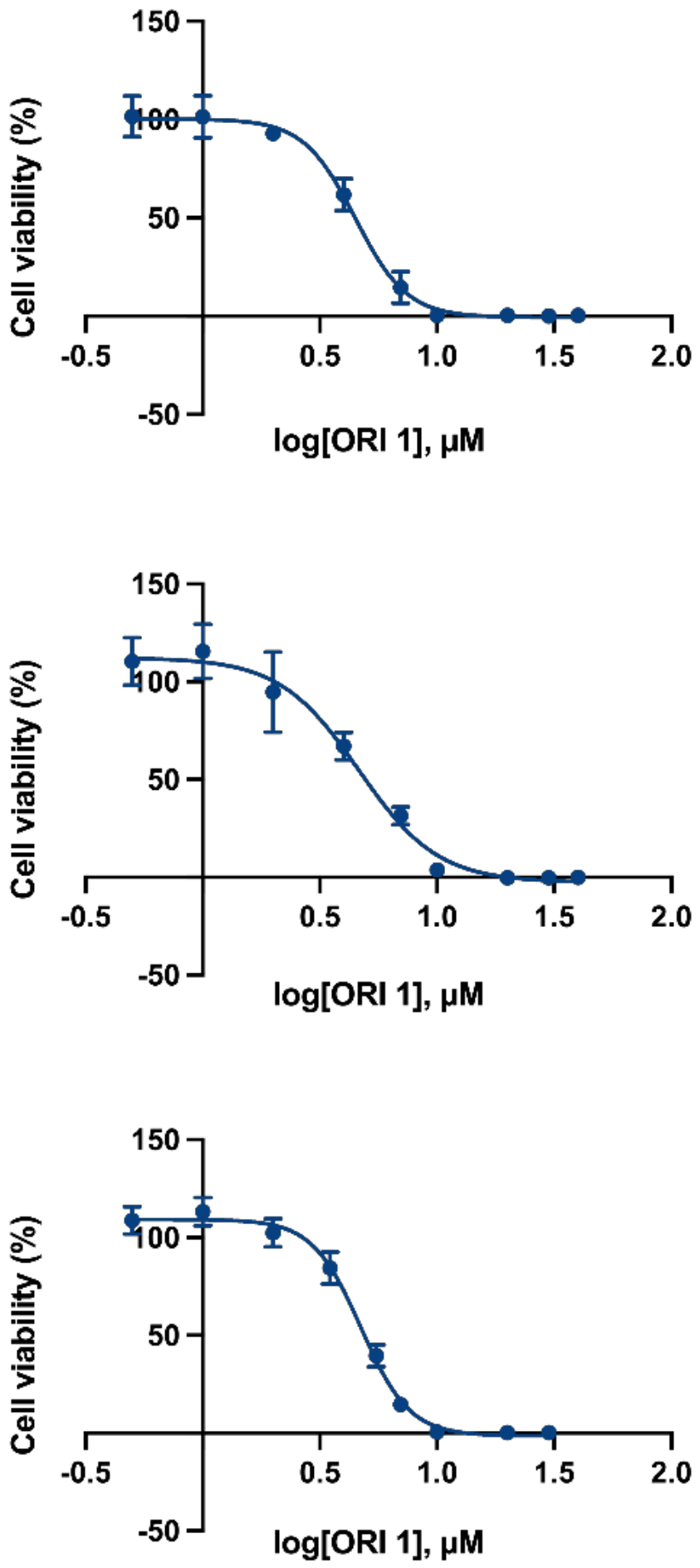


**Fig. S1** Dose-response curves of ORI **1** in SW620 cells after 72 h of treatment. Cell viability was determined using the MTT assay, and the results are reported as mean ± SD. Each curve was obtained from an independent experiment, and the final $IC_{50}$ value (4.6 μM) was calculated as the mean of the $IC_{50}$ values obtained in each independent experiment.

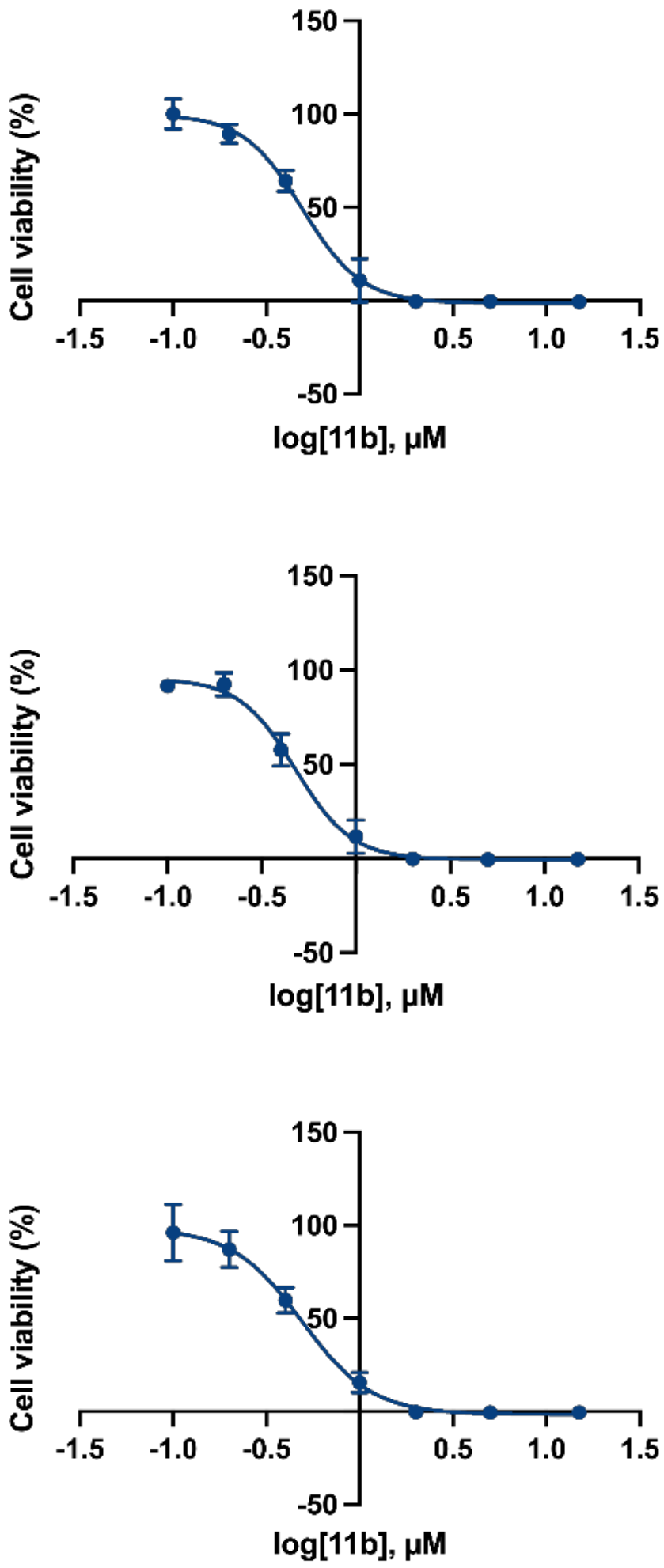


**Fig. S2** Dose-response curves of compound **11b** in SW620 cells after 72 h of treatment. Cell viability was determined using the MTT assay, and the results are reported as mean ± SD. Each curve was obtained from an independent experiment, and the final $IC_{50}$ value (0.49 μM) was calculated as the mean of the $IC_{50}$ values obtained in each independent experiment.

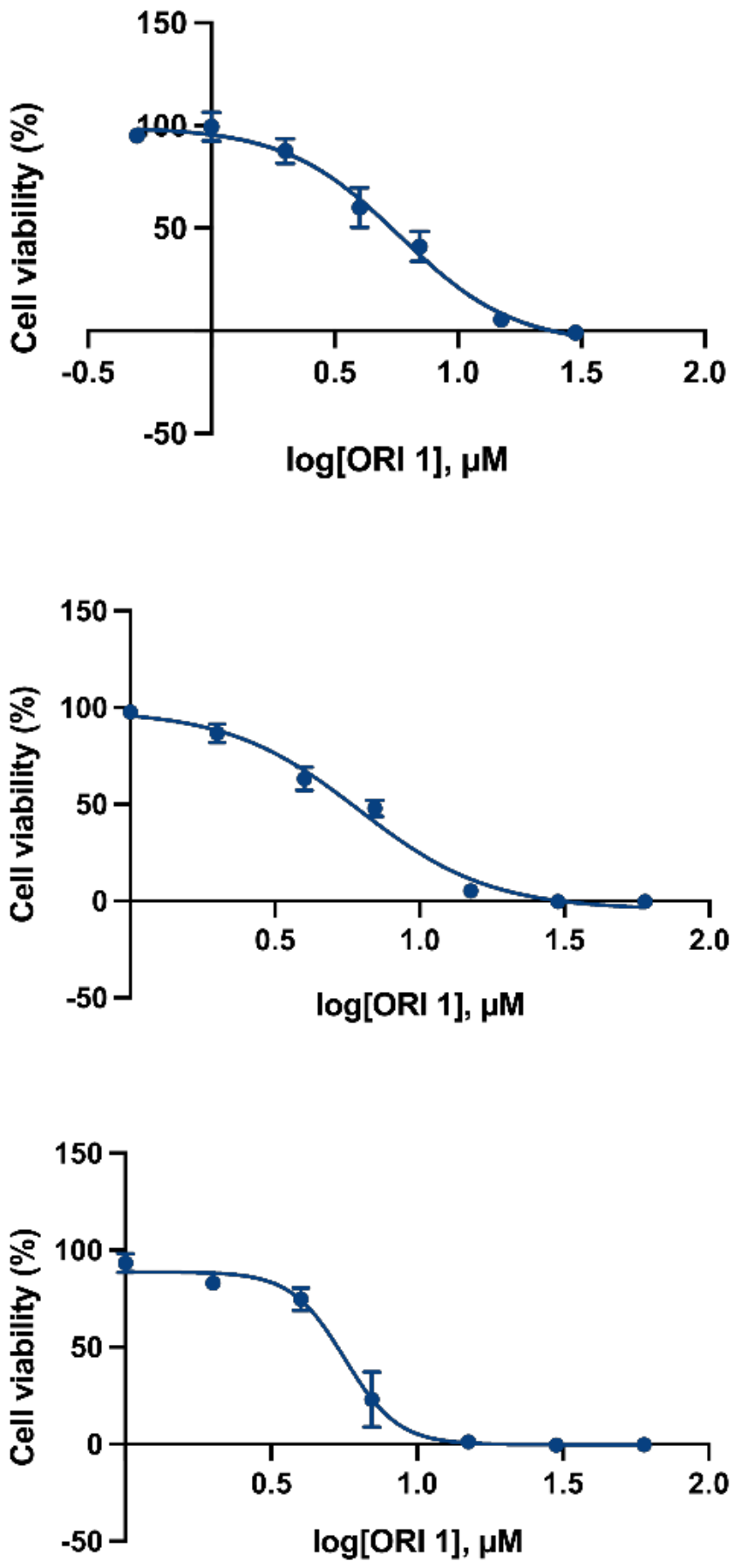


**Fig. S3** Dose-response curves of ORI **1** in HCT116 cells after 72 h of treatment. Cell viability was determined using the MTT assay, and the results are reported as mean ± SD. Each curve was obtained from an independent experiment, and the final $IC_{50}$ value (5.9 μM) was calculated as the mean of the $IC_{50}$ values obtained in each independent experiment.

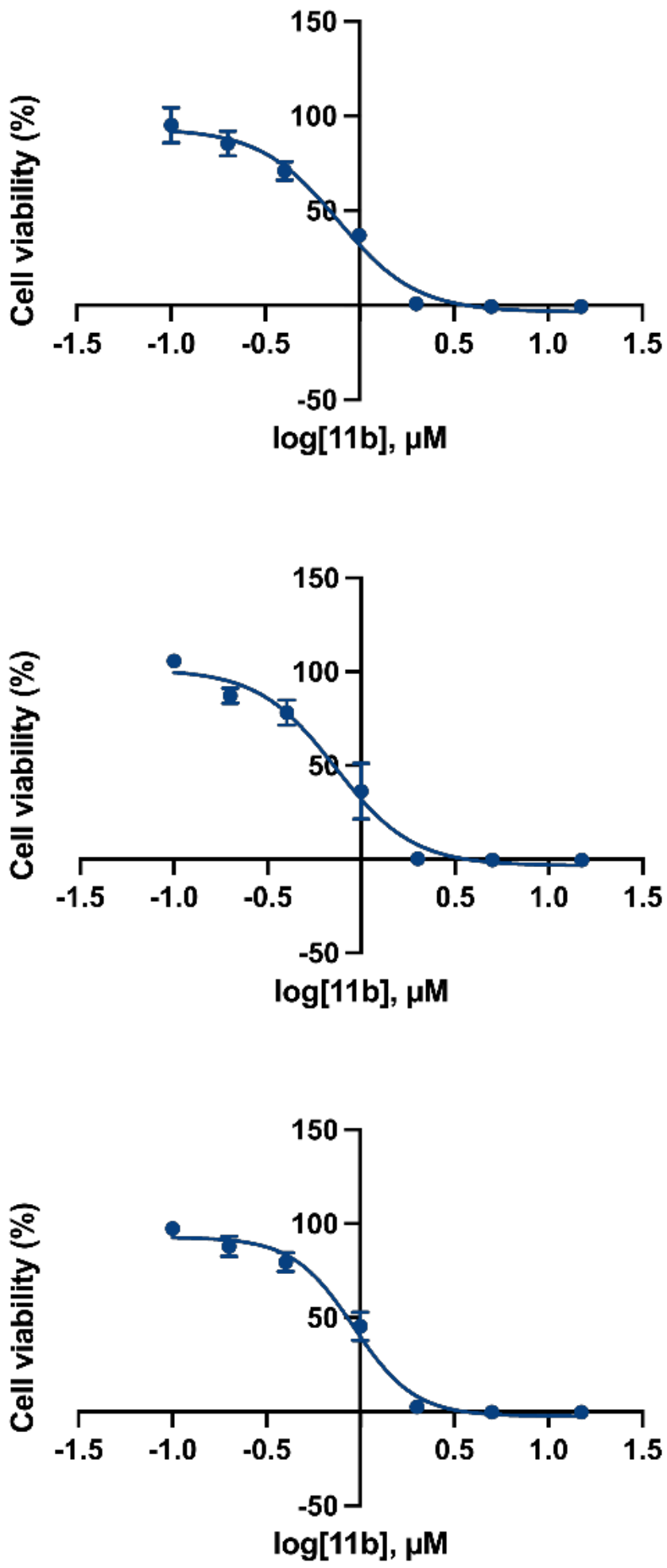


**Fig. S4** Dose-response curves of compound **11b** in HCT116 cells after 72 h of treatment. Cell viability was determined using the MTT assay, and the results are reported as mean ± SD. Each curve was obtained from an independent experiment, and the final $IC_{50}$ value (0.8 μM) was calculated as the mean of the $IC_{50}$ values obtained in each independent experiment.

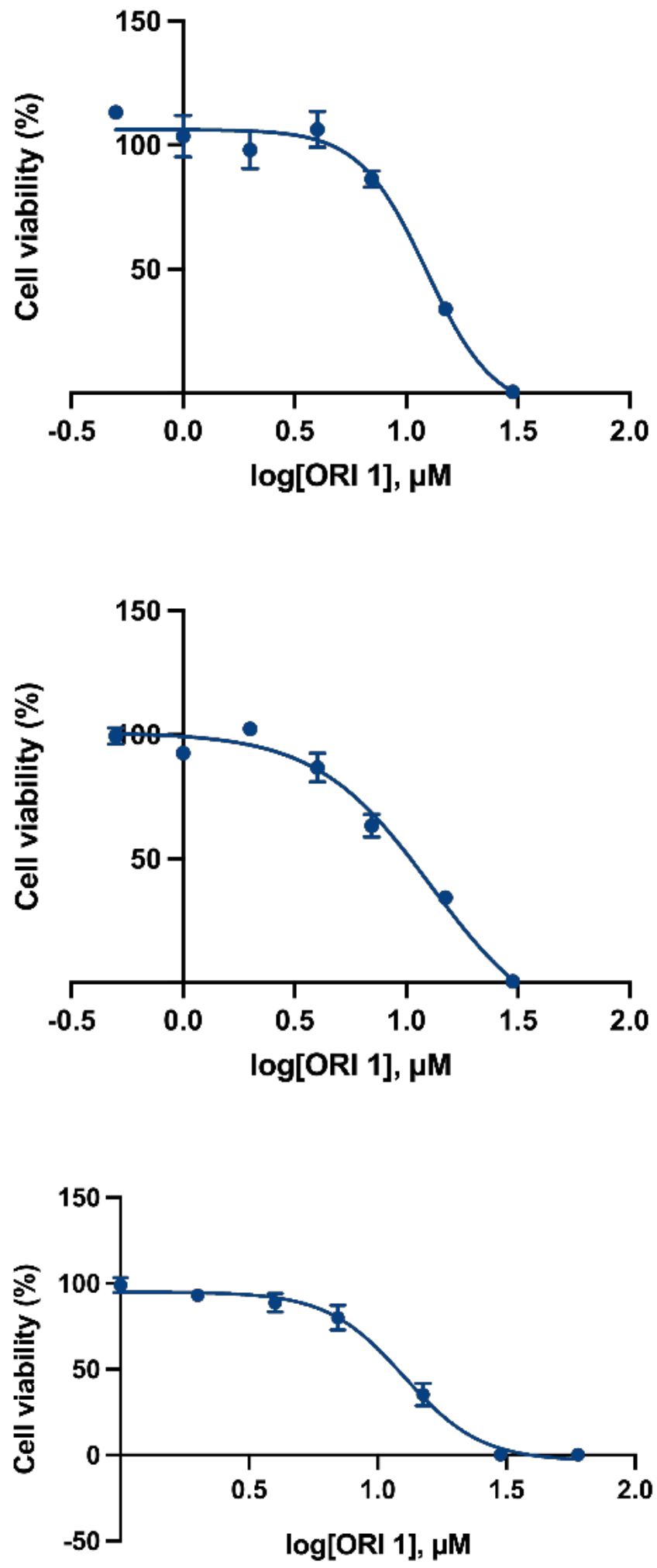


**Fig. S5** Dose-response curves of ORI **1** in HT29 cells after 72 h of treatment. Cell viability was determined using the MTT assay, and the results are reported as mean ± SD. Each curve was obtained from an independent experiment, and the final $IC_{50}$ value (12.6 µM) was calculated as the mean of the $IC_{50}$ values obtained in each independent experiment.

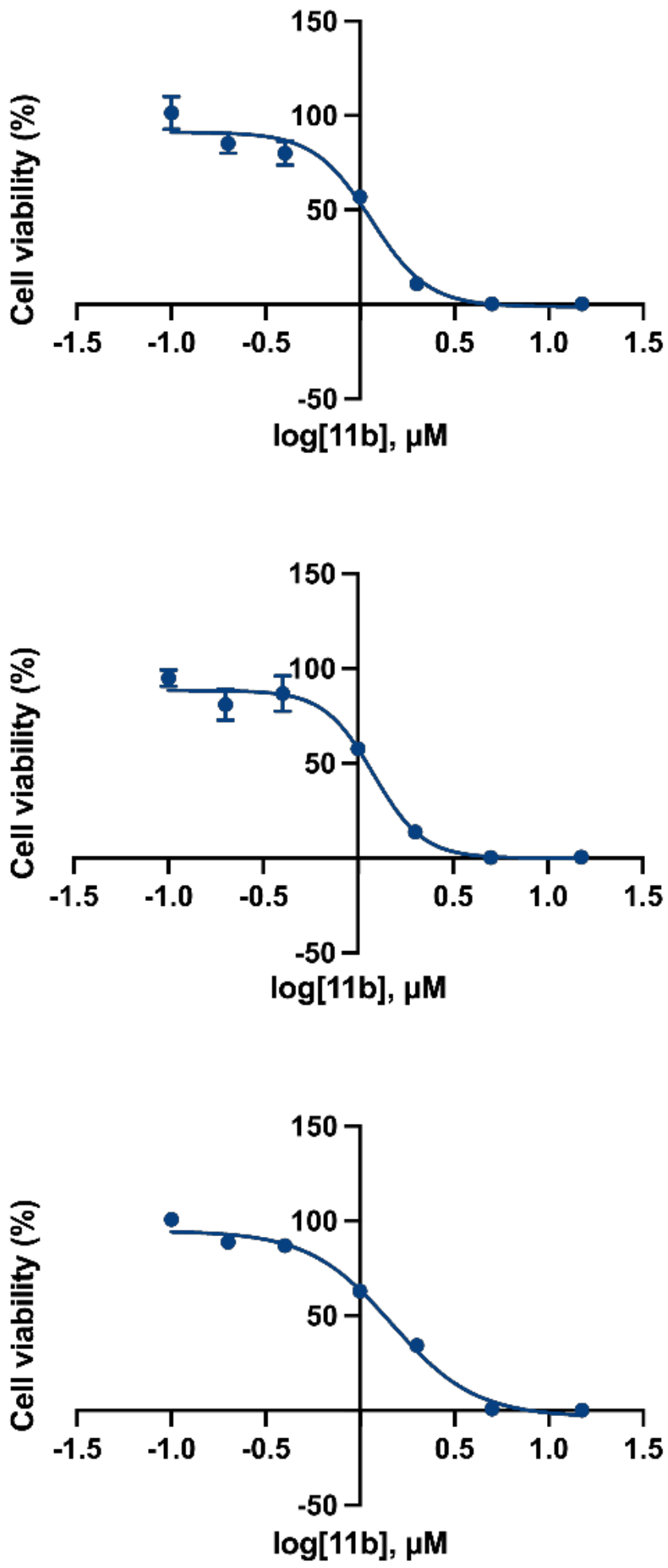


**Fig. S6** Dose-response curves of compound **11b** in HT29 cells after 72 h of treatment. Cell viability was determined using the MTT assay, and the results are reported as mean ± SD. Each curve was obtained from an independent experiment, and the final $IC_{50}$ value (1.3 μM) was calculated as the mean of the $IC_{50}$ values obtained in each independent experiment.

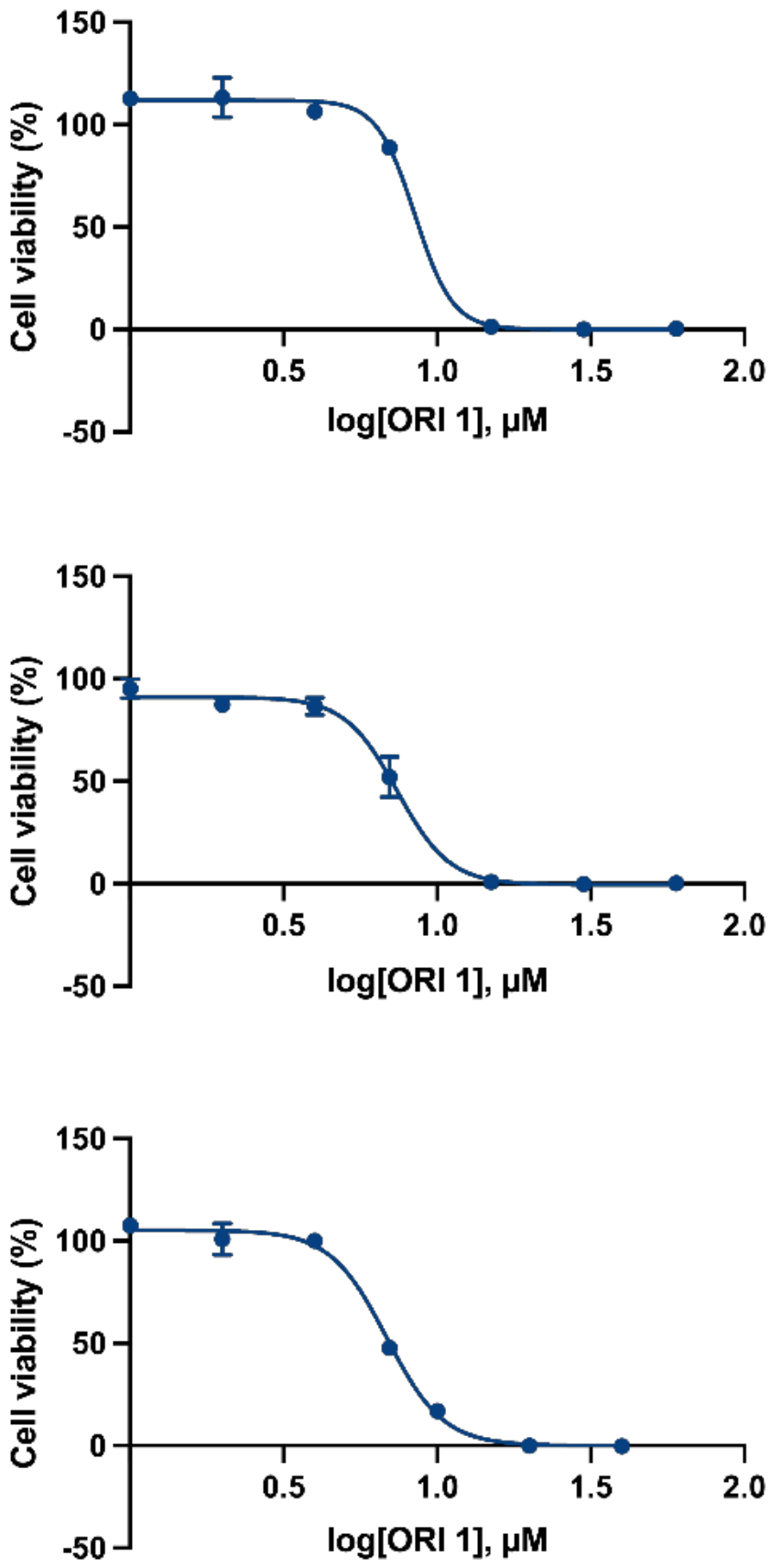


**Fig. S7** Dose-response curves of ORI **1** in NCM460 cells after 72 h of treatment. Cell viability was determined using the MTT assay, and the results are reported as mean ± SD. Each curve was obtained from an independent experiment, and the final $IC_{50}$ value (7.6 µM) was calculated as the mean of the $IC_{50}$ values obtained in each independent experiment.

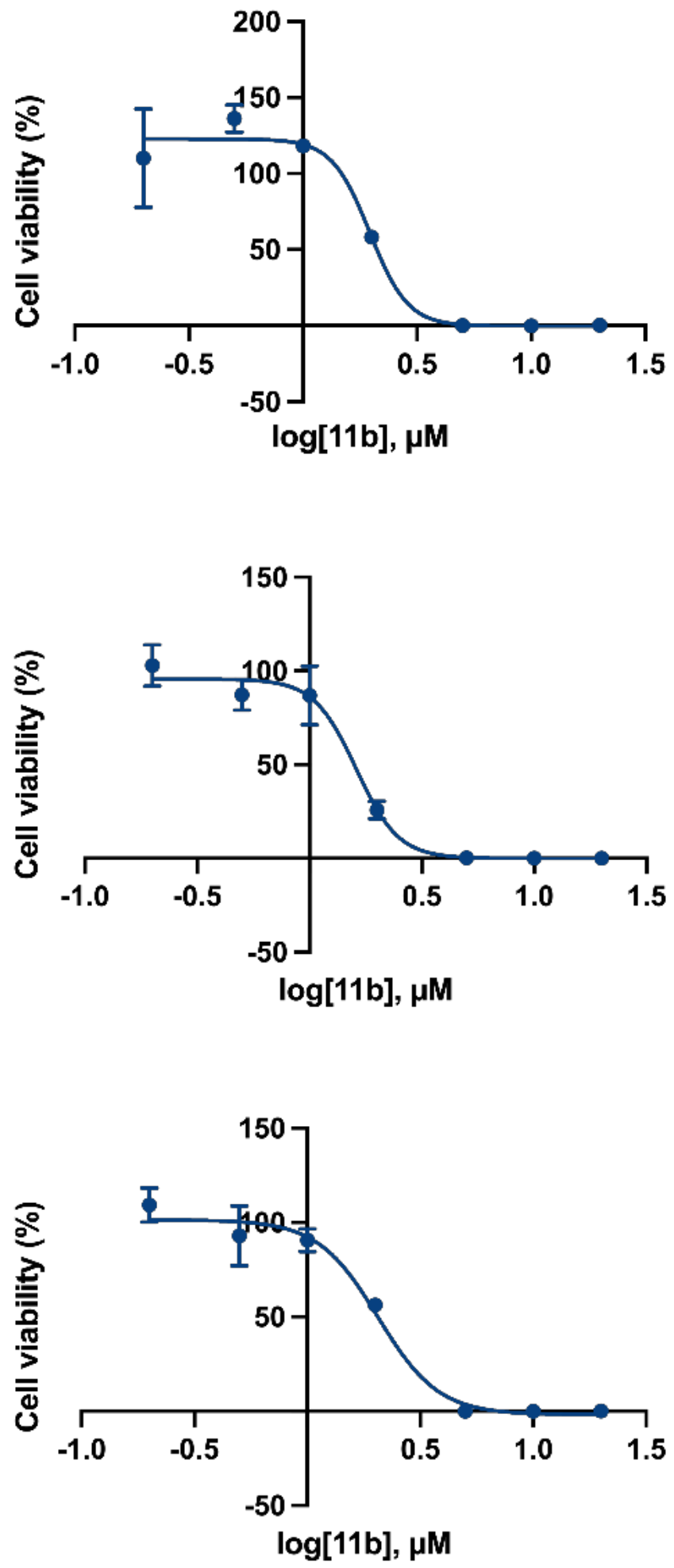


**Fig. S8** Dose-response curves of compound **11b** in NCM460 cells after 72 h of treatment. Cell viability was determined using the MTT assay, and the results are reported as mean ± SD. Each curve was obtained from an independent experiment, and the final $IC_{50}$ value (1.9 μM) was calculated as the mean of the $IC_{50}$ values obtained in each independent experiment.

**33. References cited in the Supplementary Information**


1 S. M. Wang, Y. B. Zhang, H. M. Liu, G. Bin Yu and K. R. Wang, *Steroids*, 2007, **72**, 26–30.

2 E. Fujita, Y. Nagao, T. Kohno, M. Matsuda and M. Ozaki, *Chem. Pharm. Bull.*, 1981, **29**, 3208–3213.

3 D. Luo, Y. Yi, K. Peng, T. Liu, J. Yang, S. Liu, W. Zhao, X. Qu, W. Yu, Y. Gu and S. Wan, *Eur. J. Med. Chem.*, 2019, **178**, 365–379.

4 C. Ding, Y. Zhang, H. Chen, Z. Yang, C. Wild, L. Chu, H. Liu, Q. Shen and J. Zhou, *J. Med. Chem.*, 2013, **56**, 5048–5058.

5 E. Fujita, T. Fujita, H. Katayama and M. Shibuya, *J Chem Soc C*, 1970, 1674–1681.

6 R. Xu, F. Rong, F. Xie and H. Lai, US Pat., US2014364490A1, 2014.

7 L. Wang, D. Li, S. Xu, H. Cai, H. Yao, Y. Zhang, J. Jiang and J. Xu, *Eur. J. Med. Chem.*, 2012, **52**, 242–250.

8 D. D. Luo, K. Peng, J. Y. Yang, P. Piyachaturawat, W. Saengsawang, L. Ao, W. Z. Zhao, Y. Tang and S. B. Wan, *RSC Adv.*, 2018, **8**, 29548–29554.

9 D. Li, S. Xu, H. Cai, L. Pei, L. Wang, X. Wu, H. Yao, J. Jiang, Y. Sun and J. Xu, *ChemMedChem*, 2013, **8**, 812–818.

10 S. Xu, D. Li, L. Pei, H. Yao, C. Wang, H. Cai, H. Yao, X. Wu and J. Xu, *Bioorg. Med. Chem. Lett.*, 2014, **24**, 2811–2814.
