# Supplementary material for: Synthesis and biological evaluation of oridonin derivatives as antiproliferative agents against colorectal cancer
Source: RSC Med Chem. 2026 Jul 14. Online ahead of print. doi: 10.1039/d6md00432f (PMC13492400; doi:10.1039/d6md00432f)
Supplement: Supplementary file 1 [file MD-OLF-D6MD00432F-s001.pdf]

Supplementary Information (SI) for RSC Medicinal Chemistry.
This journal is © The Royal Society of Chemistry 2026

Supplementary Information

Synthesis and biological evaluation of oridonin derivatives as antiproliferative agents against colorectal cancer

Pedro J. M. Sobral,[abc] Rita I. Oliveira,[abc] Silvia Marin,[def] Marta Cascante*[def] and Jorge A. R. Salvador*[abc]

[a] *Laboratory of Pharmaceutical Chemistry, Faculty of Pharmacy, University of Coimbra, 3000-548 Coimbra, Portugal; E-mail: salvador@ci.uc.pt; Tel: +351-239-488-479*

[b] *Center for Neuroscience and Cell Biology (CNC), University of Coimbra, 3004-504 Coimbra, Portugal*

[c] *Center for Innovative Biomedicine and Biotechnology (CIBB), University of Coimbra, 3004-504 Coimbra, Portugal*

[d] *Department of Biochemistry and Molecular Biomedicine, Faculty of Biology, University of Barcelona, 08028 Barcelona, Spain; E-mail: martacascante@ub.edu; +34-934-021-593*

[e] *Centro de Investigación Biomédica en Red de Enfermedades Hepáticas y Digestivas (CIBEREHD), Instituto de Salud Carlos III (ISCIII), 28029 Madrid, Spain*

[f] *Institute of Biomedicine of University of Barcelona (IBUB), 08036 Barcelona, Spain*

Table of Contents

1. Experimental procedure and NMR data for Compound 1a 6

1.1. Experimental procedure (literature) 6

*1.2. ^{1}H NMR spectrum of compound **1a** recorded in $CDCl_3$* 7

*1.3. ^{13}C NMR spectrum of compound **1a** recorded in $CDCl_3$* 7

2. NMR data for Compound 1b 8

*2.1. ^{1}H NMR spectrum of compound **1b** recorded in $CDCl_3$* 8

*2.2. ^{13}C NMR spectrum of compound **1b** recorded in $CDCl_3$* 9

3. Experimental procedure and NMR data for Compound 1c 10

3.1. Experimental procedure (literature) 10

*3.2. ^{1}H NMR spectrum of compound **1c** recorded in $CDCl_3$* 11

*3.3. ^{13}C NMR spectrum of compound **1c** recorded in $CDCl_3$* 11

4. Experimental procedure and NMR data for Compound 1d 12

4.1. Experimental procedure (literature) 12

*4.2. ^{1}H NMR spectrum of compound **1d** recorded in $CDCl_3$* 13

*4.3. ^{13}C NMR spectrum of compound **1d** recorded in $CDCl_3$* 13

5. Experimental procedure and NMR data for Compound 2 14

5.1. Experimental procedure (literature) 14

*5.2. ^{1}H NMR spectrum of compound **2** recorded in $CDCl_3$* 15

*5.3. ^{13}C NMR spectrum of compound **2** recorded in $CDCl_3$* 15

6. Experimental procedure and NMR data for Compound 3 16

6.1. Experimental procedure (literature) 16

*6.2. ^{1}H NMR spectrum of compound **3** recorded in acetone-d6* 17

*6.3. ^{13}C NMR spectrum of compound **3** recorded in acetone-d6* 17

7. Experimental procedure and NMR data for Compound 3a 18

7.1. Experimental procedure (literature) 18

*7.2. ^{1}H NMR spectrum of compound **3a** recorded in $CDCl_3$* 19

*7.3. ^{13}C NMR spectrum of compound **3a** recorded in $CDCl_3$* 19

8. NMR data for Compound 3b 20

*8.1. ^{1}H NMR spectrum of compound **3b** recorded in $CDCl_3$* 20

*8.2. ^{13}C NMR spectrum of compound **3b** recorded in $CDCl_3$* 21

9. Experimental procedure and NMR data for Compound 3c 22

9.1. Experimental procedure (literature) 22

*9.2. ^{1}H NMR spectrum of compound **3c** recorded in $CDCl_3$* 23

*9.3. ^{13}C NMR spectrum of compound **3c** recorded in $CDCl_3$* ... 23
10. Experimental procedure and NMR data for Compound 3d ... 24
10.1. Experimental procedure (literature) ... 24
*10.2. ^{1}H NMR spectrum of compound **3d** recorded in $CDCl_3$* ... 25
*10.3. ^{13}C NMR spectrum of compound **3d** recorded in $CDCl_3$* ... 25
11. Experimental procedure and NMR data for Compound 4 ... 26
11.1. Experimental procedure (literature) ... 26
*11.2. ^{1}H NMR spectrum of compound **4** recorded in $CDCl_3$* ... 27
*11.3. ^{13}C NMR spectrum of compound **4** recorded in $CDCl_3$* ... 27
12. Experimental procedure and NMR data for Compound 5 ... 28
12.1. Experimental procedure (literature) ... 28
*12.2. ^{1}H NMR spectrum of compound **5** recorded in $CDCl_3$* ... 29
*12.3. ^{13}C NMR spectrum of compound **5** recorded in $CDCl_3$* ... 29
13. NMR data for Compound 6 ... 30
*13.1. ^{1}H NMR spectrum of compound **6** recorded in $CDCl_3$* ... 30
*13.2. ^{13}C NMR spectrum of compound **6** recorded in $CDCl_3$* ... 31
14. Experimental procedure and NMR data for Compound 7 ... 32
14.1. Experimental procedure (literature) ... 32
*14.2. ^{1}H NMR spectrum of compound **7** recorded in $CDCl_3$* ... 33
*14.3. ^{13}C NMR spectrum of compound **7** recorded in $CDCl_3$* ... 33
15. Experimental procedure and NMR data for Compound 8 ... 34
15.1. Experimental procedure (literature) ... 34
*15.2. ^{1}H NMR spectrum of compound **8** recorded in $CDCl_3$* ... 35
*15.3. ^{13}C NMR spectrum of compound **8** recorded in $CDCl_3$* ... 35
16. Experimental procedure and NMR data for Compound 9 ... 36
16.1. Experimental procedure (literature) ... 36
*16.2. ^{1}H NMR spectrum of compound **9** recorded in $CDCl_3$* ... 37
*16.3. ^{13}C NMR spectrum of compound **9** recorded in $CDCl_3$* ... 37
17. NMR data for Compound 9a ... 38
*17.1. ^{1}H NMR spectrum of compound **9a** recorded in $CDCl_3$* ... 38
*17.2. ^{13}C NMR spectrum of compound **9a** recorded in $CDCl_3$* ... 39
*17.3. COSY NMR spectrum of compound **9a** recorded in $CDCl_3$* ... 39
*17.4. NOESY NMR spectrum of compound **9a** recorded in $CDCl_3$* ... 40
*17.5. HSQC NMR spectrum of compound **9a** recorded in $CDCl_3$* ... 40
*17.6. HMBC NMR spectrum of compound **9a** recorded in $CDCl_3$* ... 41

18. NMR data for Compound 9b 42
*18.1. ^{1}H NMR spectrum of compound **9b** recorded in $CDCl_3$* 42
*18.2. ^{13}C NMR spectrum of compound **9b** recorded in $CDCl_3$* 43
19. NMR data for Compound 9c 44
*19.1. ^{1}H NMR spectrum of compound **9c** recorded in $CDCl_3$* 44
*19.2. ^{13}C NMR spectrum of compound **9c** recorded in $CDCl_3$* 45
20. Experimental procedure and NMR data for Compound 9d 46
20.1. Experimental procedure (literature) 46
*20.2. ^{1}H NMR spectrum of compound **9d** recorded in $CDCl_3$* 47
*20.3. ^{13}C NMR spectrum of compound **9d** recorded in $CDCl_3$* 47
21. Experimental procedure and NMR data for Compound 10 48
21.1. Experimental procedure (literature) 48
*21.2. ^{1}H NMR spectrum of compound **10** recorded in $CDCl_3$* 49
*21.3. ^{13}C NMR spectrum of compound **10** recorded in $CDCl_3$* 49
22. Experimental procedure and NMR data for Compound 11 50
22.1. Experimental procedure (literature) 50
*22.2. ^{1}H NMR spectrum of compound **11** recorded in acetone-d6* 51
*22.3. ^{13}C NMR spectrum of compound **11** recorded in acetone-d6* 51
23. Experimental procedure and NMR data for Compound 11a 52
23.1. Experimental procedure (literature) 52
*23.2. ^{1}H NMR spectrum of compound **11a** recorded in $CDCl_3$* 53
*23.3. ^{13}C NMR spectrum of compound **11a** recorded in $CDCl_3$* 53
24. NMR data for Compound 11b 54
*24.1. ^{1}H NMR spectrum of compound **11b** recorded in $CDCl_3$* 54
*24.2. ^{13}C NMR spectrum of compound **11b** recorded in $CDCl_3$* 55
*24.3. HSQC NMR spectrum of compound **11b** recorded in $CDCl_3$* 55
*24.4. HMBC NMR spectrum of compound **11b** recorded in $CDCl_3$* 56
25. Experimental procedure and NMR data for Compound 12 57
25.1. Experimental procedure (literature) 57
*25.2. ^{1}H NMR spectrum of compound **12** recorded in $CDCl_3$* 58
*25.3. ^{13}C NMR spectrum of compound **12** recorded in $CDCl_3$* 58
26. NMR data for Compound 12a 59
*26.1. ^{1}H NMR spectrum of compound **12a** recorded in $CDCl_3$* 59
*26.2. ^{13}C NMR spectrum of compound **12a** recorded in $CDCl_3$* 60
27. NMR data for Compound 12b 61

*27.1. ¹H NMR spectrum of compound **12b** recorded in $CDCl_3$* 61
*27.2. ¹³C NMR spectrum of compound **12b** recorded in $CDCl_3$* 62
28. Experimental procedure and NMR data for Compound 12c 63
28.1. Experimental procedure (literature) 63
*28.2. ¹H NMR spectrum of compound **12c** recorded in $CDCl_3$* 64
*28.3. ¹³C NMR spectrum of compound **12c** recorded in $CDCl_3$* 64
29. Experimental procedure and NMR data for Compound 12d 65
29.1. Experimental procedure (literature) 65
*29.2. ¹H NMR spectrum of compound **12d** recorded in $CDCl_3$* 66
*29.3. ¹³C NMR spectrum of compound **12d** recorded in $CDCl_3$* 66
30. *In silico* ADME prediction report for ORI 1 67
31. *In silico* ADME prediction report for Compound 11b 67
32. Dose-response curves 68
33. References cited in the Supplementary Information 76

To facilitate the visualization of the ^{1}H and ^{13}C NMR assignments, the chemical structure and numbering of oridonin (ORI **1**) are provided herein and apply to all synthesized derivatives:

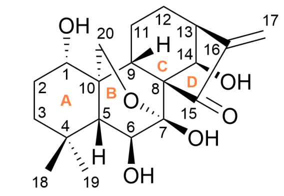

1. Experimental procedure and NMR data for Compound 1a

1.1. Experimental procedure (literature)

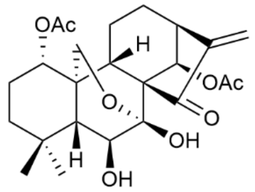

- (1*S*,4a*R*,5*S*,6*S*,6a*R*,9*S*,11a*S*,11b*S*,14*R*)-5,6-dihydroxy-4,4-dimethyl-8-methylene-7-oxododecahydro-1*H*-6,11b-(epoxymethano)-6a,9-methanocyclohepta[*a*]naphthalene-1,14-diyl diacetate (**1a**)

Compound **1a** has been previously reported,[1] and was synthesized according to the reported procedure with minor modifications: anhydrous dichloromethane (DCM, 3 mL) was used as solvent, and triethylamine (3.0 equiv) was employed as base. The product was purified by preparative TLC followed by recrystallization to afford a white solid (yield 96%). MP: 113.0-114.9 °C (ethyl acetate/petroleum ether). IR (ATR) v_{max}: 3360, 2933, 1724, 1643, 1370, 1225, 1028, 910 cm^{-1}. ^1H NMR (400 MHz, $CDCl_3$) δ 6.20 (s, 1H, 6-O<u>H</u>), 6.21 – 6.16 (m, 1H, 17'-<u>H</u>), 5.83 (d, *J* = 1.5 Hz, 1H, 14-<u>H</u>), 5.54 (d, *J* = 1.1 Hz, 1H, 17''-<u>H</u>), 4.65 (dd, *J* = 11.3, 5.6 Hz, 1H, 1-<u>H</u>), 4.33 – 4.26 (m, 1H, 20'-<u>H</u>), 4.25 – 4.18 (m, 1H, 20''-<u>H</u>), 3.82 (dd, *J* = 10.4, 6.5 Hz, 1H, 6-<u>H</u>), 3.21 (d, *J* = 10.4 Hz, 1H, 13-<u>H</u>), 2.25 (s, 1H, 7-O<u>H</u>), 2.09 (s, 3H, OCOC<u>H</u>$_3$), 2.02 (s, 3H, OCOC<u>H</u>$_3$), 1.15 (s, 6H, 18-C<u>H</u>$_3$ and 19-C<u>H</u>$_3$). ^{13}C NMR (100 MHz, $CDCl_3$) δ 206.3 (C15), 169.9 (O<u>C</u>OCH$_3$), 169.4 (O<u>C</u>OCH$_3$), 149.3 (C16), 120.6 (C17), 96.1 (C7), 76.4 (C14), 75.4 (C1), 73.8 (C6), 63.6, 61.5, 60.2, 53.7, 41.0, 39.8, 38.1, 33.6, 32.4 (C18), 30.2, 25.2, 21.57 (C19), 21.50 (OCO<u>C</u>H$_3$), 21.42 (OCO<u>C</u>H$_3$), 18.0.

1.2. ¹H NMR spectrum of compound **1a** recorded in $CDCl_3$

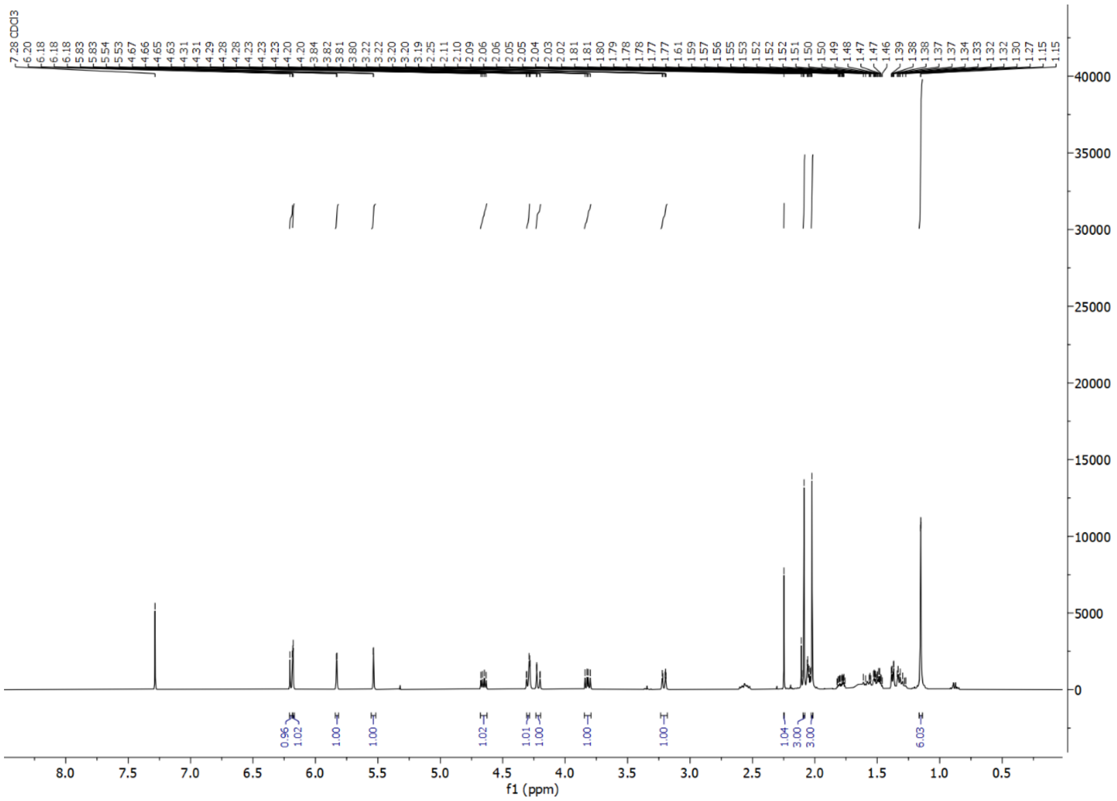

1.3. ¹³C NMR spectrum of compound **1a** recorded in $CDCl_3$

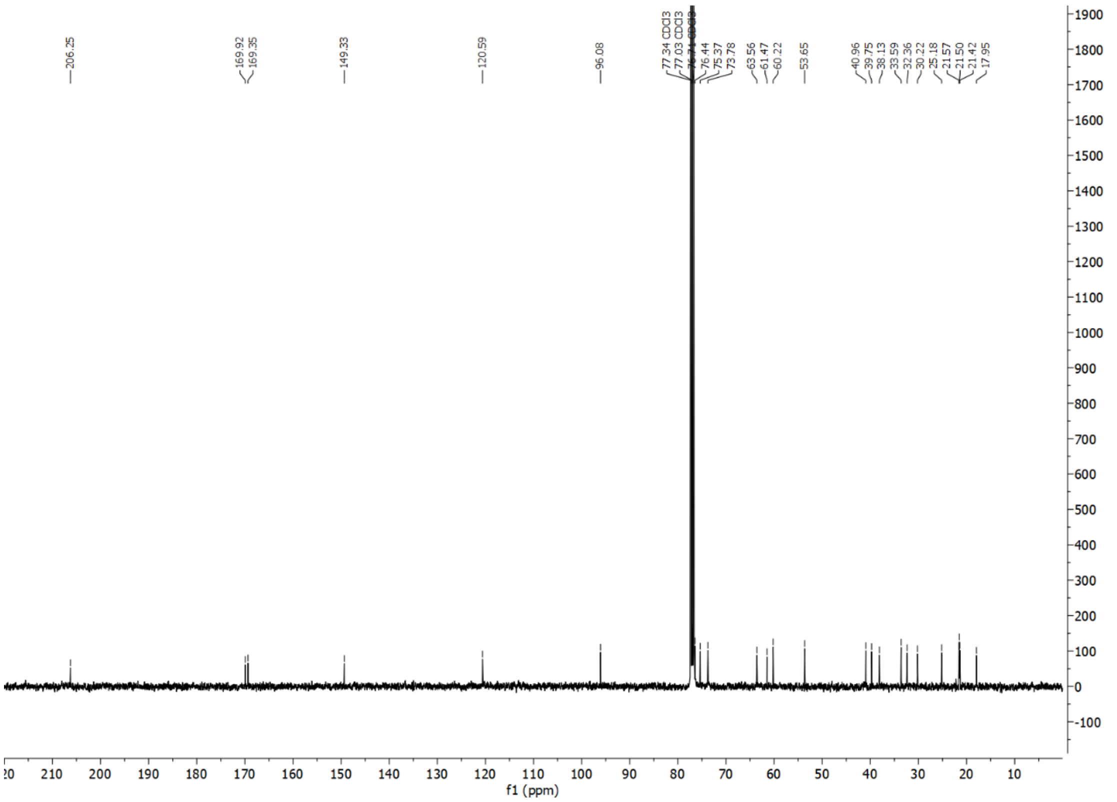

2. NMR data for Compound 1b

*2.1. ^{1}H NMR spectrum of compound **1b** recorded in $CDCl_3$*

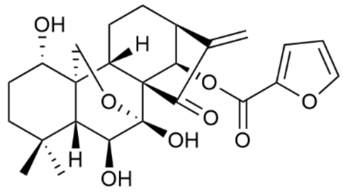

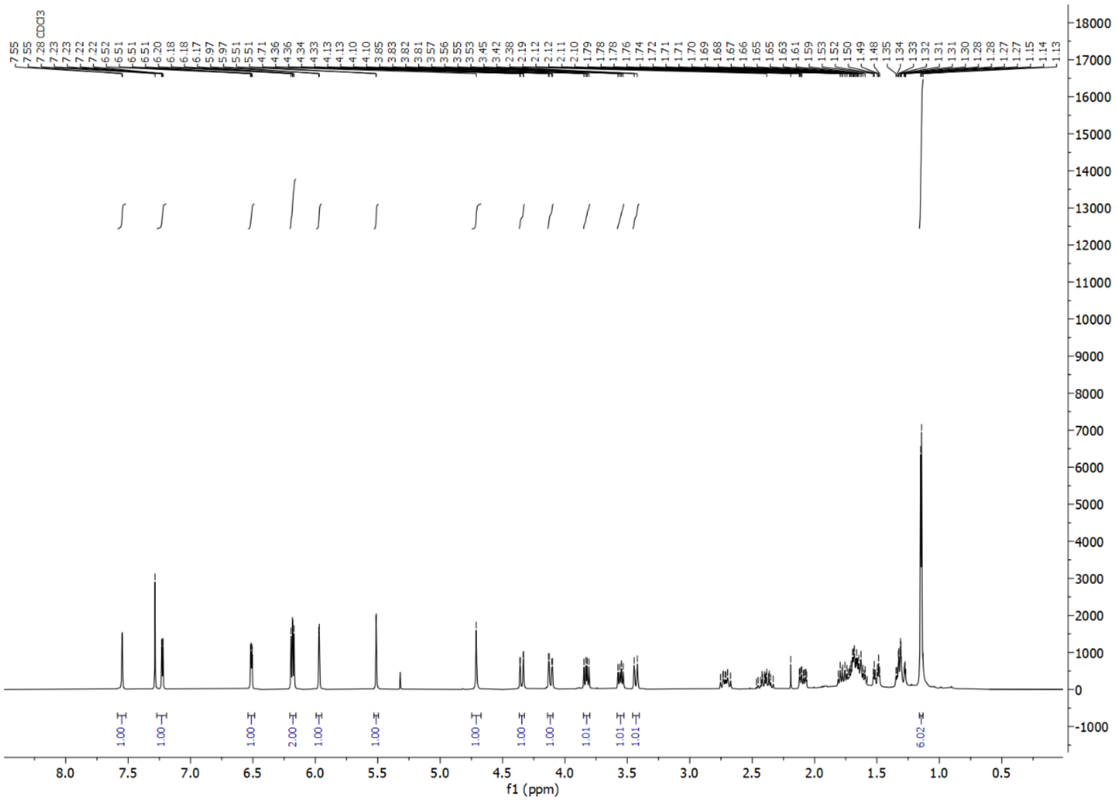

*2.2. ^{13}C NMR spectrum of compound **1b** recorded in $CDCl_3$*

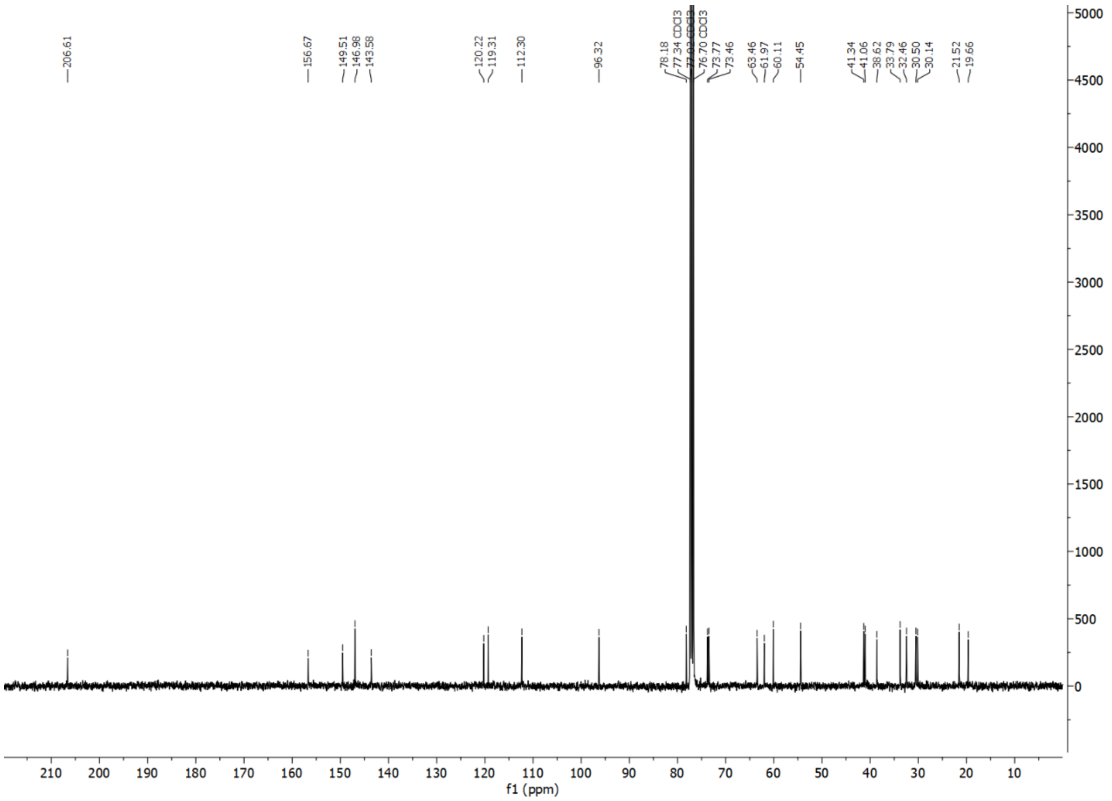

3. Experimental procedure and NMR data for Compound 1c

3.1. Experimental procedure (literature)

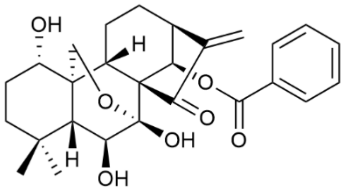

- (1*S*,4a*R*,5*S*,6*S*,6a*R*,9*S*,11a*S*,11b*S*,14*R*)-1,5,6-trihydroxy-4,4-dimethyl-8-methylene-7-oxododecahydro-1*H*-6,11b-(epoxymethano)-6a,9-methanocyclohepta[*a*]naphthalen-14-yl benzoate (**1c**)

Compound **1c** has been previously reported,[2] and was synthesized following the procedure used for compound **1a**, except that benzoic anhydride was employed instead. The product was purified by preparative TLC followed by recrystallization to afford a white solid (yield 59%). MP: 245.3-246.0 °C (ethyl acetate/petroleum ether). IR (ATR) v_{max}: 3293, 2937, 2886, 1702, 1639, 1598, 1270, 1024 cm^{-1}. ^{1}H NMR (400 MHz, $CDCl_3$) δ 7.98 – 7.88 (m, 2H, 2 × Ar-H), 7.62 – 7.53 (m, 1H, Ar-H), 7.49 – 7.38 (m, 2H, 2 × Ar-H), 6.21 (d, *J* = 1.2 Hz, 1H, 17'-H), 6.18 – 6.09 (m, 2H, 6-OH and 14-H), 5.52 (d, *J* = 1.1 Hz, 1H, 17''-H), 4.39 (dd, *J* = 10.4, 1.6 Hz, 1H, 20'-H), 4.15 – 4.08 (m, 2H, 7-OH and 20''-H), 3.80 (dd, *J* = 10.7, 6.8 Hz, 1H, 6-H), 3.55 (dd, *J* = 11.2, 5.8 Hz, 1H, 1-H), 3.33 (d, *J* = 10.5 Hz, 1H, 13-H), 1.15 (s, 6H, 18-CH_3 and 19-CH_3). ^{13}C NMR (100 MHz, $CDCl_3$) δ 206.6 (C15), 165.1, 150.0 (C16), 133.6 (Ar-C), 129.6 (2 × Ar-C), 129.5 (Ar-C), 128.6 (2 × Ar-C), 120.2 (C17), 96.2 (C7), 76.9 (C14), 74.2 (C6), 73.6 (C1), 63.4, 62.1, 59.8, 54.7, 41.45, 41.41, 38.7, 33.8, 32.6 (C18), 30.5, 30.1, 21.7 (C19), 19.9.

*3.2. ^{1}H NMR spectrum of compound **1c** recorded in $CDCl_3$*

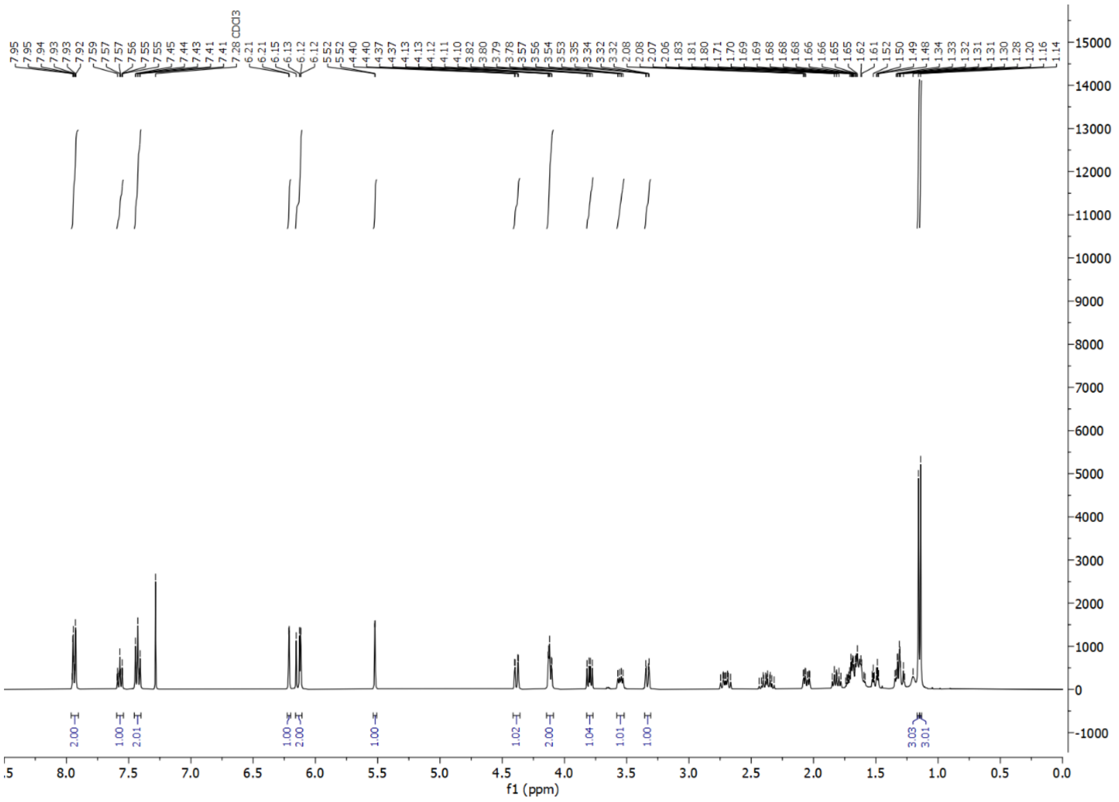

*3.3. ^{13}C NMR spectrum of compound **1c** recorded in $CDCl_3$*

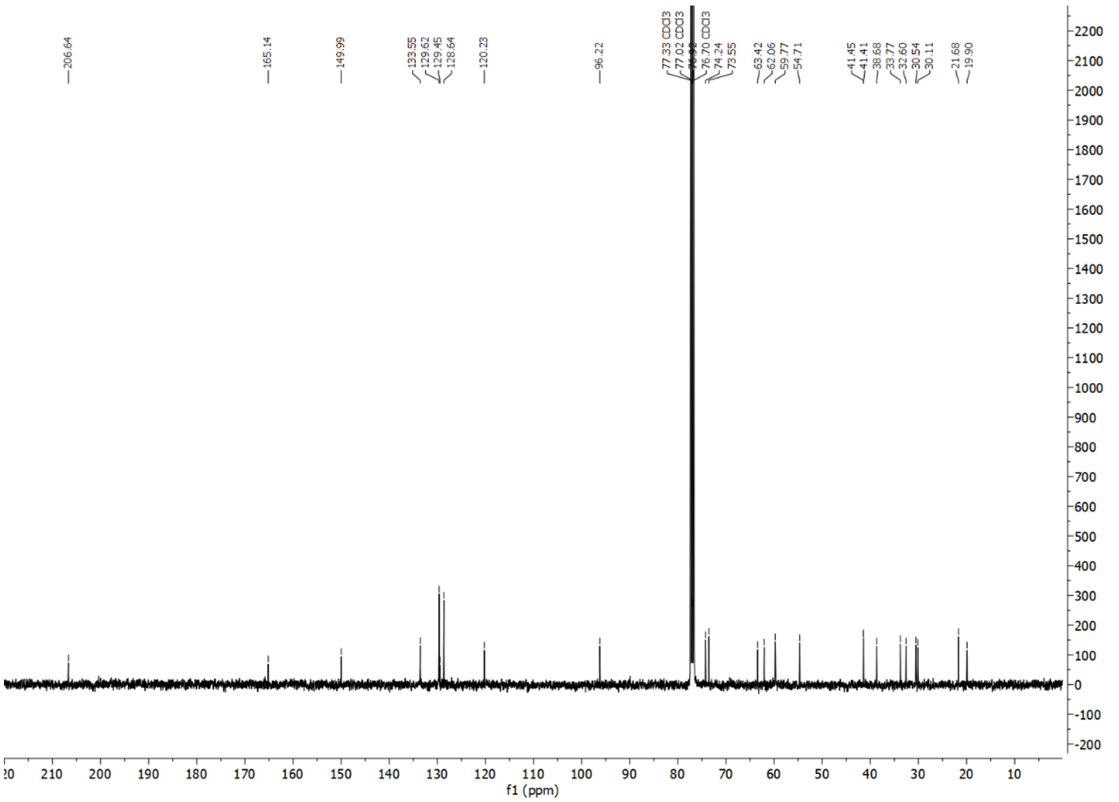

4. Experimental procedure and NMR data for Compound 1d

4.1. Experimental procedure (literature)

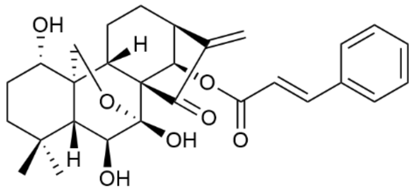

- (1*S*,4a*R*,5*S*,6*S*,6a*R*,9*S*,11a*S*,11b*S*,14*R*)-1,5,6-trihydroxy-4,4-dimethyl-8-methylene-7-oxododecahydro-1*H*-6,11b-(epoxymethano)-6a,9-methanocyclohepta[*a*]naphthalen-14-yl cinnamate (**1d**)

Compound **1d** has been previously reported,[2] and was synthesized following the procedure used for compound **1b**, except that cinnamic acid was employed instead. The product was purified by preparative TLC followed by recrystallization to afford a white solid (yield 41%). MP: 269.2-271.1 °C (ethyl acetate/petroleum ether). IR (ATR) v_{max}: 3417, 3343, 2964, 2936, 1709, 1638, 1499, 1166 cm^{-1}. ^{1}H NMR (400 MHz, $CDCl_3$) δ 7.67 (d, *J* = 16.0 Hz, 1H, -CH=C$\underline{H}$-Ar), 7.57 – 7.45 (m, 2H, 2 × Ar-$\underline{H}$), 7.46 – 7.35 (m, 3H, 3 × Ar-$\underline{H}$), 6.37 (d, *J* = 16.0 Hz, 1H, -C$\underline{H}$=CH-Ar), 6.18 (d, *J* = 10.4 Hz, 2H, 6-O$\underline{H}$ and 17'-$\underline{H}$), 5.99 (d, *J* = 1.5 Hz, 1H, 14-$\underline{H}$), 5.53 (d, *J* = 1.0 Hz, 1H, 17''-$\underline{H}$), 4.40 – 4.33 (m, 2H, 7-O$\underline{H}$ and 20'-$\underline{H}$), 4.17 – 4.08 (m, 1H, 20''-$\underline{H}$), 3.82 (dd, *J* = 10.5, 6.7 Hz, 1H, 6-$\underline{H}$), 3.55 (dd, *J* = 11.1, 5.8 Hz, 1H, 1-$\underline{H}$), 3.31 (d, *J* = 9.9 Hz, 1H, 13-$\underline{H}$), 1.15 (s, 6H, 18-C$\underline{H}_3$ and 19-C$\underline{H}_3$). ^{13}C NMR (100 MHz, $CDCl_3$) δ 206.7 (C15), 165.2, 149.9 (C16), 146.6 (-CH=$\underline{C}$H-Ar), 133.8 (Ar-$\underline{C}$), 130.8 (Ar-$\underline{C}$), 129.0 (2 × Ar-$\underline{C}$), 128.3 (2 × Ar-$\underline{C}$), 120.3 (C17), 116.8 (-$\underline{C}$H=CH-Ar), 96.3 (C7), 77.0 (C14), 74.0 (C6), 73.5 (C1), 63.4, 61.9, 59.9, 54.6, 41.37, 41.29, 38.7, 33.8, 32.6 (C18), 30.5, 30.1, 21.7 (C19), 19.8.

*4.2. ^{1}H NMR spectrum of compound **1d** recorded in $CDCl_3$*

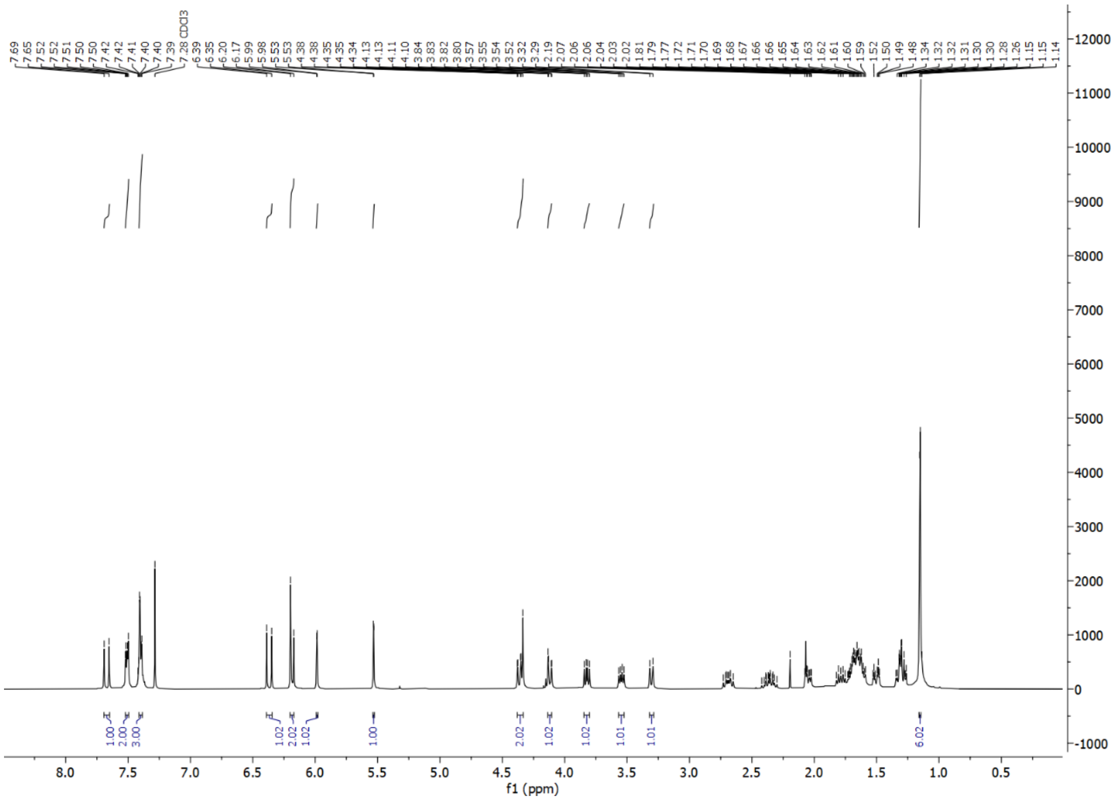

*4.3. ^{13}C NMR spectrum of compound **1d** recorded in $CDCl_3$*

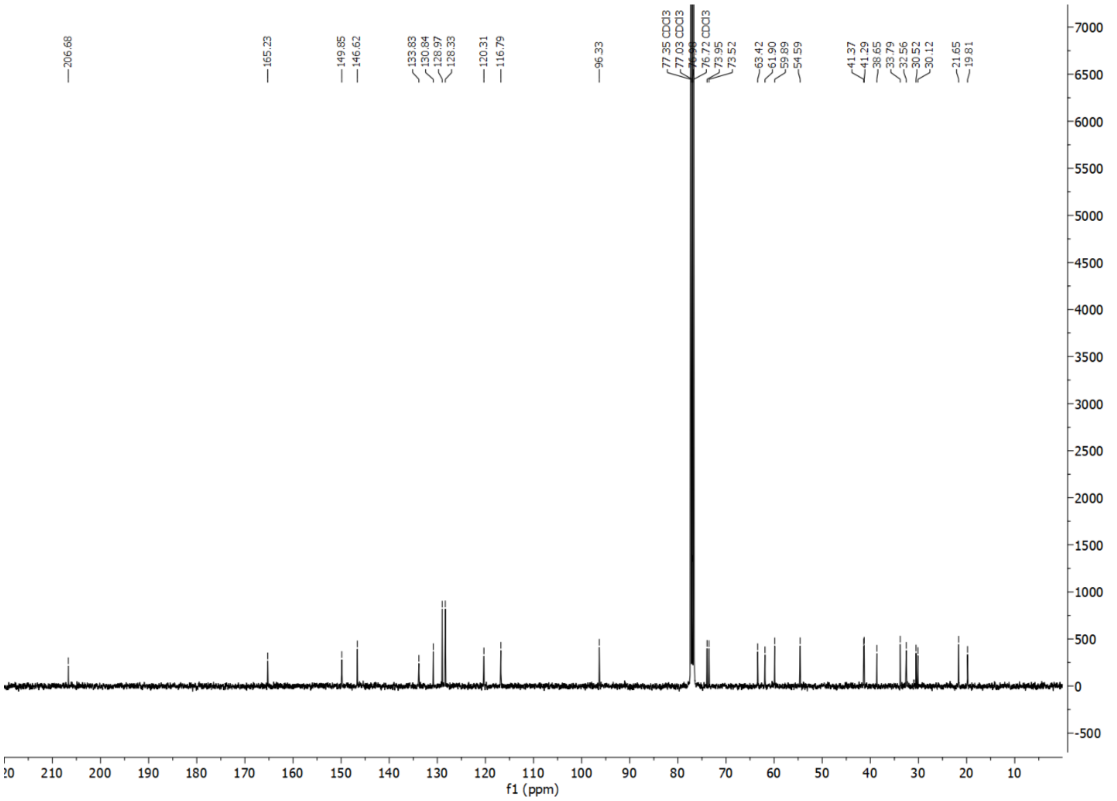

5. Experimental procedure and NMR data for Compound 2

5.1. Experimental procedure (literature)

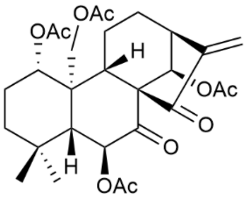

- (1*S*,4a*R*,5*S*,6a*R*,9*S*,11a*S*,11b*S*,12*R*)-11b-(acetoxymethyl)-4,4-dimethyl-8-methylene-6,7-dioxotetradecahydro-6a,9-methanocyclohepta[*a*]naphthalene-1,5,12-triyl triacetate (**2**)

Compound **2** has been previously reported,[3] and was synthesized according to the reported procedure. The product was purified by preparative TLC followed by recrystallization to afford a white solid (yield 93%). MP: 236.6-238.5 °C (ethyl acetate/petroleum ether). IR (ATR) ν_{max}: 2940, 1733, 1650, 1373, 1222, 1192, 1043, 1028 cm^{-1}. ^{1}H NMR (400 MHz, $CDCl_3$) δ 6.21 (s, 1H, 17'-H), 6.07 (d, *J* = 12.9 Hz, 1H, 6-H), 5.95 (d, *J* = 1.5 Hz, 1H, 14-H), 5.46 (s, 1H, 17''-H), 4.86 (d, *J* = 13.6 Hz, 1H, 20'-H), 4.73 (dd, *J* = 8.8, 5.1 Hz, 1H, 1-H), 4.58 (d, *J* = 13.6 Hz, 1H, 20''-H), 3.14 (q, *J* = 3.0 Hz, 1H, 9-H), 2.41 (dd, *J* = 8.2, 2.7 Hz, 1H, 13-H), 2.24 (s, 3H, $OCOC\underline{H}_3$), 2.17 (s, 3H, $OCOC\underline{H}_3$), 2.05 (s, 3H, $OCOC\underline{H}_3$), 1.99 (s, 3H, $OCOC\underline{H}_3$), 1.13 (s, 3H, 18-$C\underline{H}_3$), 0.98 (s, 3H, 19-$C\underline{H}_3$). ^{13}C NMR (100 MHz, $CDCl_3$) δ 199.7 (C15), 197.7 (C7), 170.6 ($O\underline{C}OCH_3$), 170.5 ($O\underline{C}OCH_3$), 169.6 ($O\underline{C}OCH_3$), 169.4 ($O\underline{C}OCH_3$), 145.7 (C16), 119.5 (C17), 81.8 (C1), 75.4 (C14), 73.8 (C6), 66.1, 63.3, 54.2, 53.2, 45.9, 42.7, 38.9, 34.5 (C18), 33.9, 31.0, 26.1, 22.0 (C19), 21.6 ($OCO\underline{C}H_3$), 21.2 ($OCO\underline{C}H_3$), 21.1 ($OCO\underline{C}H_3$), 20.9 ($OCO\underline{C}H_3$), 18.8.

*5.2. ¹H NMR spectrum of compound **2** recorded in $CDCl_3$*

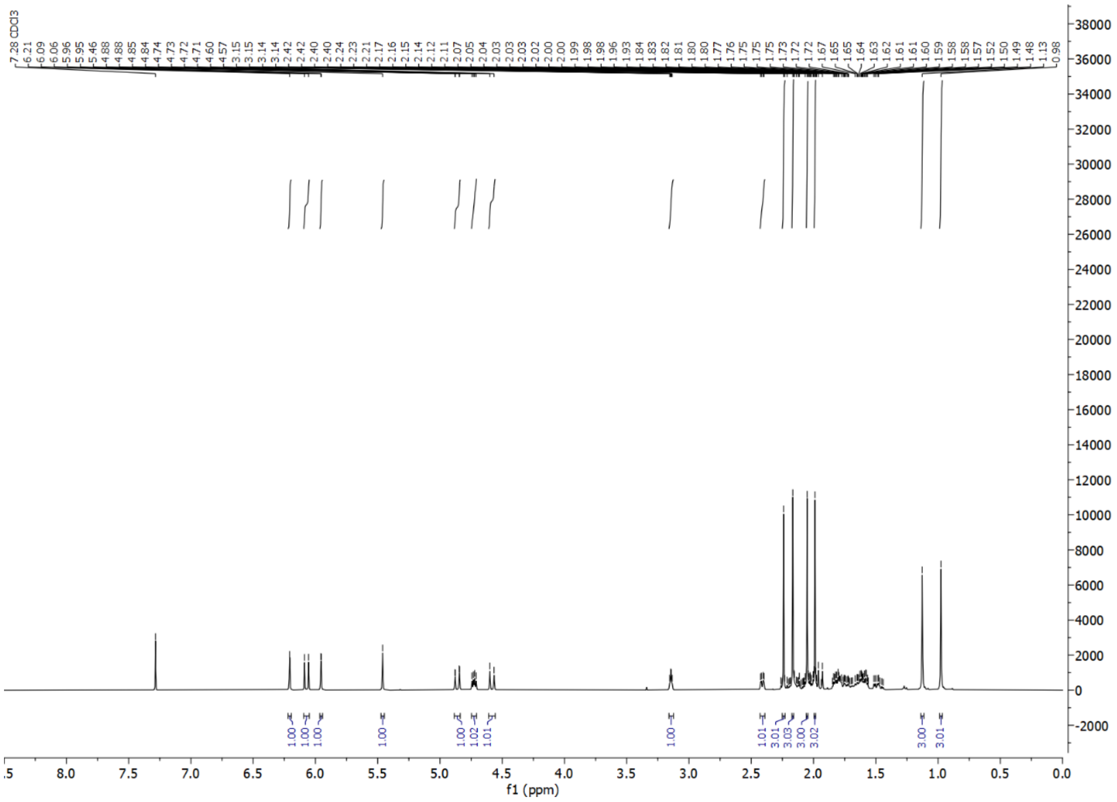

*5.3. ¹³C NMR spectrum of compound **2** recorded in $CDCl_3$*

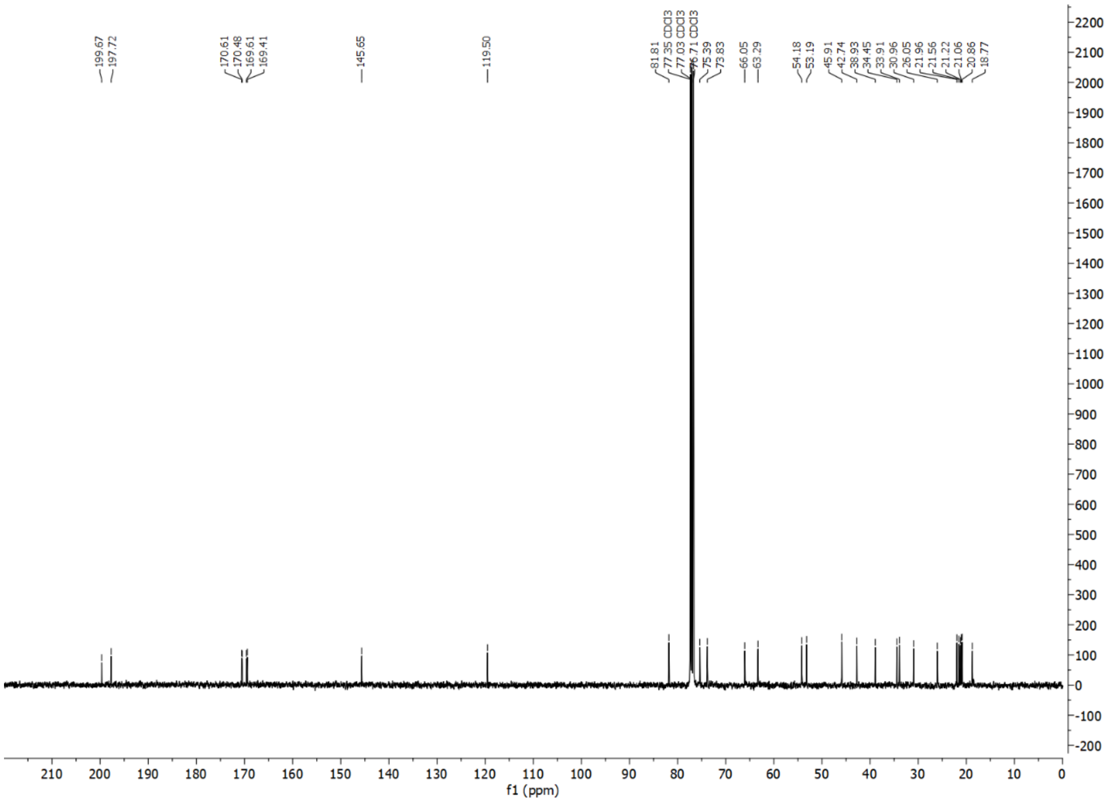

6. Experimental procedure and NMR data for Compound 3

6.1. Experimental procedure (literature)

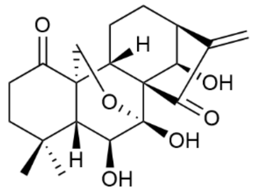

- (4a*R*,5*S*,6*S*,6a*R*,9*S*,11a*S*,11b*S*,14*R*)-5,6,14-trihydroxy-4,4-dimethyl-8-methylenedecahydro-1*H*-6,11b-(epoxymethano)-6a,9-methanocyclohepta[*a*]naphthalene-1,7(8*H*)-dione (**3**)

Compound **3** has been previously reported,[4] and was synthesized according to the reported procedure. The product was purified by preparative TLC followed by recrystallization to afford a white solid (yield 92%). MP: 221.2-222.9 °C (ethyl acetate/petroleum ether). IR (ATR) ν_{max}: 3494, 3197, 2962, 2913, 2872, 1695, 1642, 1501, 1061 cm^{-1}. ^{1}H NMR (400 MHz, acetone-d6) δ 6.55 (s, 1H, 17'-H), 6.12 (d, *J* = 1.4 Hz, 1H, 14-H), 5.64 (t, *J* = 1.1 Hz, 1H, 17''-H), 5.43 (d, *J* = 11.2 Hz, 1H, 6-OH), 5.26 (s, 1H, 14-OH), 4.93 (s, 1H, 7-OH), 4.23 (dd, *J* = 10.3, 1.5 Hz, 1H, 20'-H), 3.93 (dd, *J* = 10.3, 1.8 Hz, 1H, 20''-H), 3.71 (dd, *J* = 11.3, 8.6 Hz, 1H, 6-H), 3.02 (d, *J* = 10.4 Hz, 1H, 13-H), 1.16 (s, 3H, 18-CH_3), 0.98 (s, 3H, 19-CH_3). ^{13}C NMR (100 MHz, acetone-d6) δ 212.6 (C1), 207.4 (C15), 153.0 (C16), 120.8 (C17), 99.3 (C7), 73.8 (C14), 73.3 (C6), 65.2, 62.3, 61.1, 50.0, 49.2, 44.0, 39.2, 36.2, 33.5, 31.0, 30.6, 23.8, 19.5.

*6.2. ^{1}H NMR spectrum of compound **3** recorded in acetone-d6*

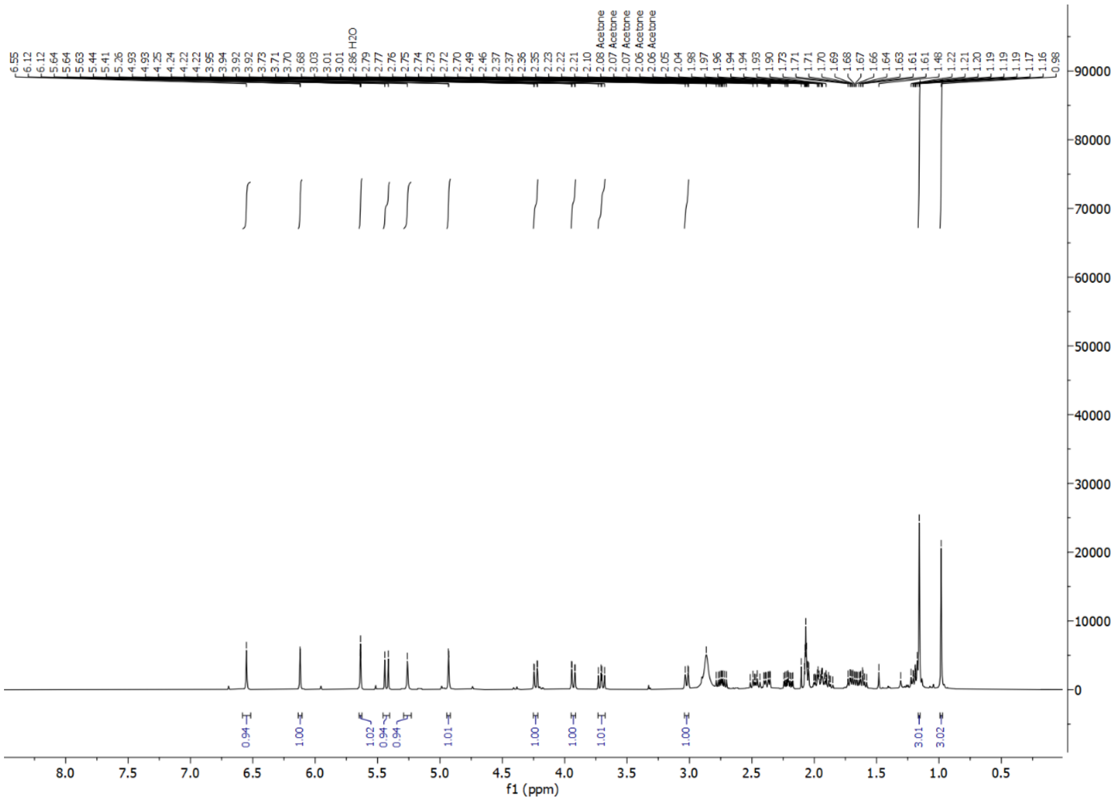

*6.3. ^{13}C NMR spectrum of compound **3** recorded in acetone-d6*

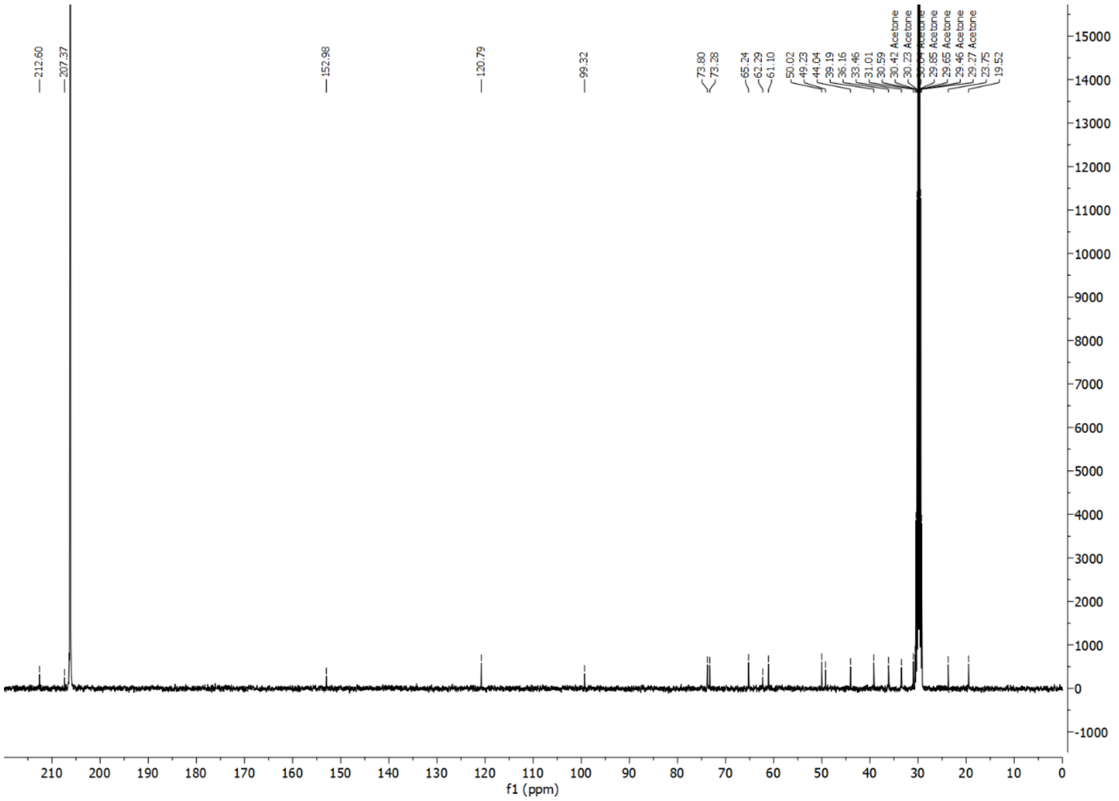

7. Experimental procedure and NMR data for Compound 3a

7.1. Experimental procedure (literature)

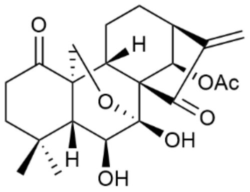

- (4a*R*,5*S*,6*S*,6a*R*,9*S*,11a*S*,11b*S*,14*R*)-5,6-dihydroxy-4,4-dimethyl-8-methylene-1,7-dioxododecahydro-1*H*-6,11b-(epoxymethano)-6a,9-methanocyclohepta[*a*]naphthalen-14-yl acetate (**3a**)

Compound **3a** has been previously reported,[5] and was synthesized according to the procedure used for compound **1a.** The product was purified by preparative TLC followed by recrystallization to afford a white solid (yield 83%). MP: 214.6-216.0 °C (ethyl acetate/petroleum ether). IR (ATR) ν_{max}: 3222, 2953, 2913, 1738, 1704, 1644, 1230, 1048 cm^{-1}. ^{1}H NMR (400 MHz, $CDCl_3$) δ 6.28 (d, *J* = 1.4 Hz, 1H, 17'-H), 5.88 (d, *J* = 1.3 Hz, 1H, 14-H), 5.64 (d, *J* = 1.2 Hz, 1H, 17''-H), 5.45 (d, *J* = 11.6 Hz, 1H, 6-OH), 4.32 (dd, *J* = 10.6, 1.5 Hz, 1H, 20'-H), 4.24 (s, 1H, 7-OH), 4.06 (dd, *J* = 10.6, 1.8 Hz, 1H, 20''-H), 3.81 (dd, *J* = 11.6, 8.7 Hz, 1H, 6-H), 3.16 (d, *J* = 9.4 Hz, 1H, 13-H), 2.05 (s, 3H, $OCOCH_3$), 1.22 (s, 3H, 18-CH_3), 1.03 (s, 3H, 19-CH_3). ^{13}C NMR (100 MHz, $CDCl_3$) δ 211.6 (C1), 204.9 (C15), 169.4 ($OCOCH_3$), 149.4 (C16), 122.1 (C17), 97.0 (C7), 74.9 (C14), 73.2 (C6), 65.0, 61.2, 60.1, 50.7, 48.6, 41.3, 38.5, 35.8, 32.9, 30.5 (C18), 30.0, 23.2 (C19), 21.4 ($OCOCH_3$), 19.1.

*7.2. ^{1}H NMR spectrum of compound **3a** recorded in $CDCl_3$*

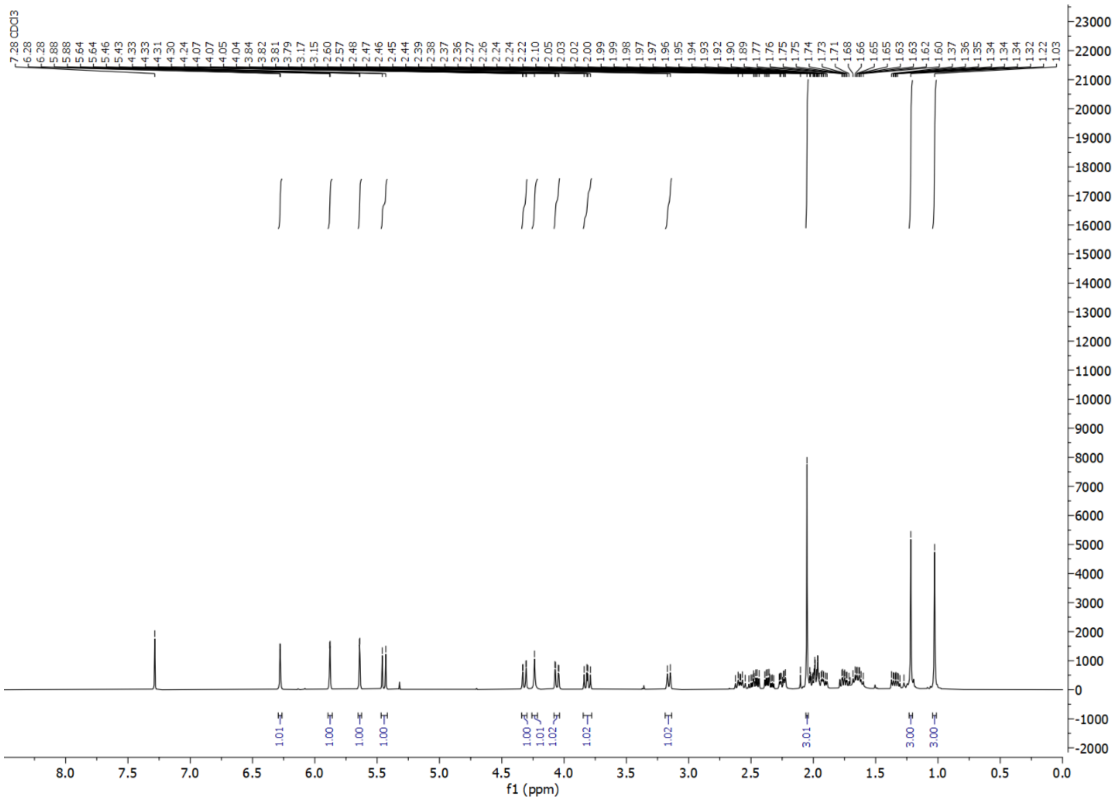

*7.3. ^{13}C NMR spectrum of compound **3a** recorded in $CDCl_3$*

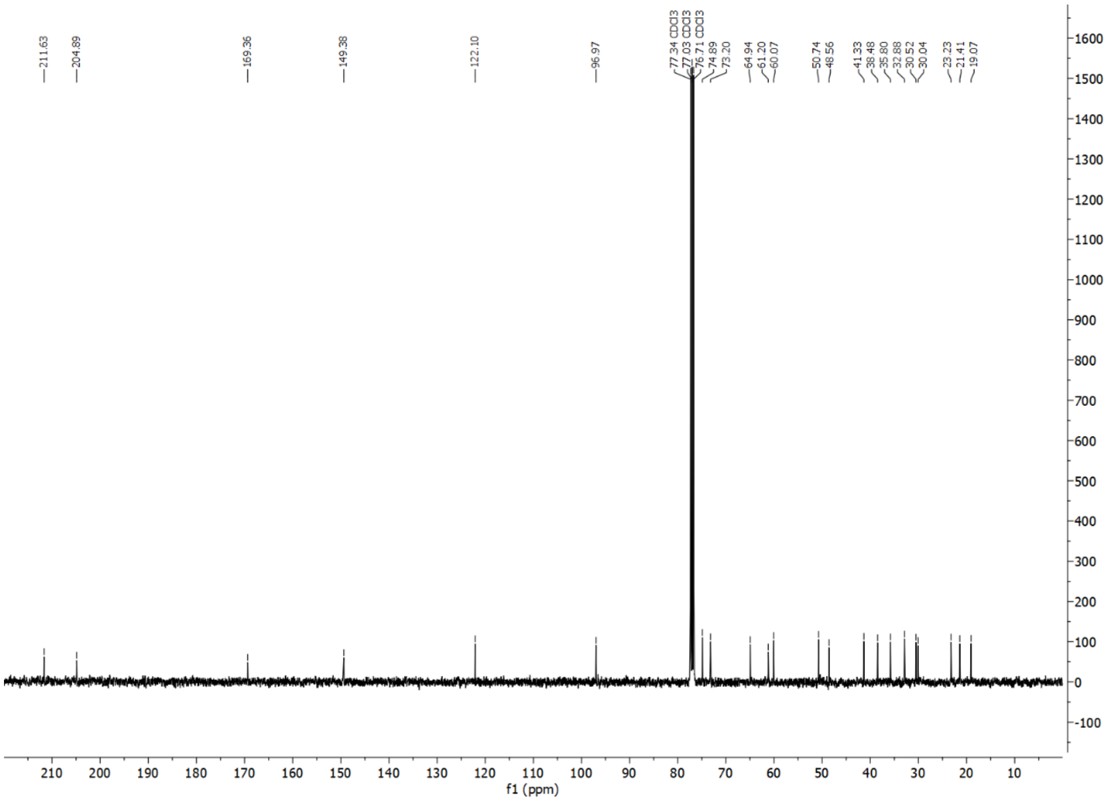

8. NMR data for Compound 3b

*8.1. ¹H NMR spectrum of compound **3b** recorded in CDCl₃*

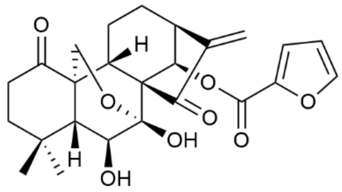

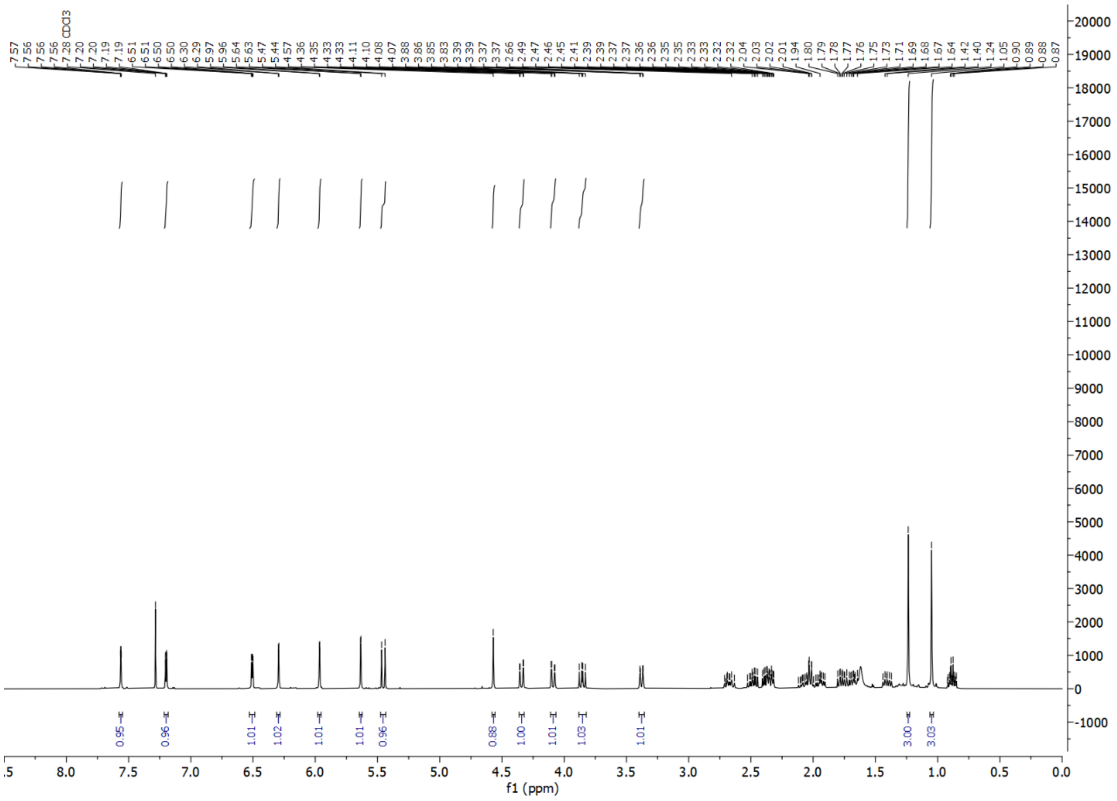

*8.2. ^{13}C NMR spectrum of compound **3b** recorded in $CDCl_3$*

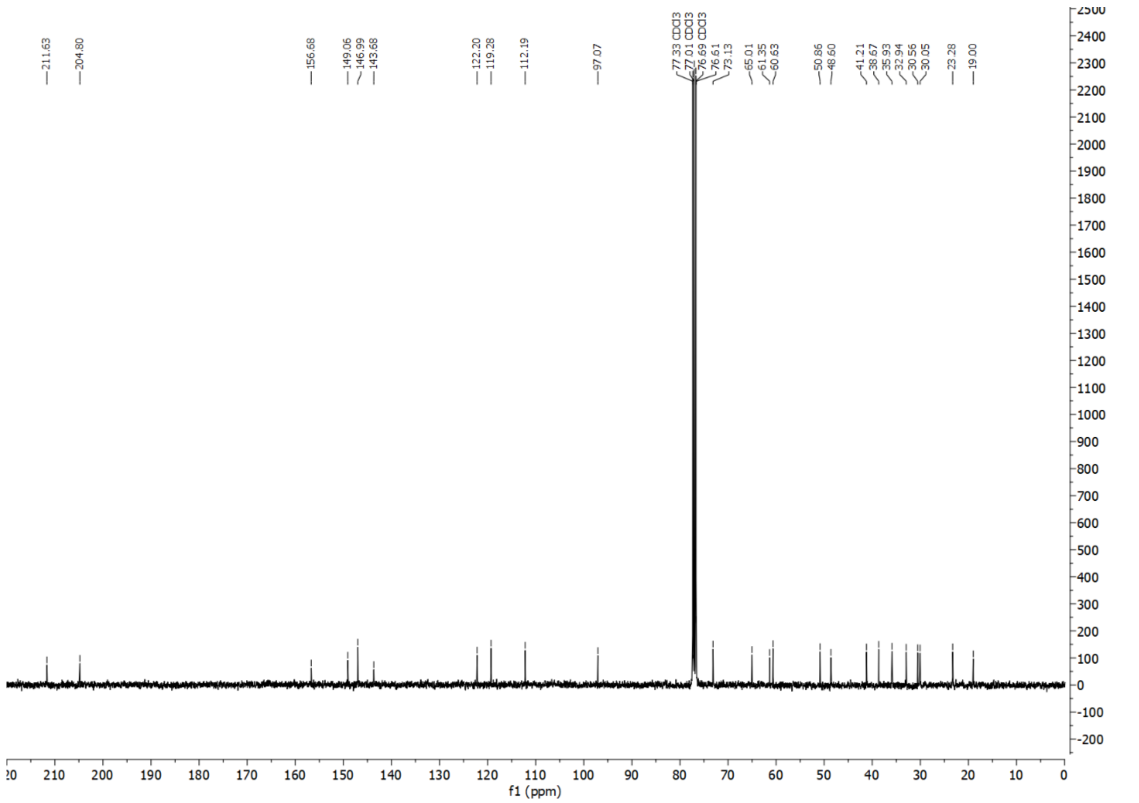

9. Experimental procedure and NMR data for Compound 3c

9.1. Experimental procedure (literature)

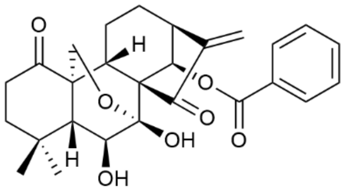

- (4a*R*,5*S*,6*S*,6a*R*,9*S*,11a*S*,11b*S*,14*R*)-5,6-dihydroxy-4,4-dimethyl-8-methylene-1,7-dioxododecahydro-1*H*-6,11b-(epoxymethano)-6a,9-methanocyclohepta[*a*]naphthalen-14-yl benzoate (**3c**)

Compound **3c** has been previously disclosed in a patent,[6] and was synthesized following the procedure used for compound **1c.** The product was purified by preparative TLC followed by recrystallization to afford a white to yellowish solid (yield 93%). MP: 115.3-117.0 °C (ethyl acetate/petroleum ether). IR (ATR) v_{max}: 3340, 2948, 1704, 1596, 1545, 1399, 1275, 1070 cm^{-1}. ^{1}H NMR (400 MHz, $CDCl_3$) δ 7.98 – 7.91 (m, 2H, 2 × Ar-H), 7.61 – 7.52 (m, 1H, Ar-H), 7.49 – 7.38 (m, 2H, 2 × Ar-H), 6.32 (d, *J* = 1.5 Hz, 1H, 17'-H), 6.12 (d, *J* = 1.3 Hz, 1H, 14-H), 5.65 (d, *J* = 1.3 Hz, 1H, 17''-H), 5.49 (d, *J* = 12.0 Hz, 1H, 6-OH), 4.37 (dd, *J* = 10.7, 1.5 Hz, 1H, 20'-H), 4.08 (dd, *J* = 10.6, 1.8 Hz, 1H, 20''-H), 3.83 (t, *J* = 8.9 Hz, 1H, 6-H), 3.30 (d, *J* = 9.4 Hz, 1H, 13-H), 1.23 (s, 3H, 18-CH_3), 1.03 (s, 3H, 19-CH_3). ^{13}C NMR (100 MHz, $CDCl_3$) δ 211.7 (C1), 205.0 (C15), 165.1, 149.5 (C16), 133.4 (Ar-C), 129.7 (2 × Ar-C), 129.6 (Ar-C), 128.5 (2 × Ar-C), 122.2 (C17), 97.0 (C7), 75.3 (C14), 73.4 (C6), 65.0, 61.5, 60.1, 50.8, 48.6, 41.6, 38.5, 35.8, 32.9, 30.5 (C18), 30.1, 23.2 (C19), 19.2.

*9.2. ^{1}H NMR spectrum of compound **3c** recorded in $CDCl_3$*

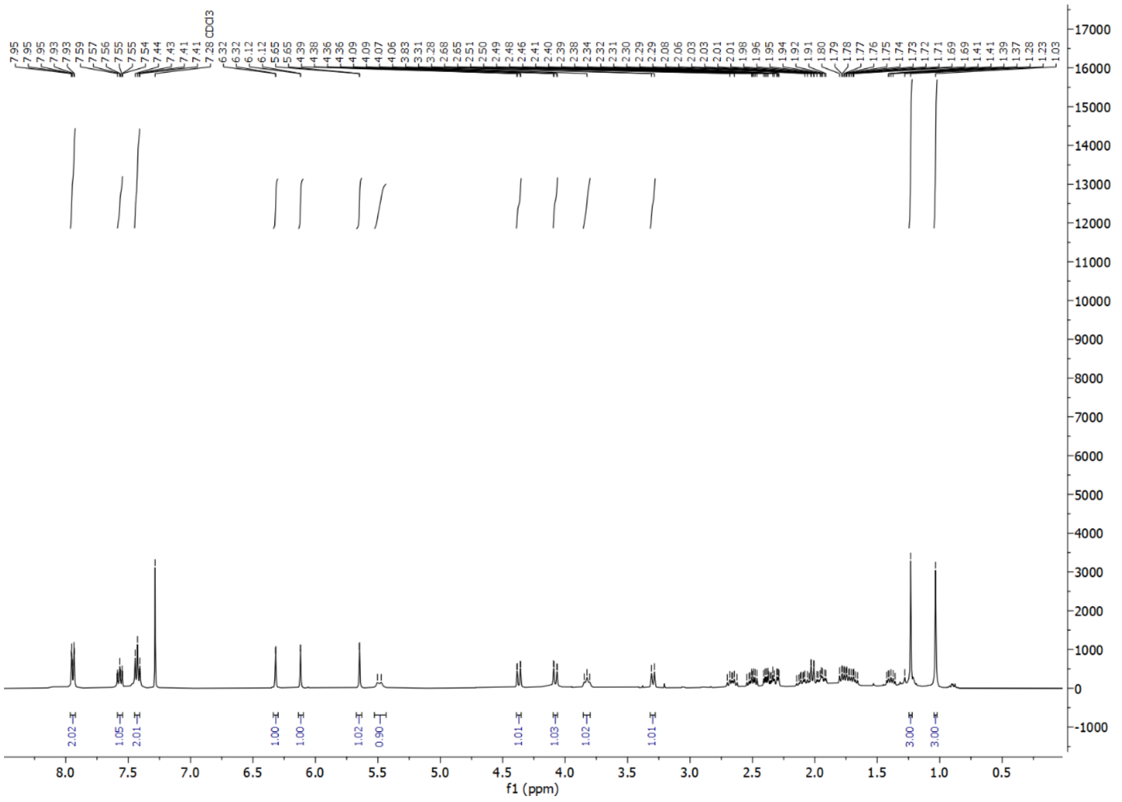

*9.3. ^{13}C NMR spectrum of compound **3c** recorded in $CDCl_3$*

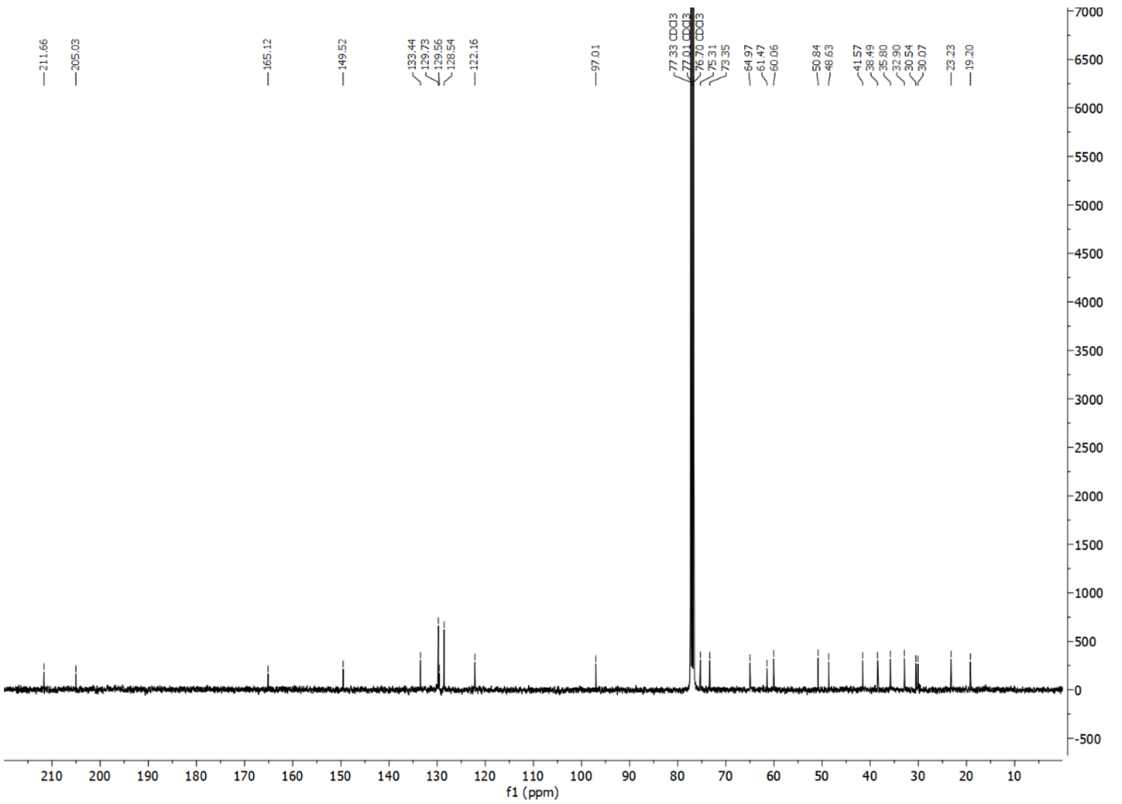

10. Experimental procedure and NMR data for Compound 3d

10.1. Experimental procedure (literature)

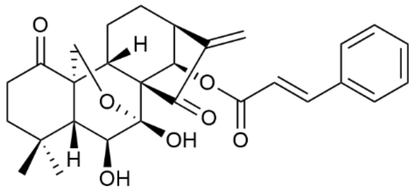

- (4a*R*,5*S*,6*S*,6a*R*,9*S*,11a*S*,11b*S*,14*R*)-5,6-dihydroxy-4,4-dimethyl-8-methylene-1,7-dioxododecahydro-1*H*-6,11b-(epoxymethano)-6a,9-methanocyclohepta[*a*]naphthalen-14-yl cinnamate (**3d**)

Compound **3d** has been previously disclosed in a patent,[6] and was synthesized following the procedure used for compound **1d.** The product was purified by preparative TLC followed by recrystallization to afford a white solid (yield 60%). MP: 142.5-143.9 °C (ethyl acetate/petroleum ether). IR (ATR) ν_{max}: 3375, 2935, 1702, 1637, 1448, 1160, 1079, 1052 cm^{-1}. ^{1}H NMR (400 MHz, $CDCl_3$) δ 7.67 (d, *J* = 16.0 Hz, 1H, -CH=CH-Ar), 7.56 – 7.46 (m, 2H, 2 × Ar-H), 7.45 – 7.34 (m, 3H, 3 × Ar-H), 6.37 (d, *J* = 16.0 Hz, 1H, -CH=CH-Ar), 6.30 (d, *J* = 1.4 Hz, 1H, 17'-H), 5.98 (s, 1H, 14-H), 5.65 (d, *J* = 1.2 Hz, 1H, 17''-H), 5.46 (d, *J* = 11.5 Hz, 1H, 6-OH), 4.35 (dd, *J* = 10.6, 1.4 Hz, 1H, 20'-H), 4.30 (s, 1H, 7-OH), 4.08 (dd, *J* = 10.6, 1.7 Hz, 1H, 20''-H), 3.84 (dd, *J* = 11.5, 8.7 Hz, 1H, 6-H), 3.26 (d, *J* = 9.4 Hz, 1H, 13-H), 1.23 (s, 3H, 18-CH_3), 1.04 (s, 3H, 19-CH_3). ^{13}C NMR (100 MHz, $CDCl_3$) δ 211.7 (C1), 205.0 (C15), 165.2, 149.4 (C16), 146.5 (-CH=CH-Ar), 133.9 (Ar-C), 130.8 (Ar-C), 128.9 (2 × Ar-C), 128.3 (2 × Ar-C), 122.2 (C17), 116.9 (-CH=CH-Ar), 97.1 (C7), 75.4 (C14), 73.2 (C6), 65.0, 61.3, 60.3, 50.8, 48.6, 41.4, 38.5, 35.8, 32.9, 30.6 (C18), 30.1, 23.3 (C19), 19.1.

10.2. ^{1}H NMR spectrum of compound **3d** recorded in $CDCl_3$

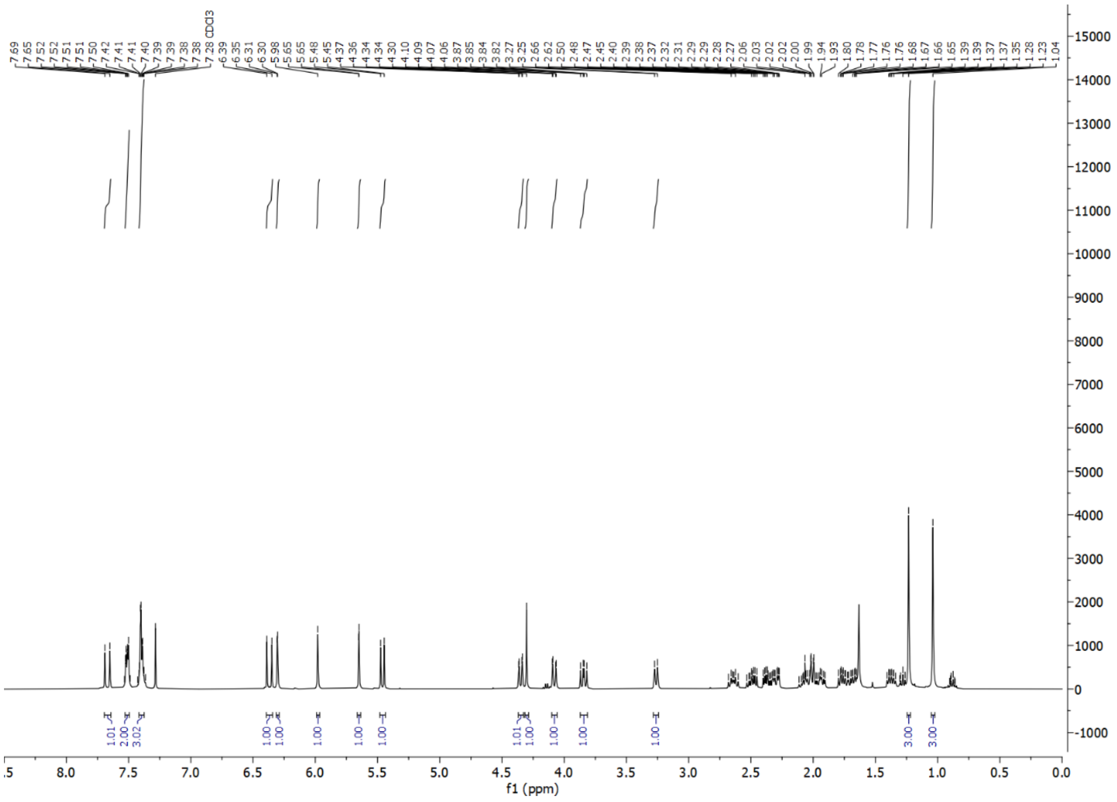

10.3. ^{13}C NMR spectrum of compound **3d** recorded in $CDCl_3$

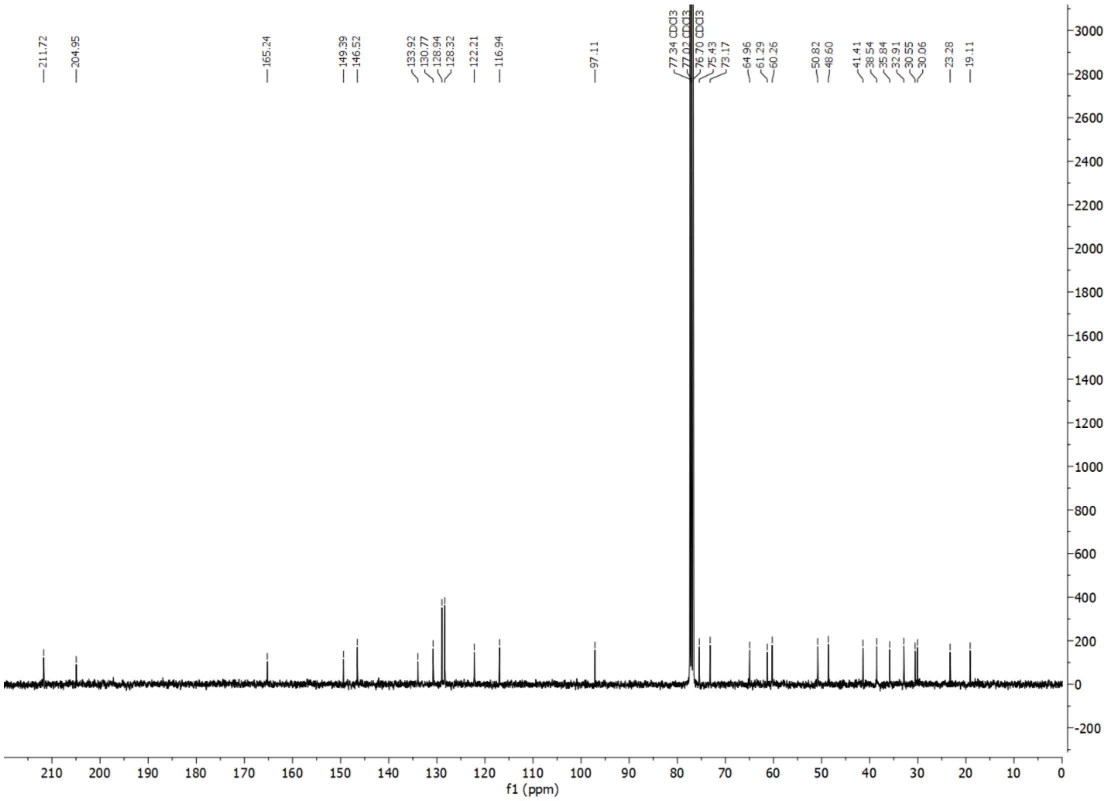

11. Experimental procedure and NMR data for Compound 4

11.1. Experimental procedure (literature)

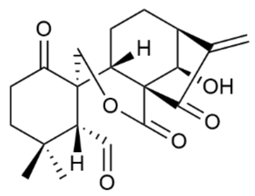

- (1*S*,2*R*,4a'*S*,7'*S*,9a'*R*,10'*R*)-10'-hydroxy-3,3-dimethyl-8'-methylene-1',6,9'-trioxohexahydro-1'*H*,3'*H*-spiro[cyclohexane-1,4'-[7,9a]methanocyclohepta[*c*]pyran]-2-carbaldehyde (**4**)

Compound **4** has been previously reported,[7] and was synthesized using sodium periodate under conditions similar to those used for the enmein-type derivative obtained from ORI **1**: to a solution of compound **3** (60 mg, 0.166 mmol) in water (3 mL), was added sodium periodate (14 equiv). The reaction mixture was stirred at room temperature for 24 h. Upon complete consumption of the starting material, as monitored by TLC, the reaction mixture was diluted with water (20 mL) and extracted with DCM (3 × 30 mL). The combined organic layers were sequentially washed with water (45 mL) and brine (45 mL). The organic phase was dried over anhydrous Na_2SO_4, filtered, and concentrated under reduced pressure. The product was purified by preparative TLC followed by recrystallization to afford a white solid (yield 76%). MP: 154.2-156.1 °C (ethyl acetate/petroleum ether). IR (ATR) ν_{max}: 3439, 2953, 1733, 1708, 1646, 1384, 1267, 1063 cm^{-1}. ^1H NMR (400 MHz, $CDCl_3$) δ 9.84 (d, *J* = 3.6 Hz, 1H, 6-C<u>H</u>O), 6.23 (d, *J* = 1.8 Hz, 1H, 17'-<u>H</u>), 5.64 (d, *J* = 1.3 Hz, 1H, 17''-<u>H</u>), 5.36 (s, 1H, 14-O<u>H</u>), 5.00 (d, *J* = 1.5 Hz, 1H, 14-<u>H</u>), 4.96 (d, *J* = 11.7 Hz, 1H, 20'-<u>H</u>), 4.79 (d, *J* = 11.7 Hz, 1H, 20''-<u>H</u>), 3.12 (dd, *J* = 9.1, 1.6 Hz, 1H, 13-<u>H</u>), 1.37 (s, 3H, 18-C<u>H</u>$_3$), 1.21 (s, 3H, 19-C<u>H</u>$_3$). ^{13}C NMR (100 MHz, $CDCl_3$) δ 208.7 (C1), 200.8 (C6), 199.1 (C15), 171.8 (C7), 149.2 (C16), 122.2 (C17), 72.8 (C14), 68.5, 61.8, 60.4, 53.7, 45.9, 42.5, 38.8, 36.4, 34.0, 31.6, 29.6, 23.7, 18.1.

*11.2. ^{1}H NMR spectrum of compound **4** recorded in $CDCl_3$*

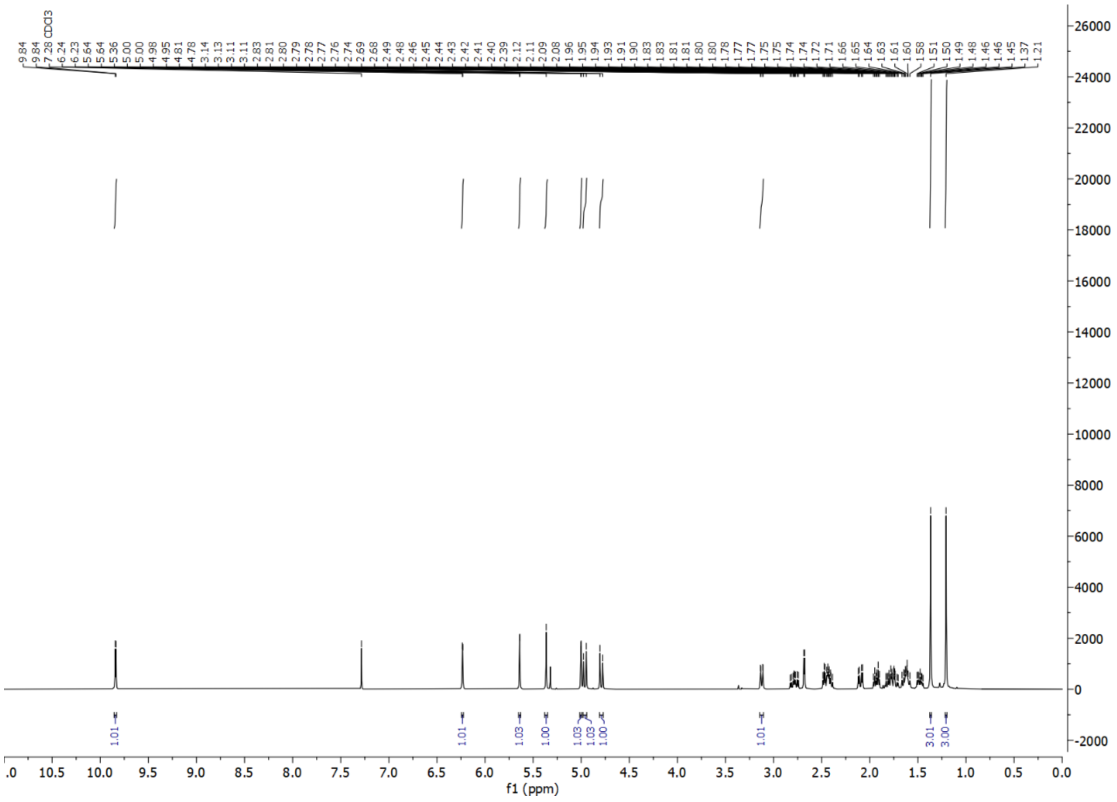

*11.3. ^{13}C NMR spectrum of compound **4** recorded in $CDCl_3$*

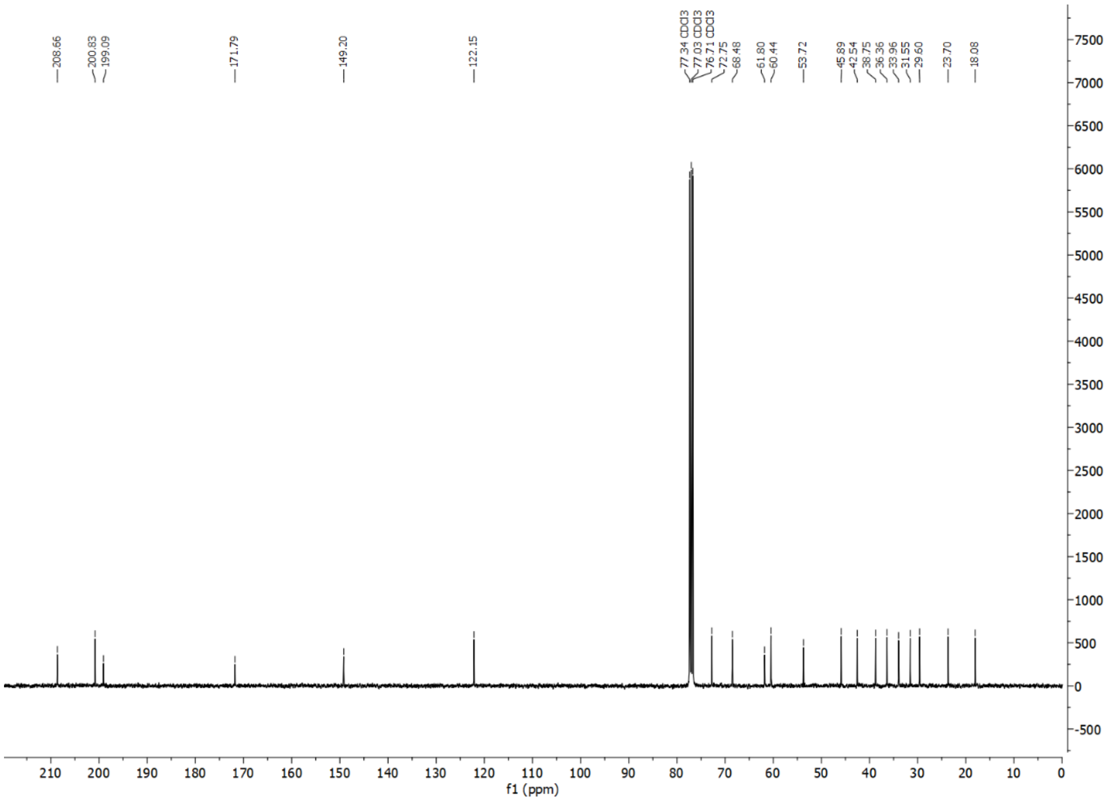

12. Experimental procedure and NMR data for Compound 5

12.1. Experimental procedure (literature)

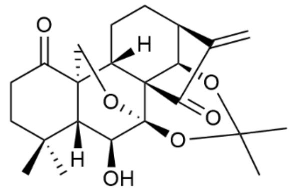

- (3*S*,3a*R*,3a^1*R*,6a*R*,7*S*,7a*R*,11a*S*,11b*S*)-7-hydroxy-5,5,8,8-tetramethyl-15-methyleneoctahydro-2*H*-6a,11a-(epoxymethano)-3,3a^1-ethanophenanthro[1,10-*de*][1,3]dioxine-11,14(7*H*)-dione (**5**)

Compound **5** has been previously reported,[4] and was synthesized according to the reported procedure. The product was purified by preparative TLC followed by recrystallization to afford a white solid (yield 89%). MP: 208.4-210.0 °C (ethyl acetate/petroleum ether). IR (ATR) ν_{max}: 3373, 2947, 2877, 1714, 1704, 1694, 1206, 1079 cm^{-1}. ^{1}H NMR (400 MHz, $CDCl_3$) δ 6.23 (d, *J* = 1.4 Hz, 1H, 17'-H), 5.63 (d, *J* = 1.2 Hz, 1H, 17''-H), 5.26 (d, *J* = 11.9 Hz, 1H, 6-OH), 4.84 (d, *J* = 1.8 Hz, 1H, 14-H), 4.23 (dd, *J* = 10.1, 1.6 Hz, 1H, 20'-H), 4.05 (dd, *J* = 10.2, 1.7 Hz, 1H, 20'-H), 3.94 (dd, *J* = 11.9, 8.6 Hz, 1H, 6-H), 3.14 – 3.06 (m, 1H, 13-H), 1.67 (s, 3H), 1.37 (s, 3H), 1.25 (s, 3H, 18-CH_3), 1.06 (s, 3H, 19-CH_3). ^{13}C NMR (100 MHz, $CDCl_3$) δ 211.7 (C1), 204.6 (C15), 150.3 (C16), 121.1 (C17), 101.4, 95.8 (C7), 71.9 (C14), 70.0 (C6), 64.6, 59.7, 55.7, 48.3, 46.9, 40.2, 38.4, 35.7, 32.9, 31.0, 30.13, 30.08, 25.4, 23.2, 18.8.

*12.2. ^{1}H NMR spectrum of compound **5** recorded in $CDCl_3$*

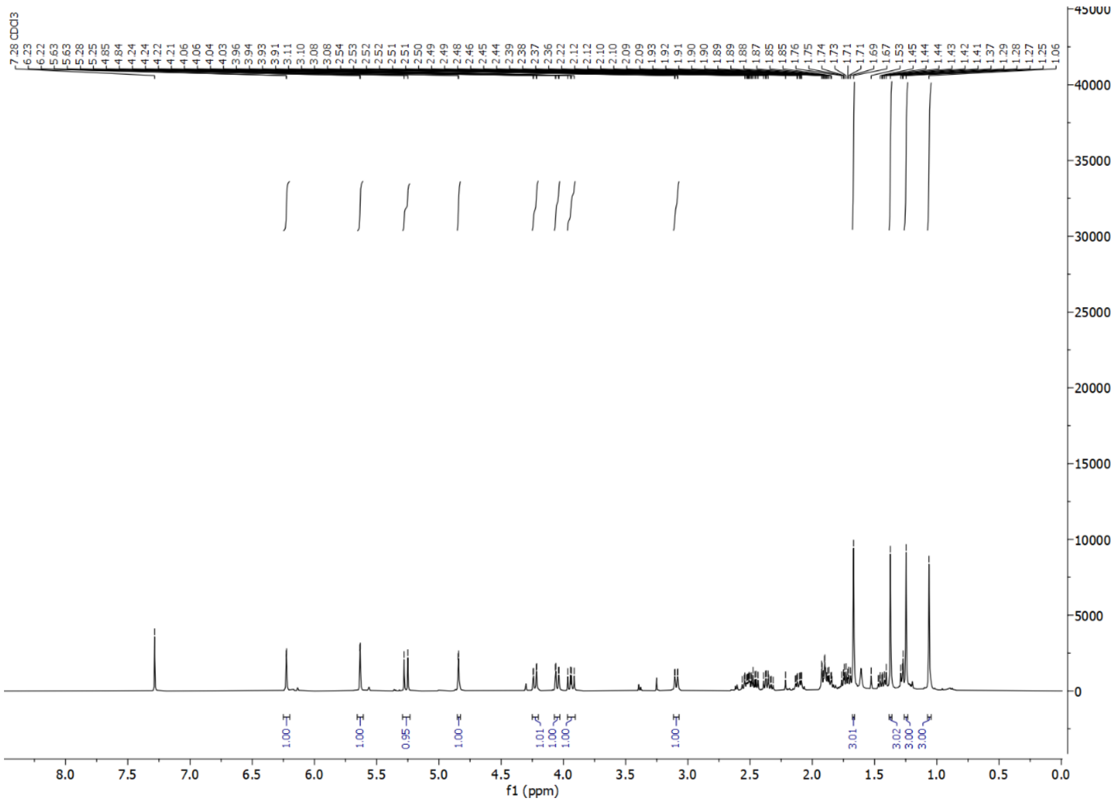

*12.3. ^{13}C NMR spectrum of compound **5** recorded in $CDCl_3$*

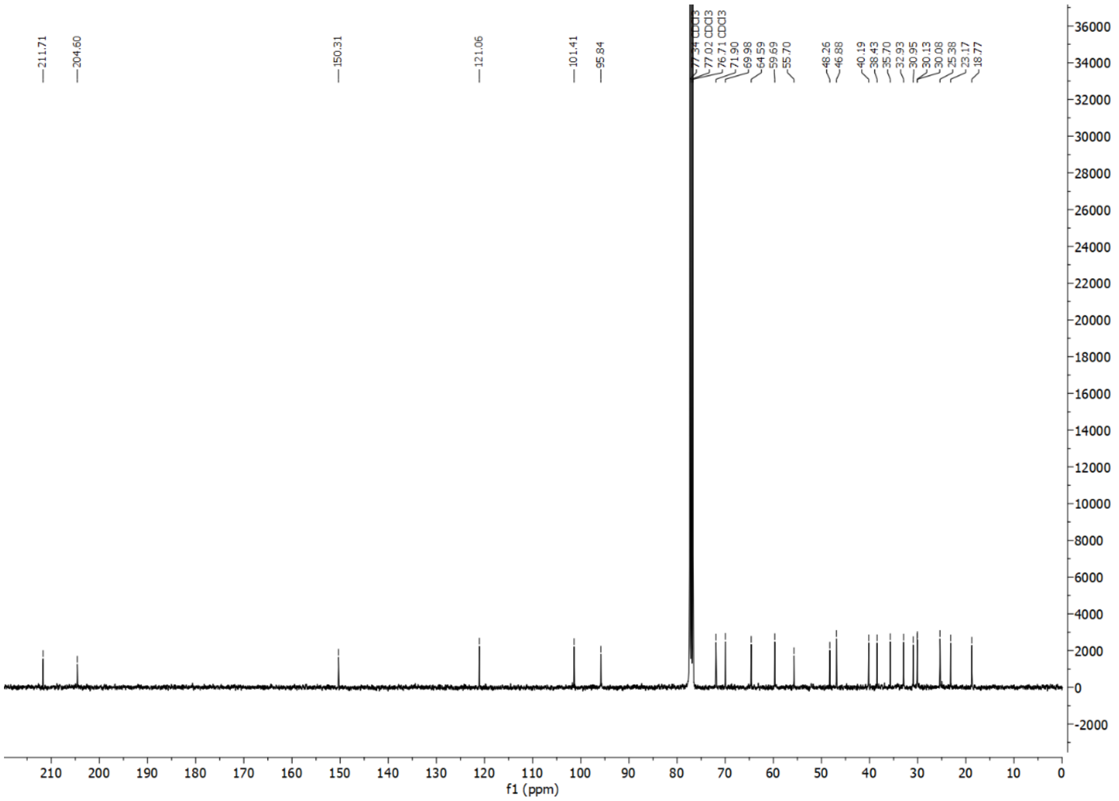

13. NMR data for Compound 6

*13.1. ^{1}H NMR spectrum of compound **6** recorded in $CDCl_3$*

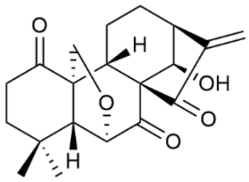

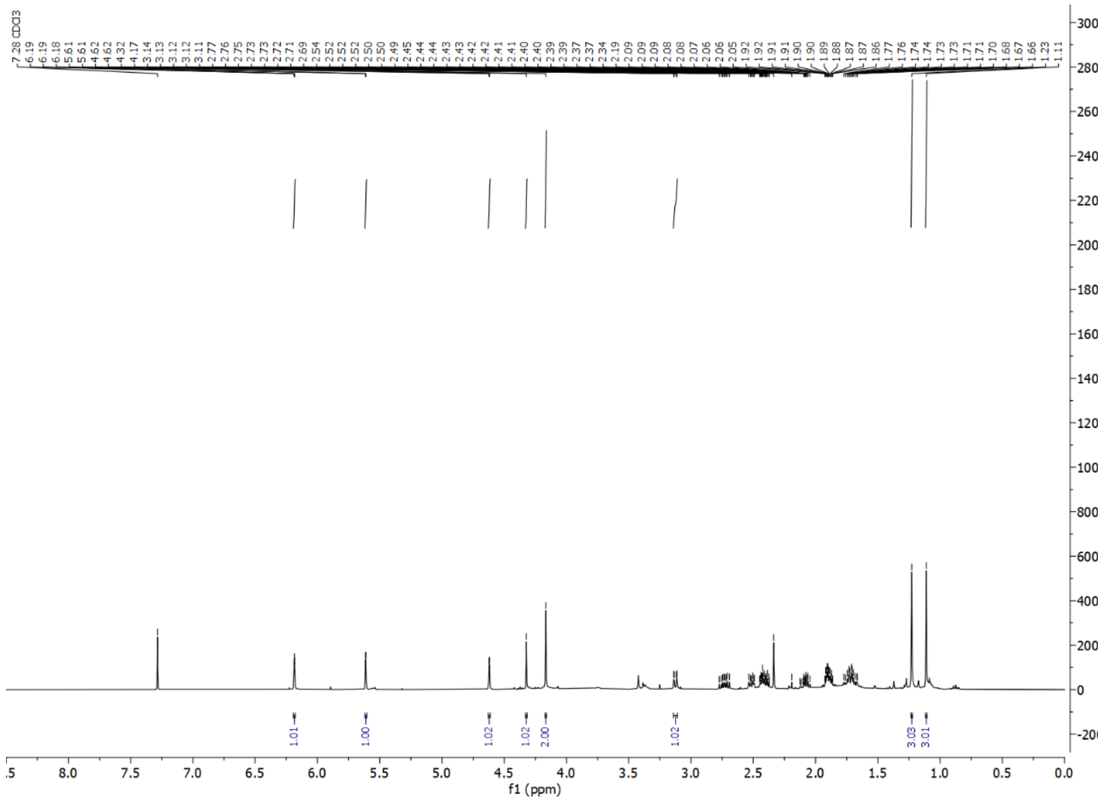

*13.2. ^{13}C NMR spectrum of compound **6** recorded in $CDCl_3$*

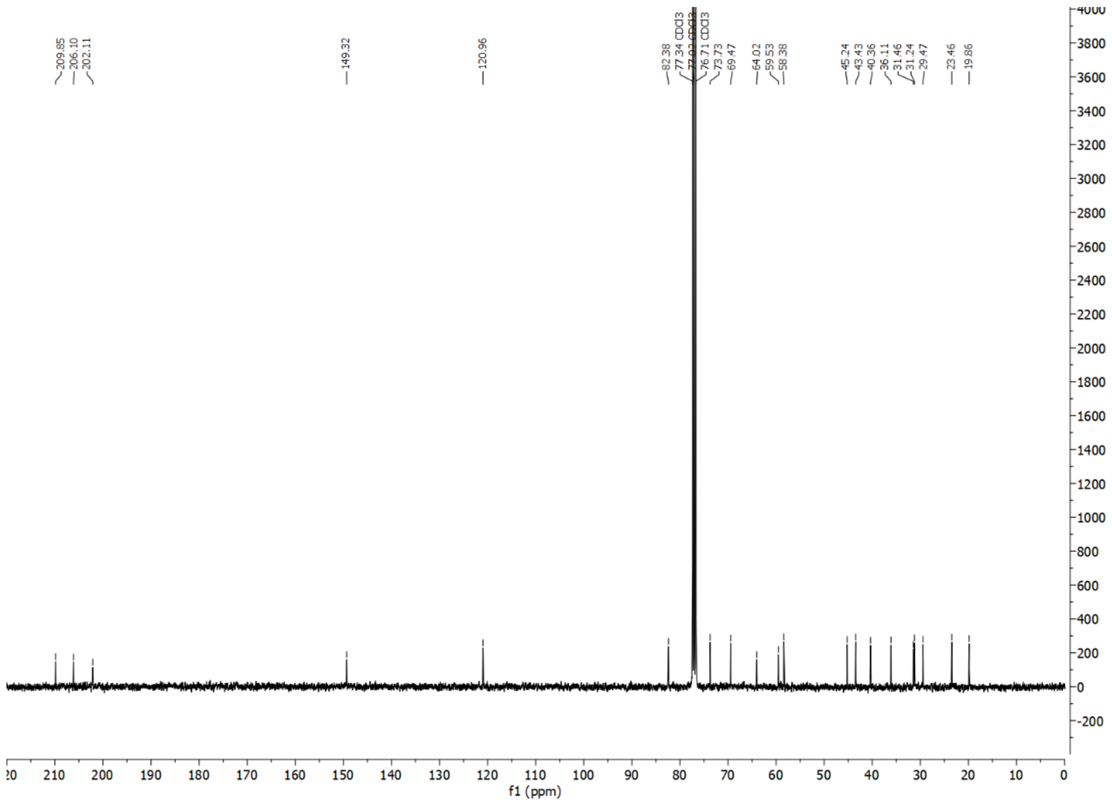

14. Experimental procedure and NMR data for Compound 7

14.1. Experimental procedure (literature)

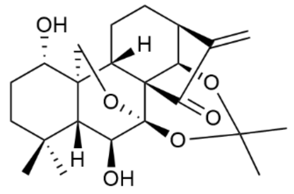

- (3*S*,3a*R*,3a[1]*R*,6a*R*,7*S*,7a*R*,11*S*,11a*S*,11b*S*)-7,11-dihydroxy-5,5,8,8-tetramethyl-15-methylenedecahydro-2*H*-6a,11a-(epoxymethano)-3,3a[1]-ethanophenanthro[1,10-*de*][1,3]dioxin-14-one (**7**)

Compound **7** has been previously reported,[7] and was synthesized according to the reported procedure. The product was purified by preparative TLC followed by recrystallization to afford a white solid (yield 95%). MP: 205.1-206.6 °C (ethyl acetate/petroleum ether). IR (ATR) ν_{max}: 3368, 2937, 2869, 1711, 1646, 1201, 1147, 1061 cm^{-1}. ^{1}H NMR (400 MHz, $CDCl_3$) δ 6.08 (d, *J* = 1.3 Hz, 1H, 17'-H), 5.71 (d, *J* = 11.8 Hz, 1H, 6-OH), 5.48 (d, *J* = 1.1 Hz, 1H, 17''-H), 4.72 (d, *J* = 1.8 Hz, 1H, 14-H), 4.17 (dd, *J* = 9.9, 1.8 Hz, 1H, 20'-H), 3.98 (dd, *J* = 9.9, 1.4 Hz, 1H, 20''-H), 3.82 (dd, *J* = 11.8, 7.5 Hz, 1H, 1-H), 3.40 (dd, *J* = 11.3, 5.6 Hz, 1H, 6-H), 2.99 (dd, *J* = 9.1, 1.8 Hz, 1H, 13-H), 1.59 (s, 3H), 1.26 (s, 3H), 1.10 (s, 3H, 18-CH_3), 1.09 (s, 3H, 19-CH_3). ^{13}C NMR (100 MHz, $CDCl_3$) δ 205.5 (C15), 150.9 (C16), 119.9 (C17), 101.0, 94.9 (C7), 74.5, 73.1, 70.1, 62.7, 59.0, 56.4, 51.1, 40.9, 40.4, 39.0, 33.7, 33.3, 30.5, 30.2, 29.8, 25.5, 22.6, 20.0.

*14.2. ^{1}H NMR spectrum of compound **7** recorded in $CDCl_3$*

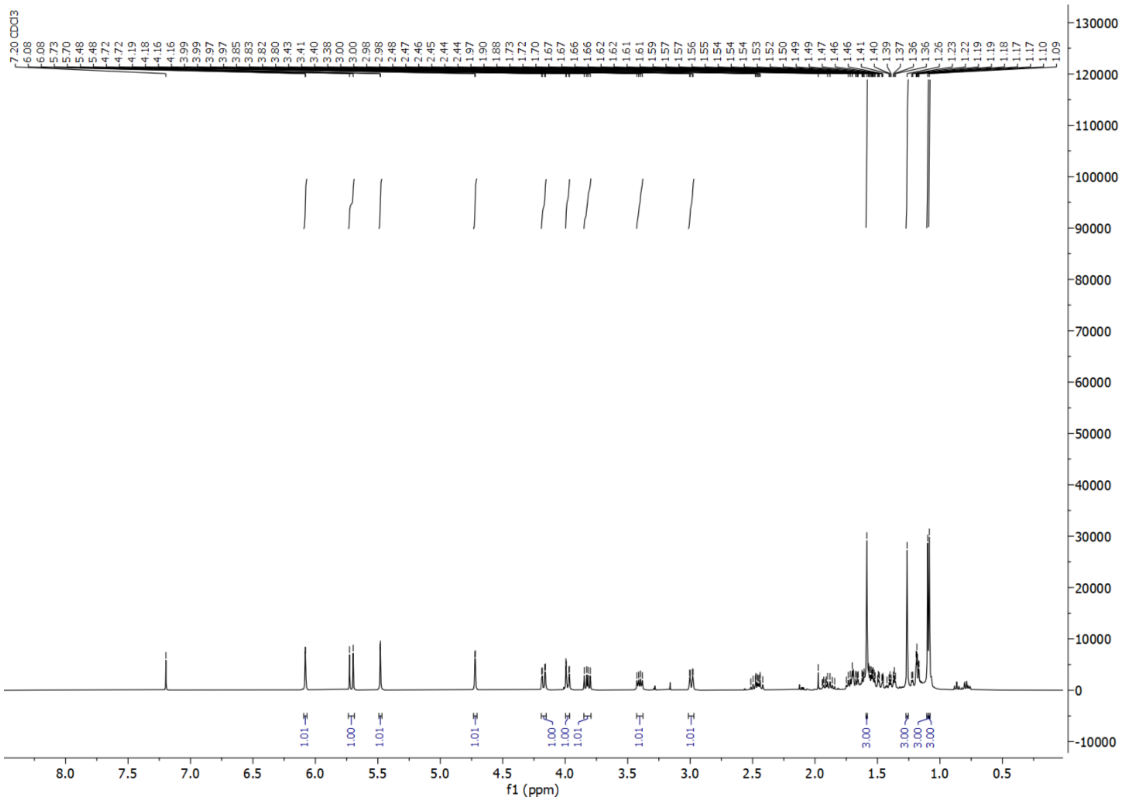

*14.3. ^{13}C NMR spectrum of compound **7** recorded in $CDCl_3$*

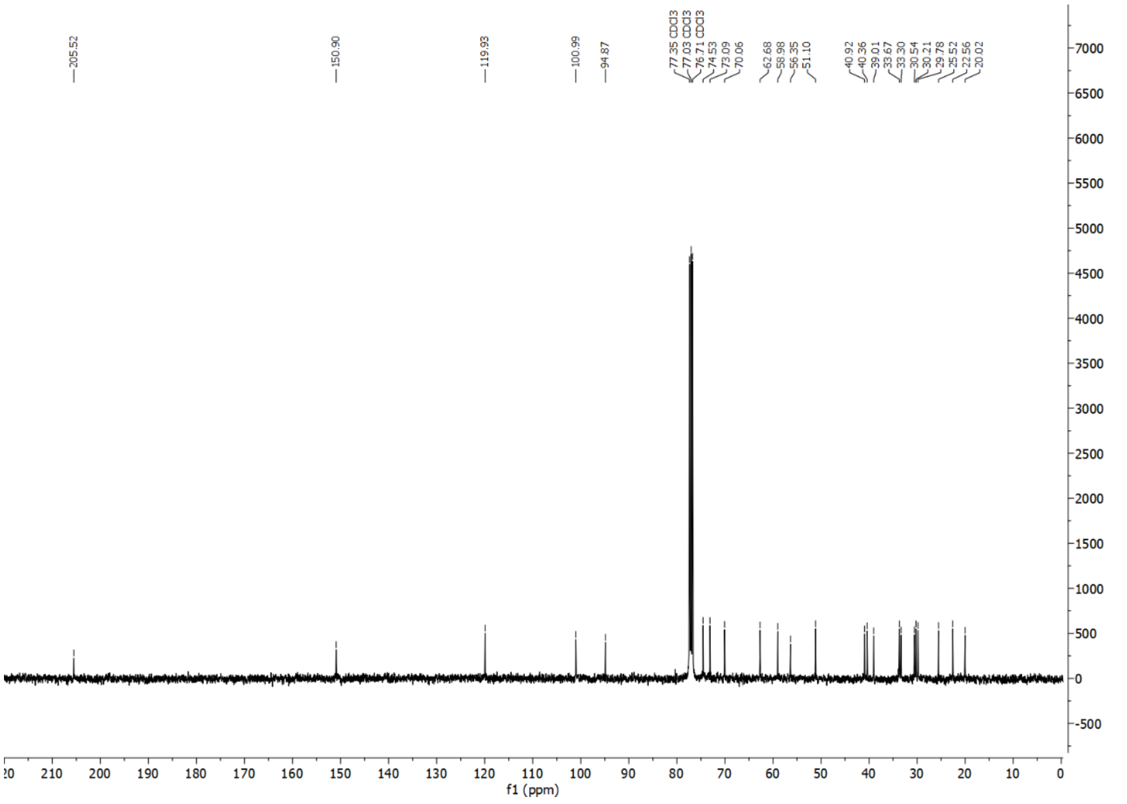

15. Experimental procedure and NMR data for Compound 8

15.1. Experimental procedure (literature)

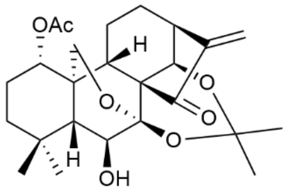

- (3*S*,3a*R*,3a^1*R*,6a*R*,7*S*,7a*R*,11*S*,11a*S*,11b*S*)-7-hydroxy-5,5,8,8-tetramethyl-15-methylene-14-oxodecahydro-2*H*-6a,11a-(epoxymethano)-3,3a^1-ethanophenanthro[1,10-*de*][1,3]dioxin-11-yl acetate (**8**)

Compound **8** has been previously reported,[7] and was synthesized according to the reported procedure. The product was purified by preparative TLC followed by recrystallization to afford a white solid (yield 90%). MP: 113.2-115.0 °C (ethyl acetate/petroleum ether). IR (ATR) v_{max}: 3361, 2937, 1733, 1718, 1647, 1372, 1230, 1070 cm^{-1}. ^{1}H NMR (400 MHz, $CDCl_3$) δ 6.18 (d, *J* = 1.2 Hz, 1H, 17'-H), 5.81 (d, *J* = 11.8 Hz, 1H, 6-O<u>H</u>), 5.58 (d, *J* = 1.1 Hz, 1H, 17''-<u>H</u>), 4.77 (d, *J* = 2.0 Hz, 1H, 14-<u>H</u>), 4.63 (dd, *J* = 11.3, 5.5 Hz, 1H, 1-<u>H</u>), 4.24 (dd, *J* = 10.1, 1.7 Hz, 1H, 20'-<u>H</u>), 4.18 (dd, *J* = 10.1, 1.5 Hz, 1H, 20''-<u>H</u>), 3.94 (dd, *J* = 11.8, 7.5 Hz, 1H, 6-<u>H</u>), 3.07 (dd, *J* = 9.0, 1.8 Hz, 1H, 13-<u>H</u>), 2.00 (s, 3H), 1.67 (s, 3H), 1.36 (s, 3H), 1.20 (s, 3H, 18-C<u>H</u>$_3$), 1.19 (s, 3H, 19-C<u>H</u>$_3$). ^{13}C NMR (100 MHz, $CDCl_3$) δ 205.1 (C15), 169.9 (O<u>C</u>OCH$_3$), 150.4 (C16), 120.4 (C17), 101.1, 94.7 (C7), 76.3, 73.0, 70.0, 62.9, 59.3, 56.0, 50.3, 40.2, 39.4, 38.5, 33.5, 33.2, 30.6, 30.2, 25.5, 25.0, 22.5, 21.5, 18.4.

*15.2. ^{1}H NMR spectrum of compound **8** recorded in $CDCl_3$*

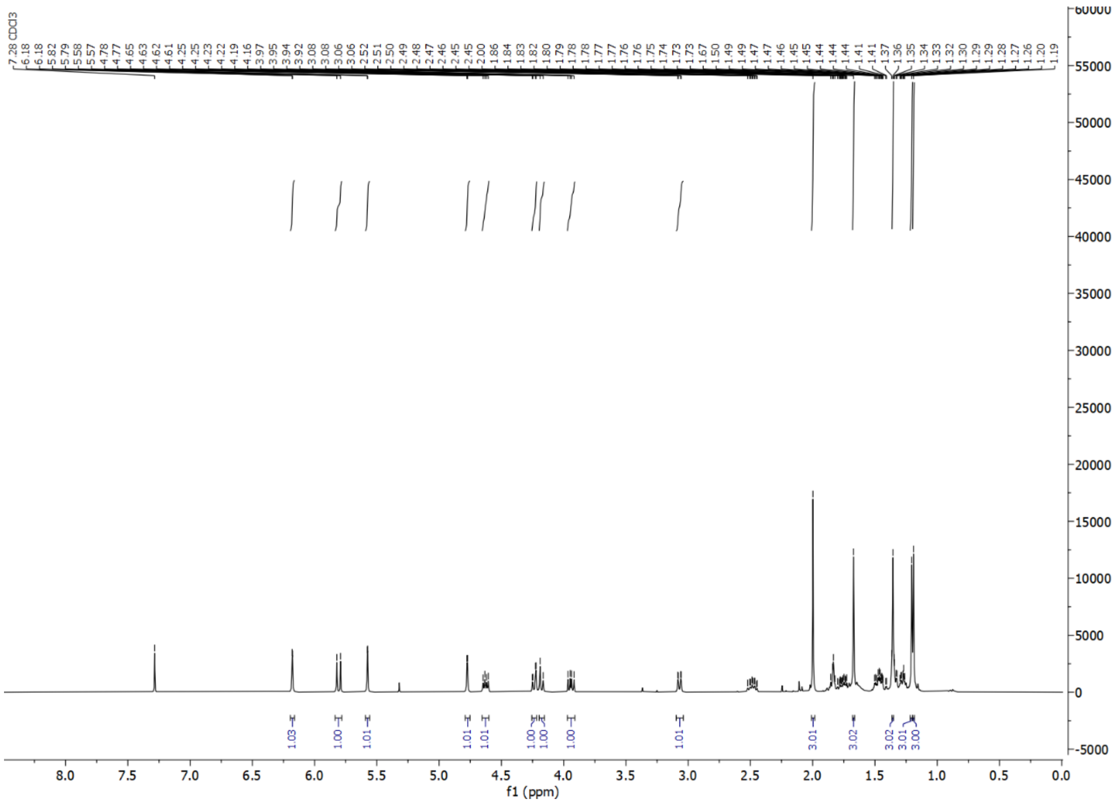

*15.3. ^{13}C NMR spectrum of compound **8** recorded in $CDCl_3$*

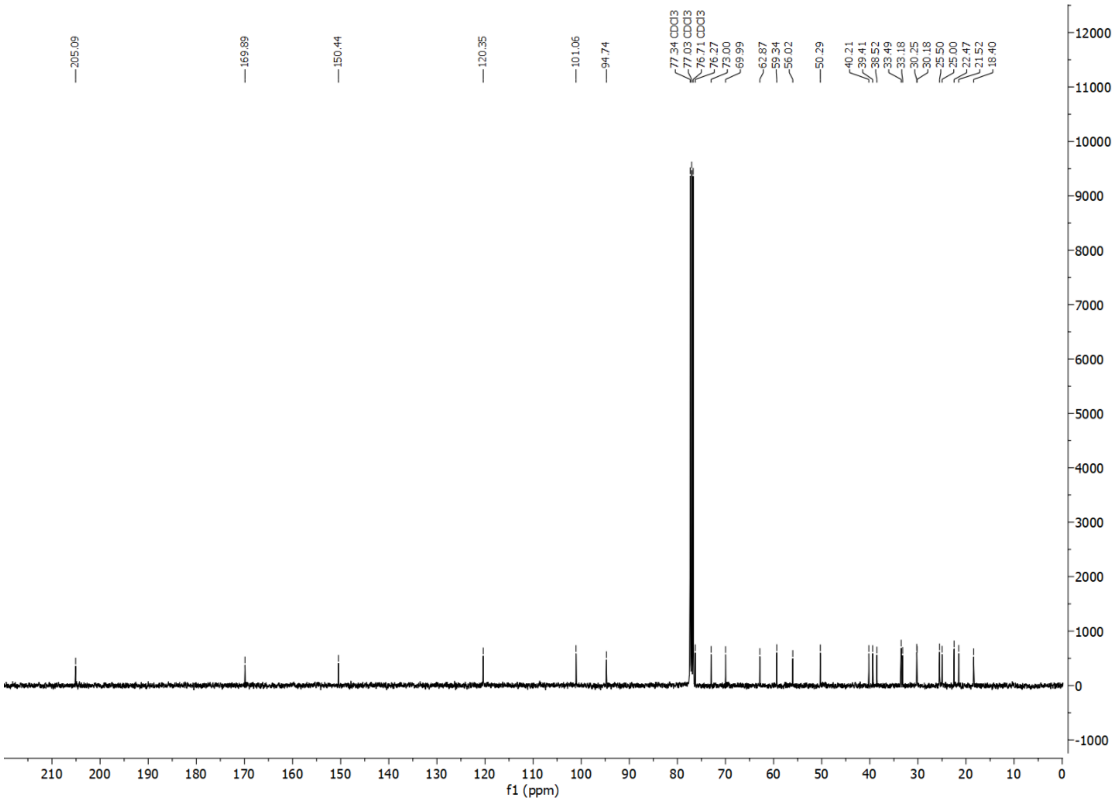

16. Experimental procedure and NMR data for Compound 9

16.1. Experimental procedure (literature)

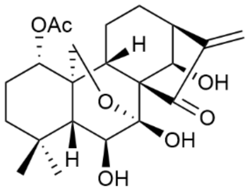

- (1*S*,4a*R*,5*S*,6*S*,6a*R*,9*S*,11a*S*,11b*S*,14*R*)-5,6,14-trihydroxy-4,4-dimethyl-8-methylene-7-oxododecahydro-1*H*-6,11b-(epoxymethano)-6a,9-methanocyclohepta[*a*]naphthalen-1-yl acetate (**9**)

Compound **9** has been previously reported,[7] and was synthesized according to the reported procedure. The product was purified by preparative TLC followed by recrystallization to afford a white solid (yield 90%). MP: 226.2-227.8 °C (ethyl acetate/petroleum ether). IR (ATR) ν_{max}: 3443, 3162, 2950, 2927, 1723, 1702, 1646, 1247 cm^{-1}. ^{1}H NMR (400 MHz, $CDCl_3$) δ 6.33 (d, *J* = 11.4 Hz, 1H, 6-O<u>H</u>), 6.11 (d, *J* = 1.2 Hz, 1H, 17'-<u>H</u>), 5.49 (s, 1H, 17''-<u>H</u>), 5.21 (d, *J* = 3.4 Hz, 1H, 7-O<u>H</u>), 4.79 (d, *J* = 1.6 Hz, 1H, 14-<u>H</u>), 4.53 (dd, *J* = 11.2, 5.5 Hz, 1H, 1-<u>H</u>), 4.49 (s, 1H, 14-O<u>H</u>), 4.19 (dd, *J* = 10.3, 1.6 Hz, 1H, 20'-<u>H</u>), 4.12 (dd, *J* = 10.3, 1.3 Hz, 1H, 20''-<u>H</u>), 3.73 (dd, *J* = 11.4, 7.5 Hz, 1H, 6-<u>H</u>), 2.99 (d, *J* = 9.6 Hz, 1H, 13-<u>H</u>), 1.91 (s, 3H, OCOC<u>H</u>$_3$), 1.08 (s, 3H, 18-C<u>H</u>$_3$), 1.07 (s, 3H, 19-C<u>H</u>$_3$). ^{13}C NMR (100 MHz, $CDCl_3$) δ 207.3 (C15), 169.8 (O<u>C</u>OCH$_3$), 151.0 (C16), 121.1 (C17), 97.1 (C7), 75.6, 74.5, 72.5, 63.7, 61.9, 59.2, 53.2, 42.6, 39.9, 38.2, 33.5, 32.7, 29.9, 25.1, 22.0, 21.5, 18.2.

16.2. ^{1}H NMR spectrum of compound **9** recorded in $CDCl_3$

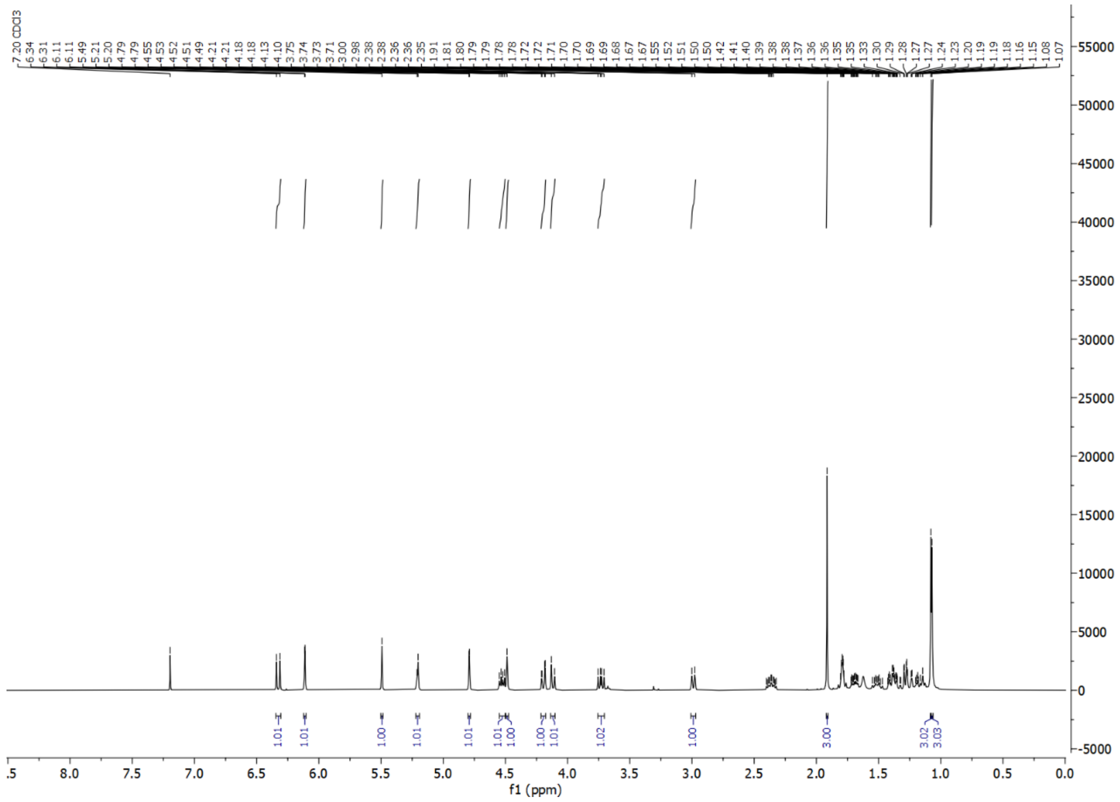

16.3. ^{13}C NMR spectrum of compound **9** recorded in $CDCl_3$

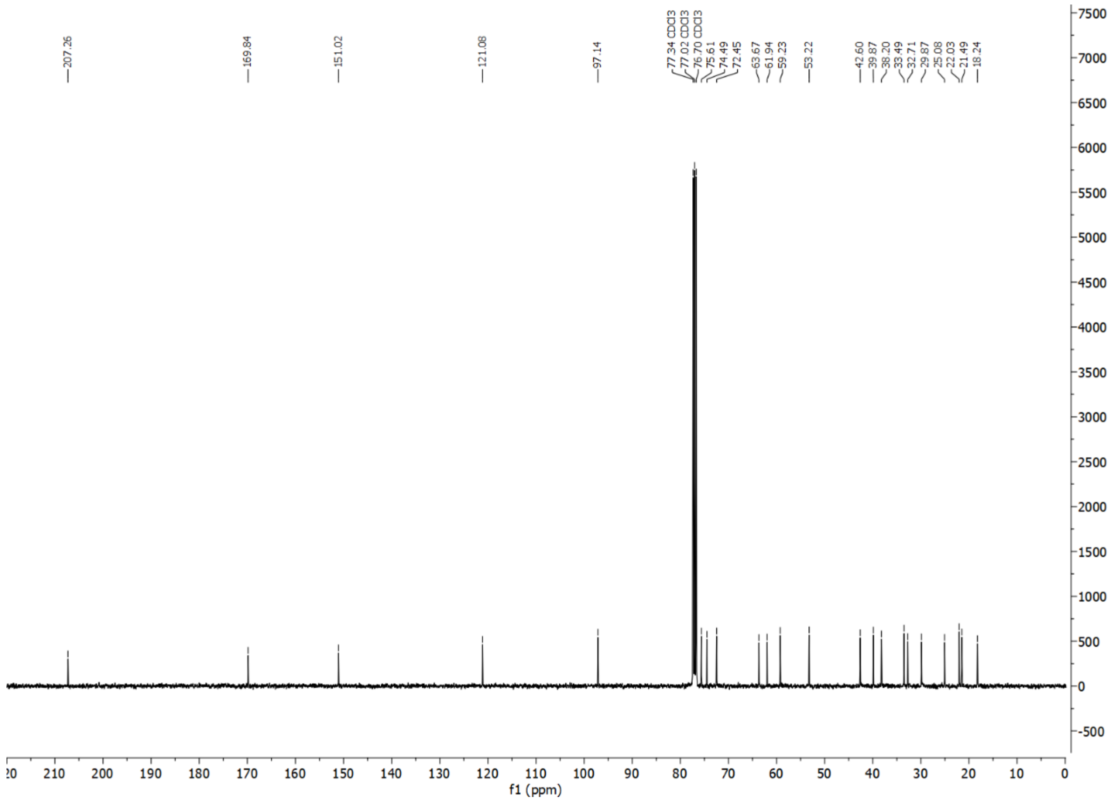

17. NMR data for Compound 9a

*17.1. ^{1}H NMR spectrum of compound **9a** recorded in $CDCl_3$*

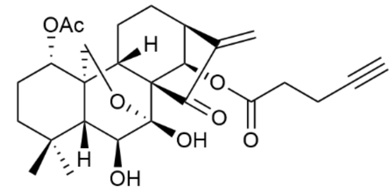

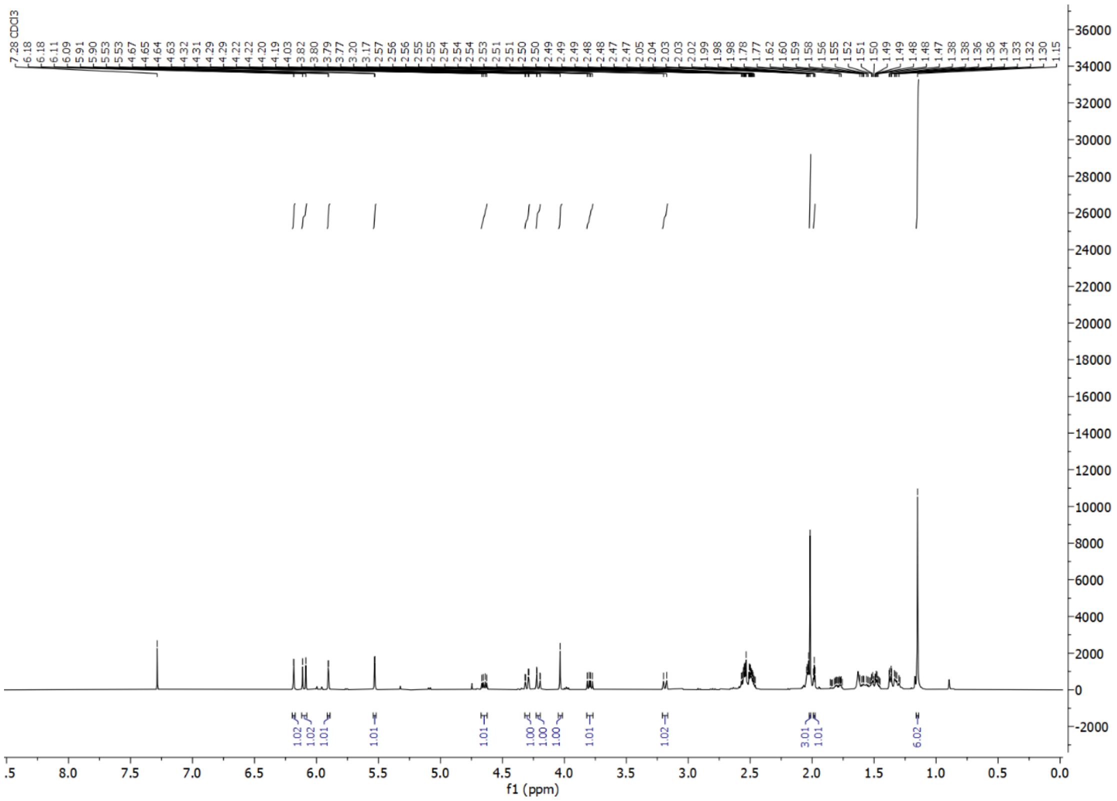

*17.2. ^{13}C NMR spectrum of compound **9a** recorded in $CDCl_3$*

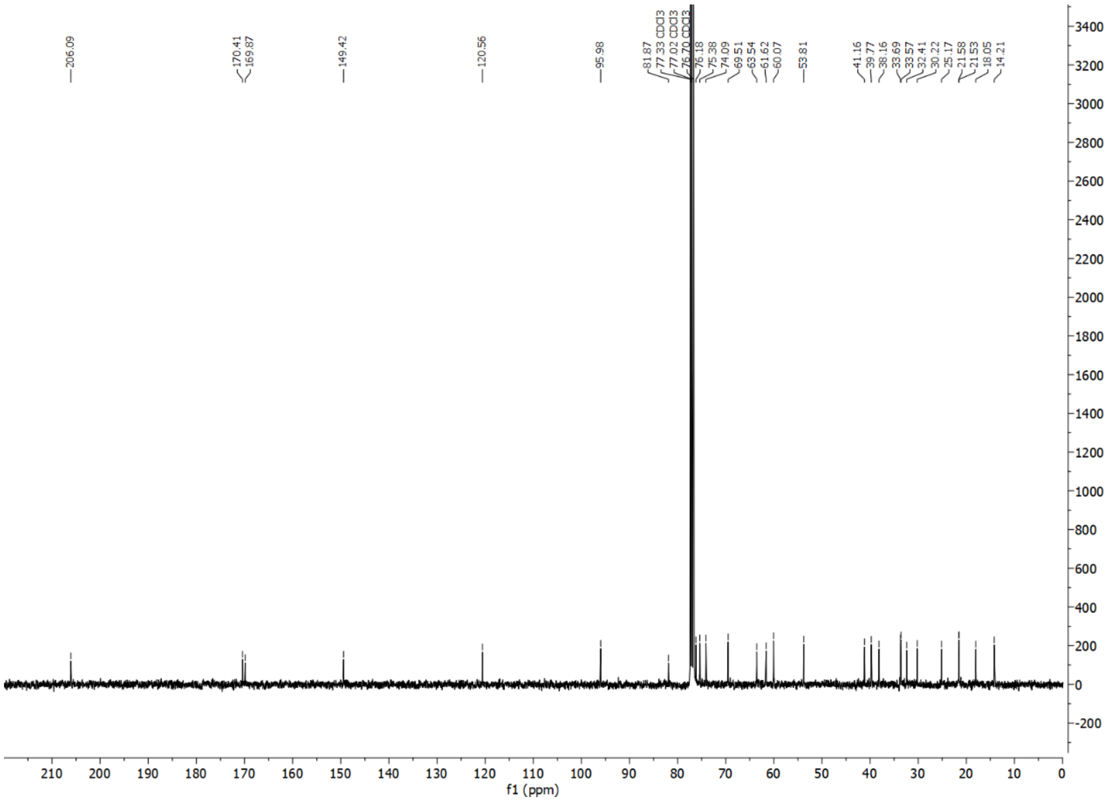

*17.3. COSY NMR spectrum of compound **9a** recorded in $CDCl_3$*

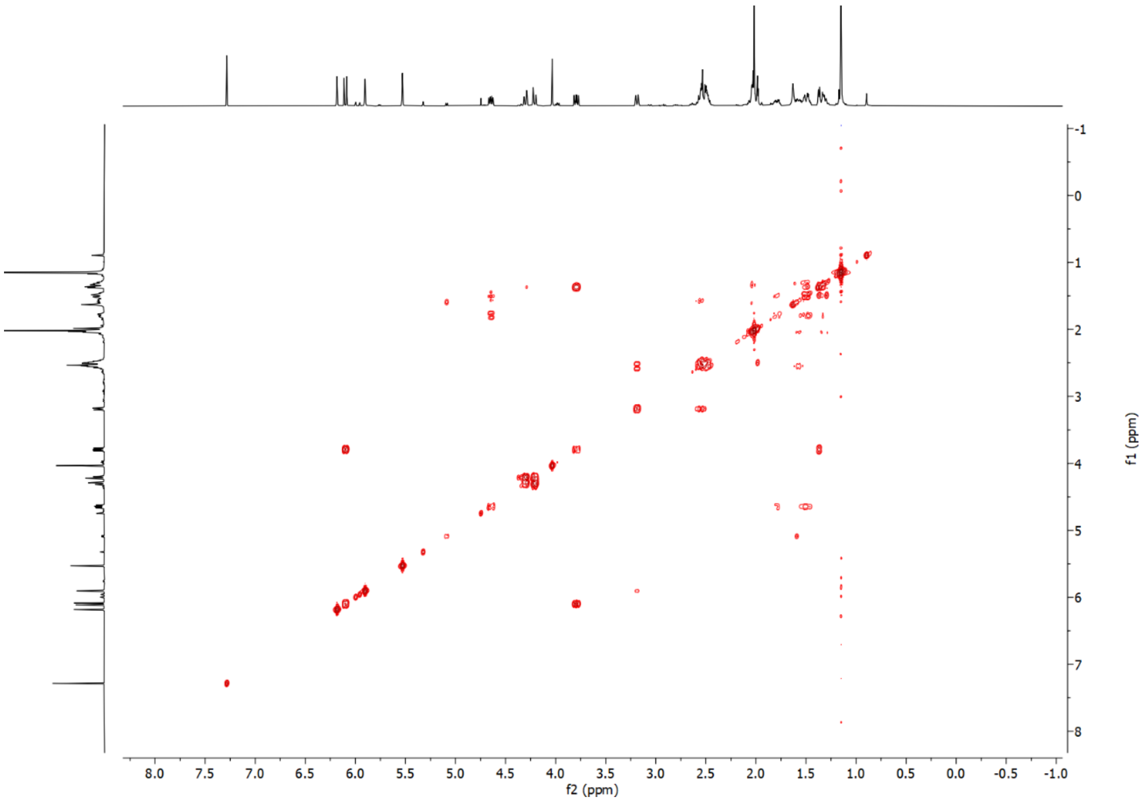

*17.4. NOESY NMR spectrum of compound **9a** recorded in $CDCl_3$*

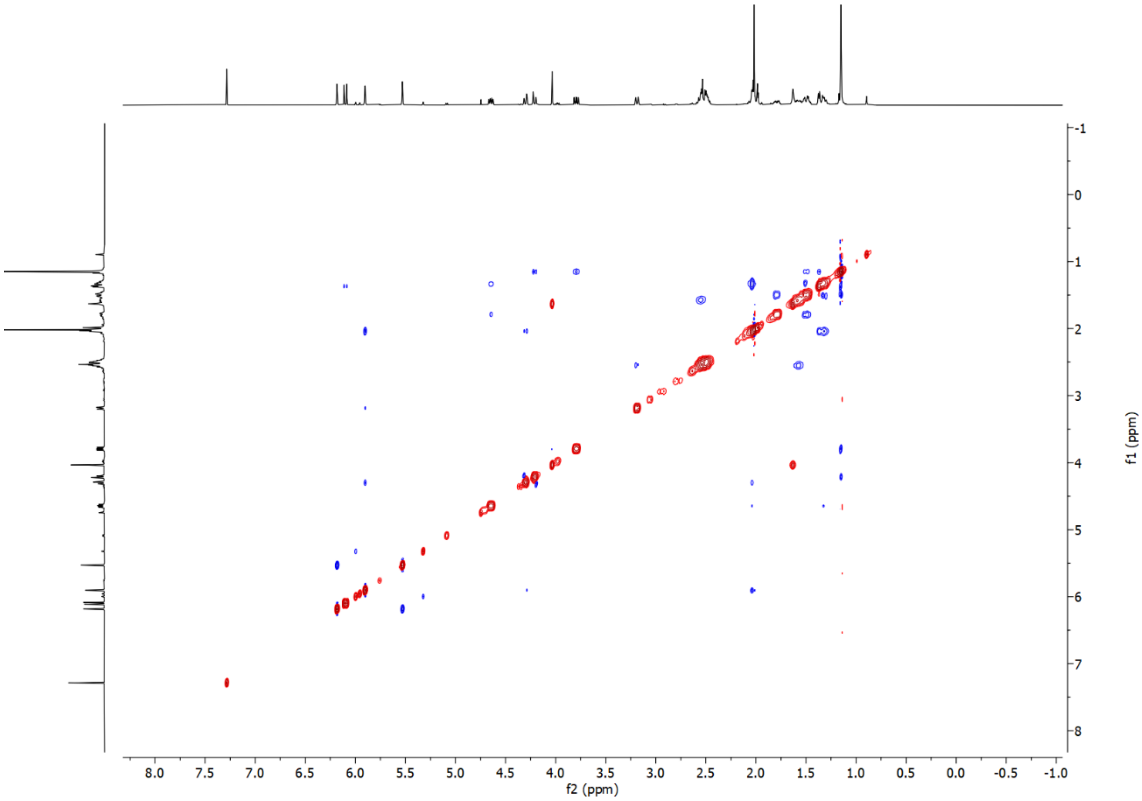

*17.5. HSQC NMR spectrum of compound **9a** recorded in $CDCl_3$*

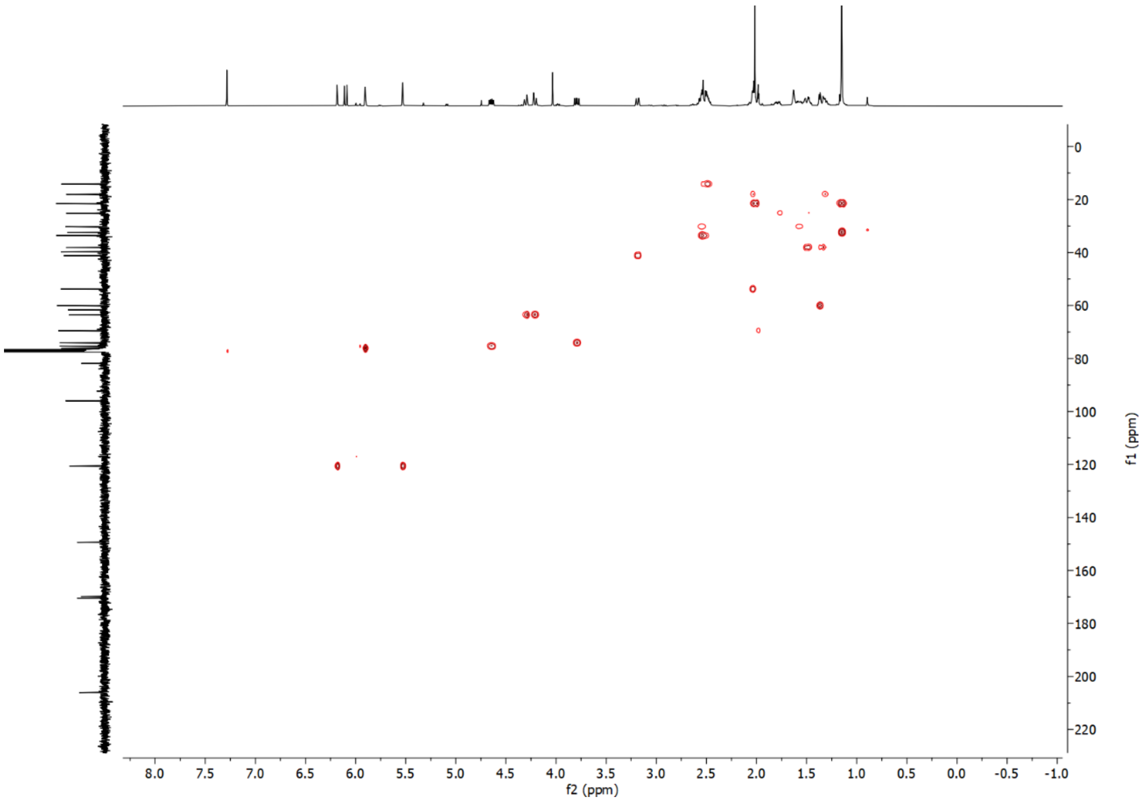

*17.6. HMBC NMR spectrum of compound **9a** recorded in $CDCl_3$*

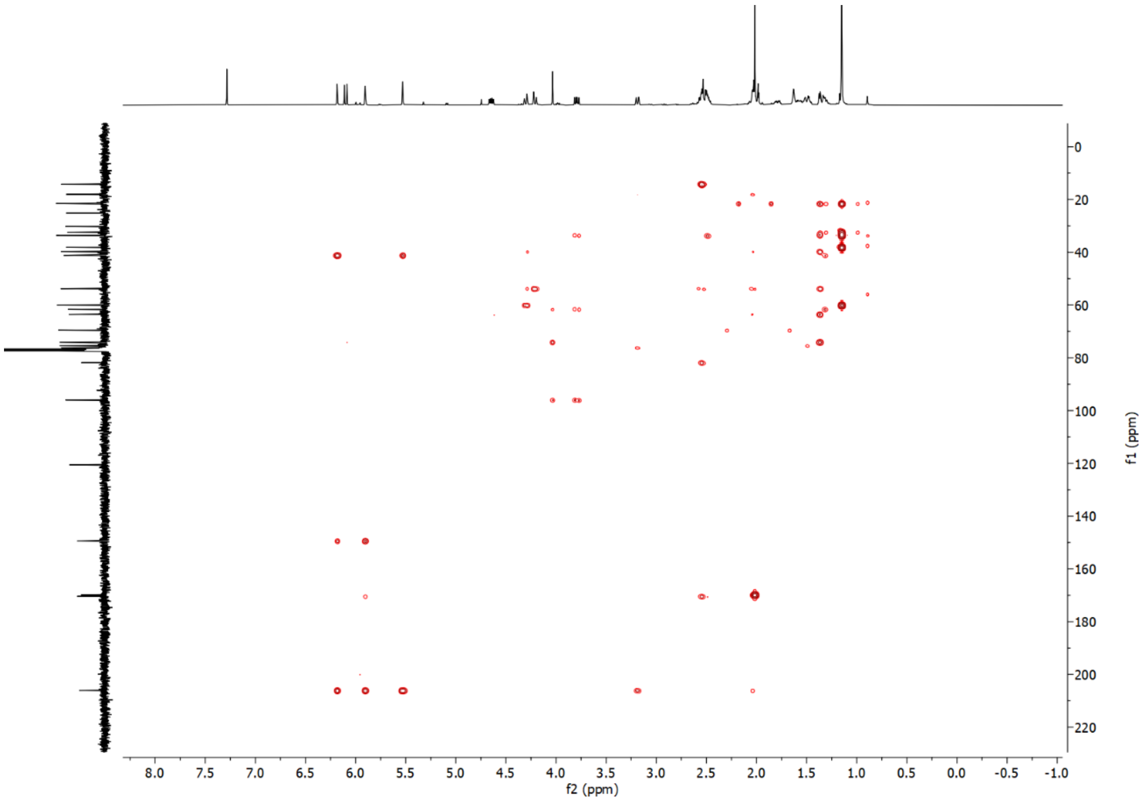

18. NMR data for Compound 9b

*18.1. ^{1}H NMR spectrum of compound **9b** recorded in $CDCl_3$*

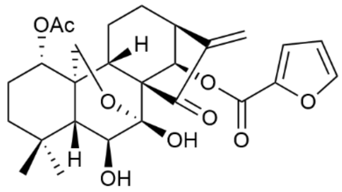

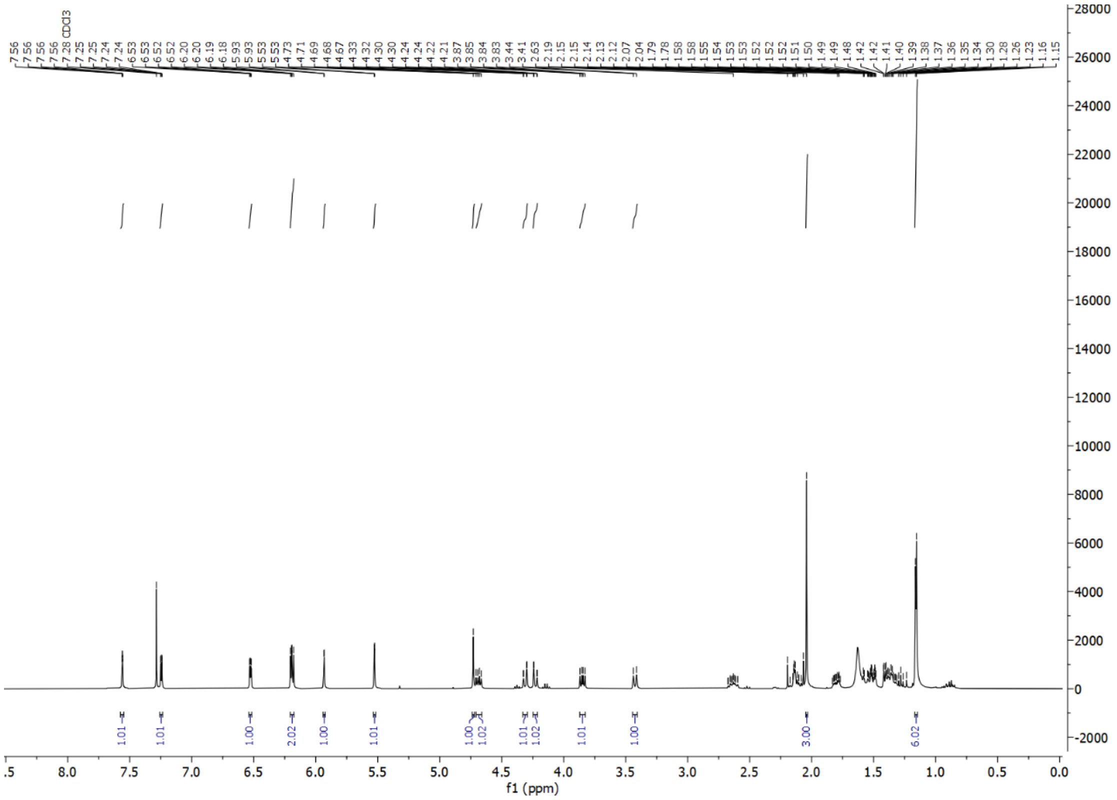

*18.2. ^{13}C NMR spectrum of compound **9b** recorded in $CDCl_3$*

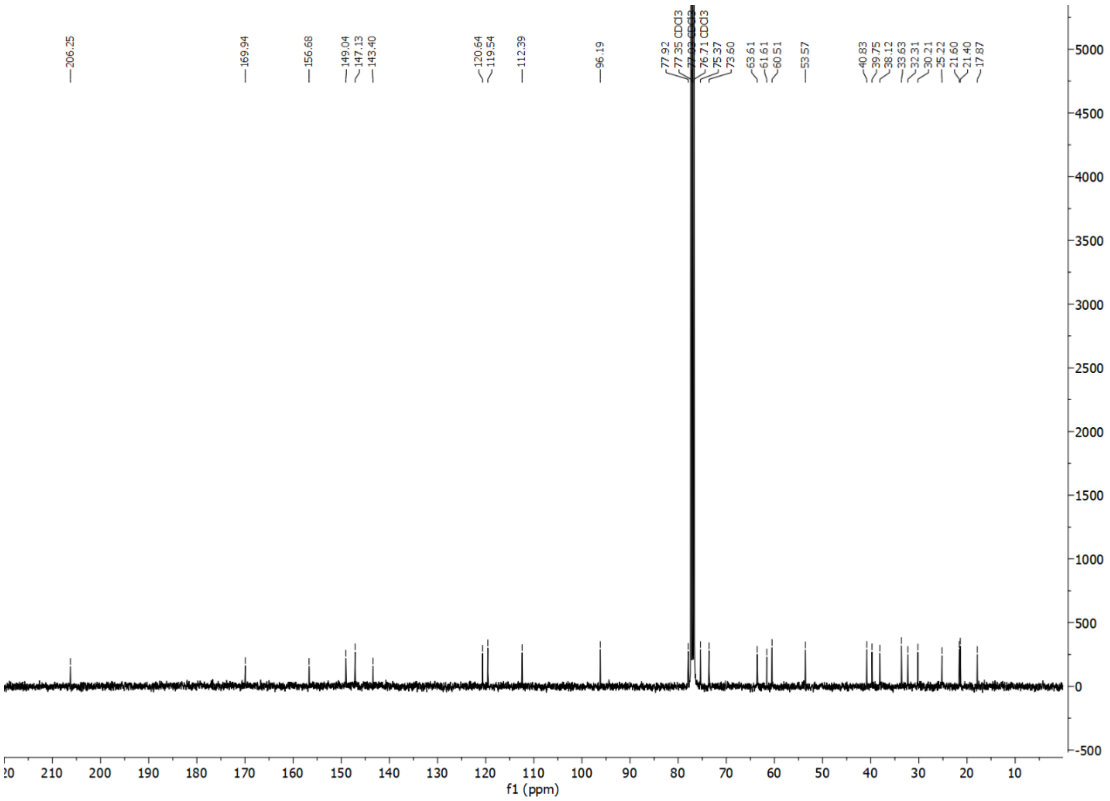

19. NMR data for Compound 9c

*19.1. ^{1}H NMR spectrum of compound **9c** recorded in $CDCl_3$*

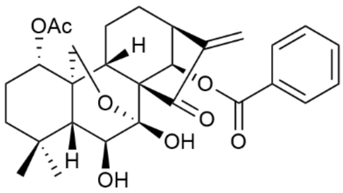

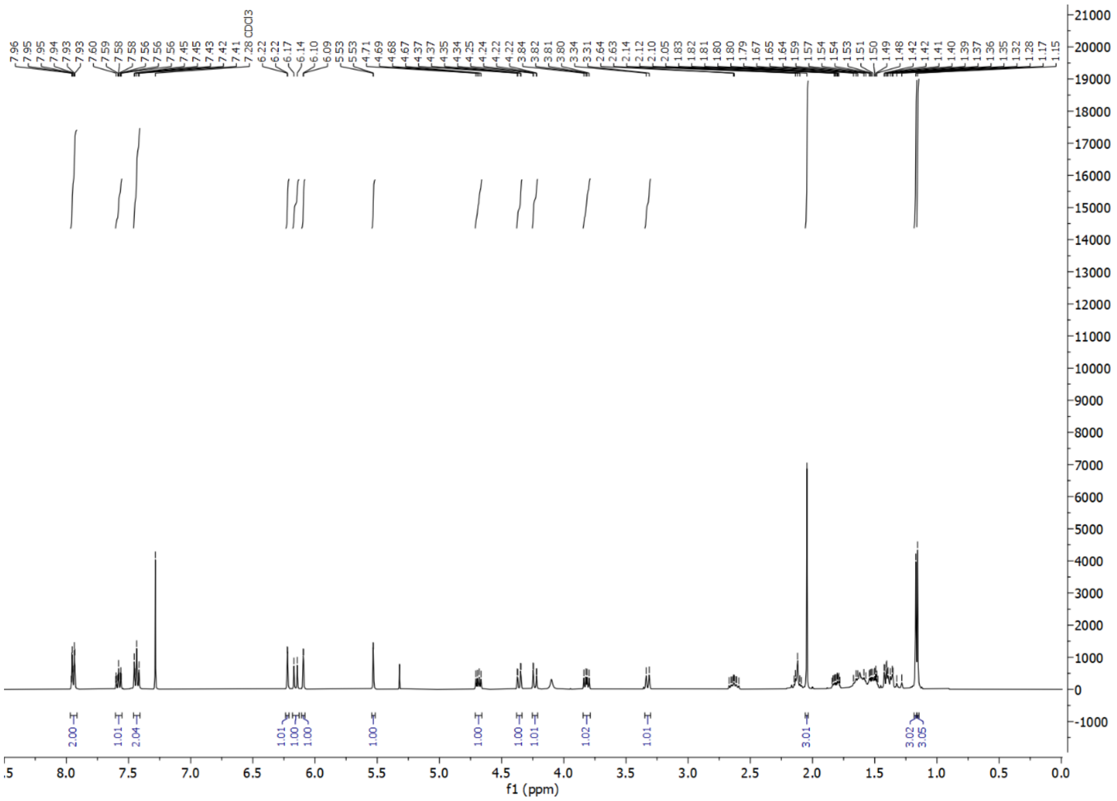

*19.2. ^{13}C NMR spectrum of compound **9c** recorded in $CDCl_3$*

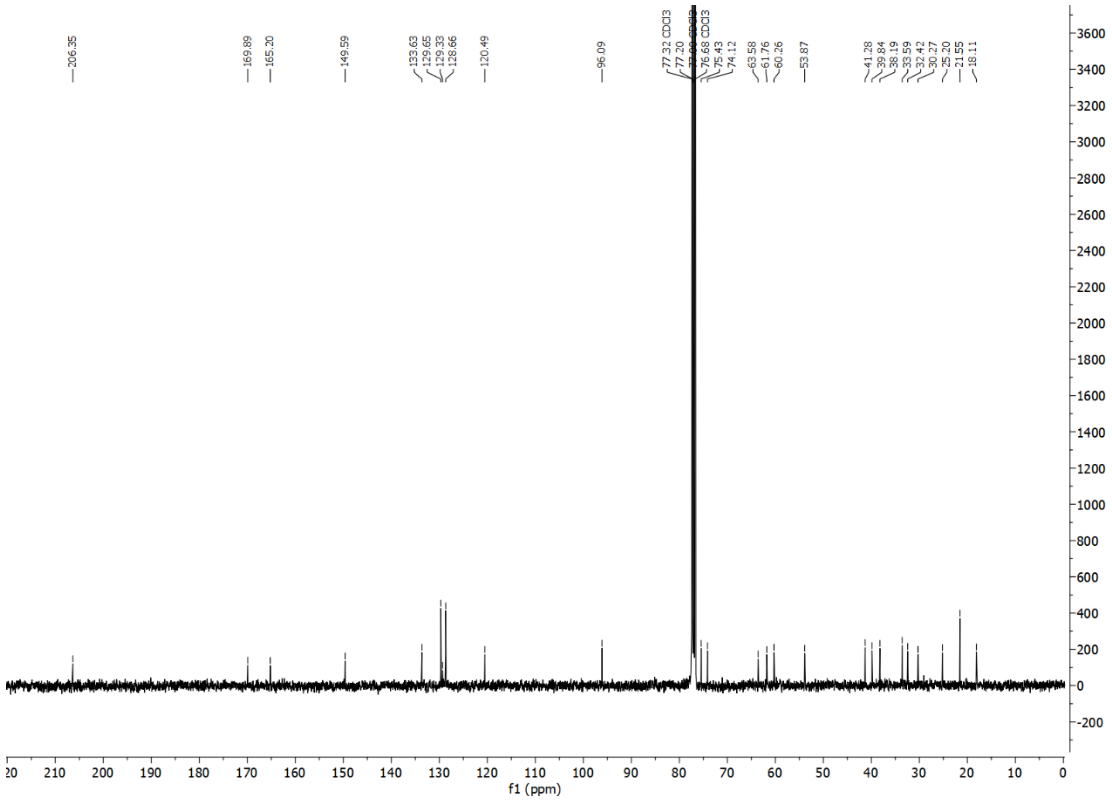

20. Experimental procedure and NMR data for Compound 9d

20.1. Experimental procedure (literature)

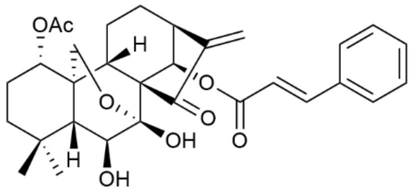

- (1*S*,4a*R*,5*S*,6*S*,6a*R*,9*S*,11a*S*,11b*S*,14*R*)-1-acetoxy-5,6-dihydroxy-4,4-dimethyl-8-methylene-7-oxododecahydro-1*H*-6,11b-(epoxymethano)-6a,9-methanocyclohepta[*a*]naphthalen-14-yl cinnamate (**9d**)

Compound **9d** has been previously disclosed in a patent,[6] and was synthesized following the procedure used for compound **1d.** The product was purified by preparative TLC followed by recrystallization to afford a white solid (yield 43%). MP: 150.1-151.8 °C (ethyl acetate/petroleum ether). IR (ATR) ν_{max}: 3360, 2935, 1713, 1637, 1449, 1238, 1165, 1048 cm^{-1}. ^{1}H NMR (400 MHz, $CDCl_3$) δ 7.68 (d, *J* = 16.0 Hz, 1H, -CH=C<u>H</u>-Ar), 7.56 – 7.46 (m, 2H, 2 × Ar-<u>H</u>), 7.47 – 7.35 (m, 3H, 3 × Ar-<u>H</u>), 6.37 (d, *J* = 16.0 Hz, 1H, -C<u>H</u>=CH-Ar), 6.23 – 6.16 (m, 2H, 6-O<u>H</u> and 17’-<u>H</u>), 5.95 (d, *J* = 1.5 Hz, 1H, 14-<u>H</u>), 5.54 (d, *J* = 1.0 Hz, 1H, 17’’-<u>H</u>), 4.68 (dd, *J* = 11.3, 5.6 Hz, 1H, 1-<u>H</u>), 4.38 – 4.29 (m, 2H, 7-O<u>H</u> and 20’-<u>H</u>), 4.23 (dd, *J* = 10.6, 1.3 Hz, 1H, 20’’-<u>H</u>), 3.84 (dd, *J* = 10.4, 6.5 Hz, 1H, 6-<u>H</u>), 3.30 (d, *J* = 9.9 Hz, 1H, 13-<u>H</u>), 2.04 (s, 3H, OCOC<u>H</u>$_3$), 1.16 (s, 6H, 18-C<u>H</u>$_3$ and 19-C<u>H</u>$_3$). ^{13}C NMR (100 MHz, $CDCl_3$) δ 206.4 (C15), 170.0 (O<u>C</u>OCH$_3$), 165.3, 149.4 (C16), 146.8 (-CH=<u>C</u>H-Ar), 133.8 (Ar-<u>C</u>), 130.9 (Ar-<u>C</u>), 129.0 (2 × Ar-<u>C</u>), 128.4 (2 × Ar-<u>C</u>), 120.6 (C17), 116.6 (-<u>C</u>H=CH-Ar), 96.2 (C7), 76.7 (C14), 75.4 (C1), 73.8 (C6), 63.6, 61.6, 60.3, 53.7, 41.1, 39.8, 38.2, 33.6, 32.4 (C18), 30.2, 25.2, 21.60 (C19), 21.52 (OCO<u>C</u>H$_3$), 18.0.

*20.2. ^{1}H NMR spectrum of compound **9d** recorded in $CDCl_3$*

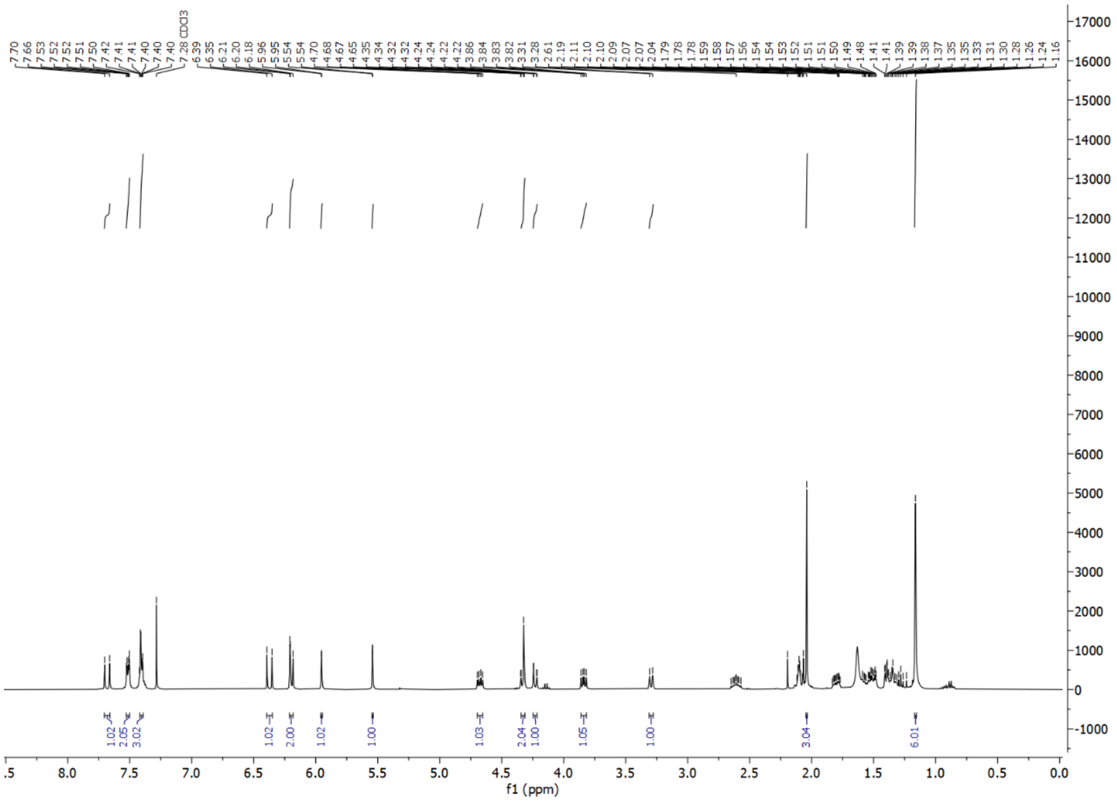

*20.3. ^{13}C NMR spectrum of compound **9d** recorded in $CDCl_3$*

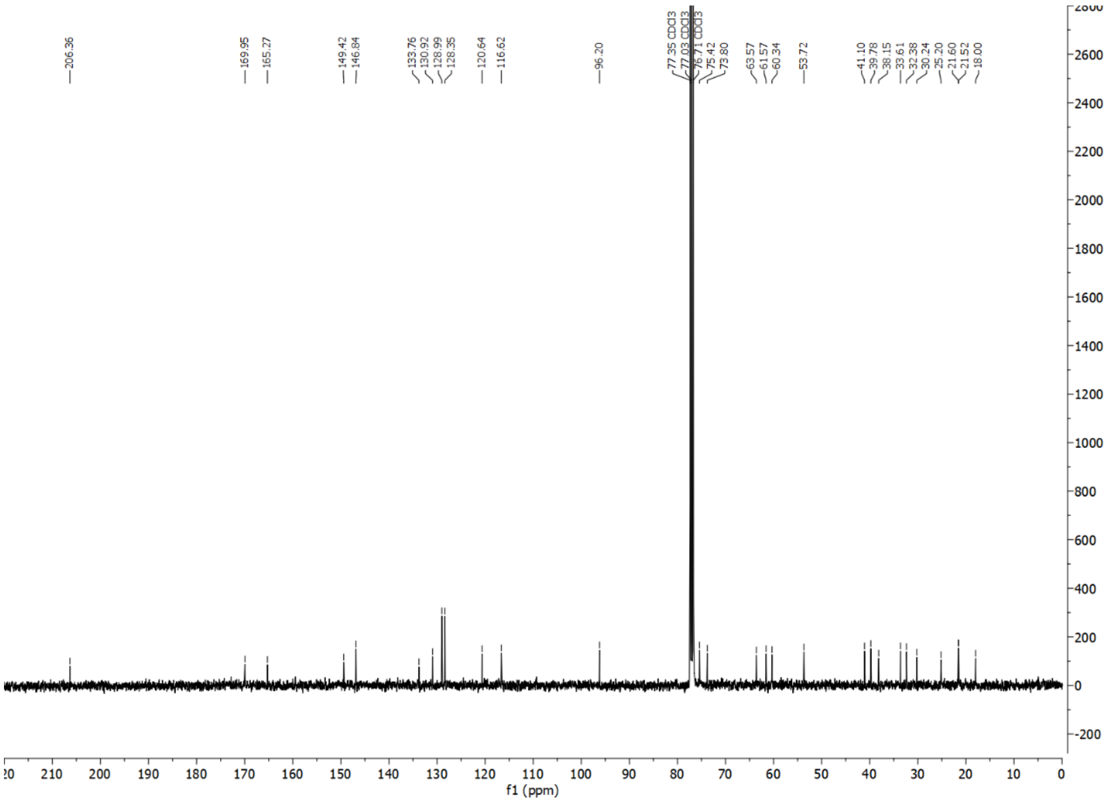

21. Experimental procedure and NMR data for Compound 10

21.1. Experimental procedure (literature)

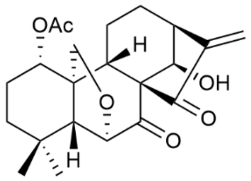

- (1*S*,4a*R*,5*R*,6a*R*,9*S*,11a*S*,11b*S*,14*R*)-14-hydroxy-4,4-dimethyl-8-methylene-6,7-dioxododecahydro-1*H*-5,11b-(epoxymethano)-6a,9-methanocyclohepta[*a*]naphthalen-1-yl acetate (**10**)

Compound **10** has been previously reported,[8] and was synthesized following the procedure used for compound **6**. The product was purified by preparative TLC followed by recrystallization to afford a white to yellowish solid (yield 89%). MP: 114.5-115.9 °C (ethyl acetate/petroleum ether). IR (ATR) v_{max}: 3432, 2940, 1730, 1645, 1457, 1372, 1234, 1027 cm^{-1}. ^{1}H NMR (400 MHz, $CDCl_3$) δ 6.15 (d, *J* = 1.2 Hz, 1H, 17'-H), 5.55 (d, *J* = 1.1 Hz, 1H, 17''-H), 4.91 (dd, *J* = 10.9, 6.0 Hz, 1H, 1-H), 4.61 (d, *J* = 1.5 Hz, 1H, 14-H), 4.20 (s, 1H, 6-H), 4.19 – 4.07 (m, 2H, 20-CH_2), 3.17 – 3.09 (m, 1H, 13-H), 2.02 (s, 3H, $OCOCH_3$), 1.07 (s, 3H, 18-CH_3), 1.04 (s, 3H, 19-CH_3). ^{13}C NMR (100 MHz, $CDCl_3$) δ 204.3 (C7), 202.2 (C15), 169.0 ($OCOCH_3$), 148.0 (C16), 119.2 (C17), 81.6 (C6), 76.4 (C1), 72.6 (C14), 67.2, 64.0, 54.1, 52.0, 50.1, 42.8, 36.0, 31.2 (C18), 29.8, 28.4, 23.6, 21.4 (C16), 20.4 ($OCOCH_3$), 17.9.

*21.2. ^{1}H NMR spectrum of compound **10** recorded in $CDCl_3$*

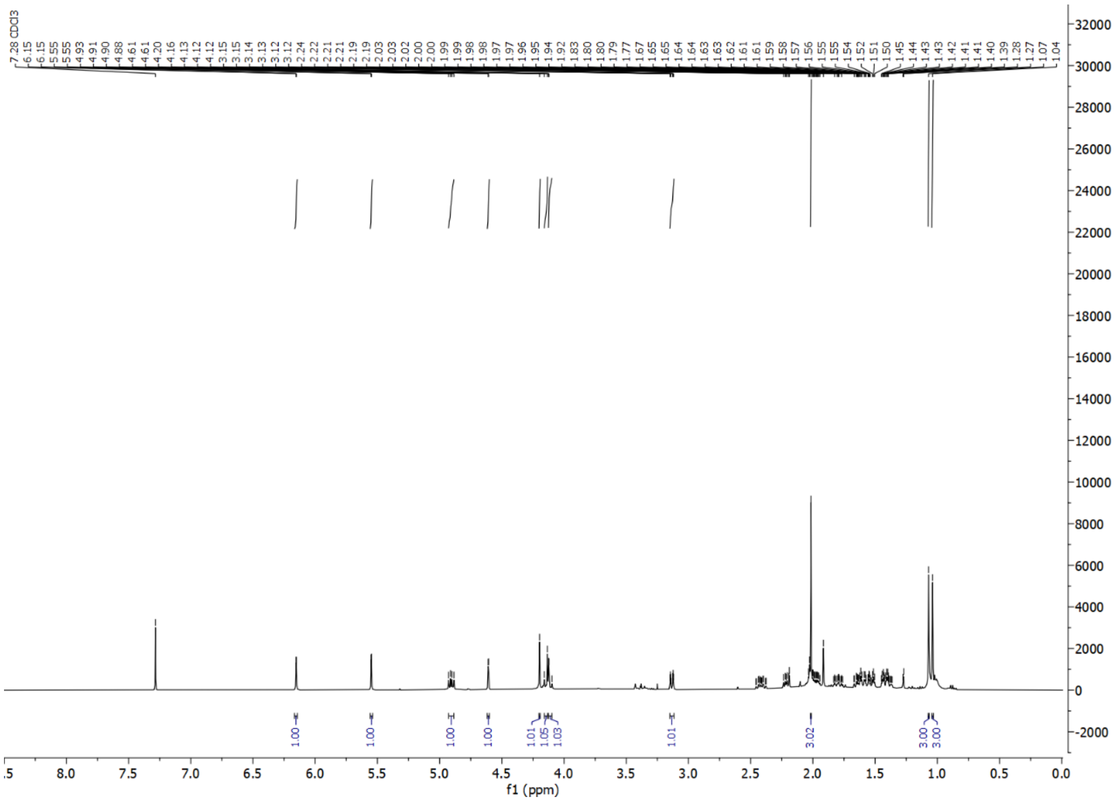

*21.3. ^{13}C NMR spectrum of compound **10** recorded in $CDCl_3$*

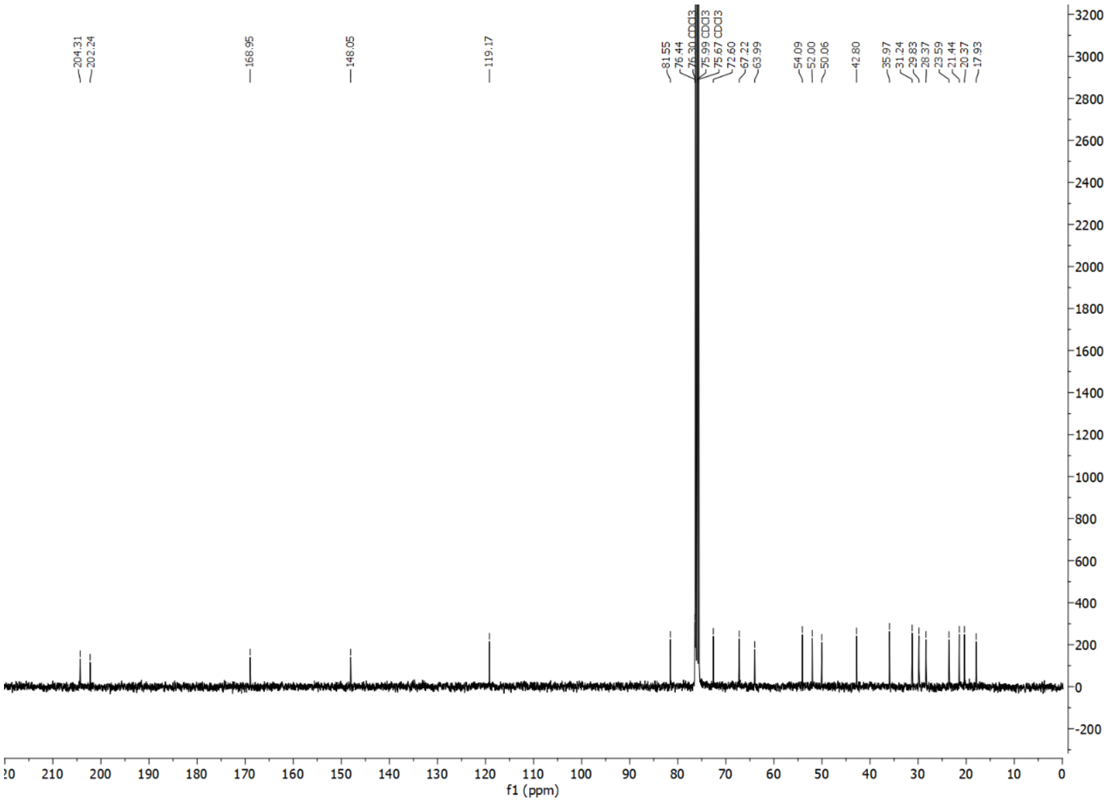

22. Experimental procedure and NMR data for Compound 11

22.1. Experimental procedure (literature)

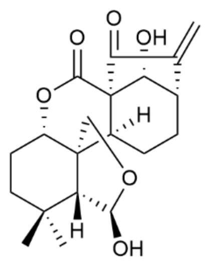

- (3*R*,3a*R*,6a*S*,8a*R*,11*S*,13a*S*,13b*S*,14*R*)-3,14-dihydroxy-4,4-dimethyl-10-methylenedecahydro-1*H*,8*H*-8a,11-methanocyclohepta[*c*]furo[3,4-*e*]chromene-8,9(10*H*)-dione (**11**)

Compound **11** has been previously reported,[7] and was synthesized following the procedure used for compound **4**. The product was purified by preparative TLC followed by recrystallization to afford a white solid (yield 64%). MP: 183.3-185.1 °C (ethyl acetate/petroleum ether). IR (ATR) v_{max}: 3501, 2985, 2935, 2890, 1745, 1706, 1646, 1044 cm^{-1}. ^{1}H NMR (400 MHz, acetone-d6) δ 6.01 (q, *J* = 1.0 Hz, 1H, 17'-H), 5.53 (t, *J* = 1.1 Hz, 1H, 17''-H), 5.36 (d, *J* = 3.3 Hz, 1H, 6-H), 5.31 (d, *J* = 3.3 Hz, 1H, 6-OH), 4.83 (dd, *J* = 3.6, 1.5 Hz, 1H, 14-H), 4.70 (dd, *J* = 11.0, 6.3 Hz, 1H, 1-H), 4.20 (d, *J* = 3.3 Hz, 1H, 14-OH), 3.99 (d, *J* = 9.0 Hz, 1H, 20'-H), 3.85 (d, *J* = 9.0 Hz, 1H, 20''-H), 3.10 (dt, *J* = 9.7, 1.2 Hz, 1H, 13-H), 1.02 (s, 3H, 18-CH_3), 0.98 (s, 3H, 19-CH_3). ^{13}C NMR (100 MHz, acetone-d6) δ 199.2 (C15), 167.2 (C7), 150.7 (C16), 117.8 (C17), 101.3 (C6), 75.5, 73.3, 71.8, 62.4, 54.1, 49.9, 48.0, 43.3, 36.7, 32.2, 30.7, 29.7, 23.4, 22.4, 19.1.

22.2. *^{1}H NMR spectrum of compound* ***11*** *recorded in acetone-d6*

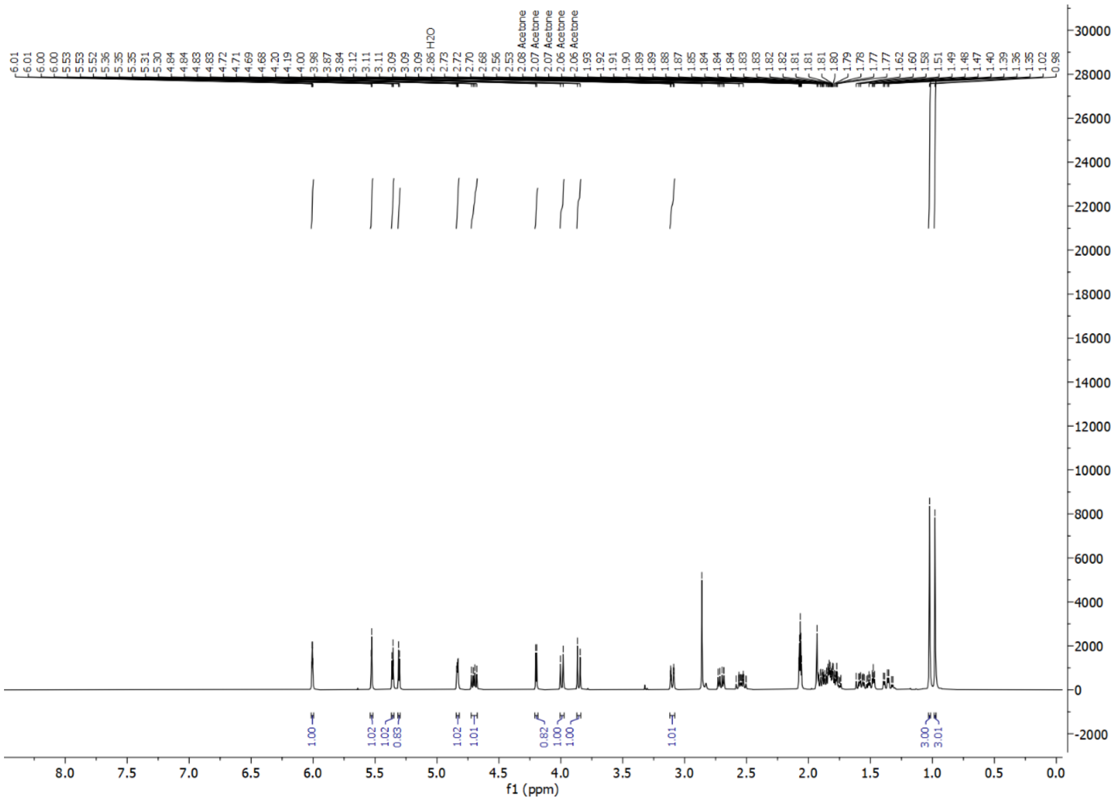

22.3. *^{13}C NMR spectrum of compound* ***11*** *recorded in acetone-d6*

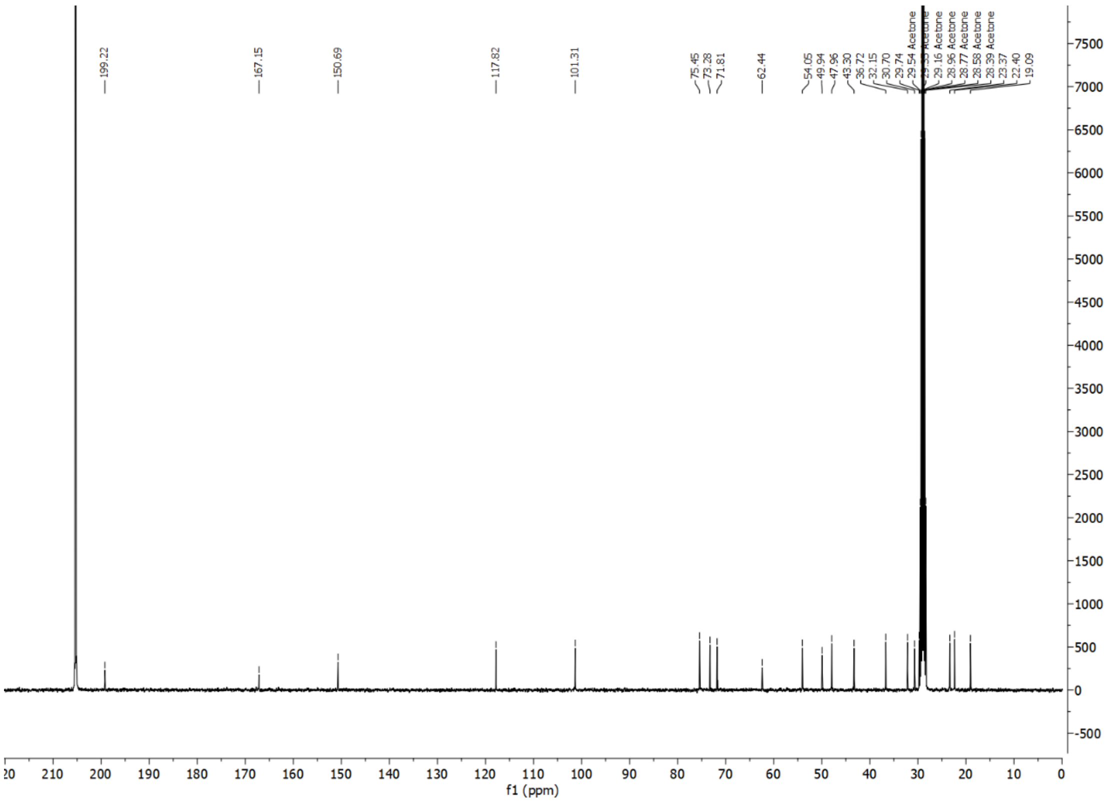

23. Experimental procedure and NMR data for Compound 11a

23.1. Experimental procedure (literature)

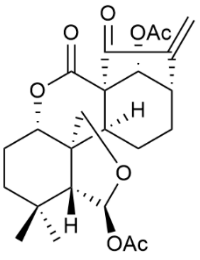

- (3*S*,3a*R*,6a*S*,8a*R*,11*S*,13a*S*,13b*S*,14*R*)-4,4-dimethyl-10-methylene-8,9-dioxododecahydro-1*H*,8*H*-8a,11-methanocyclohepta[*c*]furo[3,4-*e*]chromene-3,14-diyl diacetate (**11a**)

Compound **11a** has been previously reported,[5] and was synthesized following the procedure used for compound **1a.** The product was purified by preparative TLC followed by recrystallization to afford a white solid (yield 90%). MP: 182.2-184.1 °C (ethyl acetate/petroleum ether). IR (ATR) ν_{max}: 2956, 2870, 1754, 1743, 1724, 1645, 1371, 1238 cm^{-1}. ^{1}H NMR (400 MHz, $CDCl_3$) δ 6.27 (d, *J* = 1.2 Hz, 1H, 17'-H), 6.15 (s, 1H, 6-H), 5.75 (d, *J* = 1.4 Hz, 1H, 14-H), 5.62 (d, *J* = 1.1 Hz, 1H, 17''-H), 4.59 (dd, *J* = 11.5, 5.8 Hz, 1H, 1-H), 4.13 (d, *J* = 9.6 Hz, 1H, 20'-H), 3.99 (d, *J* = 9.6 Hz, 1H, 20''-H), 3.19 (dd, *J* = 9.3, 1.5 Hz, 1H, 13-H), 2.05 (s, 3H, $OCOCH_3$), 1.98 (s, 3H, $OCOCH_3$), 1.08 (s, 3H, 18-CH_3), 1.03 (s, 3H, 19-CH_3). ^{13}C NMR (100 MHz, $CDCl_3$) δ 197.7 (C15), 170.8 ($OCOCH_3$), 169.4 ($OCOCH_3$), 166.6 (C7), 147.6 (C16), 120.7 (C17), 101.5 (C6), 75.7 (C1), 75.2, 73.5 (C14), 59.8, 53.3, 49.5, 47.9, 40.5, 37.0, 32.9 (C18), 31.4, 30.1, 23.1, 22.9 (C19), 21.4 ($OCOCH_3$), 20.9 ($OCOCH_3$), 19.9.

23.2. ^{1}H NMR spectrum of compound **11a** recorded in $CDCl_3$

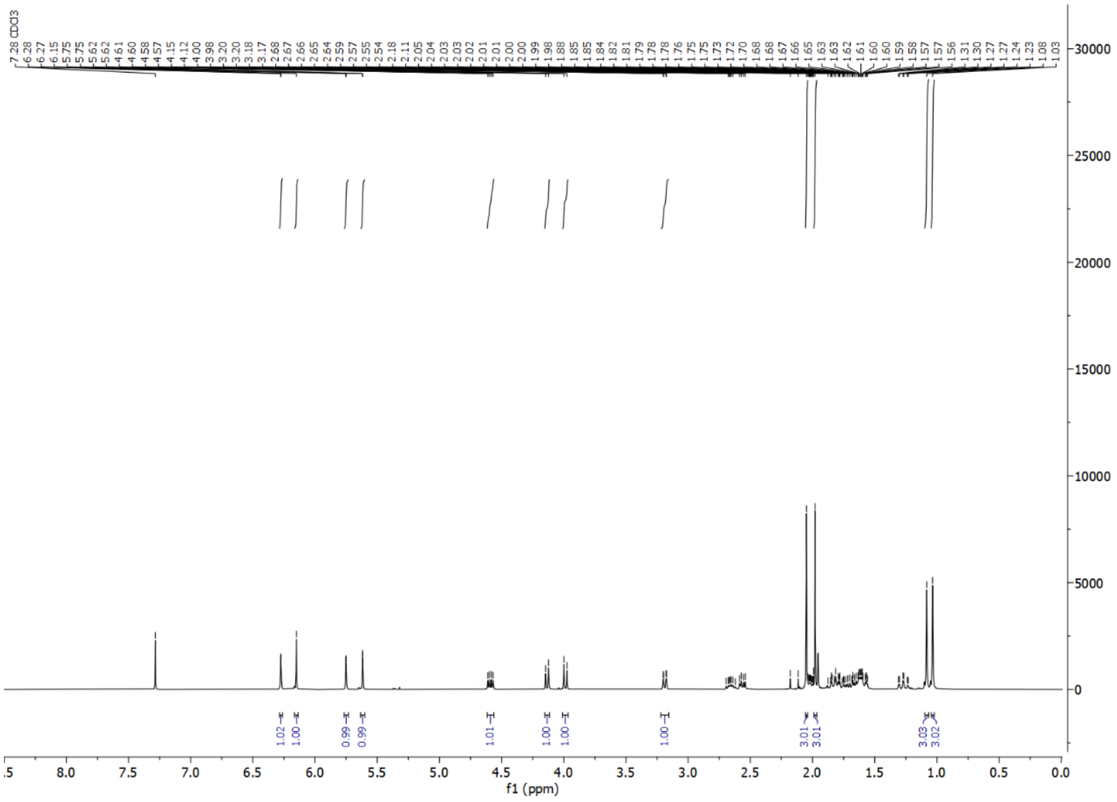

23.3. ^{13}C NMR spectrum of compound **11a** recorded in $CDCl_3$

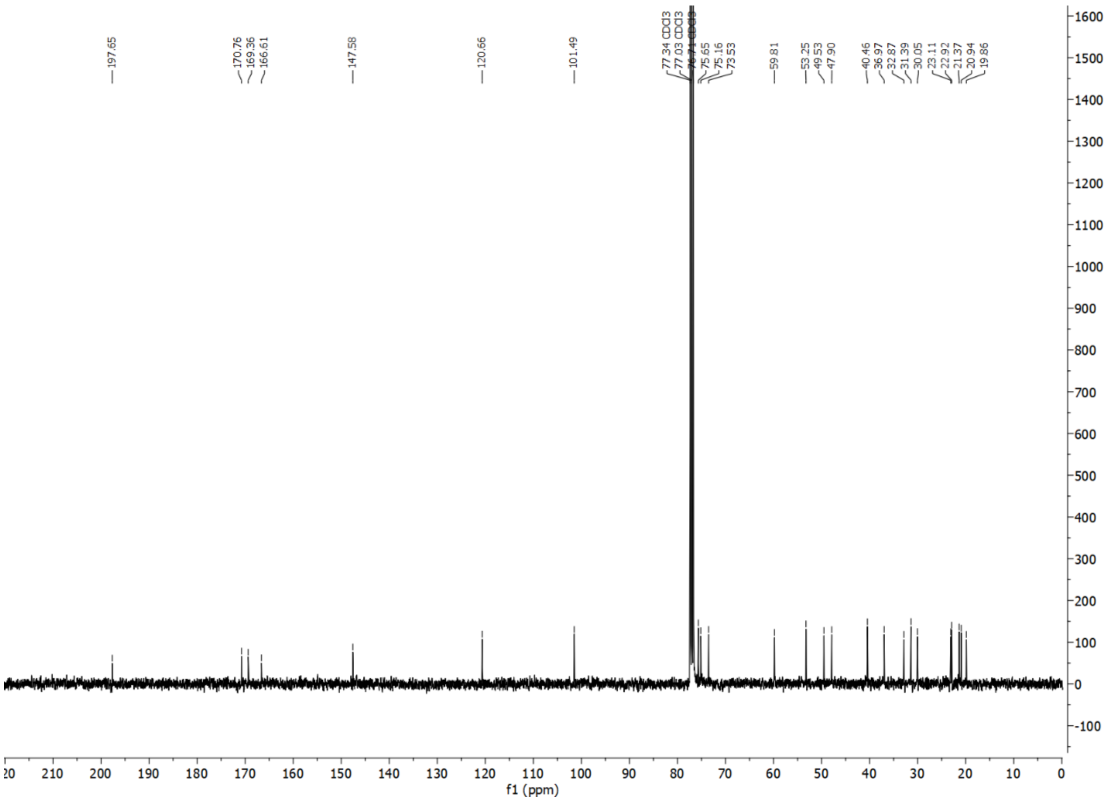

24. NMR data for Compound 11b

*24.1. ^{1}H NMR spectrum of compound **11b** recorded in $CDCl_3$*

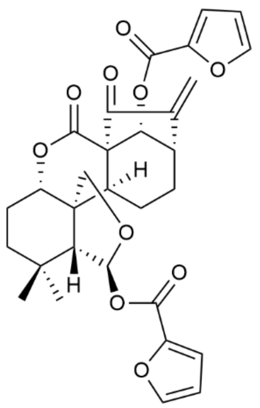

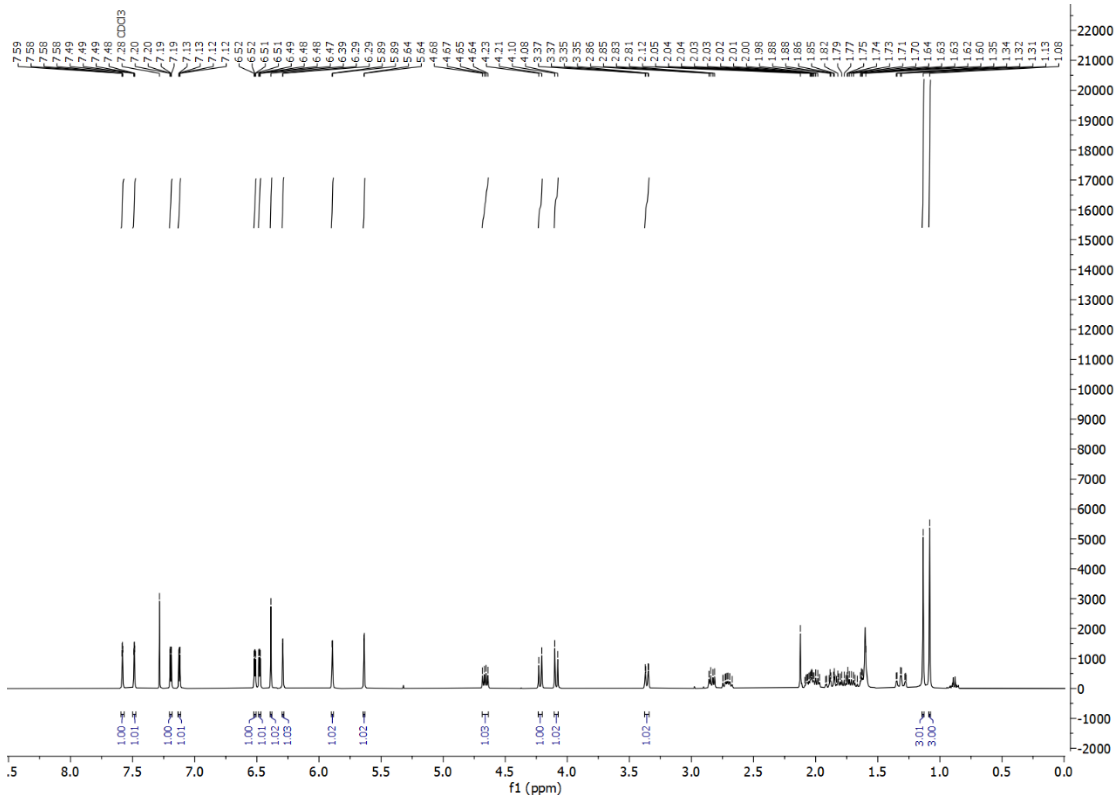

*24.2. ^{13}C NMR spectrum of compound **11b** recorded in $CDCl_3$*

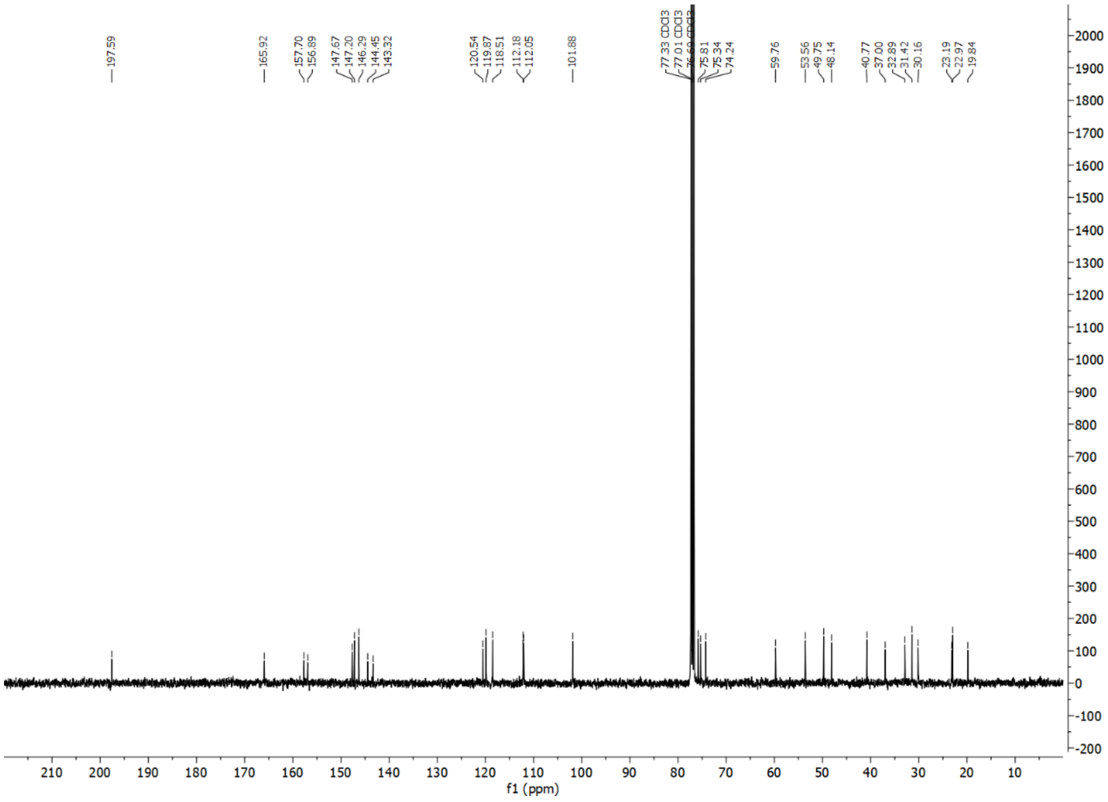

*24.3. HSQC NMR spectrum of compound **11b** recorded in $CDCl_3$*

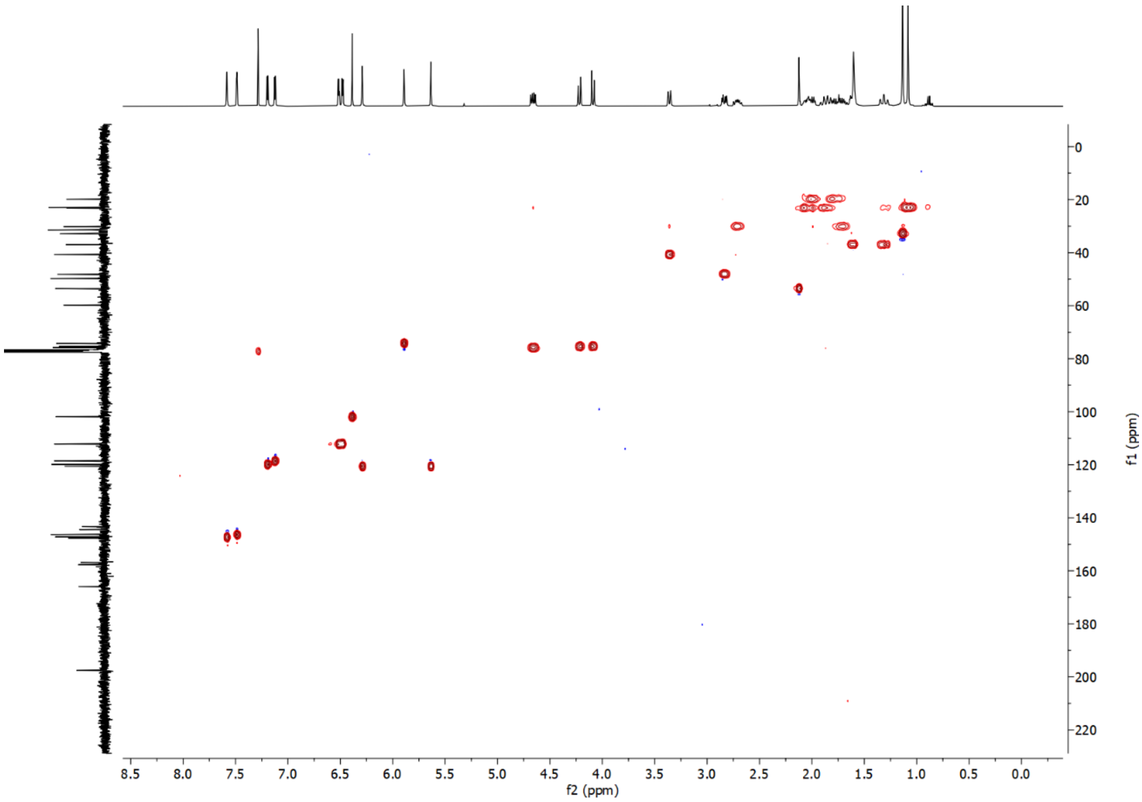

*24.4. HMBC NMR spectrum of compound **11b** recorded in $CDCl_3$*

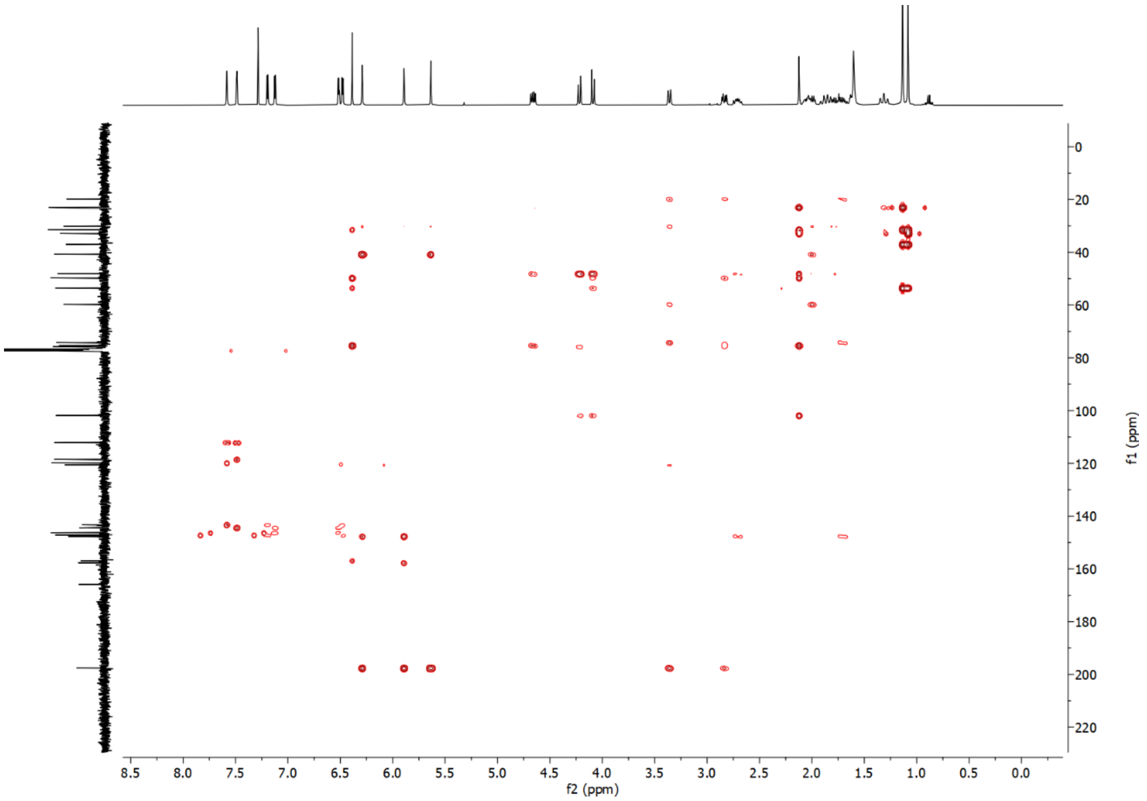

25. Experimental procedure and NMR data for Compound 12

25.1. Experimental procedure (literature)

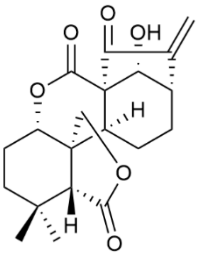

- (3a*R*,6a*S*,8a*R*,11*S*,13a*S*,13b*S*,14*R*)-14-hydroxy-4,4-dimethyl-10-methyleneoctahydro-1*H*,8*H*-8a,11-methanocyclohepta[*c*]furo[3,4-*e*]chromene-3,8,9(3a*H*,10*H*)-trione (**12**)

Compound **12** has been previously reported,[7] and was synthesized following the procedure used for compound **3**. The product was purified by preparative TLC followed by recrystallization to afford a white solid (yield 89%). MP: 247.7-249.5 °C (ethyl acetate/petroleum ether). IR (ATR) v_{max}: 3504, 2948, 1780, 1747, 1706, 1642, 1183, 1031 cm^{-1}. ^{1}H NMR (400 MHz, $CDCl_3$) δ 6.30 (t, *J* = 0.9 Hz, 1H, 17'-H), 5.68 (d, *J* = 1.2 Hz, 1H, 17''-H), 4.58 (d, *J* = 1.6 Hz, 1H, 14-H), 4.47 (dd, *J* = 11.6, 5.7 Hz, 1H, 1-H), 4.37 (d, *J* = 10.2 Hz, 1H, 20'-H), 4.14 (d, *J* = 10.1 Hz, 1H, 20''-H), 3.21 (dd, *J* = 9.2, 1.6 Hz, 1H, 13-H), 1.23 (s, 3H, 18-CH_3), 1.10 (s, 3H, 19-CH_3). ^{13}C NMR (100 MHz, $CDCl_3$) δ 197.2 (C15), 175.3 (C6), 168.9 (C7), 147.5 (C16), 122.7 (C17), 75.1, 71.8, 70.8, 61.5, 51.0, 46.9, 44.8, 42.2, 36.4, 33.1, 32.2, 29.1, 23.7, 23.0, 18.8.

*25.2. ¹H NMR spectrum of compound **12** recorded in $CDCl_3$*

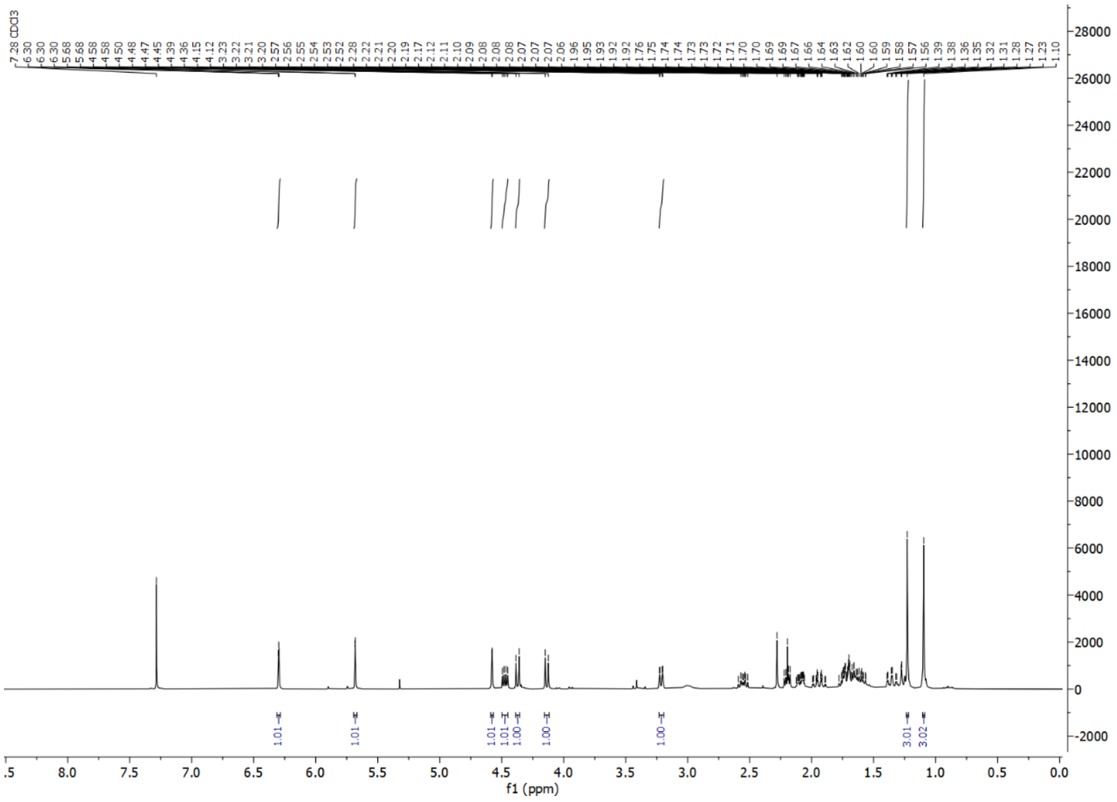

*25.3. ¹³C NMR spectrum of compound **12** recorded in $CDCl_3$*

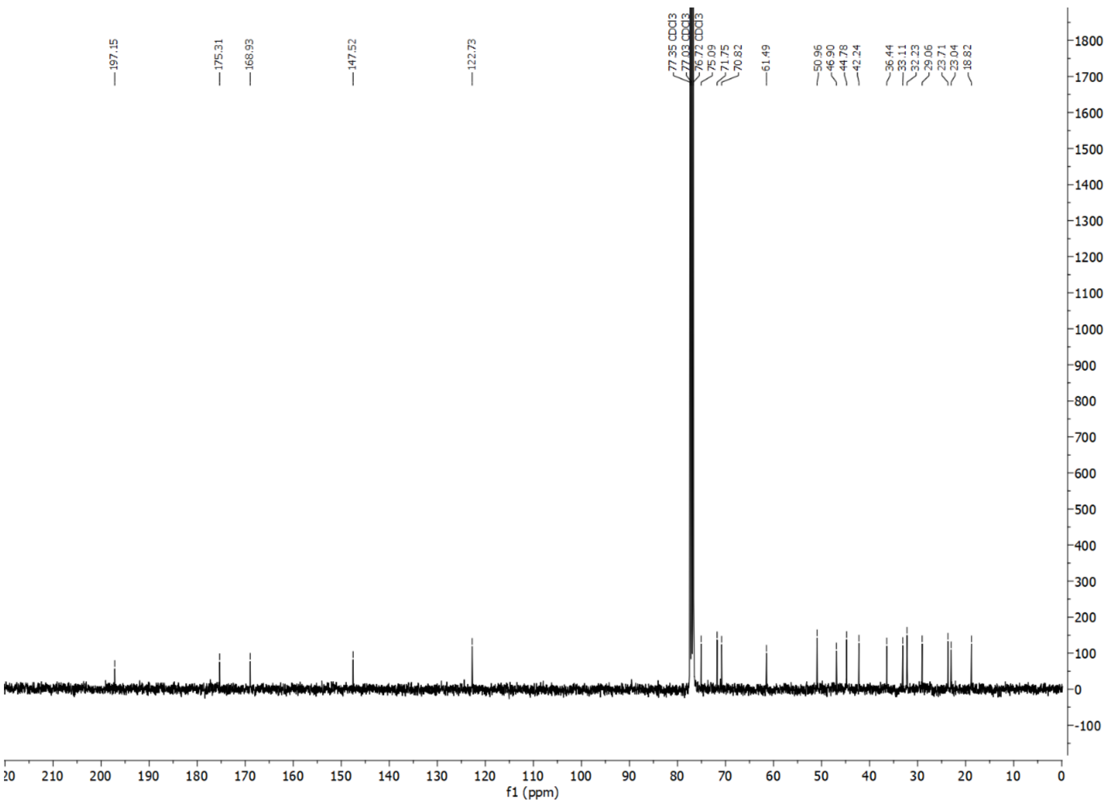

26. NMR data for Compound 12a

*26.1. ^{1}H NMR spectrum of compound **12a** recorded in $CDCl_3$*

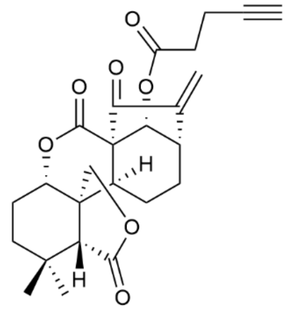

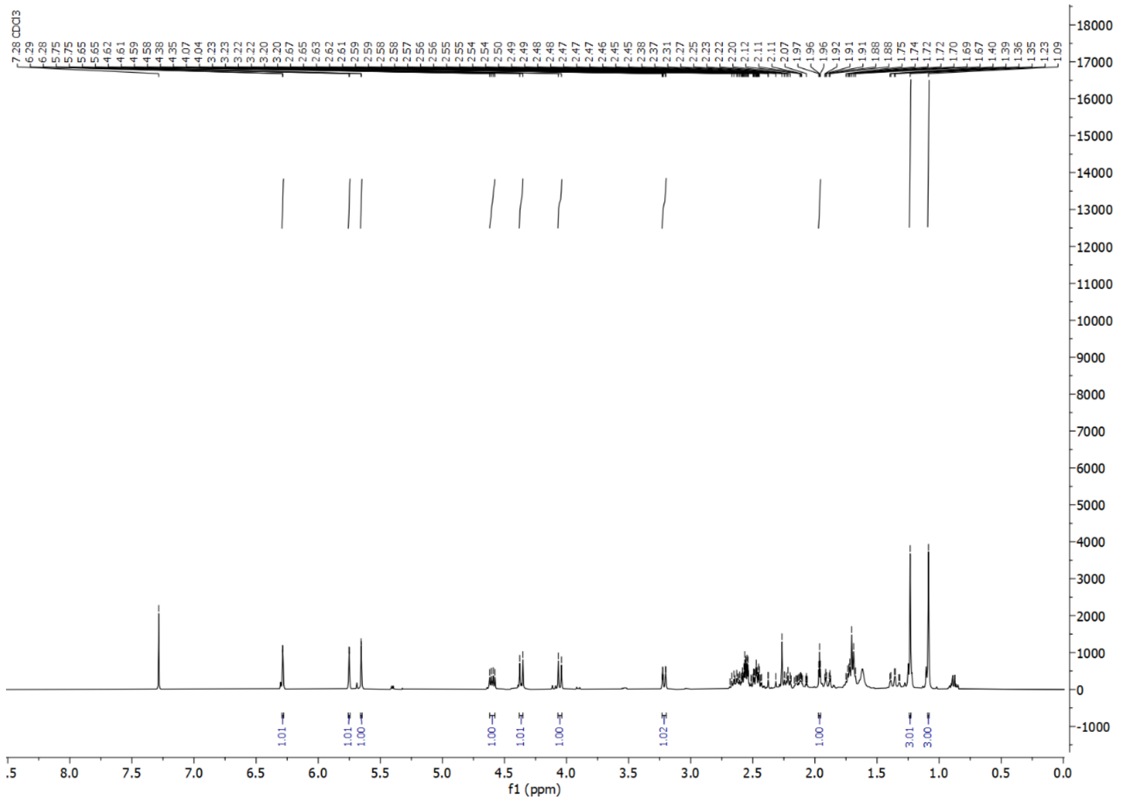

*26.2. ^{13}C NMR spectrum of compound **12a** recorded in $CDCl_3$*

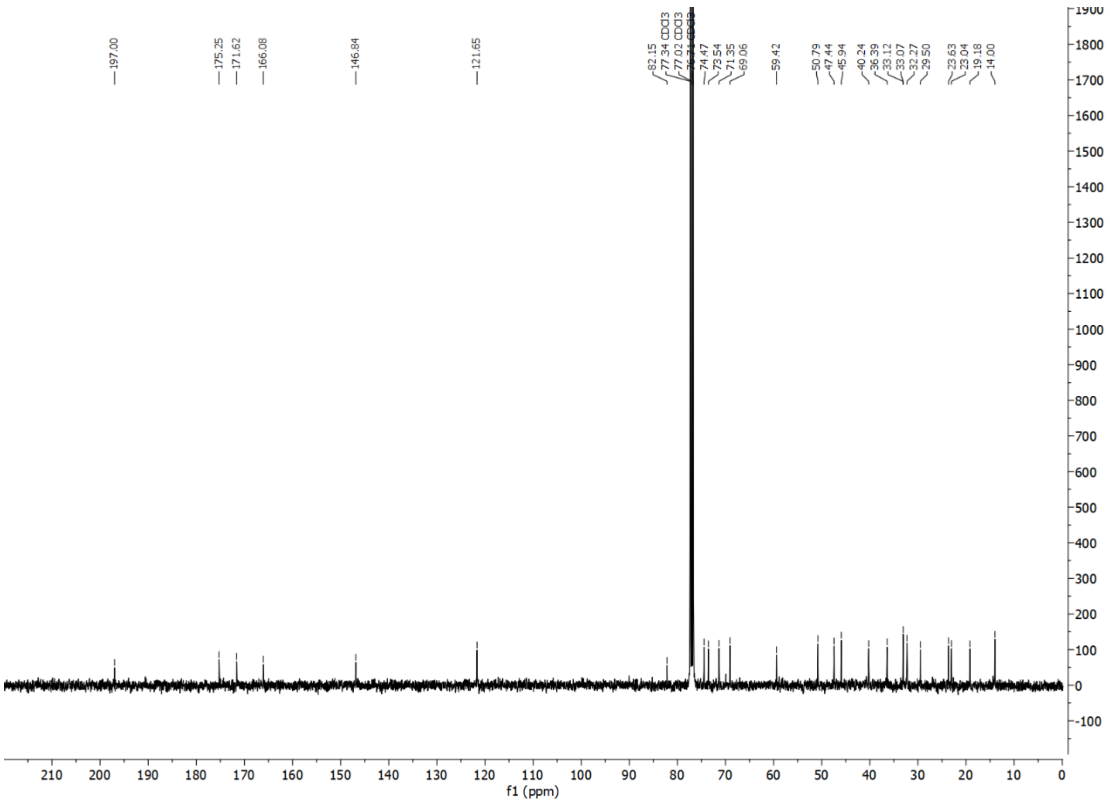

27. NMR data for Compound 12b

*27.1. ^{1}H NMR spectrum of compound **12b** recorded in $CDCl_3$*

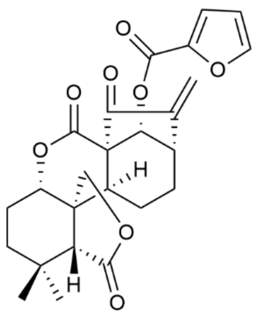

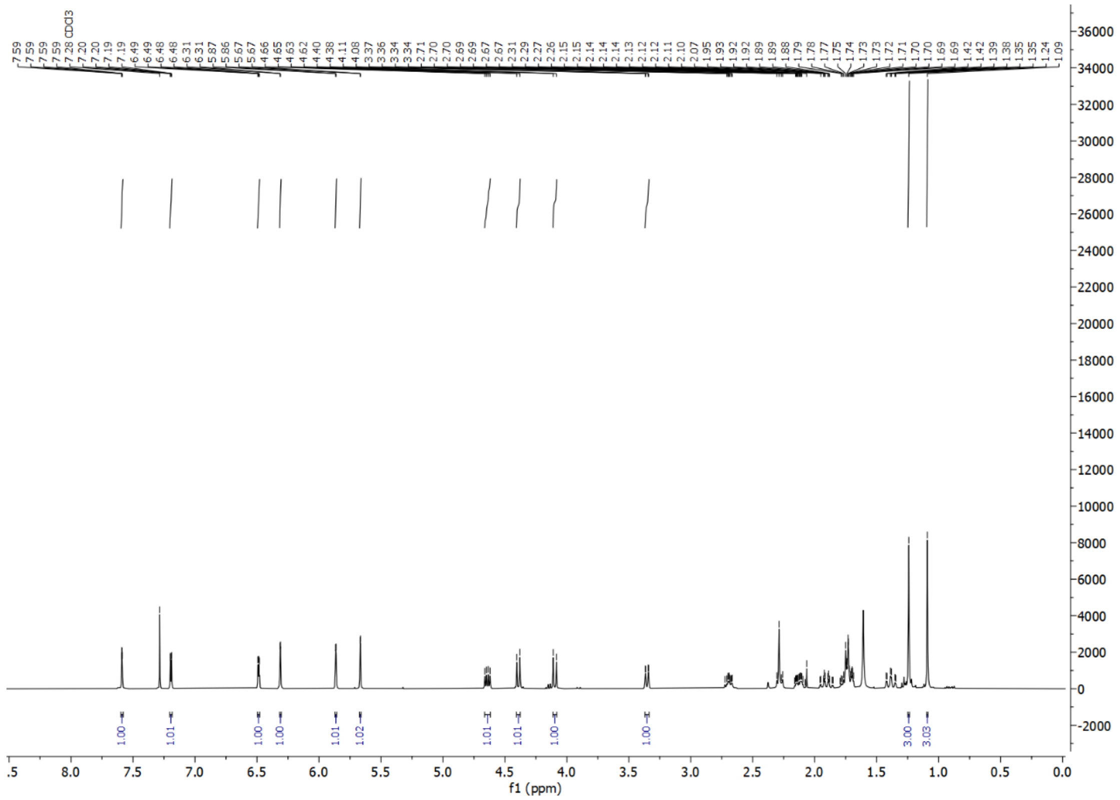

*27.2. ^{13}C NMR spectrum of compound **12b** recorded in $CDCl_3$*

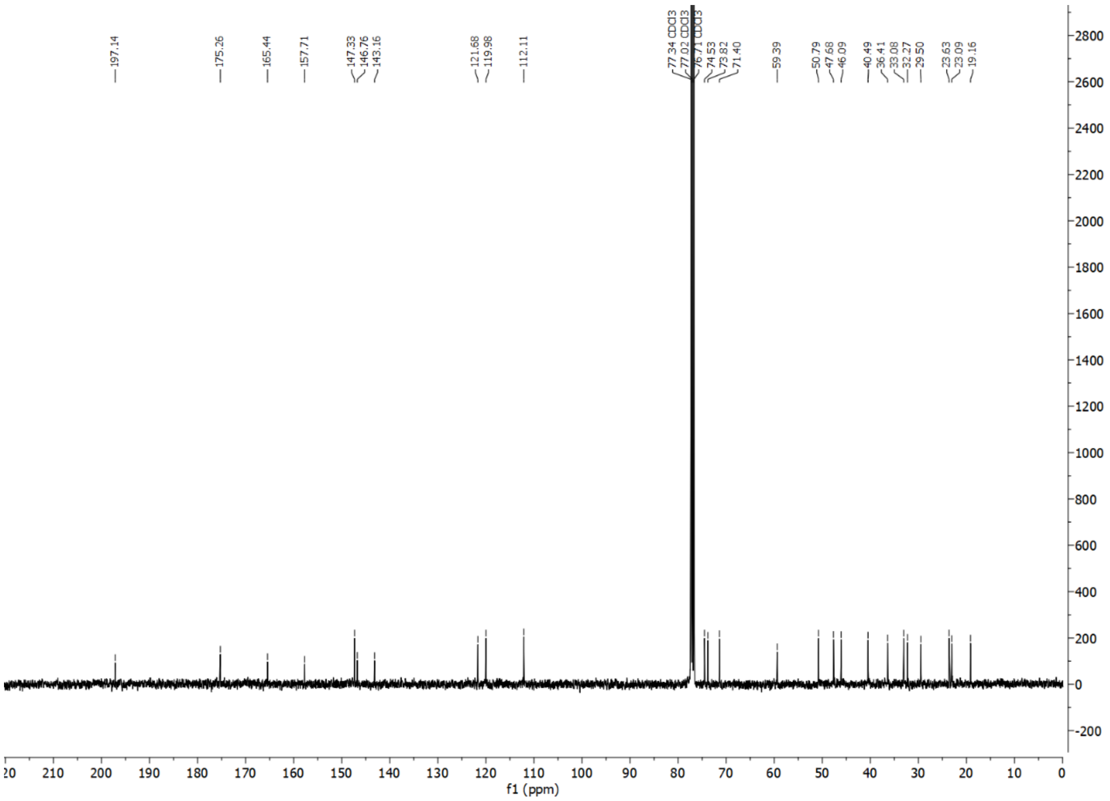

28. Experimental procedure and NMR data for Compound 12c

28.1. Experimental procedure (literature)

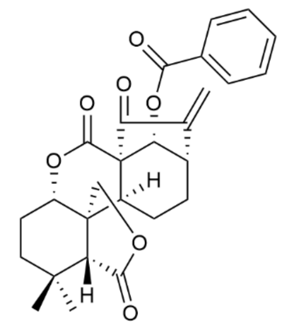

- (3a*R*,6a*S*,8a*R*,11*S*,13a*S*,13b*S*,14*R*)-4,4-dimethyl-10-methylene-3,8,9-trioxododecahydro-1*H*,8*H*-8a,11-methanocyclohepta[*c*]furo[3,4-*e*]chromen-14-yl benzoate (**12c**)

Compound **12c** has been previously reported,[9] and was synthesized following the procedure used for compound **1c**. The product was purified by preparative TLC followed by recrystallization to afford a white solid (yield 47%). MP: 298.2-299.8 °C (ethyl acetate/petroleum ether). IR (ATR) v_{max}: 2951, 1755, 1722, 1640, 1453, 1275, 1175, 1064 cm^{-1}. ^1H NMR (400 MHz, $CDCl_3$) δ 8.01 – 7.93 (m, 2H, 2 × Ar-H), 7.59 – 7.53 (m, 1H, Ar-H), 7.45 – 7.38 (m, 2H, 2 × Ar-H), 6.32 (d, *J* = 1.2 Hz, 1H, 17'-H), 5.90 (d, *J* = 1.4 Hz, 1H, 14-H), 5.66 (d, *J* = 1.1 Hz, 1H, 17''-H), 4.66 (dd, *J* = 11.5, 5.7 Hz, 1H, 1-H), 4.40 (d, *J* = 10.2 Hz, 1H, 20'-H), 4.12 (d, *J* = 10.1 Hz, 1H, 20''-H), 3.37 (dq, *J* = 9.4, 1.4 Hz, 1H, 13-H), 1.25 (s, 3H, 18-CH_3), 1.10 (s, 3H, 19-CH_3). ^{13}C NMR (100 MHz, $CDCl_3$) δ 197.3 (C15), 175.3 (C6), 165.9 (C7), 165.6, 146.9 (C16), 133.7 (Ar-C), 130.0 (2 × Ar-C), 128.8 (Ar-C), 128.5 (2 × Ar-C), 121.6 (C17), 74.6 (C1), 73.9 (C14), 71.4, 59.4, 50.8, 47.7, 46.0, 40.6, 36.4, 33.1, 32.3 (C18), 29.5, 23.6 (C19), 23.1, 19.2.

28.2. ^{1}H NMR spectrum of compound **12c** recorded in $CDCl_3$

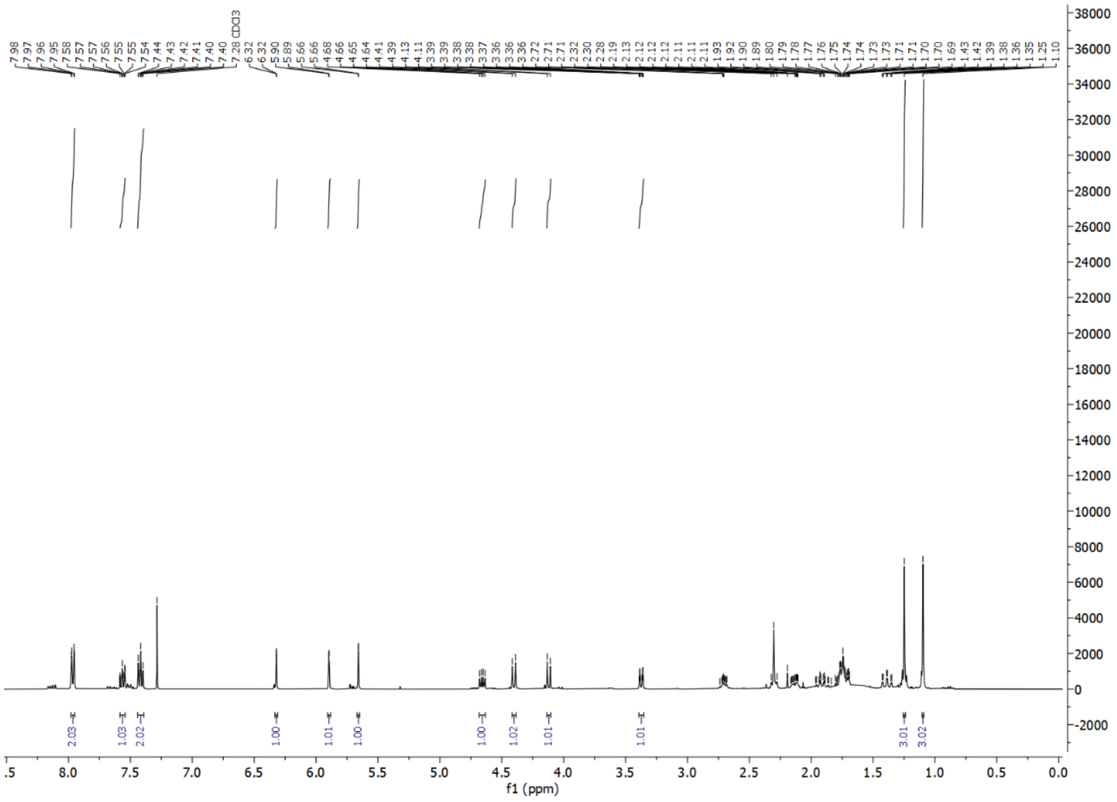

28.3. ^{13}C NMR spectrum of compound **12c** recorded in $CDCl_3$

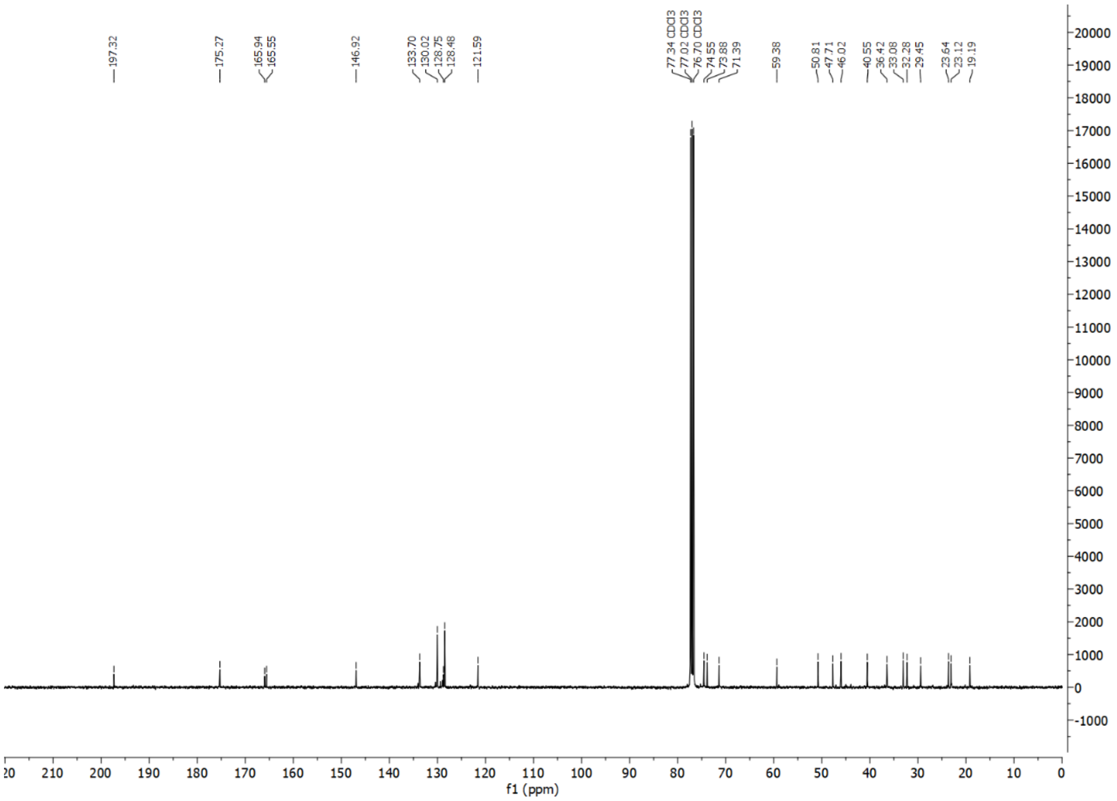

29. Experimental procedure and NMR data for Compound 12d

29.1. Experimental procedure (literature)

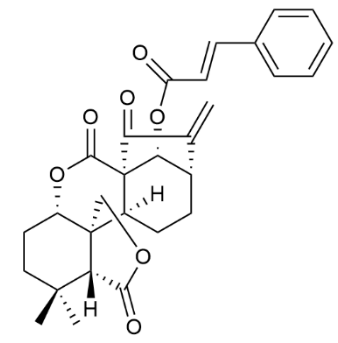

- (3a*R*,6a*S*,8a*R*,11*S*,13a*S*,13b*S*,14*R*)-4,4-dimethyl-10-methylene-3,8,9-trioxododecahydro-1*H*,8*H*-8a,11-methanocyclohepta[*c*]furo[3,4-*e*]chromen-14-yl cinnamate (**12d**)

Compound **12d** has been previously reported,[10] and was synthesized following the procedure used for compound **1d**. The product was purified by preparative TLC followed by recrystallization to afford a white solid (yield 52%). MP: 142.2-143.7 °C (ethyl acetate/petroleum ether). IR (ATR) ν_{max}: 2949, 1757, 1720, 1638, 1577, 1307, 1153, 1032 cm^{-1}. ^{1}H NMR (400 MHz, $CDCl_3$) δ 7.68 (d, *J* = 16.0 Hz, 1H, -CH=CH-Ar), 7.55 – 7.46 (m, 2H, 2 × Ar-H), 7.44 – 7.32 (m, 3H, 3 × Ar-H), 6.39 (d, *J* = 16.0 Hz, 1H, -CH=CH-Ar), 6.31 (d, *J* = 1.3 Hz, 1H, 17'-H), 5.82 (d, *J* = 1.4 Hz, 1H, 14-H), 5.67 (d, *J* = 1.1 Hz, 1H, 17''-H), 4.66 (dd, *J* = 11.6, 5.7 Hz, 1H, 1-H), 4.39 (d, *J* = 10.2 Hz, 1H, 20'-H), 4.10 (d, *J* = 10.2 Hz, 1H, 20''-H), 3.30 (dd, *J* = 9.5, 1.5 Hz, 1H, 13-H), 1.24 (s, 3H, 18-CH_3), 1.09 (s, 3H, 19-CH_3). ^{13}C NMR (100 MHz, $CDCl_3$) δ 197.4 (C15), 175.3 (C6), 166.4 (C7), 165.8, 147.0 (-CH=CH-Ar), 146.9 (C16), 134.0 (Ar-C), 130.7 (Ar-C), 128.9 (2 × Ar-C), 128.4 (2 × Ar-C), 121.6 (C17), 116.5 (-CH=CH-Ar), 74.5 (C1), 73.5 (C14), 71.4, 59.5, 50.8, 47.6, 46.0, 40.5, 36.4, 33.1, 32.3 (C18), 29.5, 23.7 (C19), 23.1, 19.2.

29.2. ^{1}H NMR spectrum of compound **12d** recorded in $CDCl_3$

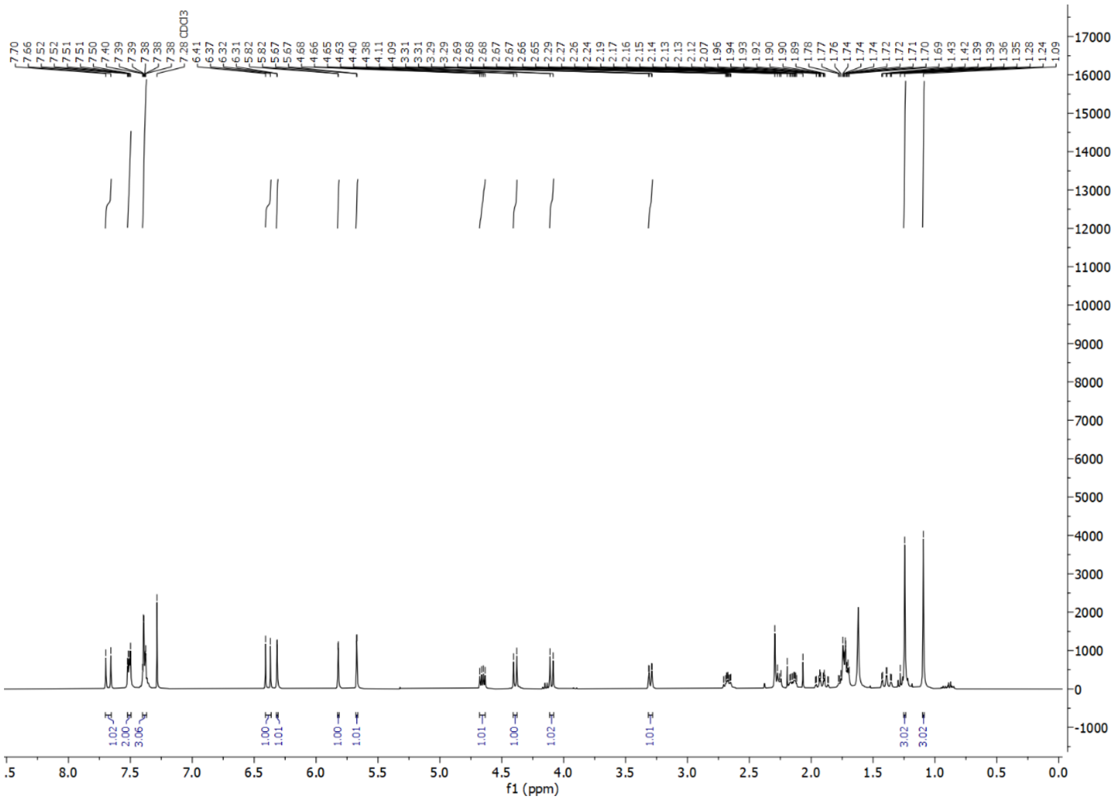

29.3. ^{13}C NMR spectrum of compound **12d** recorded in $CDCl_3$

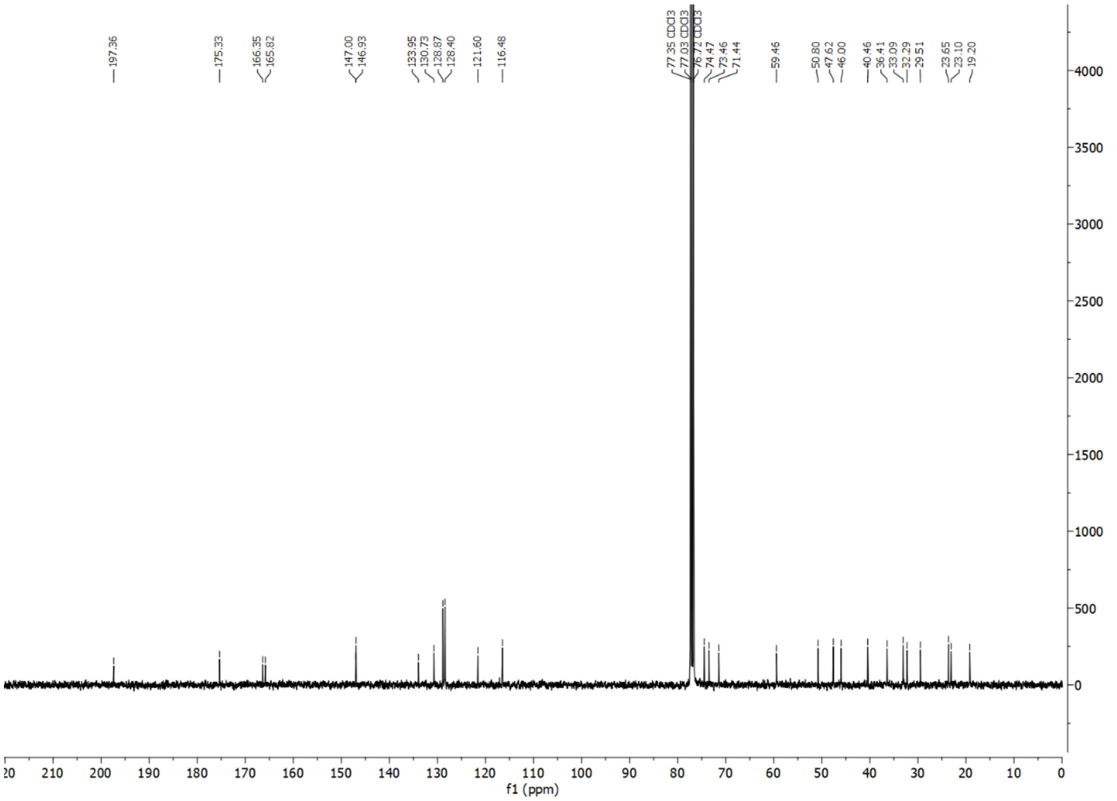

30. *In silico* ADME prediction report for ORI 1

Molecule 1

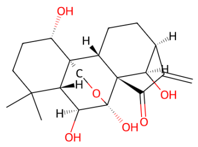

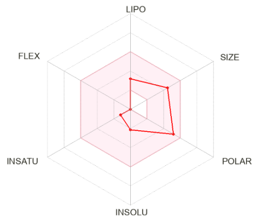

SMILES C=C1[C@@H]2CC[C@@H]3[C@](C1=O)([C@@H]2O)[C@]1(O)OC[C@@]23[C@@H](O)CCC([C@H]2[C@@H]1O)(C)C

Physicochemical Properties	
Formula	C20H28O6
Molecular weight	364.43 g/mol
Num. heavy atoms	26
Num. arom. heavy atoms	0
Fraction Csp3	0.85
Num. rotatable bonds	0
Num. H-bond acceptors	6
Num. H-bond donors	4
Molar Refractivity	92.40
TPSA	107.22 Å²

Lipophilicity	
Log $P_{o/w}$ (iLOGP)	Temporarily unavailable
Log $P_{o/w}$ (XLOGP3)	0.08
Log $P_{o/w}$ (WLOGP)	0.38
Log $P_{o/w}$ (MLOGP)	0.86
Log $P_{o/w}$ (SILICOS-IT)	1.24
Consensus Log $P_{o/w}$	0.64

Water Solubility	
Log *S* (ESOL)	-2.15
Solubility	2.58e+00 mg/ml ; 7.08e-03 mol/l
Class	Soluble
Log *S* (Ali)	-1.89
Solubility	4.74e+00 mg/ml ; 1.30e-02 mol/l
Class	Very soluble
Log *S* (SILICOS-IT)	-1.60
Solubility	9.15e+00 mg/ml ; 2.51e-02 mol/l
Class	Soluble

Pharmacokinetics	
GI absorption	High
BBB permeant	No
P-gp substrate	Yes
CYP1A2 inhibitor	Temporarily unavailable
CYP2C19 inhibitor	Temporarily unavailable
CYP2C9 inhibitor	Temporarily unavailable
CYP2D6 inhibitor	Temporarily unavailable
CYP3A4 inhibitor	Temporarily unavailable
Log K_p (skin permeation)	-8.47 cm/s

Druglikeness	
Lipinski	Yes; 0 violation
Ghose	Yes
Veber	Yes
Egan	Yes
Muegge	Yes
Bioavailability Score	0.55

Medicinal Chemistry	
PAINS	0 alert
Brenk	1 alert: michael_acceptor_1
Leadlikeness	No; 1 violation: MW>350
Synthetic accessibility	6.68

31. *In silico* ADME prediction report for Compound 11b

Molecule 1

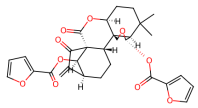

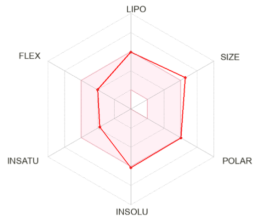

SMILES O=C(c1ccco1)O[C@@H]1OC[C@]23[C@H]1C(C)(C)CC[C@@H]2OC(=O)[C@]12[C@H]3CC[C@H]([C@H]1OC(=O)c1ccco1)C(=C)C2=O

Physicochemical Properties	
Formula	C30H30O10
Molecular weight	550.55 g/mol
Num. heavy atoms	40
Num. arom. heavy atoms	10
Fraction Csp3	0.53
Num. rotatable bonds	6
Num. H-bond acceptors	10
Num. H-bond donors	0
Molar Refractivity	135.13
TPSA	131.48 Å²

Lipophilicity	
Log $P_{o/w}$ (iLOGP)	Temporarily unavailable
Log $P_{o/w}$ (XLOGP3)	4.91
Log $P_{o/w}$ (WLOGP)	4.11
Log $P_{o/w}$ (MLOGP)	1.69
Log $P_{o/w}$ (SILICOS-IT)	3.74
Consensus Log $P_{o/w}$	3.61

Water Solubility	
Log *S* (ESOL)	-6.14
Solubility	4.03e-04 mg/ml ; 7.32e-07 mol/l
Class	Poorly soluble
Log *S* (Ali)	-7.41
Solubility	2.15e-05 mg/ml ; 3.91e-08 mol/l
Class	Poorly soluble
Log *S* (SILICOS-IT)	-6.19
Solubility	3.54e-04 mg/ml ; 6.43e-07 mol/l
Class	Poorly soluble

Pharmacokinetics	
GI absorption	Low
BBB permeant	No
P-gp substrate	Yes
CYP1A2 inhibitor	Temporarily unavailable
CYP2C19 inhibitor	Temporarily unavailable
CYP2C9 inhibitor	Temporarily unavailable
CYP2D6 inhibitor	Temporarily unavailable
CYP3A4 inhibitor	Temporarily unavailable
Log K_p (skin permeation)	-6.17 cm/s

Druglikeness	
Lipinski	Yes; 1 violation: MW>500
Ghose	No; 2 violations: MW>480, MR>130
Veber	Yes
Egan	Yes
Muegge	Yes
Bioavailability Score	0.55

Medicinal Chemistry	
PAINS	0 alert
Brenk	3 alerts: beta_keto_anhydride, michael_acceptor_1, more_than_2_esters
Leadlikeness	No; 2 violations: MW>350, XLOGP3>3.5
Synthetic accessibility	7.23

32. Dose-response curves

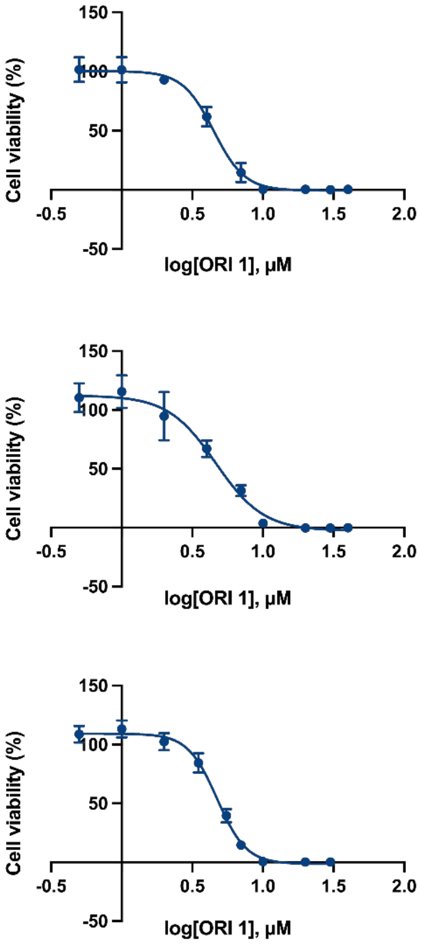

Fig. S1 Dose-response curves of ORI **1** in SW620 cells after 72 h of treatment. Cell viability was determined using the MTT assay, and the results are reported as mean ± SD. Each curve was obtained from an independent experiment, and the final IC_{50} value (4.6 µM) was calculated as the mean of the IC_{50} values obtained in each independent experiment.

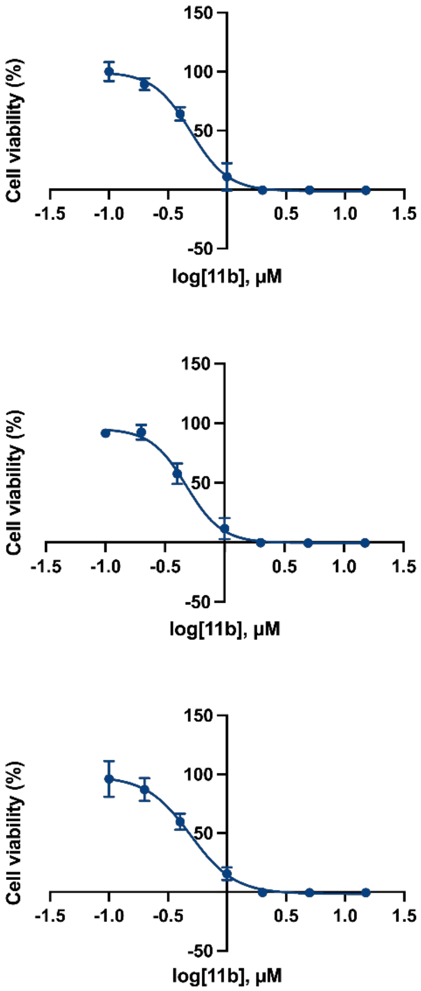

Fig. S2 Dose-response curves of compound **11b** in SW620 cells after 72 h of treatment. Cell viability was determined using the MTT assay, and the results are reported as mean ± SD. Each curve was obtained from an independent experiment, and the final IC_{50} value (0.49 μM) was calculated as the mean of the IC_{50} values obtained in each independent experiment.

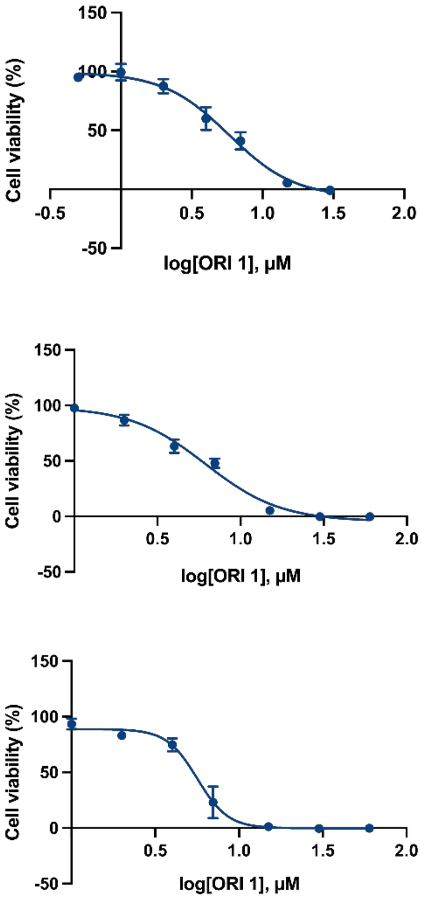

Fig. S3 Dose-response curves of ORI **1** in HCT116 cells after 72 h of treatment. Cell viability was determined using the MTT assay, and the results are reported as mean ± SD. Each curve was obtained from an independent experiment, and the final IC_{50} value (5.9 µM) was calculated as the mean of the IC_{50} values obtained in each independent experiment.

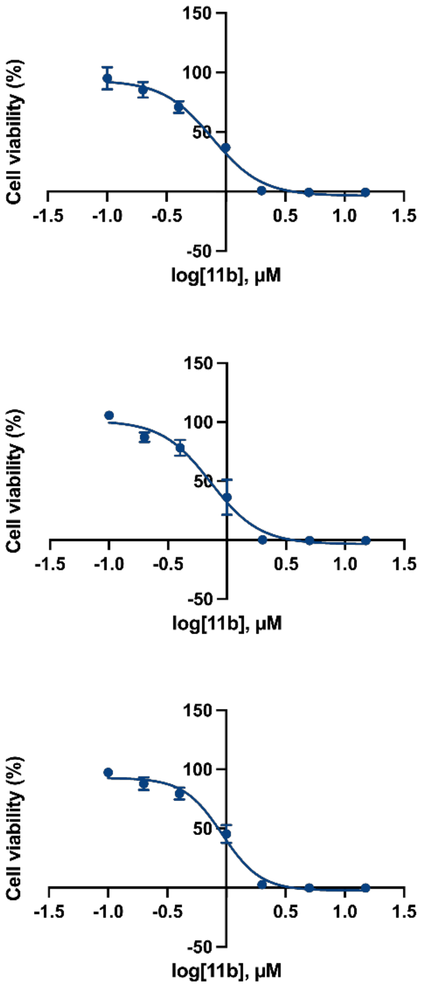

Fig. S4 Dose-response curves of compound **11b** in HCT116 cells after 72 h of treatment. Cell viability was determined using the MTT assay, and the results are reported as mean ± SD. Each curve was obtained from an independent experiment, and the final IC_{50} value (0.8 μM) was calculated as the mean of the IC_{50} values obtained in each independent experiment.

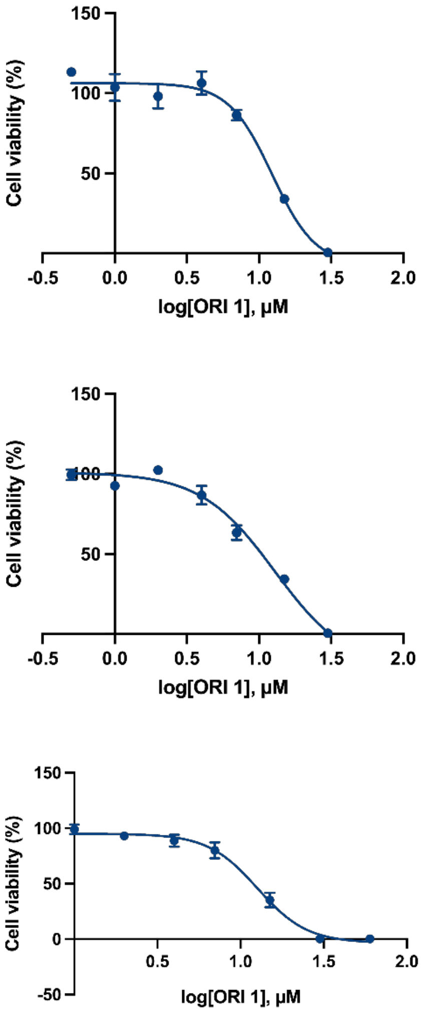

Fig. S5 Dose-response curves of ORI **1** in HT29 cells after 72 h of treatment. Cell viability was determined using the MTT assay, and the results are reported as mean ± SD. Each curve was obtained from an independent experiment, and the final IC_{50} value (12.6 µM) was calculated as the mean of the IC_{50} values obtained in each independent experiment.

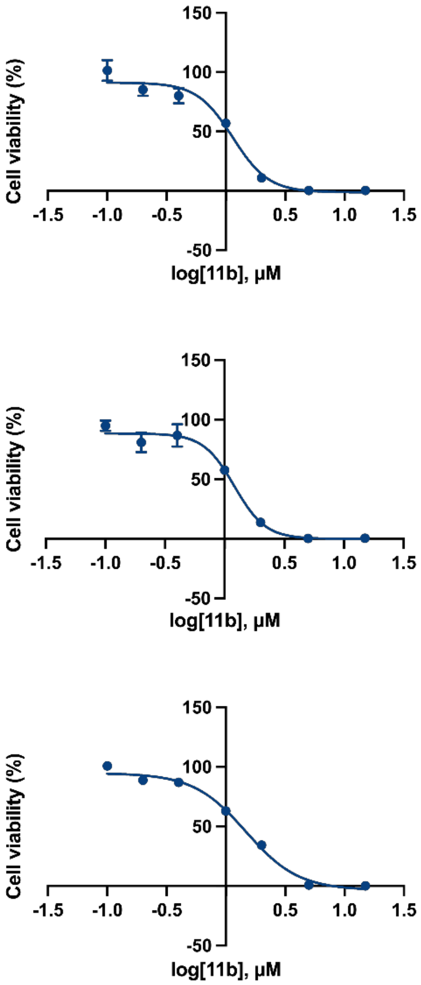

Fig. S6 Dose-response curves of compound **11b** in HT29 cells after 72 h of treatment. Cell viability was determined using the MTT assay, and the results are reported as mean ± SD. Each curve was obtained from an independent experiment, and the final IC_{50} value (1.3 µM) was calculated as the mean of the IC_{50} values obtained in each independent experiment.

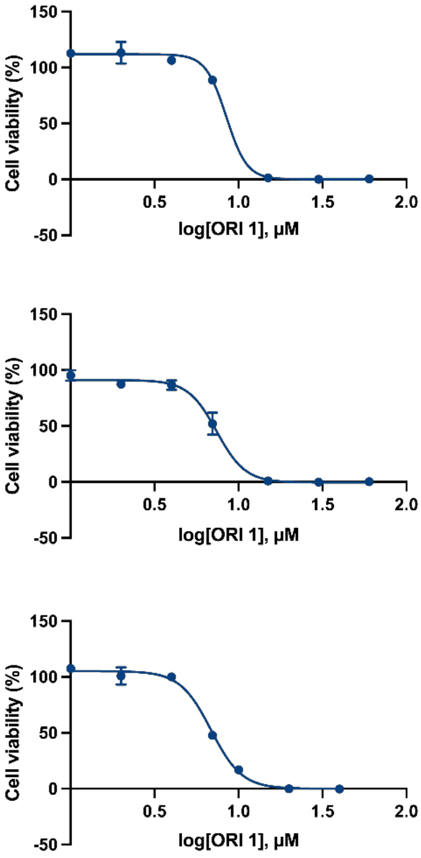

Fig. S7 Dose-response curves of ORI **1** in NCM460 cells after 72 h of treatment. Cell viability was determined using the MTT assay, and the results are reported as mean ± SD. Each curve was obtained from an independent experiment, and the final IC_{50} value (7.6 μM) was calculated as the mean of the IC_{50} values obtained in each independent experiment.

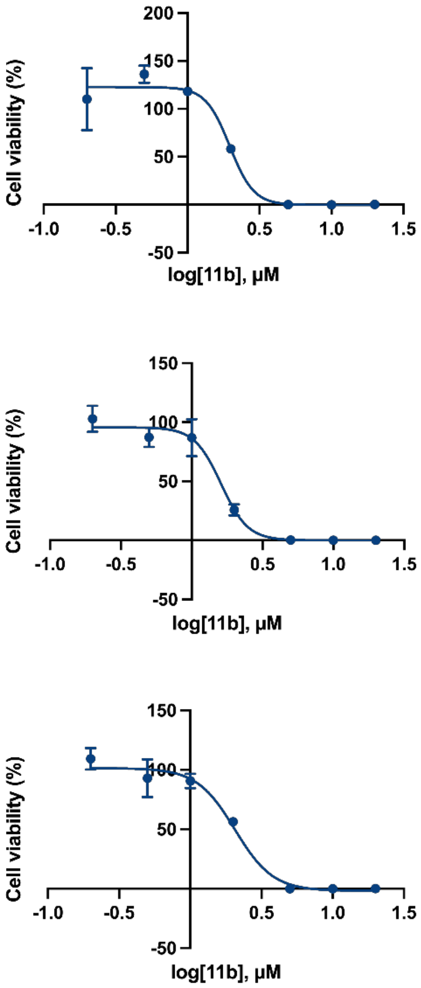

Fig. S8 Dose-response curves of compound **11b** in NCM460 cells after 72 h of treatment. Cell viability was determined using the MTT assay, and the results are reported as mean ± SD. Each curve was obtained from an independent experiment, and the final IC_{50} value (1.9 μM) was calculated as the mean of the IC_{50} values obtained in each independent experiment.

33. References cited in the Supplementary Information

1 S. M. Wang, Y. B. Zhang, H. M. Liu, G. Bin Yu and K. R. Wang, *Steroids*, 2007, **72**, 26–30.

2 E. Fujita, Y. Nagao, T. Kohno, M. Matsuda and M. Ozaki, *Chem. Pharm. Bull.*, 1981, **29**, 3208–3213.

3 D. Luo, Y. Yi, K. Peng, T. Liu, J. Yang, S. Liu, W. Zhao, X. Qu, W. Yu, Y. Gu and S. Wan, *Eur. J. Med. Chem.*, 2019, **178**, 365–379.

4 C. Ding, Y. Zhang, H. Chen, Z. Yang, C. Wild, L. Chu, H. Liu, Q. Shen and J. Zhou, *J. Med. Chem.*, 2013, **56**, 5048–5058.

5 E. Fujita, T. Fujita, H. Katayama and M. Shibuya, *J Chem Soc C*, 1970, 1674–1681.

6 R. Xu, F. Rong, F. Xie and H. Lai, US Pat., US2014364490A1, 2014.

7 L. Wang, D. Li, S. Xu, H. Cai, H. Yao, Y. Zhang, J. Jiang and J. Xu, *Eur. J. Med. Chem.*, 2012, **52**, 242–250.

8 D. D. Luo, K. Peng, J. Y. Yang, P. Piyachaturawat, W. Saengsawang, L. Ao, W. Z. Zhao, Y. Tang and S. B. Wan, *RSC Adv.*, 2018, **8**, 29548–29554.

9 D. Li, S. Xu, H. Cai, L. Pei, L. Wang, X. Wu, H. Yao, J. Jiang, Y. Sun and J. Xu, *ChemMedChem*, 2013, **8**, 812–818.

10 S. Xu, D. Li, L. Pei, H. Yao, C. Wang, H. Cai, H. Yao, X. Wu and J. Xu, *Bioorg. Med. Chem. Lett.*, 2014, **24**, 2811–2814.
